# Rapid Scaling Up of Insecticide-Treated Bed Net Coverage in Africa and Its Relationship with Development Assistance for Health: A Systematic Synthesis of Supply, Distribution, and Household Survey Data

**DOI:** 10.1371/journal.pmed.1000328

**Published:** 2010-08-17

**Authors:** Abraham D. Flaxman, Nancy Fullman, Mac W. Otten, Manoj Menon, Richard E. Cibulskis, Marie Ng, Christopher J. L. Murray, Stephen S. Lim

**Affiliations:** 1 Institute for Health Metrics and Evaluation, University of Washington, Seattle, Washington, United States of America; 2 Surveillance, Monitoring, and Evaluation, Global Malaria Programme, World Health Organization, Geneva, Switzerland; 3 Malaria Branch, Division of Parasitic Diseases, Centers for Disease Control and Prevention, Atlanta, Georgia, United States of America; Papua New Guinea Institute of Medical Research, Papua New Guinea

## Abstract

**Background:**

Development assistance for health (DAH) targeted at malaria has risen exponentially over the last 10 years, with a large fraction of these resources directed toward the distribution of insecticide-treated bed nets (ITNs). Identifying countries that have been successful in scaling up ITN coverage and understanding the role of DAH is critical for making progress in countries where coverage remains low. Sparse and inconsistent sources of data have prevented robust estimates of the coverage of ITNs over time.

**Methods and Principal Findings:**

We combined data from manufacturer reports of ITN deliveries to countries, National Malaria Control Program (NMCP) reports of ITNs distributed to health facilities and operational partners, and household survey data using Bayesian inference on a deterministic compartmental model of ITN distribution. For 44 countries in Africa, we calculated (1) ITN ownership coverage, defined as the proportion of households that own at least one ITN, and (2) ITN use in children under 5 coverage, defined as the proportion of children under the age of 5 years who slept under an ITN. Using regression, we examined the relationship between cumulative DAH targeted at malaria between 2000 and 2008 and the change in national-level ITN coverage over the same time period. In 1999, assuming that all ITNs are owned and used in populations at risk of malaria, mean coverage of ITN ownership and use in children under 5 among populations at risk of malaria were 2.2% and 1.5%, respectively, and were uniformly low across all 44 countries. In 2003, coverage of ITN ownership and use in children under 5 was 5.1% (95% uncertainty interval 4.6% to 5.7%) and 3.7% (2.9% to 4.9%); in 2006 it was 17.5% (16.4% to 18.8%) and 12.9% (10.8% to 15.4%); and by 2008 it was 32.8% (31.4% to 34.4%) and 26.6% (22.3% to 30.9%), respectively. In 2008, four countries had ITN ownership coverage of 80% or greater; six countries were between 60% and 80%; nine countries were between 40% and 60%; 12 countries were between 20% and 40%; and 13 countries had coverage below 20%. Excluding four outlier countries, each US$1 per capita in malaria DAH was associated with a significant increase in ITN household coverage and ITN use in children under 5 coverage of 5.3 percentage points (3.7 to 6.9) and 4.6 percentage points (2.5 to 6.7), respectively.

**Conclusions:**

Rapid increases in ITN coverage have occurred in some of the poorest countries, but coverage remains low in large populations at risk. DAH targeted at malaria can lead to improvements in ITN coverage; inadequate financing may be a reason for lack of progress in some countries.

*Please see later in the article for the Editors' Summary*

## Abstract 2

Stephen Lim and colleagues use several sources of data to estimate the changes in distribution of insecticide-treated bed nets across Africa between 2000 and 2008, and to analyze the link between development assistance and net coverage.

## Editors' Summary

**Background:**

Malaria is a major global public-health problem. Nearly half of the world's population is at risk of this parasitic disease, which kills about one million people (mainly children living in sub-Saharan Africa) every year. Malaria is transmitted to people through the bites of infected night-flying mosquitoes. Soon after entering the human body, the parasite begins to replicate in red blood cells, bursting out every 2–3 days and infecting more red blood cells. The parasite's presence in the bloodstream causes malaria's characteristic fever and can cause fatal organ damage. Malaria can be prevented by controlling the mosquitoes that spread the parasite and by sleeping under insecticide-treated bed nets (ITNs) to avoid mosquito bites. In trials, ITN use reduced deaths in young children by about 20%. Consequently, the widespread provision of ITNs is a mainstay of the World Health Organization's efforts to control malaria and in 2005 the World Assembly agreed to a target of providing ITNs for 80% of the people at risk of malaria by 2010.

**Why Was This Study Done?:**

Development assistance for health (DAH) targeted at malaria has increased considerably over the past decade. Much of this resource has been directed toward increasing ITN coverage, but has it been used effectively? To answer this question and to track progress toward universal ITN provision, reliable estimates of ITN coverage are critical. Most attempts to quantify ITN coverage have relied on single sources of data such as manufacturers' records of ITNs supplied to individual countries, National Malaria Control Program reports on ITN distribution, or household surveys of ITN use. Because each of these data sources has weaknesses, robust estimates of ITN coverage over time cannot be calculated from a single data source. In this study, the researchers combine data from these three sources to calculate ITN ownership coverage (the proportion of households owning at least one ITN) and ITN use in children under 5 coverage (the proportion of children under the age of 5 years sleeping under an ITN) for 44 African countries between 1999 and 2008. They also investigate the relationship between changes in ITN coverage and the cumulative DAH targeted for malaria for each country over this period.

**What Did the Researchers Do and Find?:**

The researchers combined the three sources of data by applying a statistical method called Bayesian inference to a “deterministic compartmental model” of ITN distribution, a flow chart that represents ITN movement into and within countries. In 1999, the researchers estimate, ITN ownership and ITN use by young children were uniformly low across the 44 countries. On average, only 2.2% of households owned ITNs and only 1.5% of young children slept under bed nets. By 2008, 32.8% of households owned ITNs and 26.6% of young children slept under ITNs but there were now large differences in ITN coverage between countries. In four countries, 80% or more of households owned an ITN but in 13 countries (including Nigeria), ITN ownership was below 20%. Finally, the researchers used a statistical technique called regression to reveal that the estimated increase in national ITN coverage between 2000 and 2008 was strongly related to the cumulative national DAH targeted for malaria (calculated by identifying all the grants and loans provided for malaria control) over the same period.

**What Do These Findings Mean?:**

The accuracy of these findings depends on the assumptions included in the model of ITN distribution and the quality of the data fed into it. Nevertheless, this systematic analysis provides new insights into the progress of ITN provision in Africa and a robust way to monitor future ITN coverage. Its findings show that several countries, even some very poor countries, have managed to scale up their ITN coverage from near zero to above 60%. However, because countries such as Nigeria that have large populations at risk of malaria continue to have low ITN coverage, Africa as a whole falls far short of the target of 80% ITN coverage by 2010. Finally, the clear relationship between the expansion of DAH targeted at malaria and increased ITN coverage suggests that inadequate funding may be responsible for the lack of progress in some countries and indicates that continued external financial assistance will be required to maintain the improvements in ITN coverage that have already been achieved.

**Additional Information:**

Please access these Web sites via the online version of this summary at http://dx.doi.org/10.1371/journal.pmed.1000328.

- Further information is available on the Institute for Health Metrics and Evaluation at the University of Washington
- Information is available from the World Health Organization on malaria (in several languages); the 2009 World Malaria Report provides details of the current global malaria situation
- The US Centers for Disease Control and Prevention provide information on malaria and on insecticide-treated bed nets (in English and Spanish)
- Information is available from the Roll Back Malaria Partnership on its approach to the global control of malaria including fact sheets on malaria in Africa and on insecticide-treated bed nets (in English, French and Portuguese)
- MedlinePlus provides links to additional information on malaria (in English and Spanish)

## Introduction

In 2006, the World Health Organization (WHO) estimated that malaria caused about 900,000 deaths, with the vast majority of these in children under the age of 5 years in Africa [1]. Reductions in malaria incidence and mortality are included as part of Millennium Development Goal Six. Over the last decade, development assistance for health (DAH) targeted at reducing the burden of malaria has risen dramatically [2],[3]. Before 1998, DAH disbursed for malaria was less than US$40 million per annum. Through new initiatives such as the Global Fund to Fight AIDS, Tuberculosis and Malaria, this increased to US$724 million in 2007 [2]. While a number of interventions can reduce the burden of malaria, attention has focused on insecticide-treated bed nets (ITNs). ITNs have been shown in randomized controlled trials to be efficacious in reducing mortality [4]. Long-lasting insecticide-treated bed nets (LLINs) have also removed the need for regular net retreatment. As a result, many countries have switched to distributing LLINs rather than conventional ITNs. At the 2005 World Health Assembly, member states agreed to raise ITN coverage to 80% by 2010 [5],[6], increasing the previous target of 60% set in Abuja in 2000 [7].

In order to understand progress toward goals and the effectiveness of DAH, it is critical to have valid, reliable, comparable, and timely measurements of ITN coverage. Existing studies that have quantified ITN coverage have several limitations, a common one being a reliance on a single type of data. WHO in its 2008 World Malaria Report [1] used annual reports from National Malaria Control Programs (NMCPs) on ITN distribution to health facilities and operational partners to estimate ITN operational coverage by assuming that one ITN distributed is needed for every two persons. Milliner [8] assumes the same net-to-person ratio but uses the number of LLINs delivered by manufacturers to countries. Manufacturer and NMCP reports capture important aspects of the net distribution system; however, they do not accurately measure the number of ITNs that ultimately reach or are used by households. Noor and colleagues [9] rely on estimates of ITN use in children under the age of 5 from household survey reports to estimate coverage for two time periods: 2000 to 2003 and 2004 to 2007, with linear extrapolation between available data to estimate coverage in 2007. This approach is limited by the infrequency of household surveys and hence may not reflect recent trends in ITN supply or distribution evident from NMCP or manufacturer data. Additionally, LLINs are often distributed through national campaigns, which cause a sharp increase in coverage that cannot be predicted by linear extrapolation.

Combining multiple sources of data uses the strengths of one source to counteract the weaknesses of another, potentially improving the accuracy and reducing uncertainty in estimates of coverage. Survey data directly measure the outcome of interest—the number of ITNs owned and used—and are therefore considered the “gold standard.” Survey data, however, are available only for a limited number of country-years. Manufacturer and NMCP data, on the other hand, are reported annually but do not capture the outcome of interest directly. Triangulating data sources to estimate ITN coverage will allow one to identify countries that are on track to meet coverage goals and those that are lagging behind. Timely measurement is critical given the rapid pace in which programs have been distributing ITNs. Comparable estimates also allow one to explore determinants of ITN coverage; for example, are efforts to increase ITN coverage limited by inadequate financial resources? Answers to these questions are also central to the sustainability of malaria control efforts given that LLINs must be replaced periodically; the WHO Pesticide Evaluation Scheme (WHOPES) recommends that LLINs should be replaced every 3 years [10]–[12]. ITN coverage estimates are also crucial for estimating the population impact of ITNs in reducing the burden of malaria.

In this study, we combine all available data from manufacturer reports, NMCP reports, and household surveys to systematically assess: (i) the trend over time in ITN ownership coverage and ITN use in children under 5 coverage with uncertainty intervals for the period 1999 to 2008 in 44 malaria-endemic countries in Africa; and (ii) the relationship between changes in ITN coverage between 2000 and 2008 and cumulative DAH targeted at malaria disbursed to countries over the same period.

## Methods

### Definitions

We systematically assessed two measures of ITN coverage: (i) ITN ownership, defined as the proportion of households that own at least one ITN; and (ii) ITN use in children under 5, defined as the proportion of children under the age of 5 years sleeping under an ITN the previous night during the wet season. Coverage was assessed for 44 African countries.

### Data Sources

Available data sources on ITN distribution and coverage fall into three categories. The first is data collected by the AMP (Alliance for Malaria Prevention) on the number of LLINs delivered from 2004 to 2008 by the following manufacturers: Sumitomo/A-Z, Vestergaard-Frandsen, Clarke, BASF, Intection, Tana Netting, and Yorkool [9]. Although we do not know precisely the percentage of all LLINs that are represented by these manufacturers, we believe this data source accounts for almost all LLINs that are supplied to these 44 countries.

The second category is data collected by WHO on the number of ITNs that are reported to be distributed to health facilities and operational partners by NMCPs from 1999 to 2008. Numbers are provided separately for LLINs and conventional ITNs and are intended to reflect distribution by all relevant agencies, including the Ministry of Health (MOH) and nongovernmental organizations. NMCP reports constitute only an indirect measure of the number of nets received by households, and are prone to bias. Reported numbers reflect the number distributed from the central level to outlying administrative units or operational partners and not the number reaching households. ITNs distributed may be double-counted if multiple agencies are involved in distribution. Not all agencies may be included, and in most cases reports do not include purchase of nets by households. We were not always able to verify the accuracy of reports from NMCPs; some numbers were rounded, indicating imprecise record keeping. Reports may also be biased due to pressure to meet coverage targets [13].

The third category of data is from household surveys. This includes standardized multi-country surveys such as the Demographic and Health Surveys (DHS), Multiple Indicator Cluster Surveys (MICS), and Malaria Indicator Surveys (MIS), as well as country-specific surveys. Depending on the survey, national ITN ownership coverage can be computed directly; in many surveys, the number of nets owned by the household is based on visual confirmation by the interviewer. Nets present are classified as LLINs by asking about or by visual confirmation of the brand. Nets were classified as conventional ITNs if they were a non-LLIN brand and reported to have been treated or newly acquired within the last year. We can also determine the number of LLINs or ITNs received each year based on household reports for up to three years prior to the survey, but this quantity does not include nets received and then discarded before the time of the survey.

**Figure 1:**
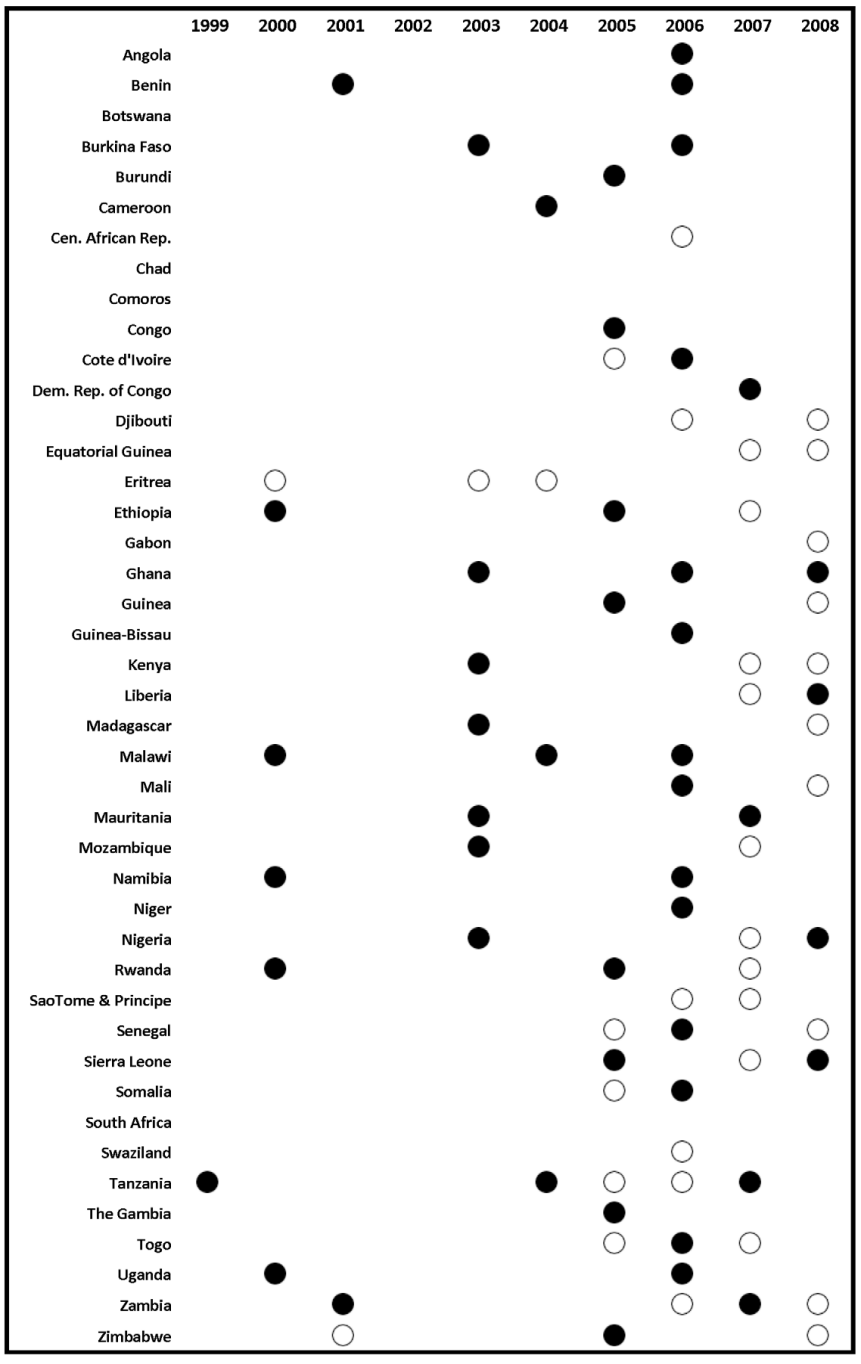
Survey and survey report availability by country and year for household ITN ownership. Solid circles indicate surveys with micro-data available; non-solid circles indicate surveys with estimates available from survey reports.

**Figure 2:**
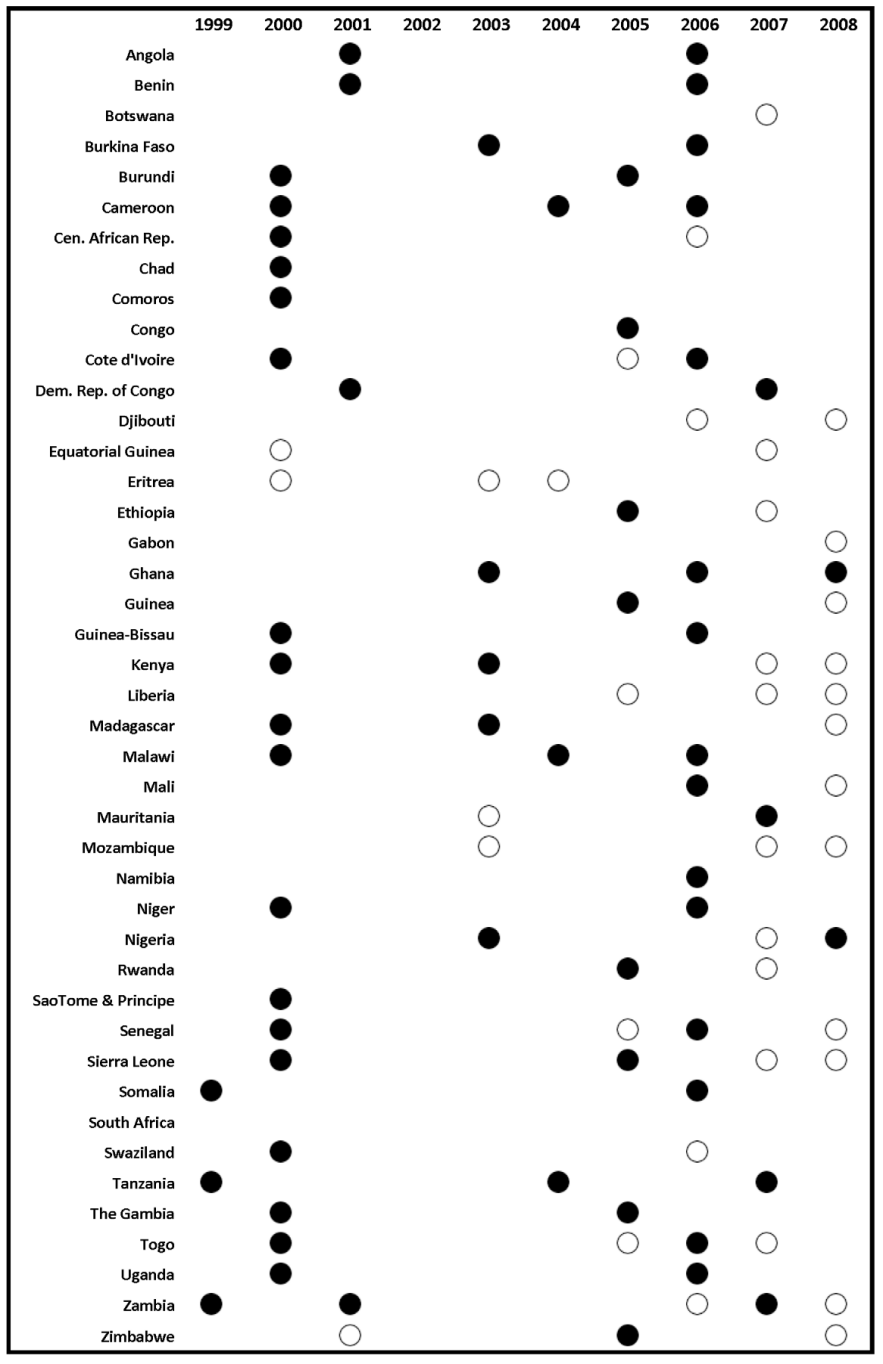
Survey and survey report availability by country and year for under-5 ITN use. Solid circles indicate surveys with micro-data available; non-solid circles indicate surveys with estimates available from survey reports.

Surveys also allow us to compute ITN use in children under 5 coverage based on reports by the mother on whether the child slept under a net the previous night. We analyzed unit-record data for 48 household surveys, including 32 DHS, 13 MICS, and three MIS, taking into account the multi-stage sampling design. We also extracted information from survey reports where available; this included six DHS, 28 MICS, ten MIS, and 21 country-specific surveys (Figures 1 and 2). Additional information on three surveys was obtained based on MOH reports to WHO.

### Estimating ITN Distribution and Coverage over Time

There are sparse data on LLIN and ITN ownership and use by children under the age of 5 from surveys. The challenge is to impute missing survey-based coverage that also appropriately characterizes uncertainty. The method used must also resolve the issue that available data capture the stock and flow of nets at different points of the supply and distribution chain. For example, surveys measure the stock of nets in households at a specified time, whereas manufacturer data represent flows to a country over a one-year time period.

### Compartmental Model

**Figure 3:**
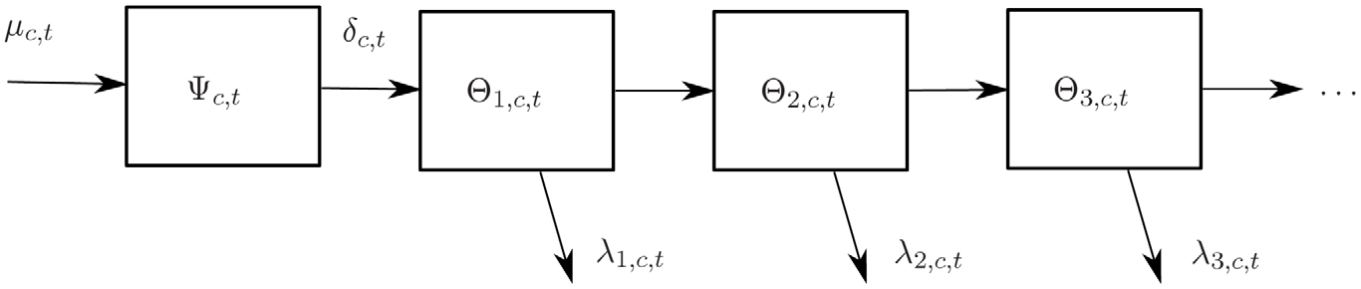
Stock-and-flow model of the LLIN distribution system within a country. μ*_c,t_* indicates the number of LLINs supplied to country *c* by manufacturers during year *t*; ψ*_c,t_* indicates the number of LLINs available for distribution or purchase that are in a country *c* in year *t* but are not in households, e.g. in warehouses, retail stores; δ*_c,t_* indicates the number of LLINs that are received or purchased by households in country *c* during year *t*; θ_1,*c,t*_ indicates the number of 0- to 1-year-old LLINs in households in country *c* during year *t*; θ_2,*c,t*_ indicates the number of 1- to 2-year-old LLINs in households in country *c* during year *t*; θ_3,*c,t*_ indicates the number of 2- to 3-year-old LLINs in households in country *c* during year *t*; λ_1,*c,t*_ indicates the number of 0- to 1-year-old LLINs discarded by households in country *c* during year *t*; λ_2,*c,t*_ indicates the number of 1- to 2-year-old LLINs discarded by households in country *c* during year *t*; λ_3,*c,t*_ indicates the number of 2- to 3-year-old LLINs discarded by households in country *c* during year *t*.

We developed a deterministic compartmental model and applied Bayesian inference to estimate ITN ownership coverage over time. UNAIDS uses a similar method for their widely used estimates of HIV prevalence [14],[15]. Detailed information on the model is provided in Text S1. Briefly, the model uses the precise relationship between supply, distribution, and ownership over time; for example, for a net to be owned by a household it must have been distributed or purchased sometime in the past, and before that, it must have been manufactured and sent to organizations responsible for distribution, or to the commercial sector for purchase. We reflect this by using a compartmental model describing the supply, distribution, ownership, and discard of nets by households (Figure 3). The supply compartment reflects both public and commercial supply, and distribution includes public distribution as well as the purchase of nets by households from the commercial sector. The model uses a discrete one-year time step and allows flows into a compartment to be part of flows out of the compartment for the same year. This model ensures that estimates of supply, distribution, ownership, and discard of nets are consistent with one another over time. Compartmental model parameters are limited to LLINs as manufacturer data is available for LLINs only. We also do not include conventional ITNs as they must be retreated at least annually; as a result, in a compartmental model with a one-year time step the stock of conventional ITNs (i.e. the number present in households for a given year) is essentially equivalent to the flow of conventional ITNs (i.e. the number purchased, received, or retreated by households in that year). Based on previous studies [10]–[12],[16], the primary assumption is that after three years, an LLIN is no longer active and is not included in the household stock.

The compartmental model estimates the total number of LLINs in households in each country over time. We add to this a parameter that accounts for conventional ITNs in households to determine the total number of ITNs in households. We estimate the number ITNs in households per capita in each country by dividing by the estimated total population [17]. The fraction of households with zero, one, two or three or more ITNs we model as negative-binomially distributed. This distribution was found to fit well to available survey data (average root mean squared error [RMSE] = 0.32%; Text S1). ITN ownership coverage is calculated as one minus the fraction of households with zero ITNs.

### Statistical Model

Parameters of the model are estimated using Bayesian inference, by inverting the likelihood of observing the data given the relevant parameters [18]. This allows us to combine all available data on parameters for a given country with prior beliefs about parameters. We use empirical priors, based on observations of parameter values across all countries, to borrow strength in countries with sparse data. This approach also provides a way of assessing uncertainty about the inputs and outputs of the model.

The total likelihood function is the product of five individual data likelihoods: (1) manufacturer data on the number of LLINs delivered to each country; (2) number of LLINs received or purchased by households up to three years prior to the survey. As this is based on the stock of nets at the time of the survey, we include an adjustment that accounts for nets that were purchased or received in the three years prior to the survey but discarded before the time of the survey; (3) NMCP data on the number of LLINs distributed. We include a parameter that captures the relative bias in NMCP reports to account for potential bias as described earlier; (4) LLIN ownership coverage calculated from survey data or from survey reports; and (5) ITN ownership coverage calculated from survey data or from survey reports.

We include non-informative priors (Text S1) for the parameters with the following exceptions. For the net discard parameter, we use an empirical prior based on published studies (Table S1) that suggest that, on average, around 4% of nets are discarded per year. For the mean and dispersion parameters of the negative binomial distribution used to estimate the household ITN distribution, we use an empirical prior based on available survey data. For the bias and error parameter for NMCP data, we use an empirical prior based on the observed relationship between survey data on the number of nets received or purchased by households and NMCP reports (see Text S1 for further details). These data suggest that, on average, NMCP reports overestimate the number of nets that are received or purchased by households by approximately two-and-a-half times. We include two additional priors based on expert judgment: our proven capacity prior captures the notion that, if a given quantity of nets was distributed in a year, then it is unlikely that many fewer nets will be distributed in future years (provided that nets are available for distribution); our ITN composition prior is used to capture the belief that in more recent years, most of the ITNs distributed are LLINs, while in earlier years most of the ITNs are non-LLINs.

We used Markov chain Monte Carlo (MCMC), implemented in Python by the PyMC package [19], to draw 2,000 samples from the model posterior to estimate the mean and 95% uncertainty intervals for all parameters of interest. The model is fit to each country separately and therefore produces a set of posterior distributions for all parameters that are specific to each country.

### ITN Use in Children under 5

A potential confounding factor in determining use in children under 5 is the season in which surveys are conducted; individuals are more likely to sleep under ITNs during times of the year when the risk of mosquito bites is higher [20]–[22]. We used mixed-effects regression to estimate ITN use in children under 5 from ITN ownership coverage, the proportion of the total population that are children under 5 and controlling for the season (wet or dry; as defined by the Mapping Malaria Risk in Africa project, http://www.mara.org.za/) in which the survey was conducted (100 surveys). We include a random coefficient on ITN ownership coverage and estimate the posterior value to capture country-level variation in the relationship between ITN ownership and ITN use in children under 5. Predicted ITN use in children under 5 coverage showed a good fit with the observed data (RMSE = 7.13%). The regression parameters are then applied to the Bayesian inference-based compartmental model estimates of ITN ownership coverage to predict ITN use in children under 5 during the wet season. MCMC (2,000 draws) was used to reflect uncertainty due to the model estimates of ITN ownership coverage and both parameter and fundamental uncertainty in the regression model [23].

### National and Regional-Level ITN Coverage in Populations at Risk of Malaria

We provide two sets of estimates of ITN coverage in this paper. First, we estimate ITN coverage—both ITN ownership coverage and ITN use in children under 5 coverage—at the national level. This is conventional practice in household surveys. In countries with less than 100% of the population at risk of malaria, the denominator in this estimate includes both populations at risk of malaria and populations that are not. As a measure of ITN coverage among populations at risk of malaria in these countries, this assumes that ITNs are equally distributed between populations at risk of malaria and populations not at risk of malaria. Second, we estimate ITN coverage in populations at risk of malaria (PAR), assuming that ITNs are owned and used only in populations that are at risk of malaria. The PAR (Table S2) was determined as the fraction of the population at risk of malaria in 2006 based on reports by countries to WHO [1] multiplied by UN population estimates [17]. The second set of estimates applies to only 12 out of the 44 countries with an estimated fraction of the population at risk of malaria of less than 100%. For both sets of estimates of ITN coverage, we calculate mean ITN coverage among populations at risk of malaria across the 44 countries.

### Relationship between Changes in ITN Ownership Coverage and Development Assistance for Health

Using ordinary least squares (OLS) regression we examined the relationship between the absolute change in national-level ITN ownership coverage and ITN use in children under five between 2000 and 2008 and cumulative DAH disbursed per capita targeted toward malaria for the same time period. DAH for malaria was derived from the disaggregation of total global aid flows as previously described [2]. Briefly, a project-level database on grant and loan information from bilateral agencies, the European Commission, the Global Fund to Fight AIDS, Tuberculosis and Malaria, the GAVI Alliance, the World Bank, the Asian Development Bank, the Inter-American Development Bank, and the Bill & Melinda Gates Foundation was compiled. Disease-specific grants and loans were identified using a keyword search of descriptive fields. Keywords for malaria-specific DAH were: malaria, paludisme, *Plasmodium falciparum*, *Anopheles*, bednets, insecticide, artemisinin, indoor residual spraying. It is important to note that grants and loans from UN agencies, NGOs other than the Bill & Melinda Gates Foundation, and non-OECD bilateral aid are not allocable into disease-specific DAH, as project-level detail is not available for these funders. We controlled for GDP per capita and all other DAH; this includes both DAH targeted at other diseases, such as HIV/AIDS, and DAH that was not allocable to specific diseases. In addition, we also examined the relationship between malaria DAH and changes in ITN coverage excluding four outliers (Ethiopia, The Gambia, Mali, and São Tomé and Principe) identified using a bivariate analog of the boxplot [24]. In order to obtain accurate confidence intervals for the regression coefficients, we used simulation to reflect the uncertainty in the estimates of ITN coverage from the model as well as the uncertainty in the estimation of the regression parameters [23].

Analyses were conducted in Stata 11.0 (Stata Corporation, Texas, USA), R 2.10.1 (The R Foundation for Statistical Computing, Vienna, Austria), and Python 2.5.2 (Python Software Foundation, Hampton, New Hampshire, USA).

## Results

### Examples of Data and Model Estimates

**Figure 4:**
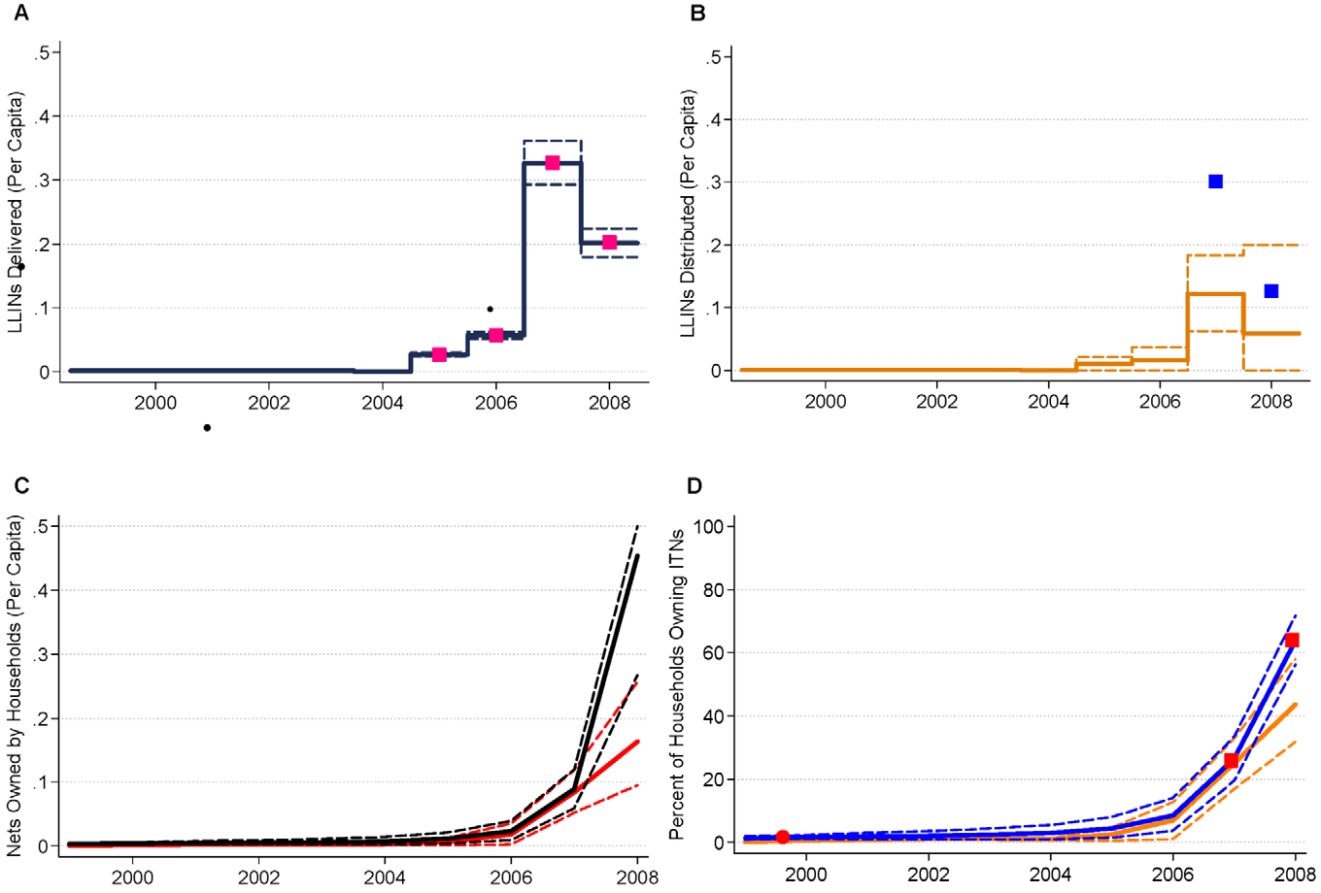
Data and model estimates for Equatorial Guinea at the national level. (A) LLINs per capita delivered to countries. Data are from manufacturer reports (pink squares). Model estimates with 95% uncertainty intervals are shown as black lines; (B) LLINs per capita distributed. Data are from National Malaria Control Program reports (blue squares). Model estimates with 95% uncertainty intervals are shown as orange lines; (C) ITNs and LLINs per capita in households. Model estimates with 95% uncertainty intervals are shown as black lines for ITNs and red lines for LLINs; (D) ITN and LLIN ownership coverage. Data are predicted from under 5 use coverage from surveys (red circles for ITN coverage); and from survey reports from countries to WHO (red squares for ITN coverage). Model estimates with 95% uncertainty intervals are shown as blue lines for ITNs, orange lines for LLINs. Years indicate the mid-point of the calendar year.

**Figure 5:**
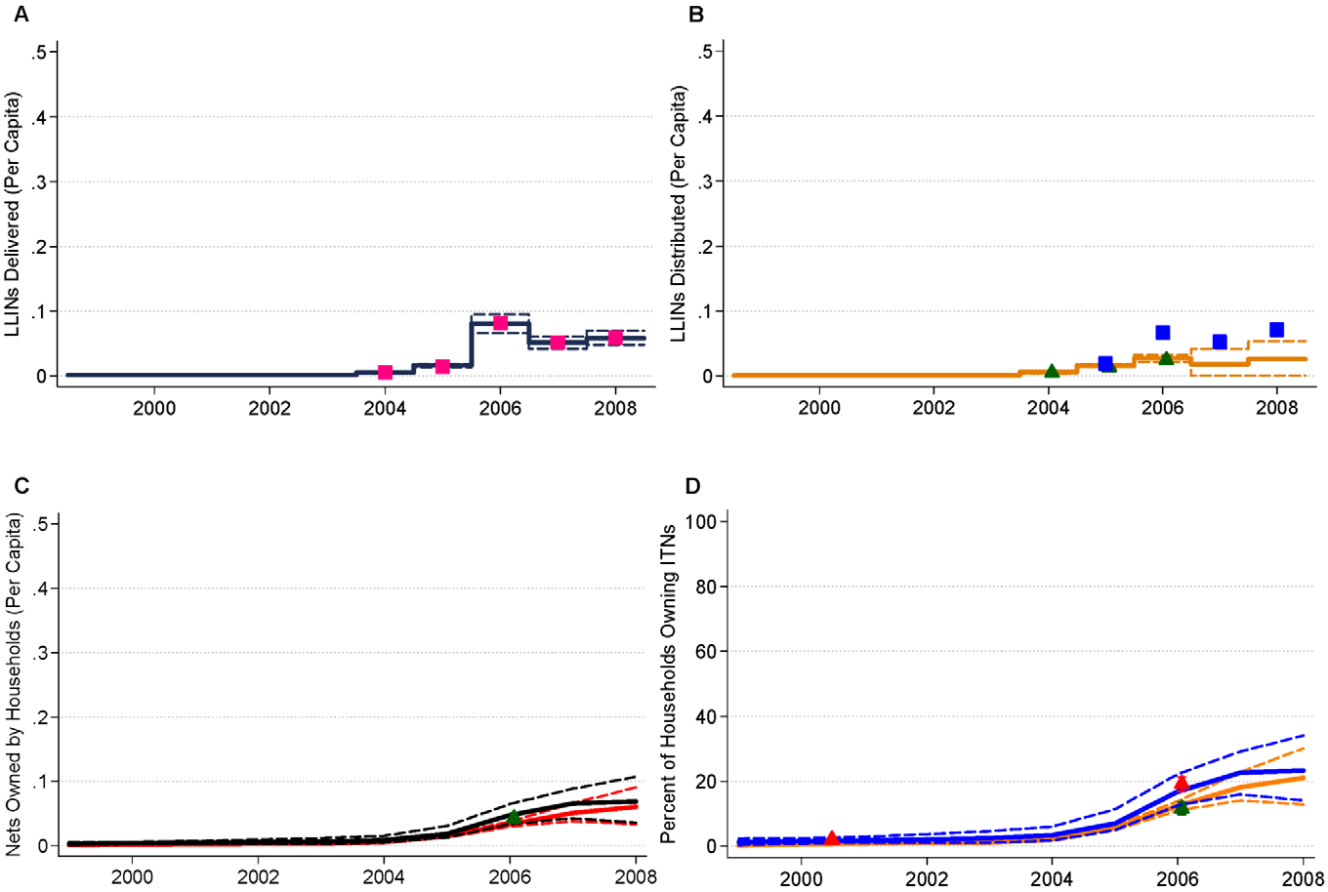
Data and model estimates for Uganda at the national level. (A) LLINs per capita delivered to countries. Data are from manufacturer reports (pink squares). Model estimates with 95% uncertainty intervals are shown as black lines; (B) LLINs per capita distributed. Data are from National Malaria Control Program reports (blue squares); and calculated from surveys (green triangles). Model estimates with 95% uncertainty intervals are shown as orange lines; (C) ITNs and LLINs per capita in households. Data are calculated from surveys (green triangles for LLINs). Model estimates with 95% uncertainty intervals are shown as black lines for ITNs and red lines for LLINs; (D) ITN and LLIN ownership coverage. Data are calculated from survey data (green triangles for LLIN coverage, red triangles for ITN coverage). Model estimates with 95% uncertainty intervals are shown as blue lines for ITNs, orange lines for LLINs. Years indicate the mid-point of the calendar year.

Figures 4 and 5 show national-level data from manufacturers, NMCPs, household surveys, and model estimates with 95% uncertainty intervals for the supply, distribution, and coverage of ITNs and LLINs in Equatorial Guinea and Uganda. These two examples are illustrative of the sparse data available from surveys on ITN ownership coverage and the value of reconciling inconsistent data from manufacturers, NMCPs, and household surveys. For example, in 2007 in Equatorial Guinea, there were a large number of LLINs supplied by manufacturers (Figure 4A), and the NMCP reported distributing a large number in the same year to health facilities and operational partners (Figure 4B). The reported supply and distribution of ITNs was not fully reflected in increases in the stock of ITNs in households (Figure 4C) or ITN ownership coverage until 2008, as indicated by household survey data in the middle of 2007 and at the end of 2008 (Figure 4C and 4D). The example of Equatorial Guinea also emphasizes the importance of timely monitoring, given that the increases in coverage have occurred over a very short period.

Uganda (Figure 5) further highlights the ability of the model to provide internally consistent, empirically based estimates of coverage in the presence of sparse survey data by reflecting the information content in data from manufacturers and NMCP reports. This is most evident for the 2007 and 2008 time periods. The most recent household survey for Uganda was conducted in 2006 with ITN coverage of 19.1% and LLIN ownership coverage of 11.6% (Figure 5D). NMCP reports of the number of LLINs delivered to health facilities and operational partners in 2006 over-estimated the number received by households based on survey data. (Figure 5B). This is then reflected in estimates of ITN ownership coverage of 22.6% (15.9% to 29.1%) for 2007 and 23.3% (14.1% to 34.0%) for 2008 (Figure 5D). The widening of the uncertainty interval of ITN ownership coverage for these time points reflects the time since the last survey data point—the further away from the survey the greater the uncertainty—as well as the uncertainty, due to both bias and error, in the NMCP reports of nets distributed. As the model is fit to all available data for a given country, survey estimates are highly concordant with the results from the model across all countries (concordance correlation coefficient of 0.99; 95% confidence interval 0.98 to 0.99).

### ITN Ownership Coverage, 1999 to 2008

**Figure 6:**
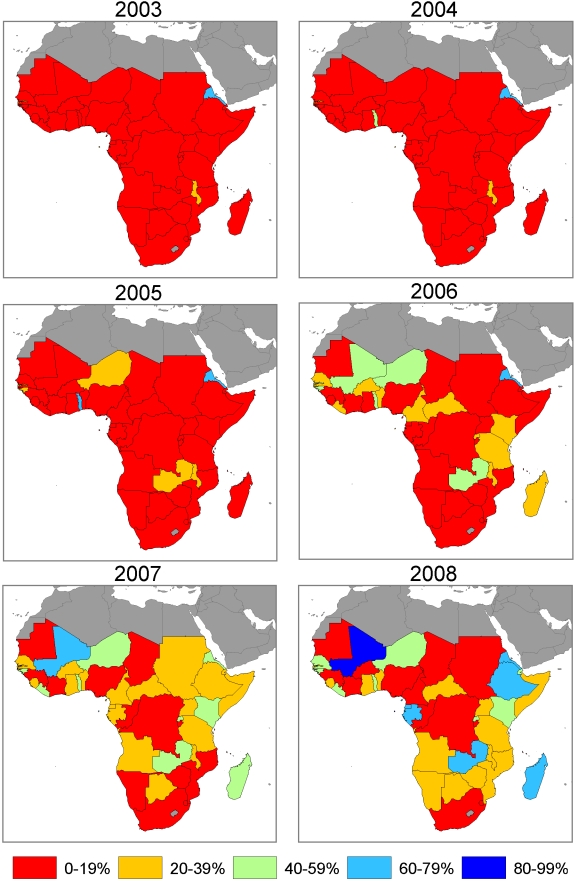
Annual maps of ITN household ownership coverage at the national level in 44 African countries.

**Figure 7:**
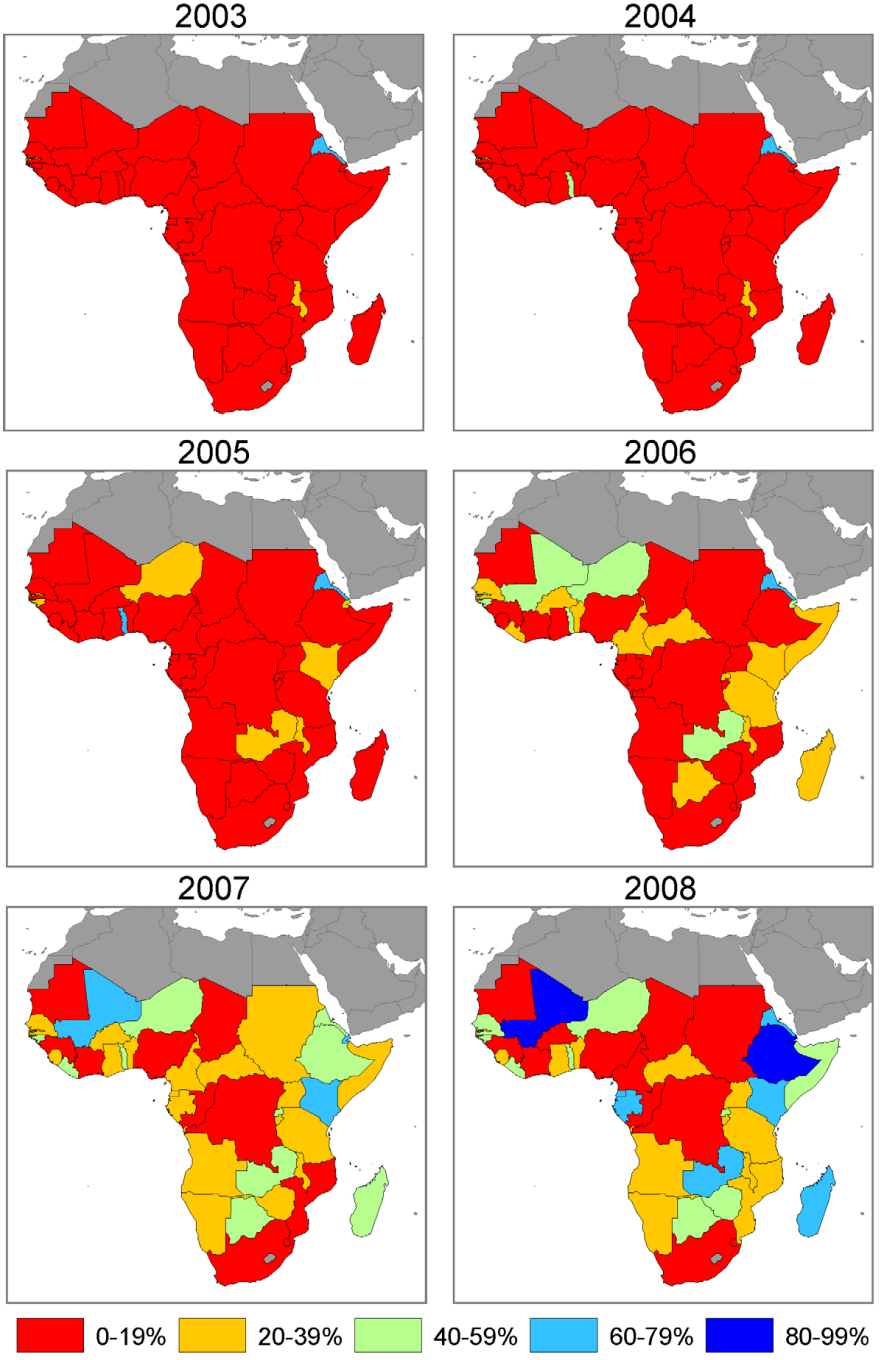
Annual maps of ITN household ownership coverage among the population at risk of malaria in 44 African countries. Coverage is estimated in the population at risk of malaria by assuming that ITNs are owned only in populations that are at risk of malaria.

Figure S1 shows country plots of ITN ownership coverage from surveys and model estimates with 95% uncertainty intervals over the period 1999 to 2008 at the national level and in the population at risk of malaria. The diversity of progress and the rapidity of the scale-up in some countries can be clearly seen by examining annual maps of ITN ownership coverage from 2003 to 2008. Figure 6 shows national-level estimates; and Figure 7 shows estimates in the population at risk of malaria assuming that ITNs are owned only in populations at risk of malaria. In 2003, only Eritrea, Malawi, The Gambia, and São Tomé and Principe had ITN ownership coverage greater than 20% with Eritrea already above 60% coverage. In 2004, Togo scaled up coverage above 20% and in 2005 was joined by countries such as Guinea-Bissau, Niger, and Zambia. By 2006, continued progress in countries such as Niger, Guinea-Bissau, Togo, and Zambia saw coverage rise above 40%, while coverage was above 20% in a host of other countries such as Benin, Liberia, Madagascar, Rwanda, and Tanzania. The most dramatic improvements in coverage were seen in 2007 and 2008; for example, rapid increases in coverage occurred in Equatorial Guinea, Ethiopia, Liberia, Madagascar, Mali, Rwanda, and São Tomé and Principe (Figure S1, 6, and 7). While improvements were seen in almost all countries, a number of countries continue to have coverage below 20% (Figures 6 and 7). By the middle of 2008, two countries—São Tomé and Principe, and Mali—had national-level ITN ownership coverage of 80% or greater; six countries were between 60% and 80%; eight countries were between 40% and 60%; 14 countries were between 20% and 40%; and 14 countries had coverage below 20%. Assuming that ITNs are distributed only to population at risk of malaria, four countries—Djibouti, Ethiopia, Mali, and São Tomé and Principe—had ITN ownership coverage in populations at risk of malaria of 80% or greater; six countries were between 60% and 80%; nine countries were between 40% and 60%; 12 countries were between 20% and 40%; and 13 countries had coverage below 20%.

With countries such as Nigeria and DRC having low ITN coverage and large absolute populations at risk of malaria, ITN ownership coverage across all 44 countries remains suboptimal. Assuming that ITNs are equally distributed between population at risk and not at risk of malaria, mean ITN ownership coverage across the 44 countries amongst populations at risk of malaria was 2.1% (1.7% to 2.4%) in 1999, 4.8% (4.3% to 5.4%) in 2003, increasing to 16.4% (15.5% to 17.3%) in 2006 and 29.1% (27.9% to 30.6%) in 2008. Assuming that ITNs are distributed only to populations at risk, mean ITN ownership coverage across the 44 countries was 2.2% (1.8% to 2.6%) in 1999, 5.1% (4.6% to 5.7%) in 2003, increasing to 17.5% (16.4% to 18.8%) in 2006 and 32.8% (31.4% to 34.4%) in 2008.

### ITN Use in Children under 5 Coverage, 1999 to 2008

**Figure 8:**
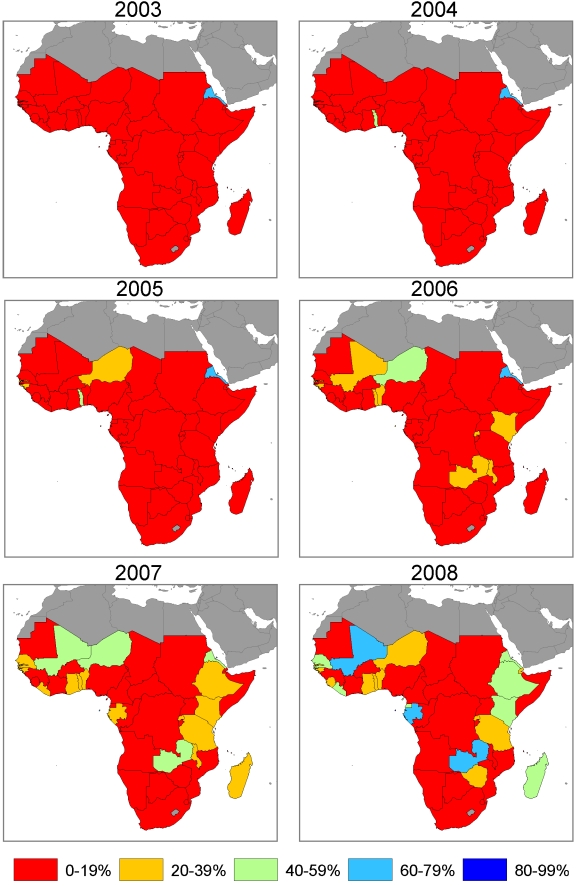
Annual maps of ITN use in children under 5 coverage at the national level in 44 African countries.

**Figure 9:**
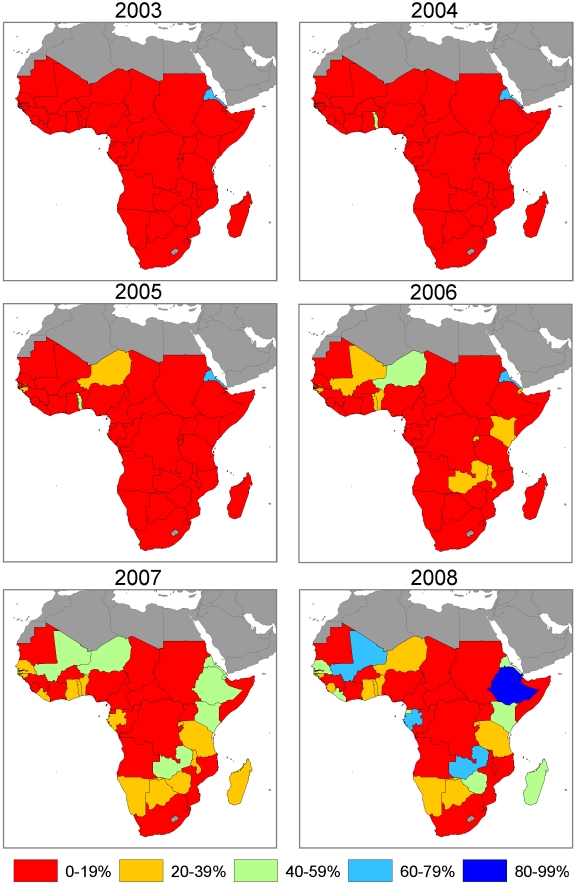
Annual maps of ITN use in children under 5 coverage among the population at risk of malaria in 44 African countries. Coverage is estimated in the population at risk of malaria by assuming that ITNs are used only in populations that are at risk of malaria.

Figure S2 shows country plots of ITN use in children under 5 coverage from surveys and model estimates with 95% uncertainty intervals over the period 1999 to 2008 at the national level and in the population at risk of malaria. As demonstrated by annual maps of ITN use in children under 5 coverage (Figures 8 and 9), the scale-up over time across countries in general mimics the pattern seen for ITN ownership coverage but at lower levels. ITN use in children under 5 coverage, on average, is about two-thirds of ITN ownership coverage.

By the middle of 2008, one country had national-level ITN use in children under 5 of 80% or greater; three countries were between 60% and 80%; six countries were between 40% and 60%; 12 countries were between 20% and 40%; and 22 countries had coverage below 20%. Assuming that ITNs are used only in populations at risk of malaria, three countries had ITN use in children under 5 coverage in populations at risk of malaria of 80% or greater; three countries were between 60% and 80%; six countries were between 40% and 60%; 12 countries were between 20% and 40%; and 20 countries had coverage below 20%.

Mean ITN use in children under 5 coverage among the population at risk of malaria in the 44 countries, assuming equal distribution of ITNs among populations at risk and not at risk of malaria was 1.3% (0.9% to 2.2%) in 1999, 3.3% (2.3% to 4.7%) in 2003, 11.5% (8.8% to 15.1%) in 2006 and 23.6% (18.4% to 29.3%) in 2008. Assuming that ITNs are used only in populations at risk, mean ITN use in children under 5 coverage across the 44 countries was 1.5% (1.1% to 2.2%) in 1999, 3.7% (2.9% to 4.9%) in 2003, increasing to 12.9% (10.8% to 15.4%) in 2006 and 26.6% (22.30% to 30.9%) in 2008.

### Relationship between Changes in ITN Ownership Coverage and Development Assistance for Health

**Figure 10:**
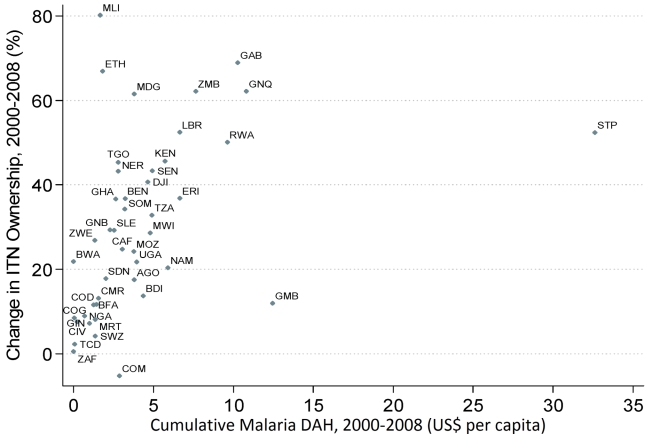
Relationship between cumulative DAH targeted at malaria (2000 to 2008) and the change in national-level ITN household ownership coverage (2000 to 2008). Full country names for the abbreviations are provided in Table S2.

Figures 9 and 10 show the observed relationship between cumulative DAH targeted at malaria for the period 2000 to 2008 and the change in both national-level ITN household ownership coverage and ITN use in children under 5 coverage over the same time period. Most countries that have received upwards of around US$5 per capita, such as Kenya, Liberia, Rwanda, Zambia, Equatorial Guinea, and São Tome and Principe, have realized large changes in ITN coverage between 2000 and 2008 (Figures 9 and 10). Substantial variation exists, however, among countries receiving less than US$5 per capita in DAH targeted at malaria. Countries such as Ethiopia, Madagascar, and Mali have achieved large changes in ITN coverage. In contrast, other countries such as Chad, Congo, Côte D'Ivoire, and Nigeria have achieved only modest increases in coverage. Controlling for GDP and all other DAH, each US$1 per capita of DAH targeted at malaria was significantly associated with an increase in ITN household ownership coverage and ITN use in children under 5 coverage of 1.5 percentage points (0.1 to 3.0) and 1.6 percentage points (0.0 to 3.3), respectively (Table 1). Excluding four outliers (Ethiopia, The Gambia, Mali, and São Tomé and Principe), increased both the strength and significance of the association between DAH and ITN coverage to 5.3 percentage points (3.7 to 6.9) for ITN household ownership coverage and 4.6 percentage points (2.5 to 6.7) for ITN use in children under 5 coverage.[Fig pmed-1000328-g011]

**Figure 11 pmed-1000328-g011:**
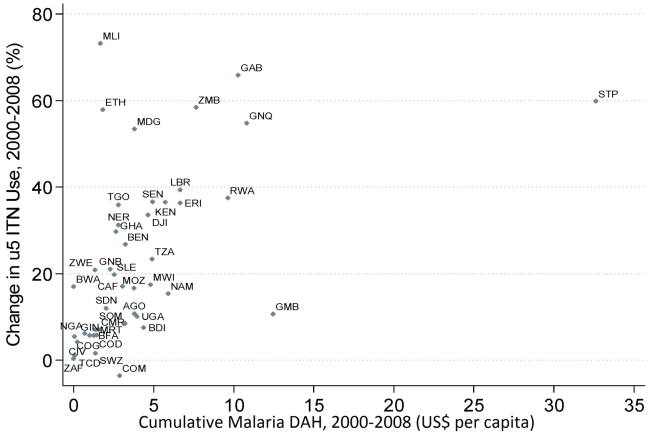
Relationship between cumulative DAH targeted at malaria (2000 to 2008) and the change in national-level ITN use in children under 5 coverage (2000 to 2008). Full country names for the abbreviations are provided in Table S2.

**Table 1:** Regression results of the effect of cumulative DAH targeted at malaria (2000 to 2008) and the change in national-level ITN household ownership coverage (2000 to 2008) and change in national-level ITN use in children under 5 coverage (2000 to 2008).

Factor	ITN Household Ownership Coverage (%)		ITN Use in Children under-5 Coverage (%)	
	All Countries (n = 44)	Excluding Outliers (n = 40)	All Countries (n = 44)	Excluding Outliers (n = 40)
Cumulative malaria DAH (US$ per capita)	1.51 (0.07 to 2.96)	5.29 (3.68 to 6.91)	1.65 (0.02 to 3.27)	4.59 (2.49 to 6.69)
Cumulative other DAH (US$ per capita)	−0.01 (−0.18 to 0.15)	0.01 (−0.10 to 0.12)	0.002 (−0.18 to 0.18)	0.02 (−0.13 to 0.16)
Log GDP per capita	−1.12 (−7.38 to 5.13)	−1.35 (−5.58 to 2.88)	−0.24 (−6.91 to 6.44)	−0.17 (−5.65 to 5.31)

Numbers are the estimated beta-coefficients with 95% uncertainty intervals reflecting uncertainty in ITN coverage and estimation of regression parameters. The four outliers excluded are Ethiopia, The Gambia, Mali, and São Tomé and Principe.

## Discussion

Our systematic analysis presents mixed findings on ITN ownership and use in children under 5 in 44 countries in Africa. On the one hand, a number of countries have been able to rapidly scale up ITN coverage from essentially zero to above 60% with the most dramatic improvements having occurred in the last two years. On the other hand, only a handful of countries have achieved ITN ownership coverage of 80% or greater. More importantly, countries with large populations at risk of malaria, such as Nigeria, continue to have low coverage, resulting in mean ITN ownership coverage in these 44 African countries of around one third, and ITN use in children under 5 coverage of around one quarter.

Efforts to increase coverage in these countries can be informed by case studies in settings where the scale-up of ITNs has been more successful. The approaches used to scale up ITN coverage in, for example, Eritrea [21],[25], Kenya [26], São Tomé and Principe [27], Tanzania [28],[29], and Zambia [30],[31] have been described in depth elsewhere. These strategies include free mass distribution, social marketing, ITN vouchers, and distribution of ITNs as part of routine antenatal care, routine immunization programs, and mass immunization campaigns. Studies to identify determinants of more recent scale-ups identified here, such as Ethiopia, Equatorial Guinea, Liberia, Mali, and Sierra Leone, can further inform strategies in those countries where coverage remains low. Notably, several countries with low levels of coverage of other key interventions have been able to scale up ITN coverage to relatively high levels. Both Niger and Ethiopia, for example, have been able to rapidly increase ITN coverage, yet Ethiopia has skilled birth attendance (SBA) coverage of only 10% while Niger has SBA coverage of less than 20% and DTP3 coverage of about 50% [13],[32],[33].

In this analysis we have quantified the relationship between DAH targeted at malaria and changes in ITN coverage. While there was substantial variation in the relationship between DAH targeted at malaria and changes in ITN coverage, our results suggest that external financial assistance can be used effectively and that increased funding could help countries with currently low levels of ITN coverage. These associations should be interpreted with caution, however, as we were not able to isolate DAH that is used specifically for ITN distribution. In other words, DAH targeted at malaria may also be used to implement indoor residual spraying (IRS) programs or purchase antimalarial medication. For example, in São Tomé and Principe—one of the identified outliers—most malaria DAH is used for IRS and case management programs rather than ITN procurement and distribution [34]. Furthermore, given that even LLINs must be replaced every few years, our results also highlight the need for these funding streams to be sustained or alternative financing to be found if ITN coverage is to be maintained into the future.

An important element of efforts to sustain ITN coverage is a precise assessment of the number of nets required to scale-up coverage and then to maintain coverage (“catch-up” versus “keep-up”). Our method can provide an accurate projection of current and future supply and distribution needs, as it tracks the number of LLINs at different points in the distribution chain, as well as the age of nets in households. This analysis extends substantially on the methods used in previous studies [1],[8],[9] that relied on a single source of data to measure the coverage of ITNs in Africa. By combining data from manufacturers, NMCPs, and household surveys, including a number of newly available surveys, we can document in a timely fashion important and substantial increases in ITN coverage in Equatorial Guinea, Liberia, Niger, Mali, Rwanda, São Tomé and Principe, Senegal, and Sierra Leone that were not previously described. We also report on ITN ownership coverage and correct for the effect of seasonality in computing use in children under 5.

While we were able to systematically assess the distribution and coverage of ITNs in African countries, a number of steps could improve the estimates shown here. Our method focuses on national-level coverage estimates. In countries with less than 100% of the population at risk of malaria, the denominator will therefore include both populations at risk and populations not at risk of malaria. As a measure of coverage this implies that ITNs are equally distributed between populations at risk and populations that are not at risk of malaria. As a sensitivity analysis, we have also estimated ITN coverage in populations at risk of malaria assuming that ITNs are exclusively owned and used in populations at risk. These two sets of estimates provide an approximation of the uncertainty of ITN coverage among population at risk of malaria given that it is unlikely that ITNs are preferentially distributed towards populations that are not at risk of malaria. It is important to note that this issue applies only to 12 out of the 44 countries with estimated populations at risk of malaria of less than 100%.

To better measure ITN coverage in populations at risk of malaria, an important direction for future work is to extend this technique to estimate ITN coverage at subnational levels paired with more robust estimates of malaria transmission risk. The estimates of the population at risk of malaria used in this analysis are based on administrative data, are estimated for a single year (2006) and are likely to be uncertain. Extending the model beyond the national level will require future collection of NMCP distribution within each country combined with household surveys that are adequately powered to estimate coverage at, for example, the province or district levels. This will also produce more locally relevant results; the model can also be adapted into software that local program managers could use to monitor and forecast ITN supply and distribution needs. Subnational estimates of ITN coverage should also be complemented by analyses of inequalities in ITN coverage related to socioeconomic indicators such as household wealth, education, and rurality [35].

We estimated the number ITNs discarded by households from a relatively small number of published studies. Additional net retention studies as well as incorporation of questions regarding past ownership of nets into routine household surveys could reduce this uncertainty. Consistent with WHOPES recommendations, we have also not included LLINs that are older than three years; studies suggest that the decline in the effectiveness of LLINs may be more gradual [36]. Improving the quality of NMCP reports would also greatly improve these estimates. Finally, work to examine the relationship between different measures of ITN coverage and health outcomes, such as parasitemia, anemia, malaria-specific mortality, or all-cause mortality, would help to resolve the debate about which measure of coverage—ownership, use, or per capita ratio—is ideal, as well as to continue to build on the available evidence [37]–[39] regarding the impact of the scale-up in ITN coverage on population health.

Large and rapid increases in ITN coverage have been realized in several countries in Africa, including some of the poorest countries with limited health infrastructure. The expansion of DAH targeted at malaria appears to be a significant contributor to these improvements. This suggests that more DAH could be effective in helping countries achieve ITN coverage goals but also that external financial assistance will need to be sustained if high coverage levels are to be maintained. When increases in ITN coverage have been made, it has often been rapid, emphasizing the importance of timely monitoring. Our method addresses this need by providing an objective and replicable way to monitor the distribution and coverage of ITNs in a continuous fashion. This is critical leading up to September 2010, when the global health community will determine whether the targets set for ITN coverage have been met.

## Supporting Information

Figure S1 Data and model estimates of ITN household ownership coverage for 44 African countries.
(0.26 MB DOC)

Figure S2 Data and model estimates of ITN use in children under 5 coverage for 44 African countries.
(0.22 MB PDF)

Table S1 Published studies used to estimate empirical prior for net discard rate.
(0.01 MB PDF)

Table S2 Total population and population at risk of malaria for 44 African countries.
(0.14 MB PDF)

Text S1 Bayesian estimation of ITN coverage.
(0.76 MB PDF)

## Abbreviations

DAH: development assistance for health
DHS: Demographic and Health Surveys
ITN: insecticide-treated bed net
LLIN: long-lasting insecticide-treated bed net
MCMC: Markov chain Monte Carlo
MICS: Multiple Indicator Cluster Surveys
MIS: Malaria Indicator Surveys
MOH: Ministry of Health
NMCP: National Malaria Control Program
PAR: populations at risk of malaria
RMSE: root mean squared error
