# Supplementary material for: Rapid Scaling Up of Insecticide-Treated Bed Net Coverage in Africa and Its Relationship with Development Assistance for Health: A Systematic Synthesis of Supply, Distribution, and Household Survey Data
Source: PLoS Med. 2010 Aug 17;7(8):e1000328. doi: 10.1371/journal.pmed.1000328 (PMC2923089; doi:10.1371/journal.pmed.1000328)
Supplement: Figure S1 — Data and model estimates of ITN household ownership coverage for 44 African countries. (0.26 MB DOC) [file pmed.1000328.s001.pdf]

Figure S1. Data and model estimates of ITN household ownership coverage for 44 African countries.
Data are: calculated from survey data (red triangles); from survey reports (red diamonds); predicted from under 5 use coverage (red circles); and reports from countries to WHO (red squares). Model estimates at the national-level are shown as blue lines with dotted lines indicating 95% uncertainty intervals. Model estimates for the population at risk malaria are shown as green lines with dotted lines indicating 95% uncertainty intervals. Years indicate the mid-point of the calendar year. Note that not all symbols are present for each country.

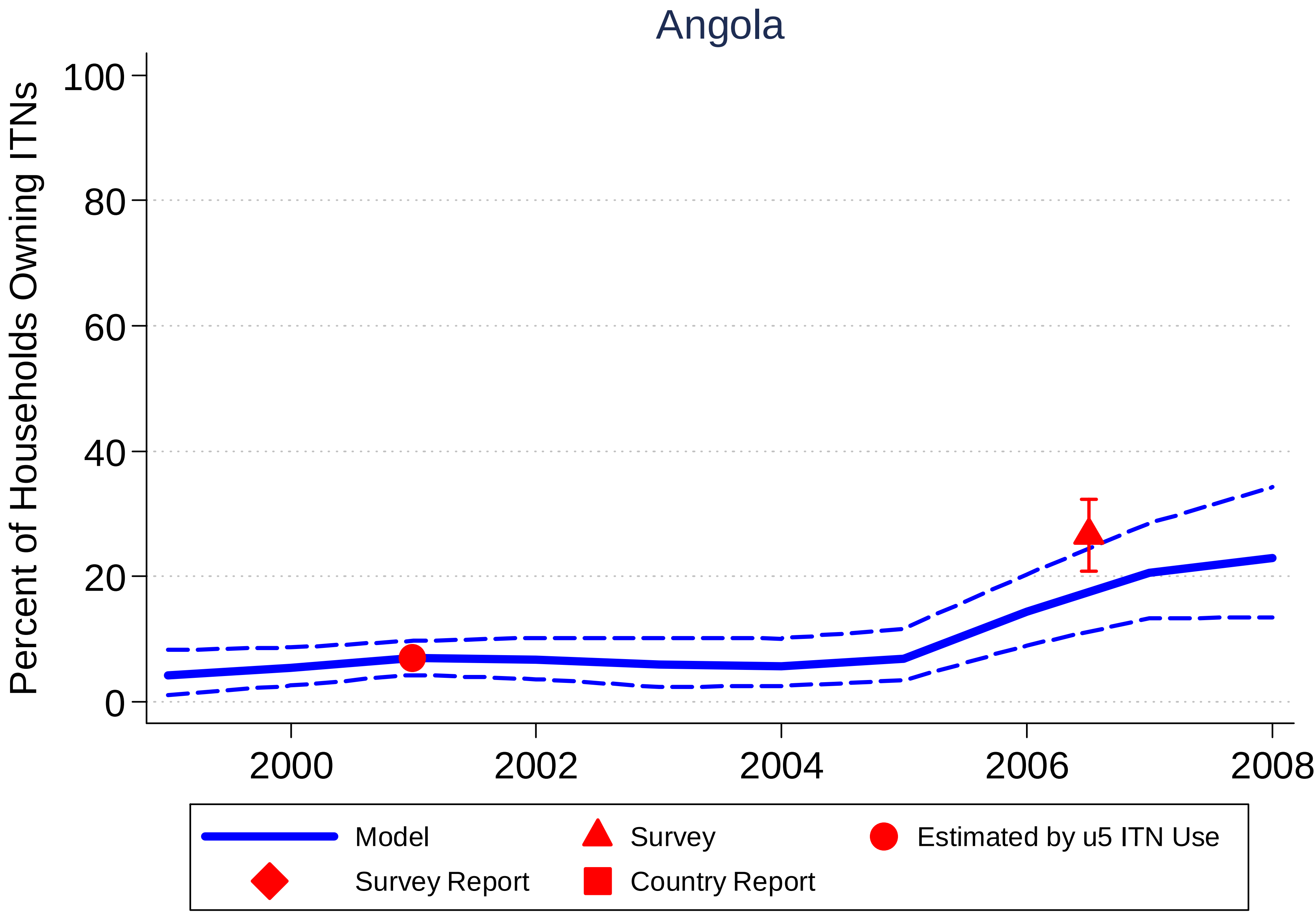

Angola
Percent of Households Owning ITNs
100
80
60
40
20
0
2000
2002
2004
2006
2008
Model
Survey
Estimated by u5 ITN Use
Survey Report
Country Report

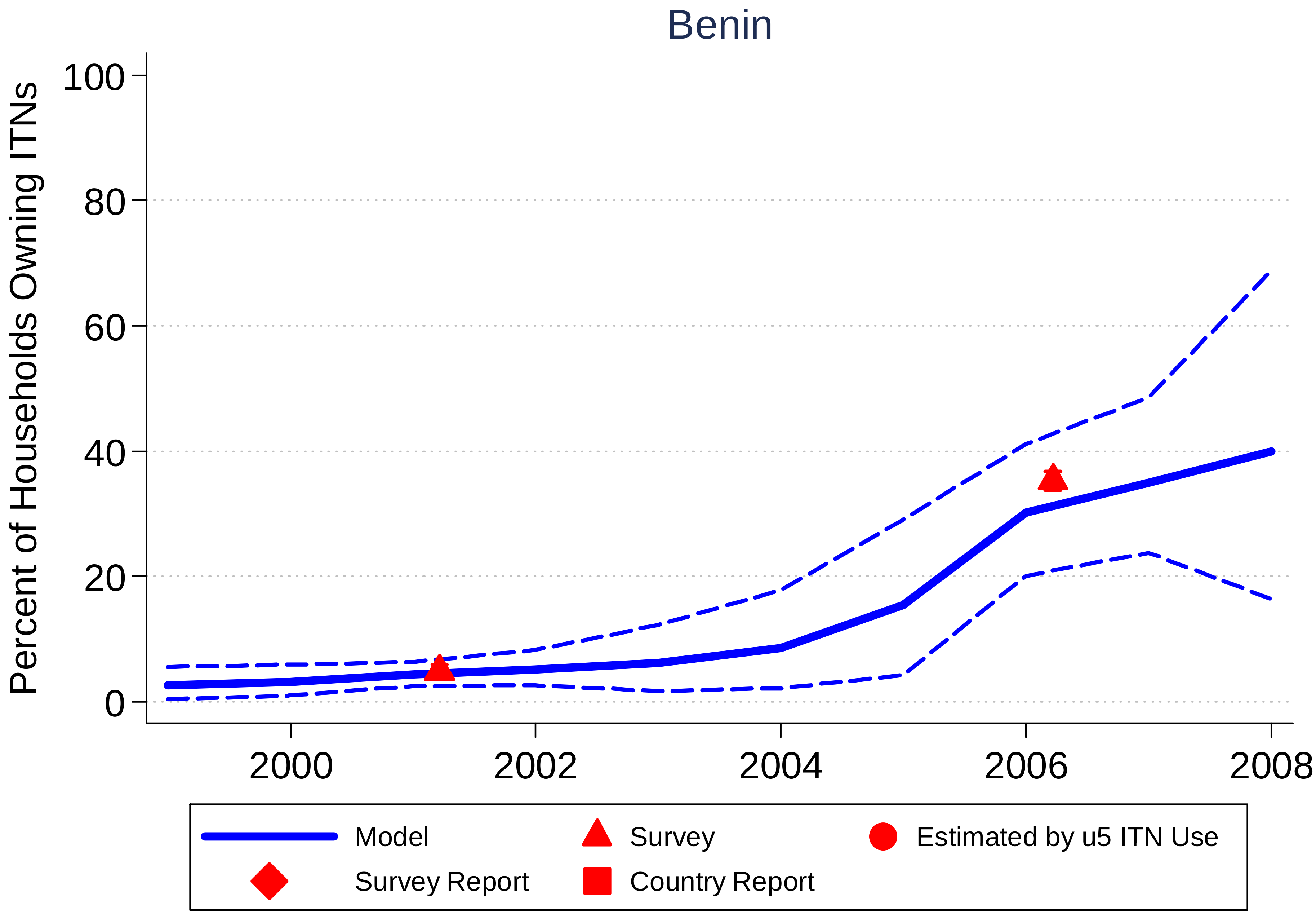
Benin
Percent of Households Owning ITNs
0
20
40
60
80
100
2000
2002
2004
2006
2008
Model
Survey
Estimated by u5 ITN Use
Survey Report
Country Report

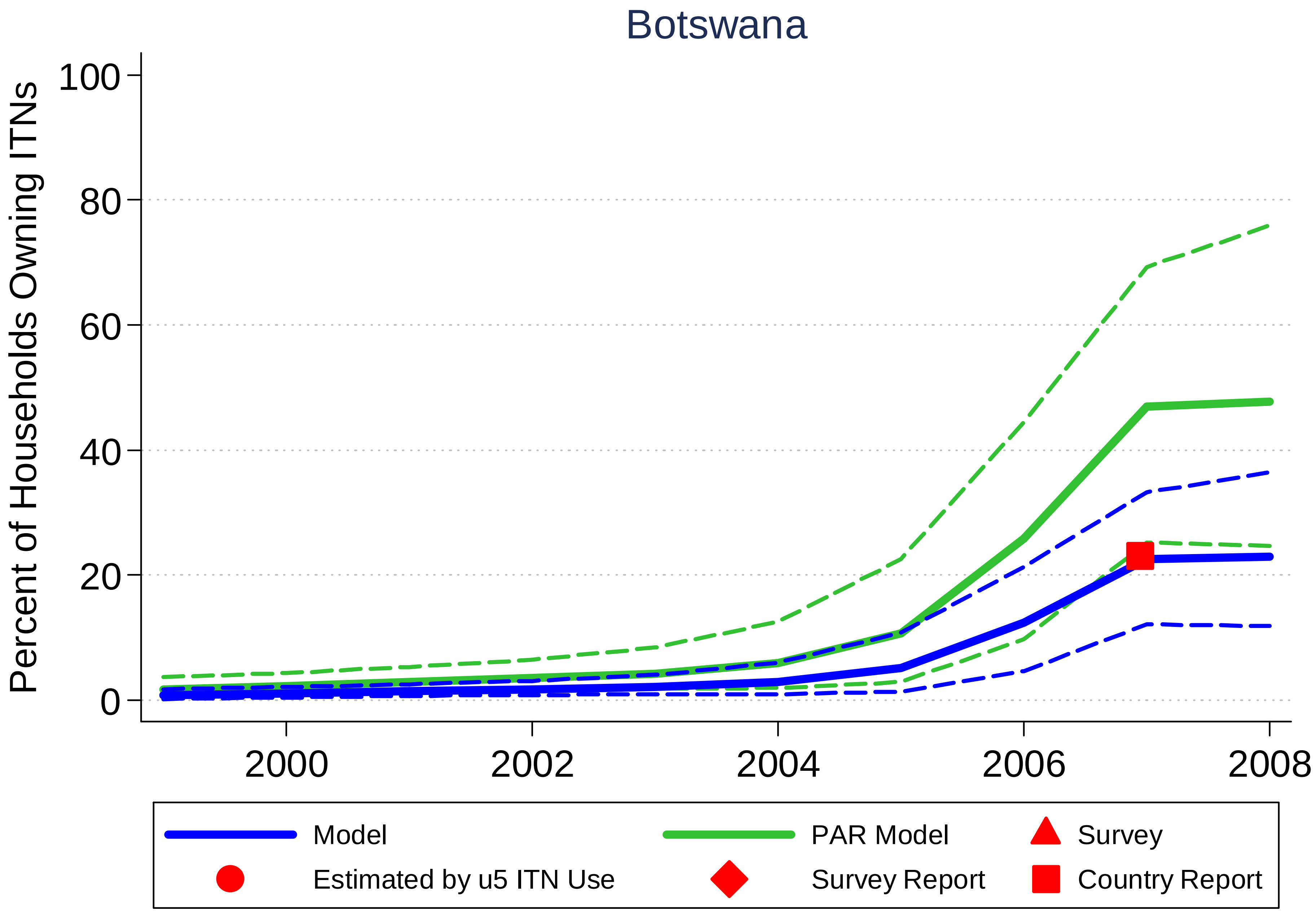
Botswana
Percent of Households Owning ITNs
100
80
60
40
20
0
2000
2002
2004
2006
2008
Model
PAR Model
Survey
Estimated by u5 ITN Use
Survey Report
Country Report

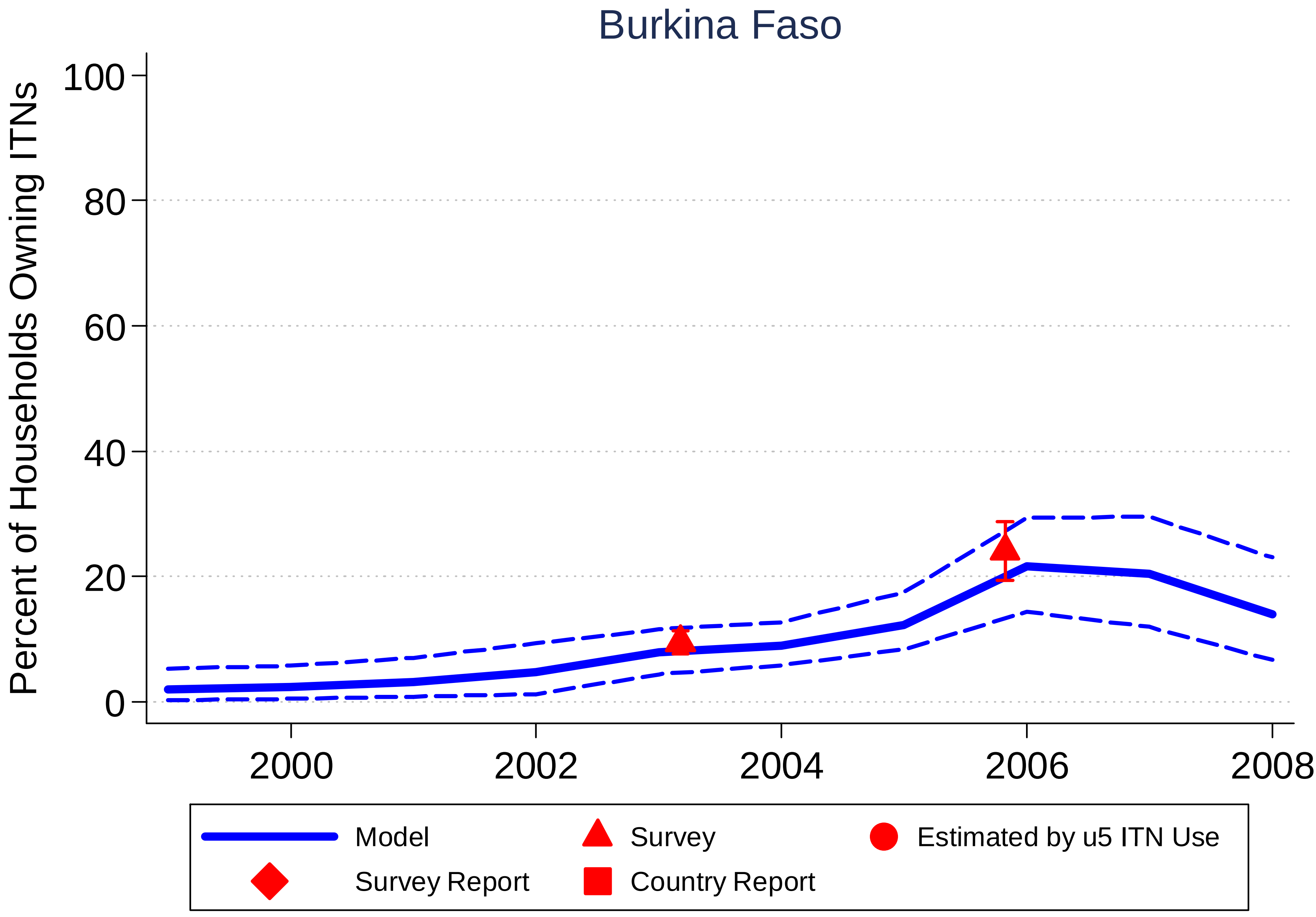

Burkina Faso
Percent of Households Owning ITNs
100
80
60
40
20
0
2000
2002
2004
2006
2008
Model
Survey
Estimated by u5 ITN Use
Survey Report
Country Report

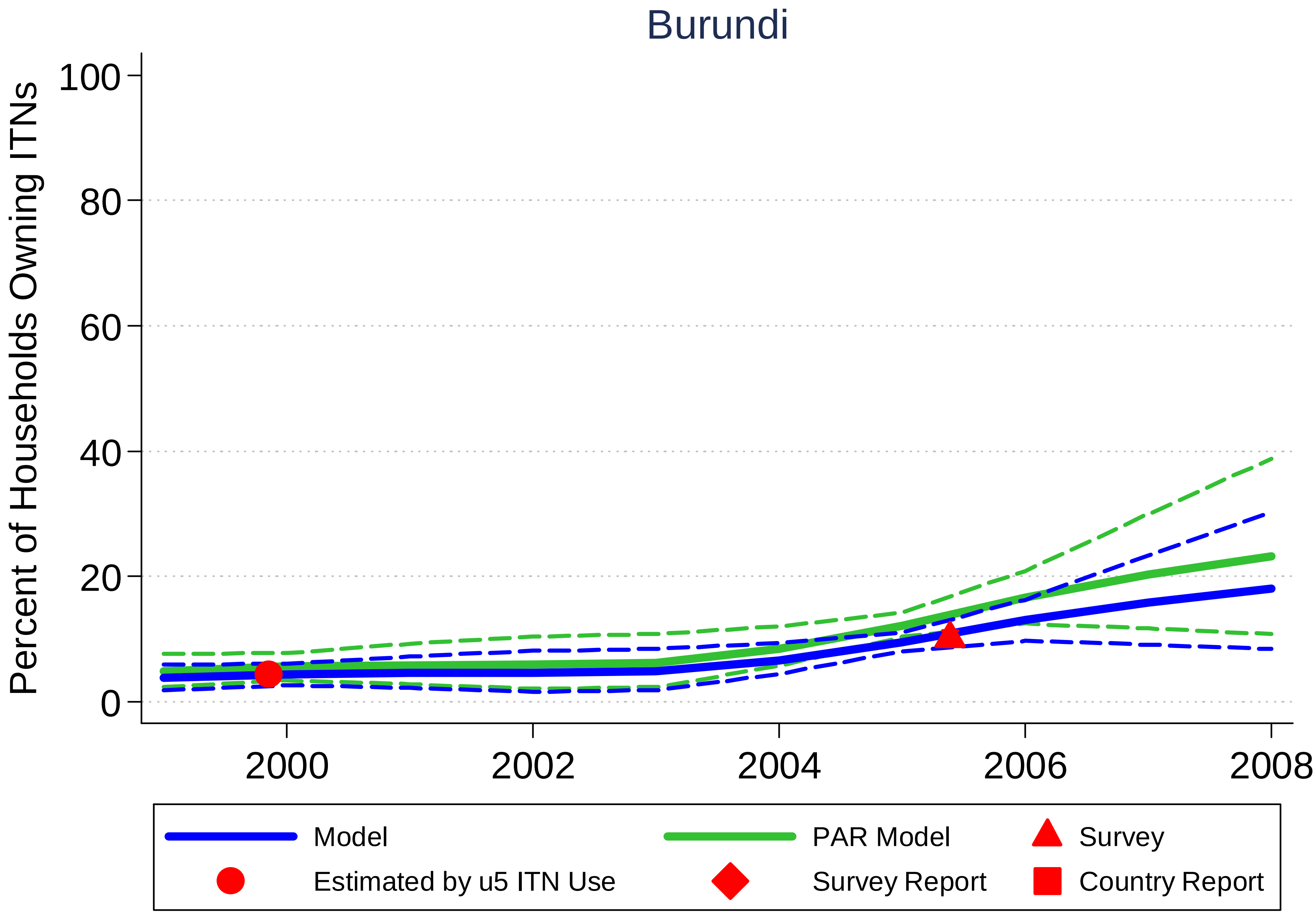
Burundi
Percent of Households Owning ITNs
0
20
40
60
80
100
2000
2002
2004
2006
2008
Model
PAR Model
Survey
Estimated by u5 ITN Use
Survey Report
Country Report

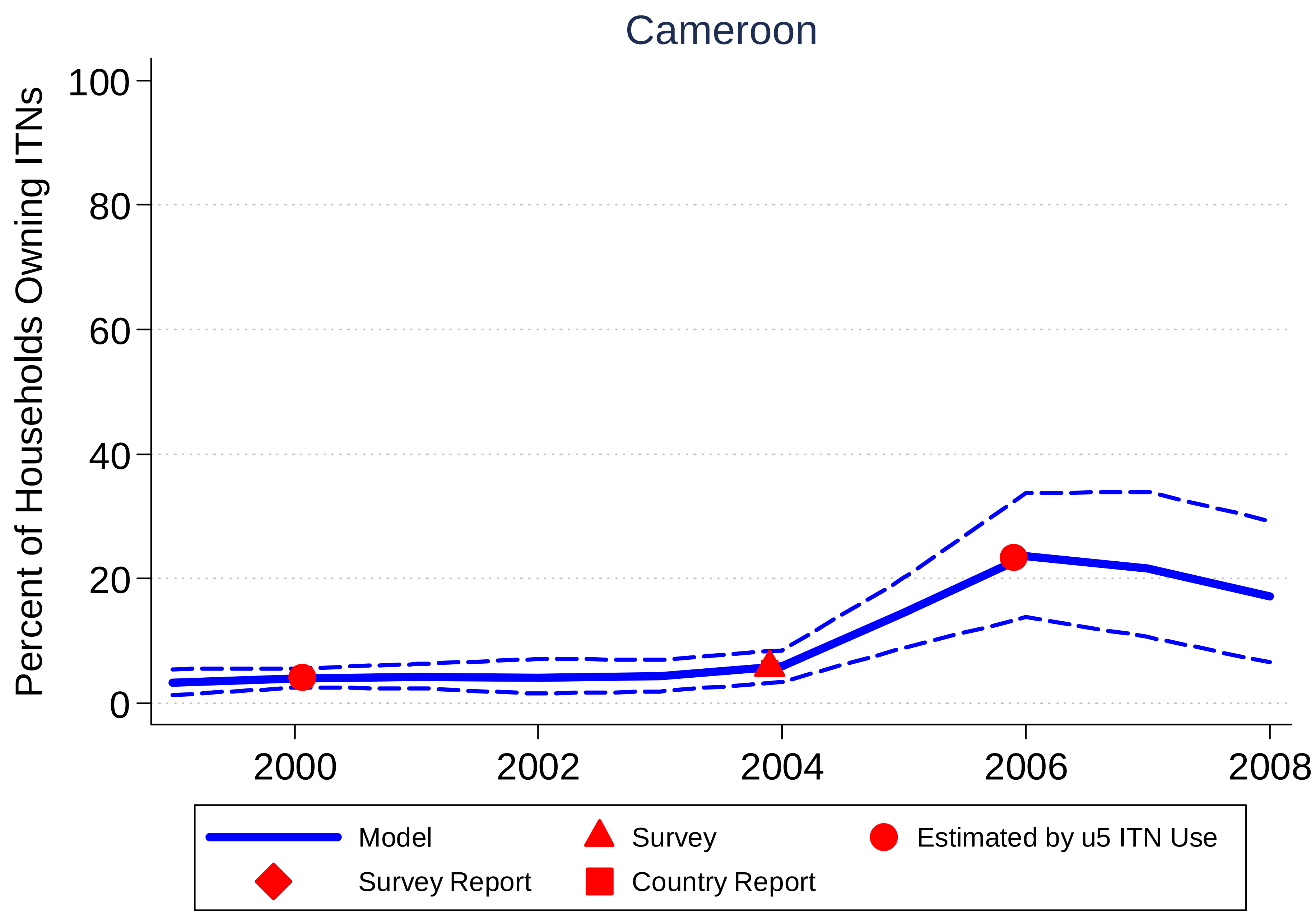
Cameroon
Percent of Households Owning ITNs
100
80
60
40
20
0
2000
2002
2004
2006
2008
Model
Survey
Estimated by u5 ITN Use
Survey Report
Country Report

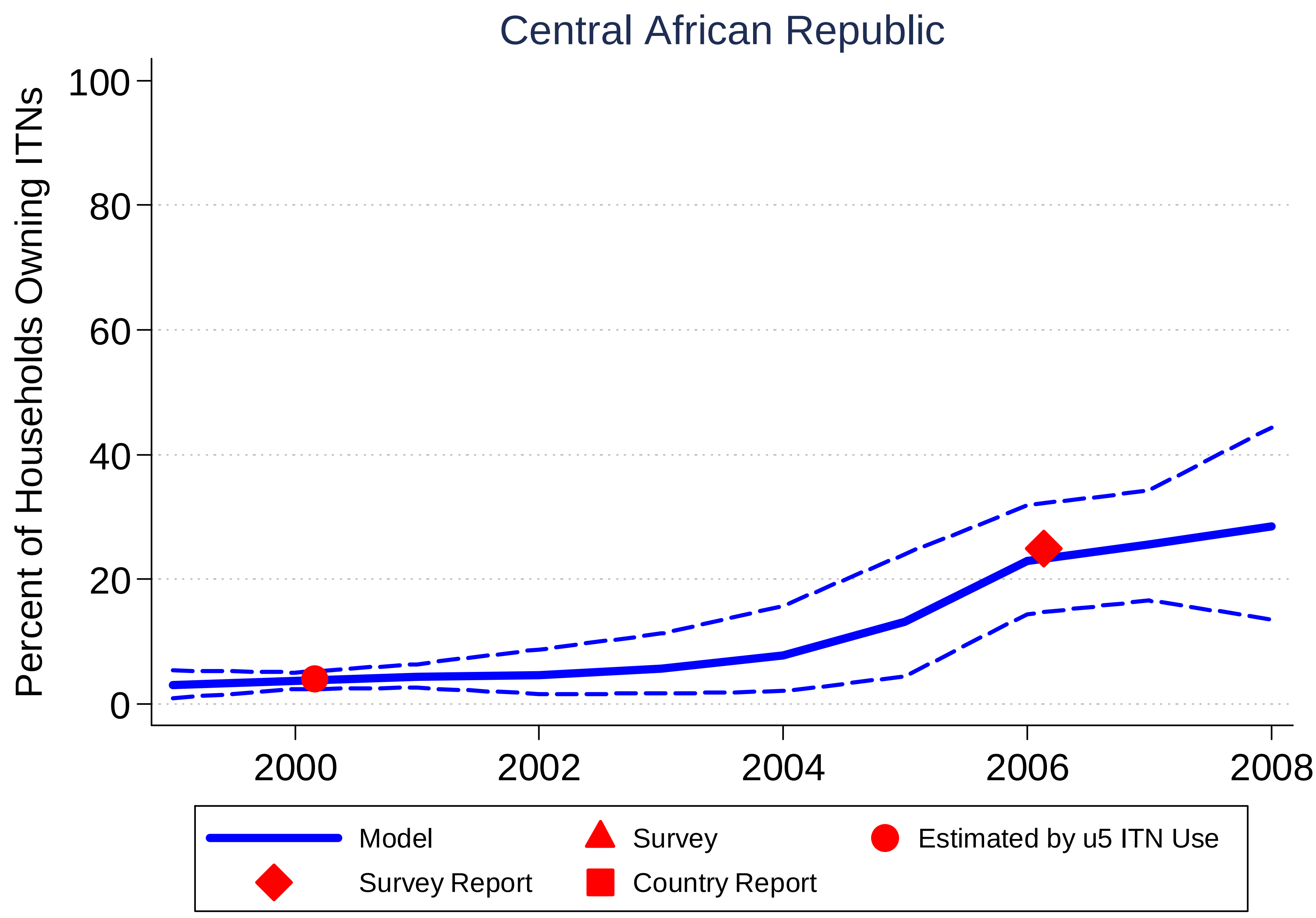
Central African Republic
Percent of Households Owning ITNs
100
80
60
40
20
0
2000
2002
2004
2006
2008
Model
Survey
Estimated by u5 ITN Use
Survey Report
Country Report

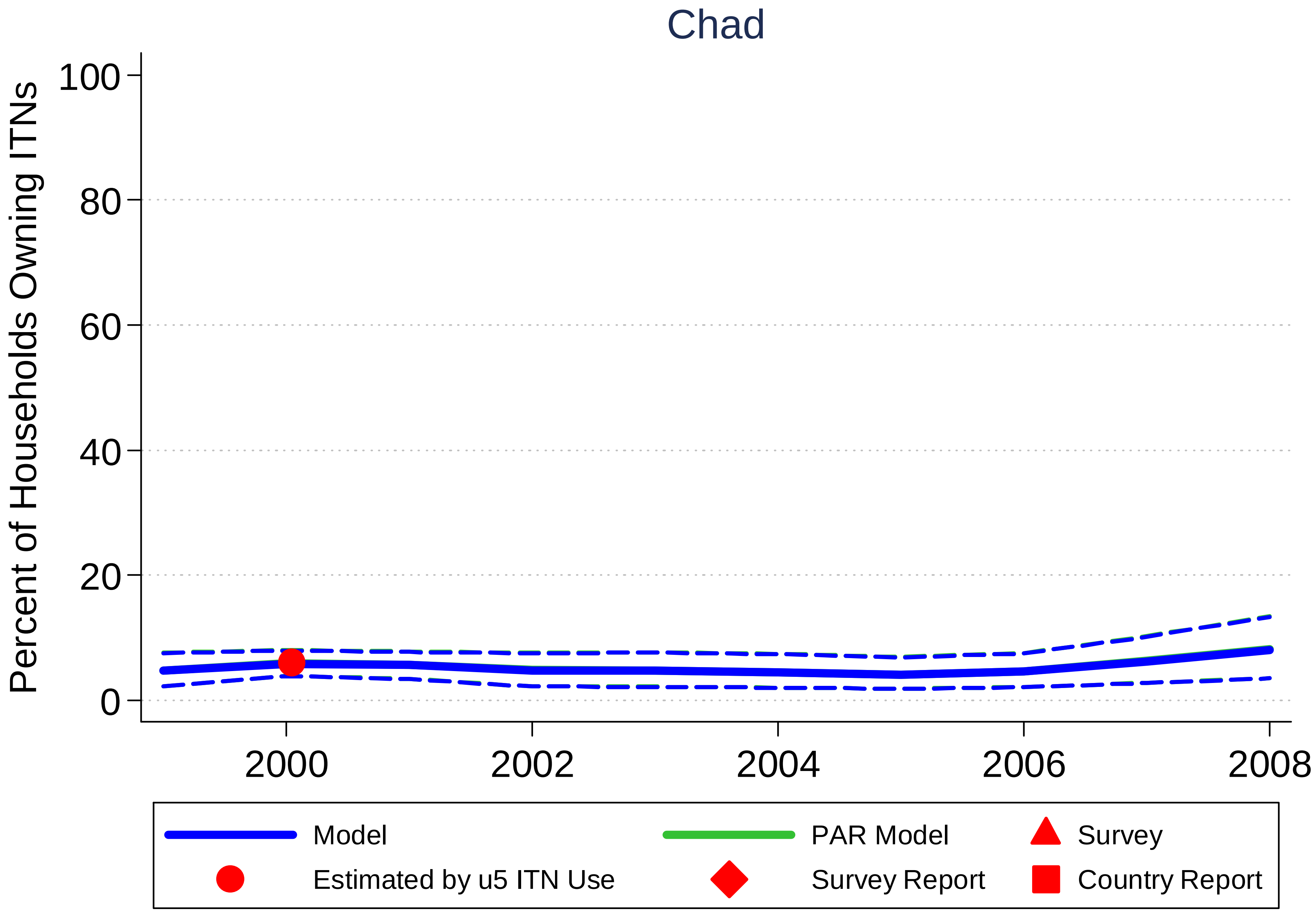

Chad
Percent of Households Owning ITNs
100
80
60
40
20
0
2000
2002
2004
2006
2008
Model
PAR Model
Survey
Estimated by u5 ITN Use
Survey Report
Country Report

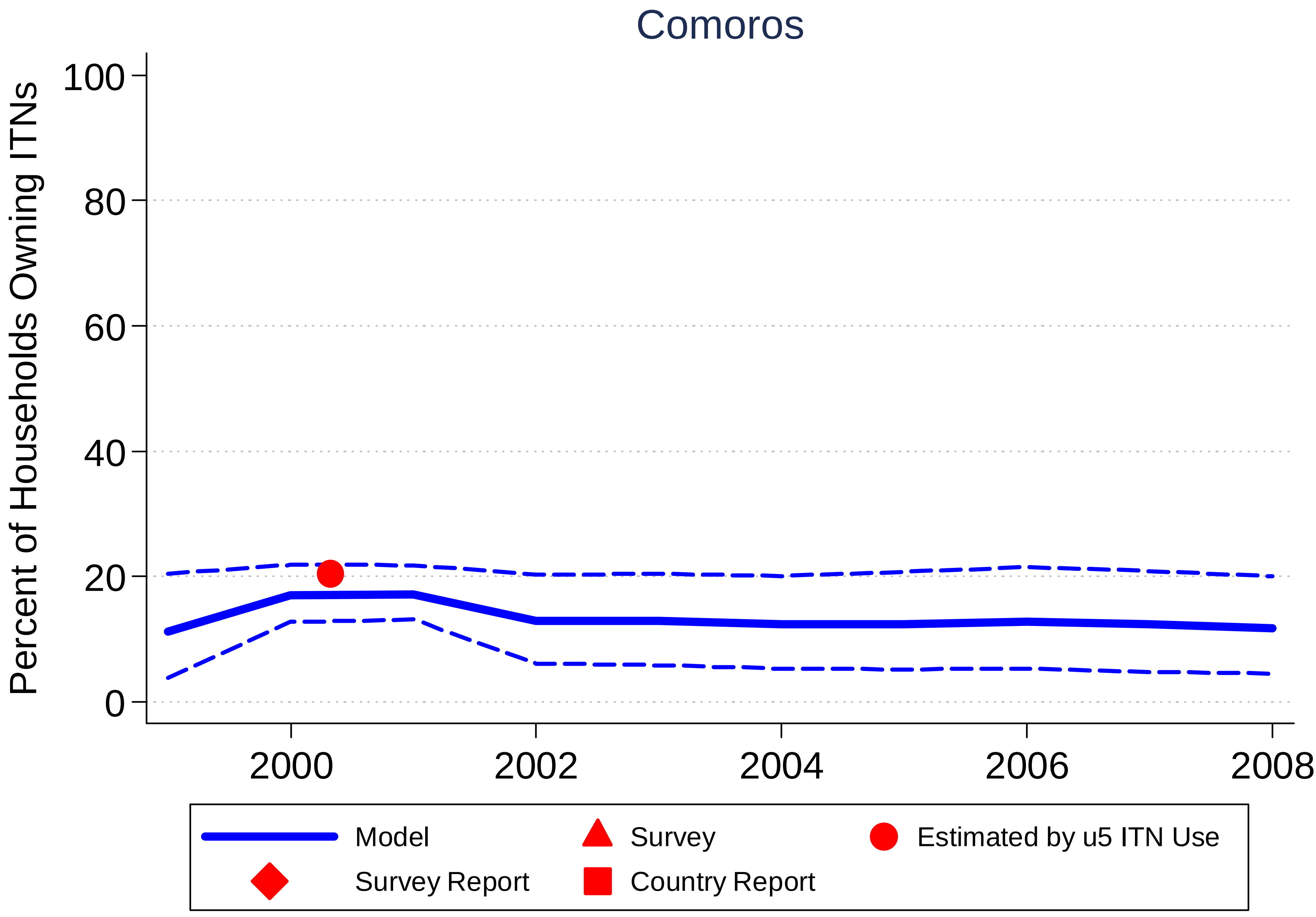

Comoros
Percent of Households Owning ITNs
100
80
60
40
20
0
2000
2002
2004
2006
2008
Model
Survey
Estimated by u5 ITN Use
Survey Report
Country Report

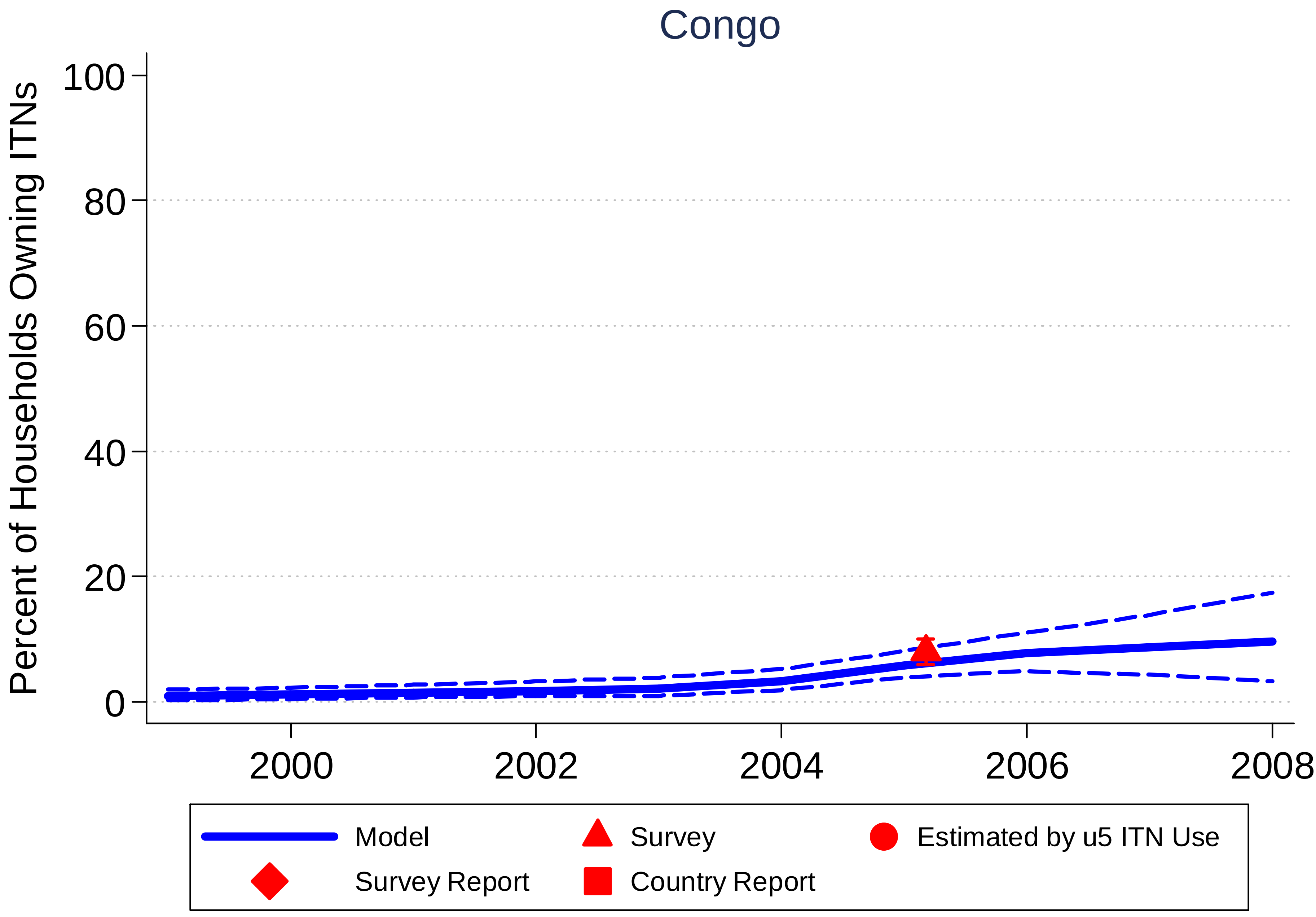

Congo
Percent of Households Owning ITNs
0
20
40
60
80
100
2000
2002
2004
2006
2008
Model
Survey
Estimated by u5 ITN Use
Survey Report
Country Report

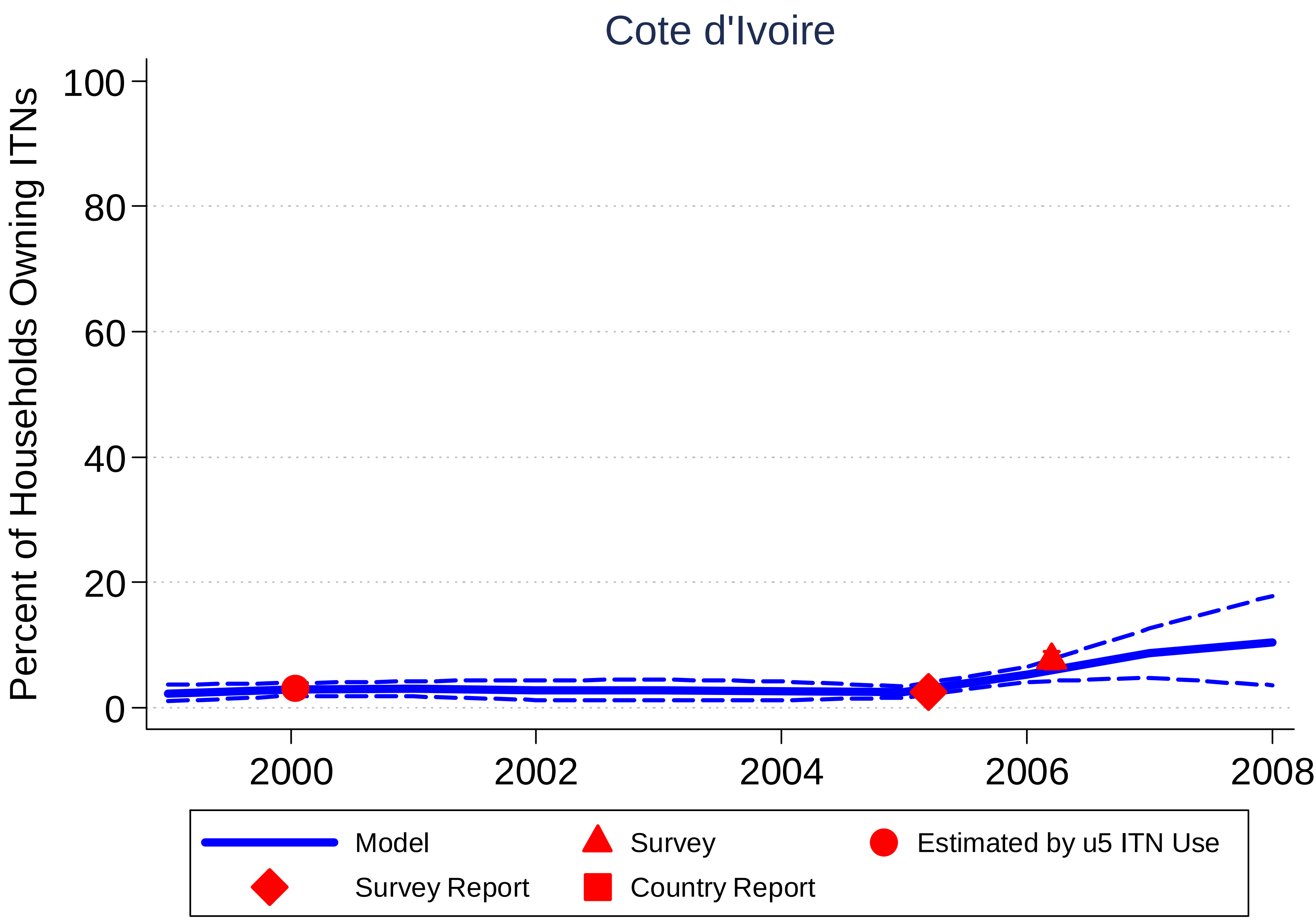

Cote d'Ivoire
Percent of Households Owning ITNs
0
20
40
60
80
100
2000
2002
2004
2006
2008
Model
Survey
Estimated by u5 ITN Use
Survey Report
Country Report

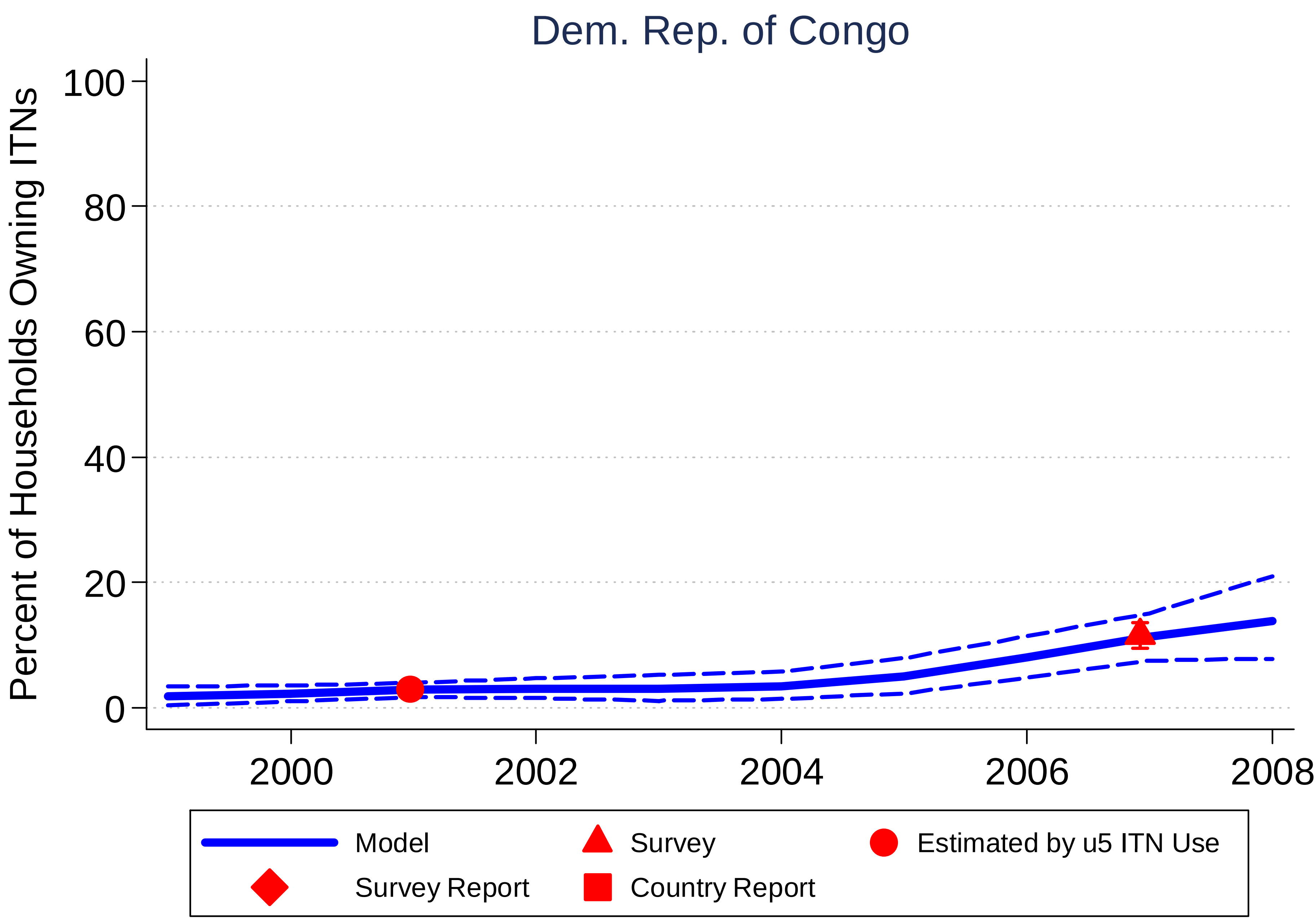

Dem. Rep. of Congo
Percent of Households Owning ITNs
100
80
60
40
20
0
2000
2002
2004
2006
2008
Model
Survey
Estimated by u5 ITN Use
Survey Report
Country Report

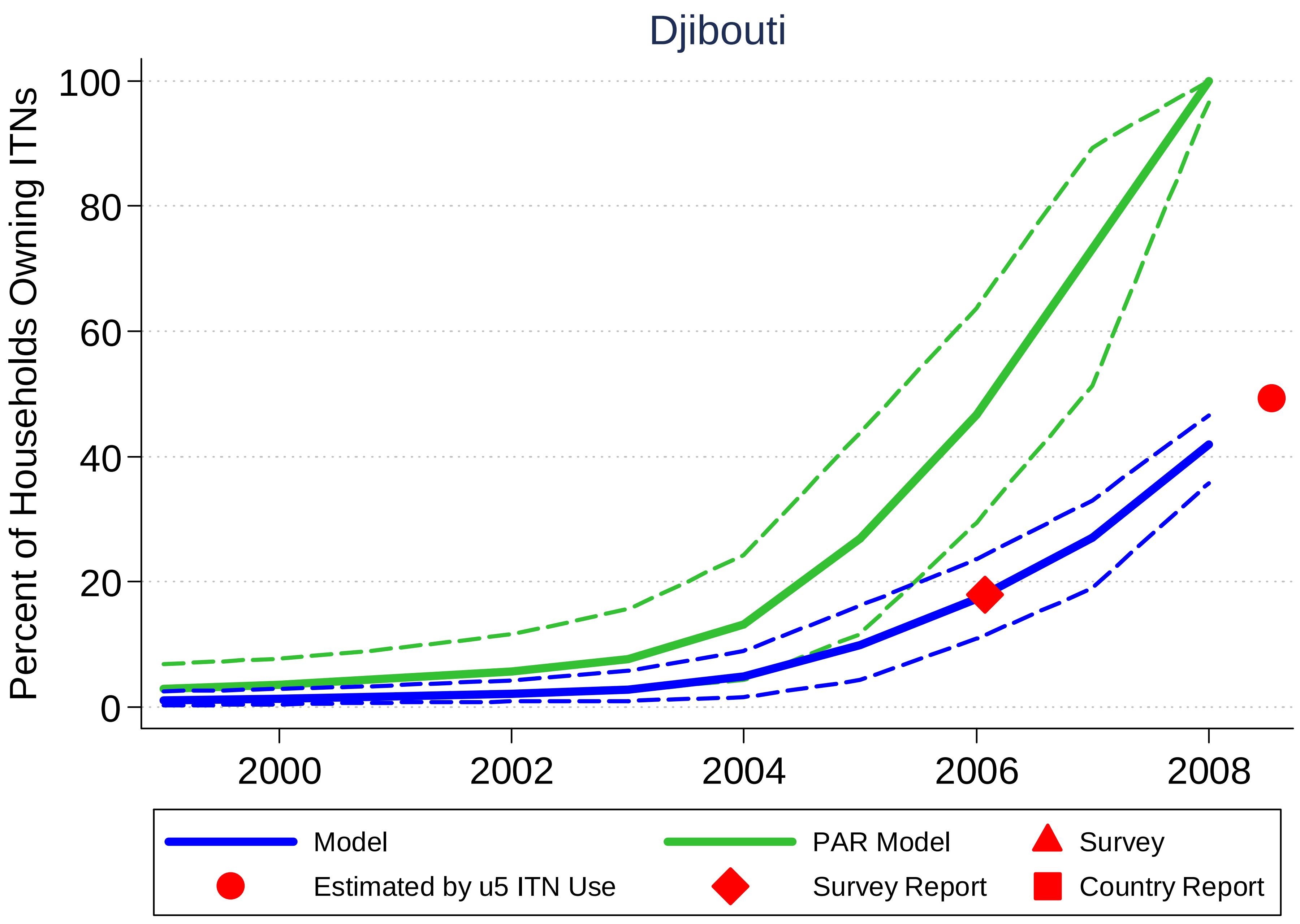

Djibouti
Percent of Households Owning ITNs
100
80
60
40
20
0
2000
2002
2004
2006
2008
Model
PAR Model
Survey
Estimated by u5 ITN Use
Survey Report
Country Report

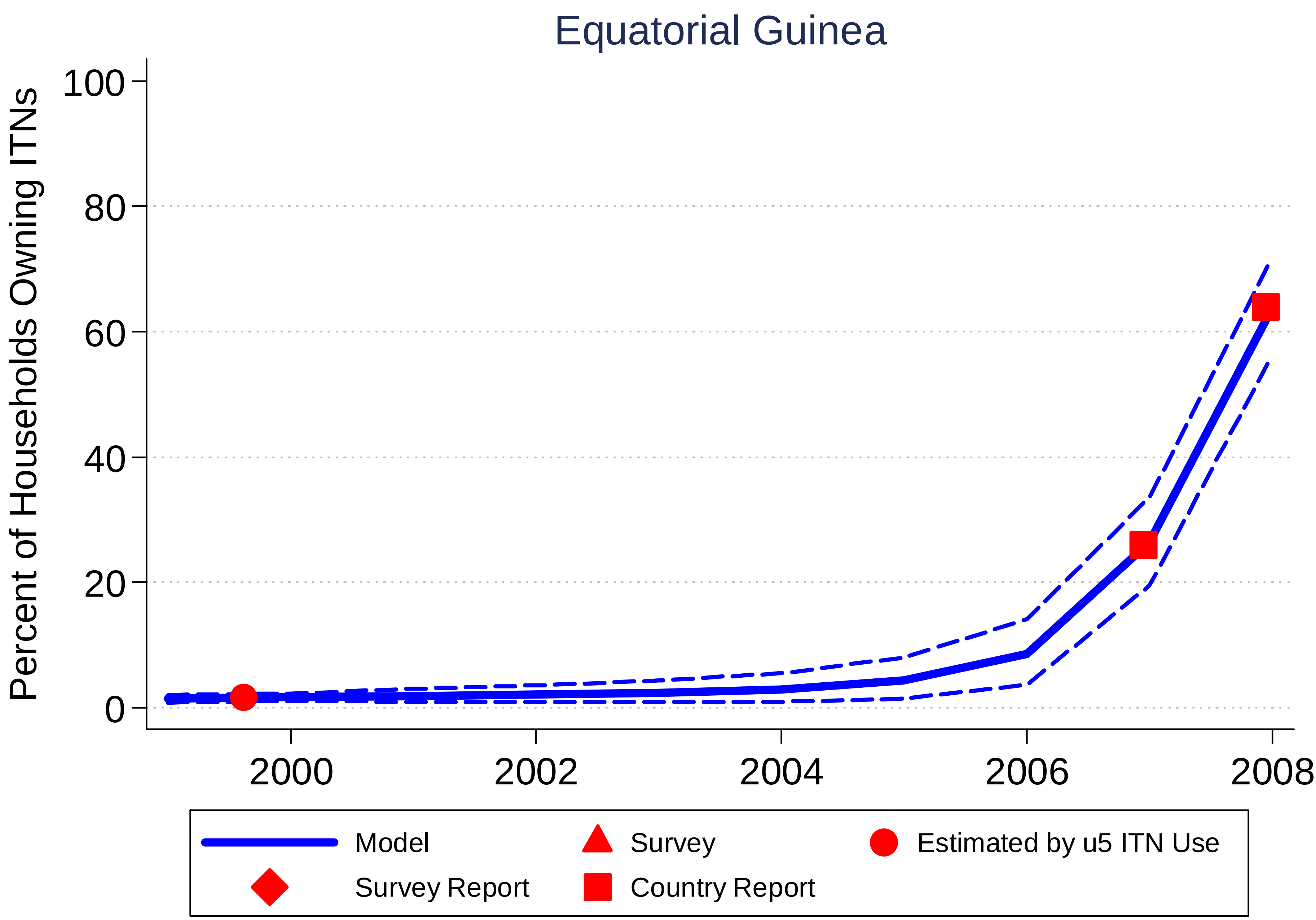

Equatorial Guinea
Percent of Households Owning ITNs
0
20
40
60
80
100
2000
2002
2004
2006
2008
Model
Survey
Estimated by u5 ITN Use
Survey Report
Country Report

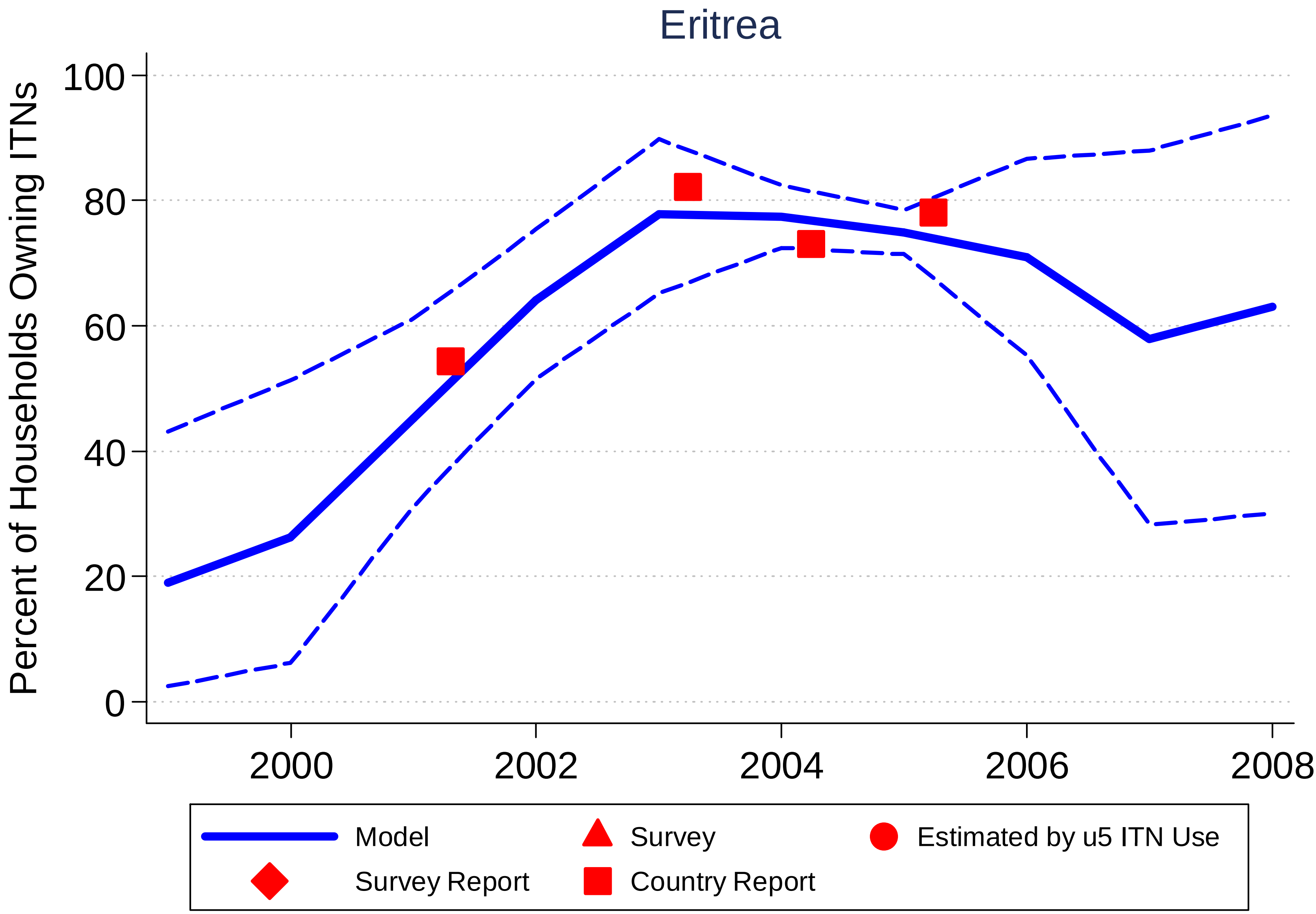

Eritrea
Percent of Households Owning ITNs
100
80
60
40
20
0
2000
2002
2004
2006
2008
Model
Survey
Estimated by u5 ITN Use
Survey Report
Country Report

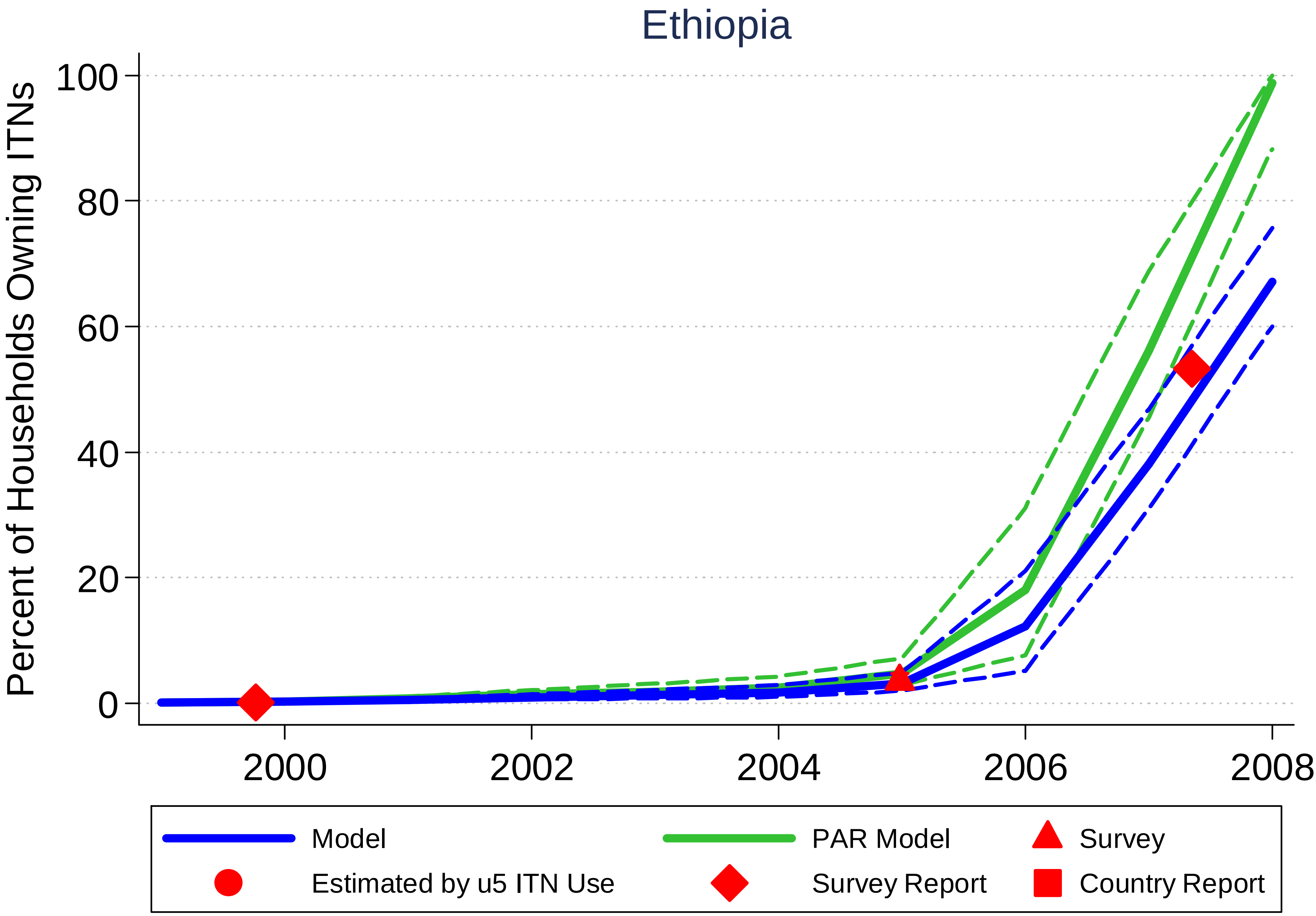

Ethiopia
Percent of Households Owning ITNs
100
80
60
40
20
0
2000
2002
2004
2006
2008
Model
PAR Model
Survey
Estimated by u5 ITN Use
Survey Report
Country Report

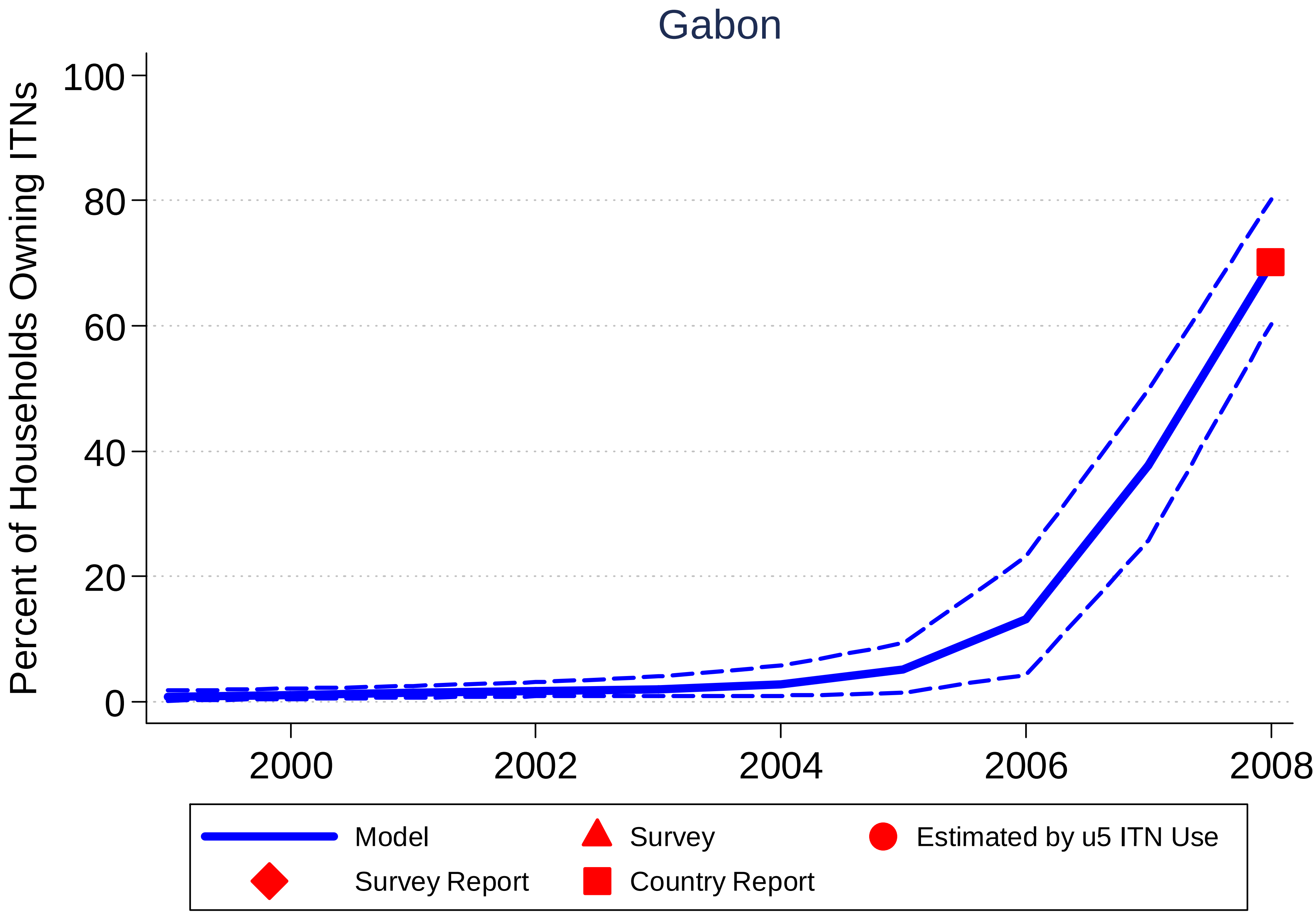
Gabon
Percent of Households Owning ITNs
100
80
60
40
20
0
2000
2002
2004
2006
2008
Model
Survey
Estimated by u5 ITN Use
Survey Report
Country Report

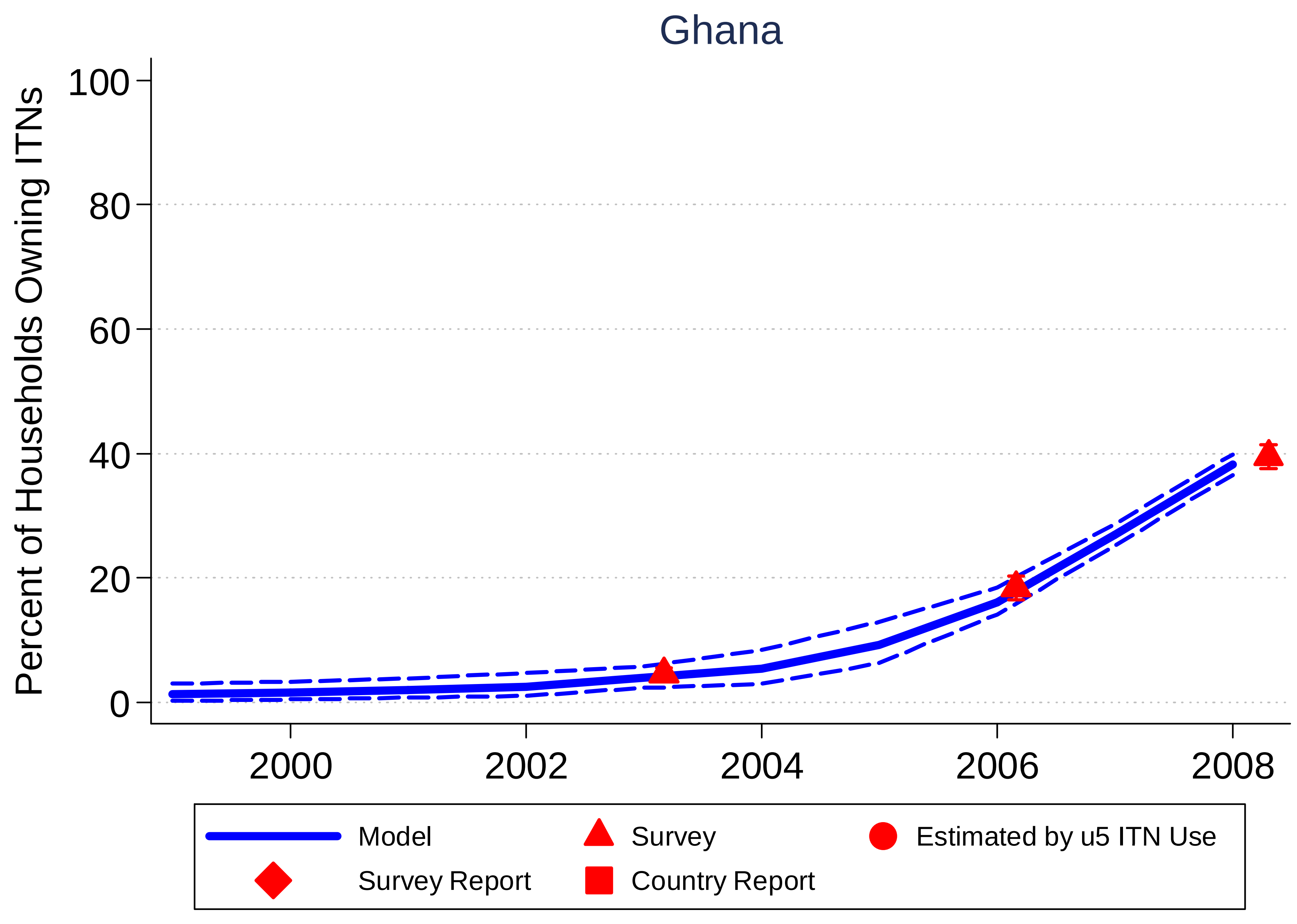

Ghana
Percent of Households Owning ITNs
0
20
40
60
80
100
2000
2002
2004
2006
2008
Model
Survey
Estimated by u5 ITN Use
Survey Report
Country Report

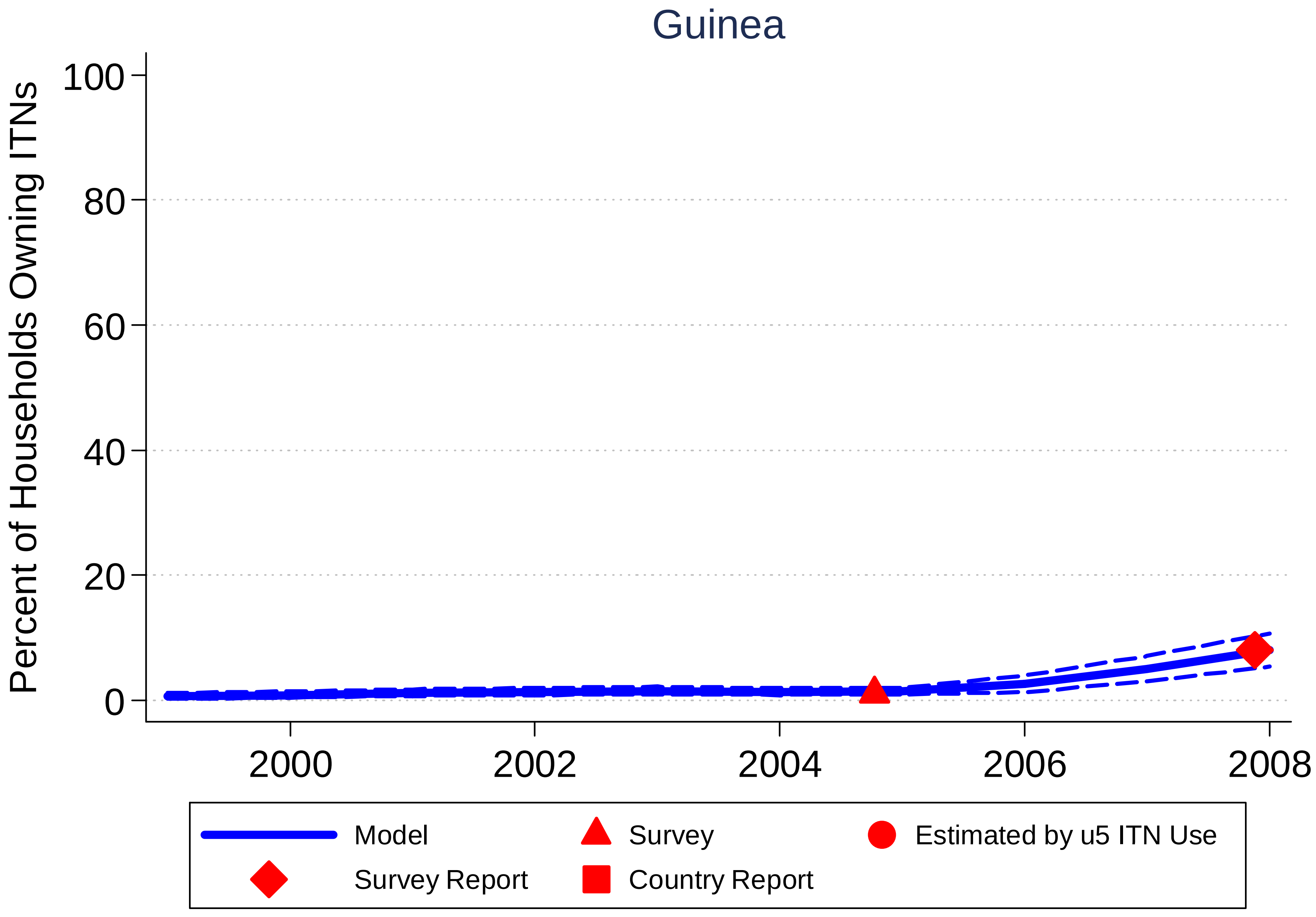

Guinea
Percent of Households Owning ITNs
100
80
60
40
20
0
2000
2002
2004
2006
2008
Model
Survey
Estimated by u5 ITN Use
Survey Report
Country Report

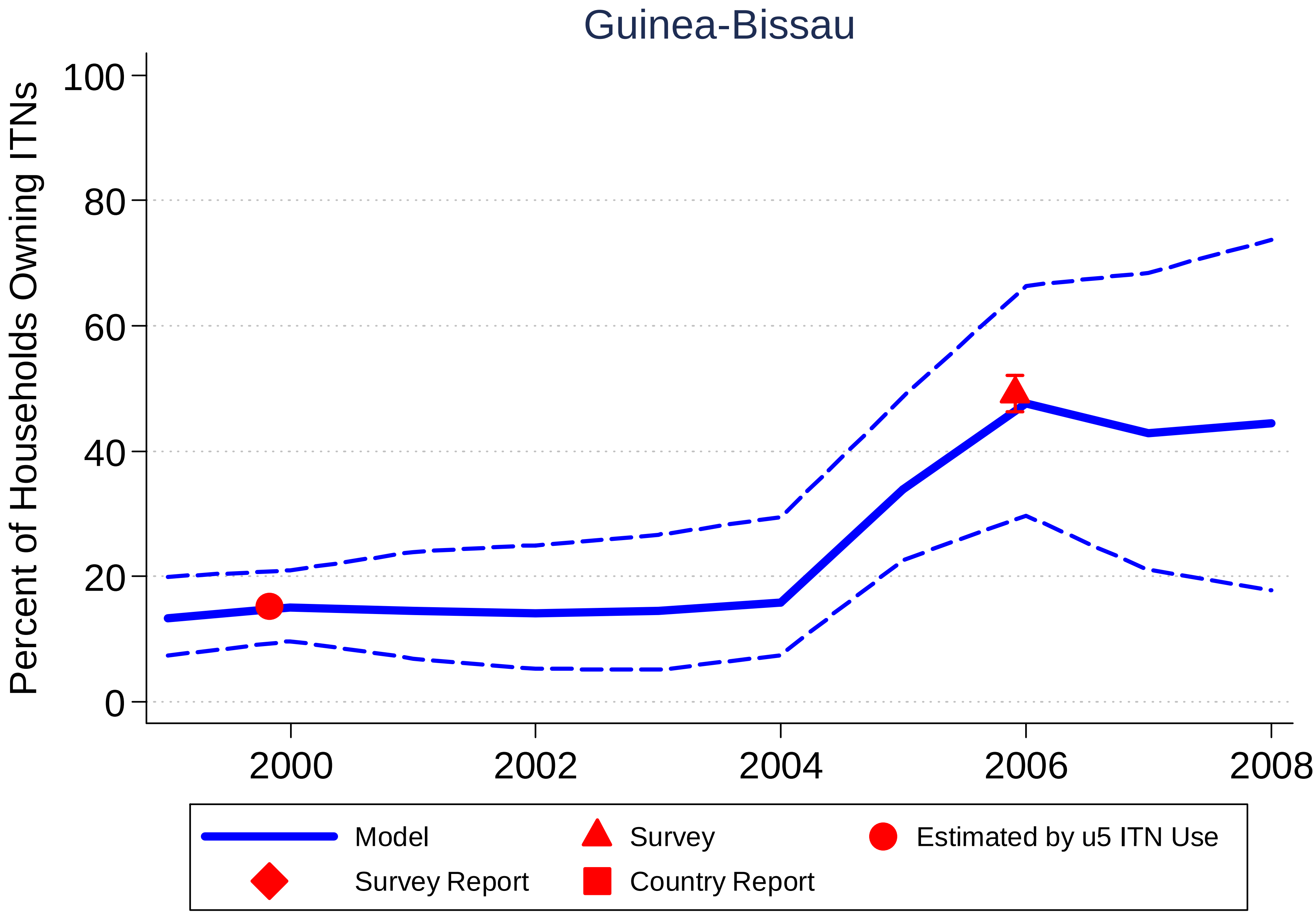
Guinea-Bissau
Percent of Households Owning ITNs
0
20
40
60
80
100
2000
2002
2004
2006
2008
Model
Survey
Estimated by u5 ITN Use
Survey Report
Country Report

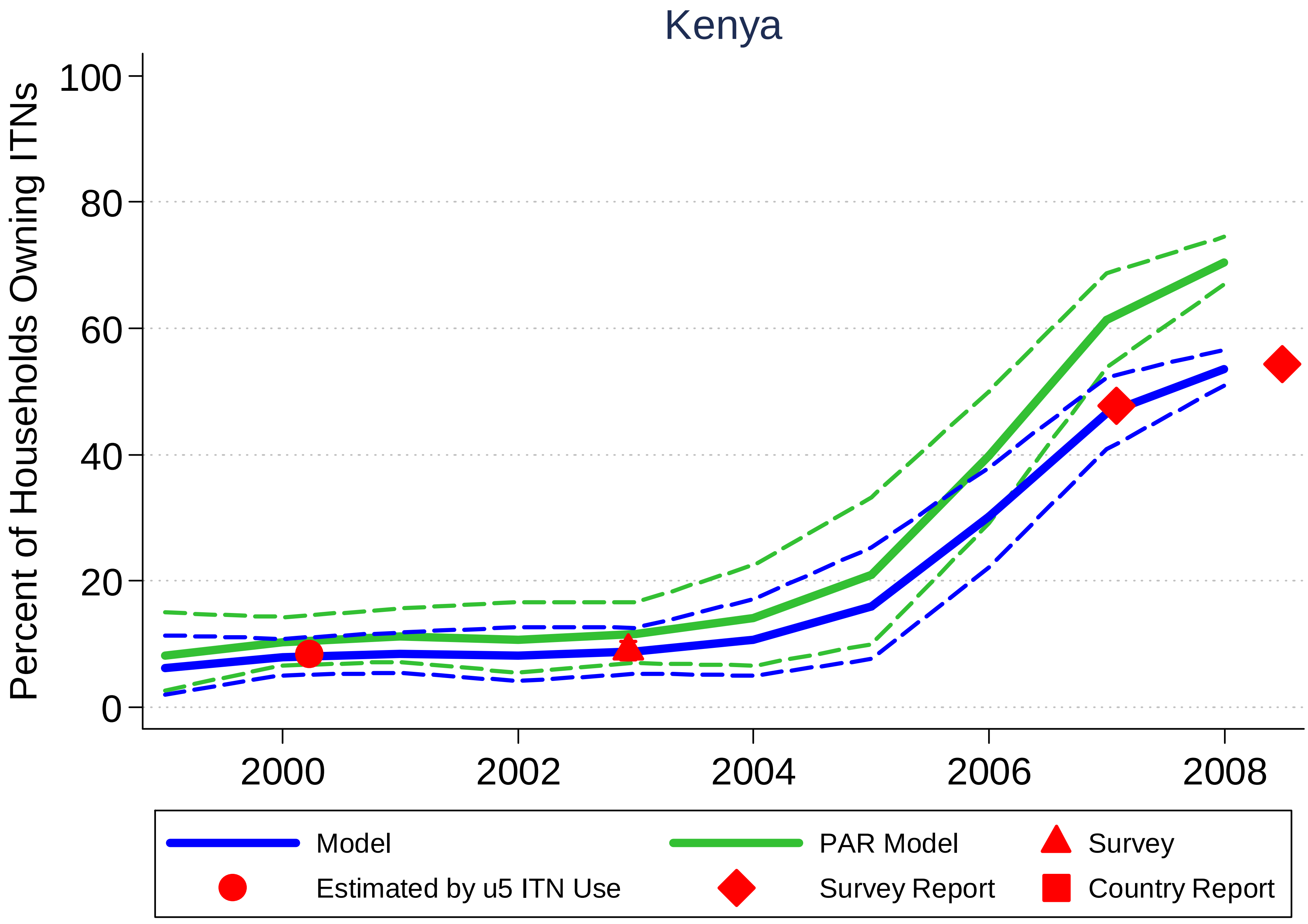
Kenya
Percent of Households Owning ITNs
0
20
40
60
80
100
2000
2002
2004
2006
2008
Model
PAR Model
Survey
Estimated by u5 ITN Use
Survey Report
Country Report

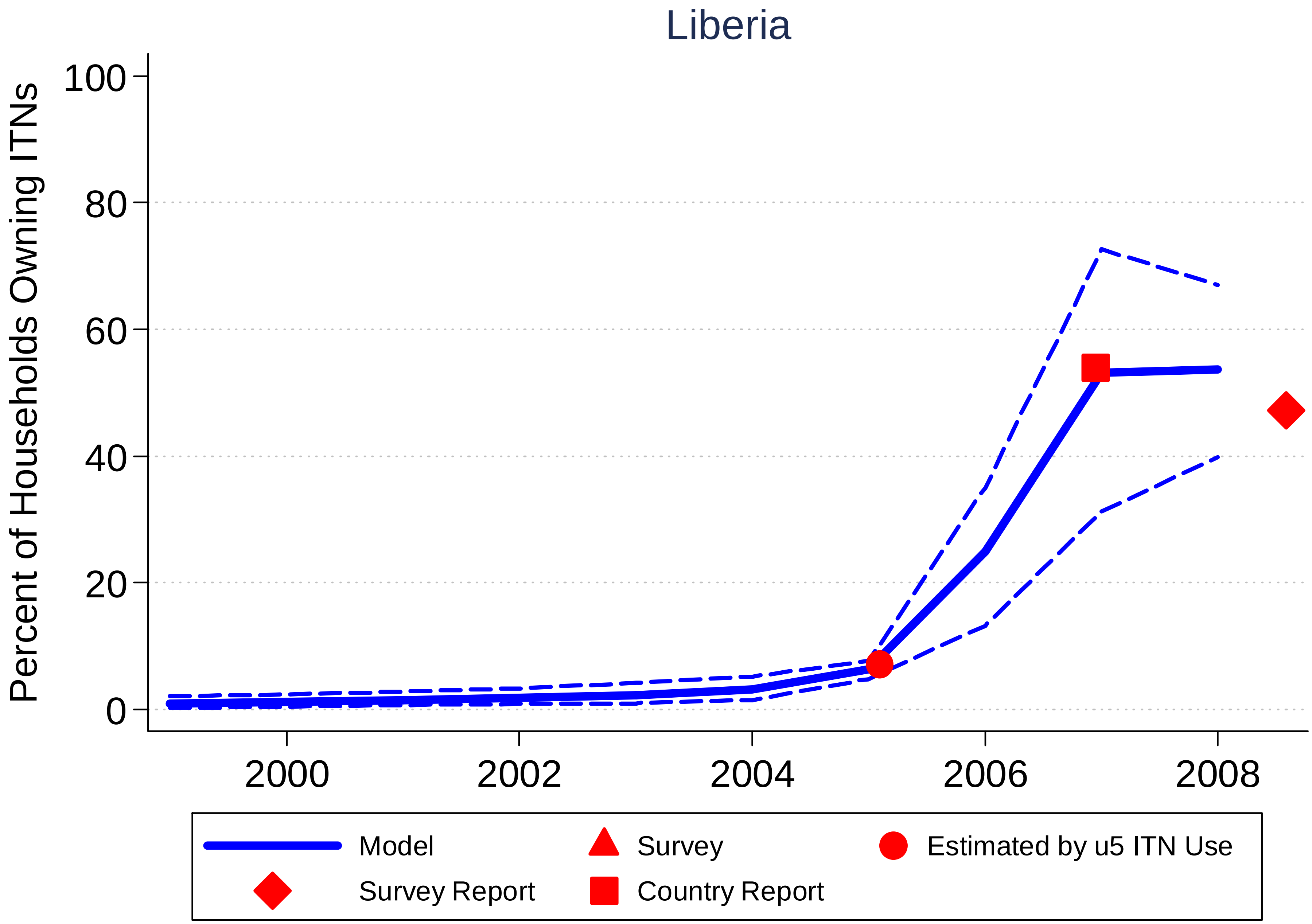
Liberia
Percent of Households Owning ITNs
100
80
60
40
20
0
2000
2002
2004
2006
2008
Model
Survey
Estimated by u5 ITN Use
Survey Report
Country Report

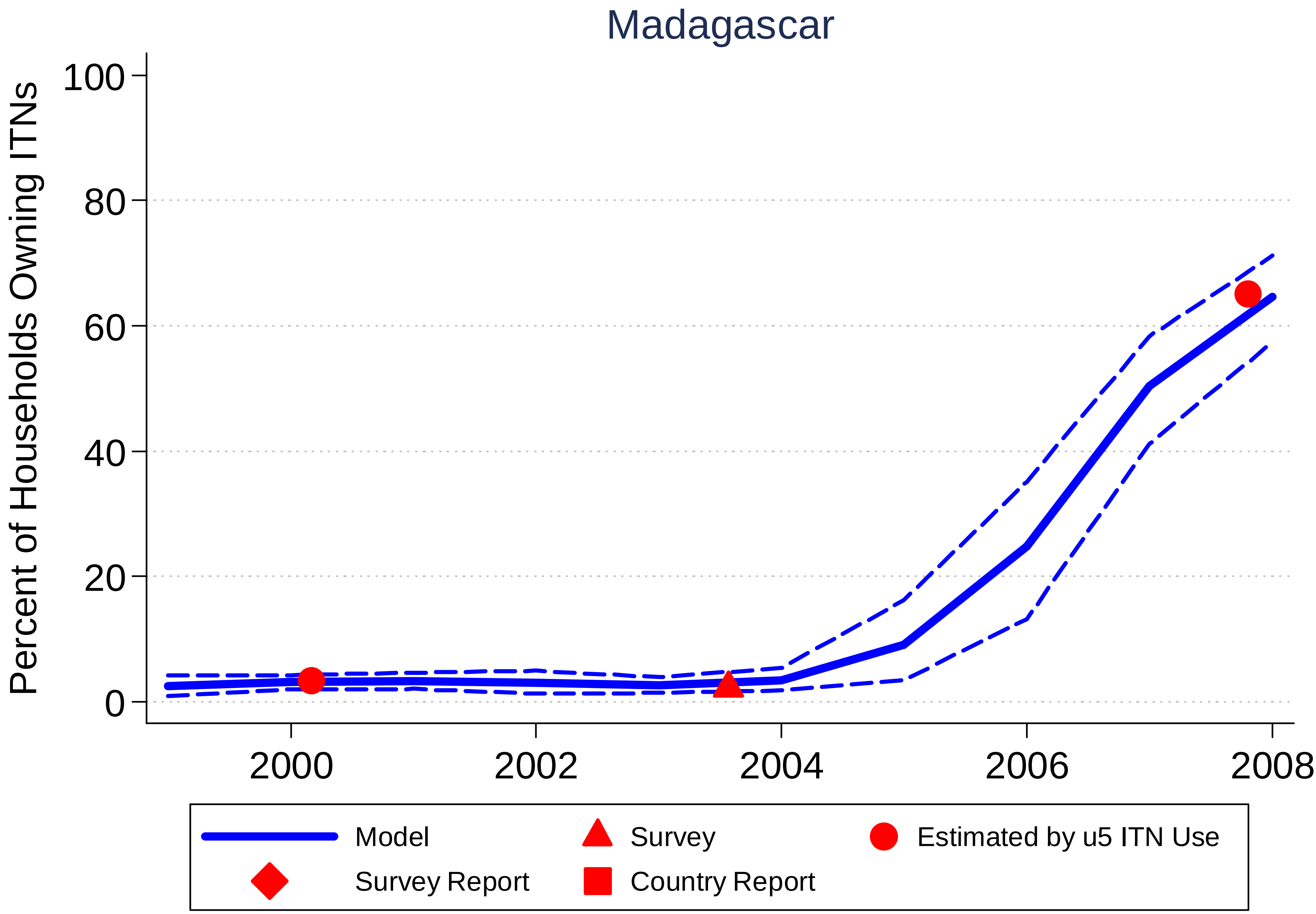

Madagascar
Percent of Households Owning ITNs
0
20
40
60
80
100
2000
2002
2004
2006
2008
Model
Survey
Estimated by u5 ITN Use
Survey Report
Country Report

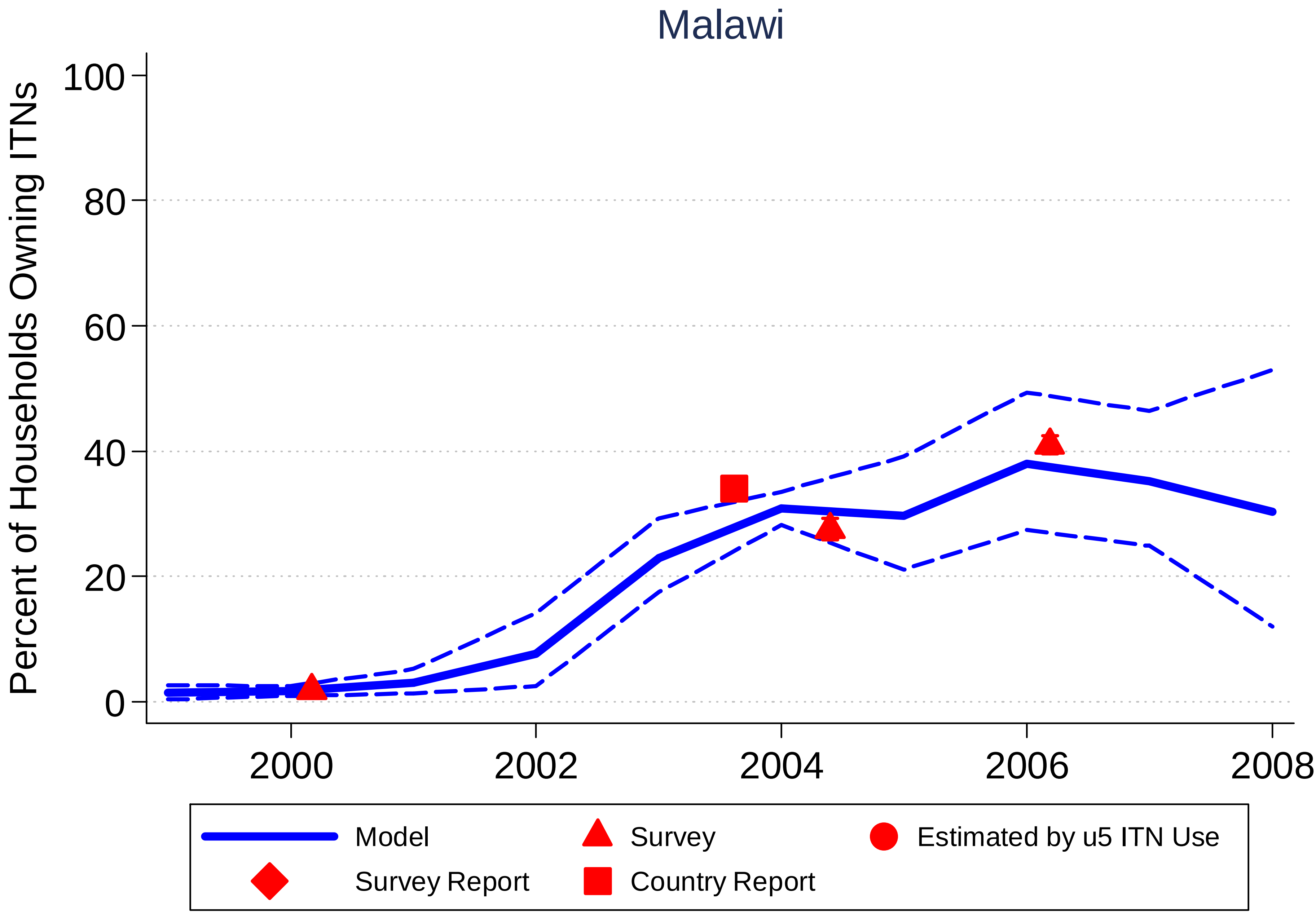
Malawi
Percent of Households Owning ITNs
0
20
40
60
80
100
2000
2002
2004
2006
2008
Model
Survey
Estimated by u5 ITN Use
Survey Report
Country Report

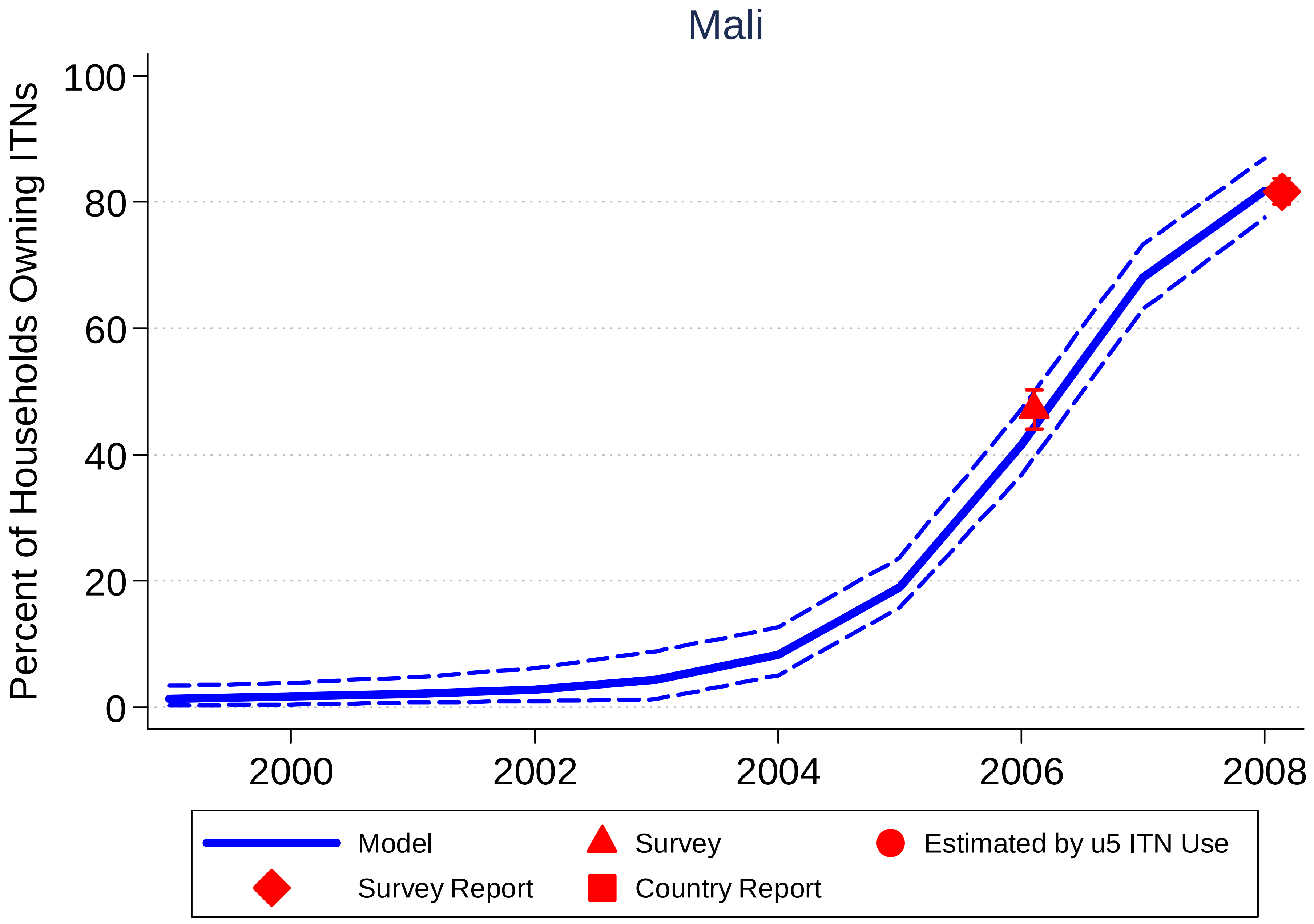

Mali
Percent of Households Owning ITNs
0
20
40
60
80
100
2000
2002
2004
2006
2008
Model
Survey
Estimated by u5 ITN Use
Survey Report
Country Report

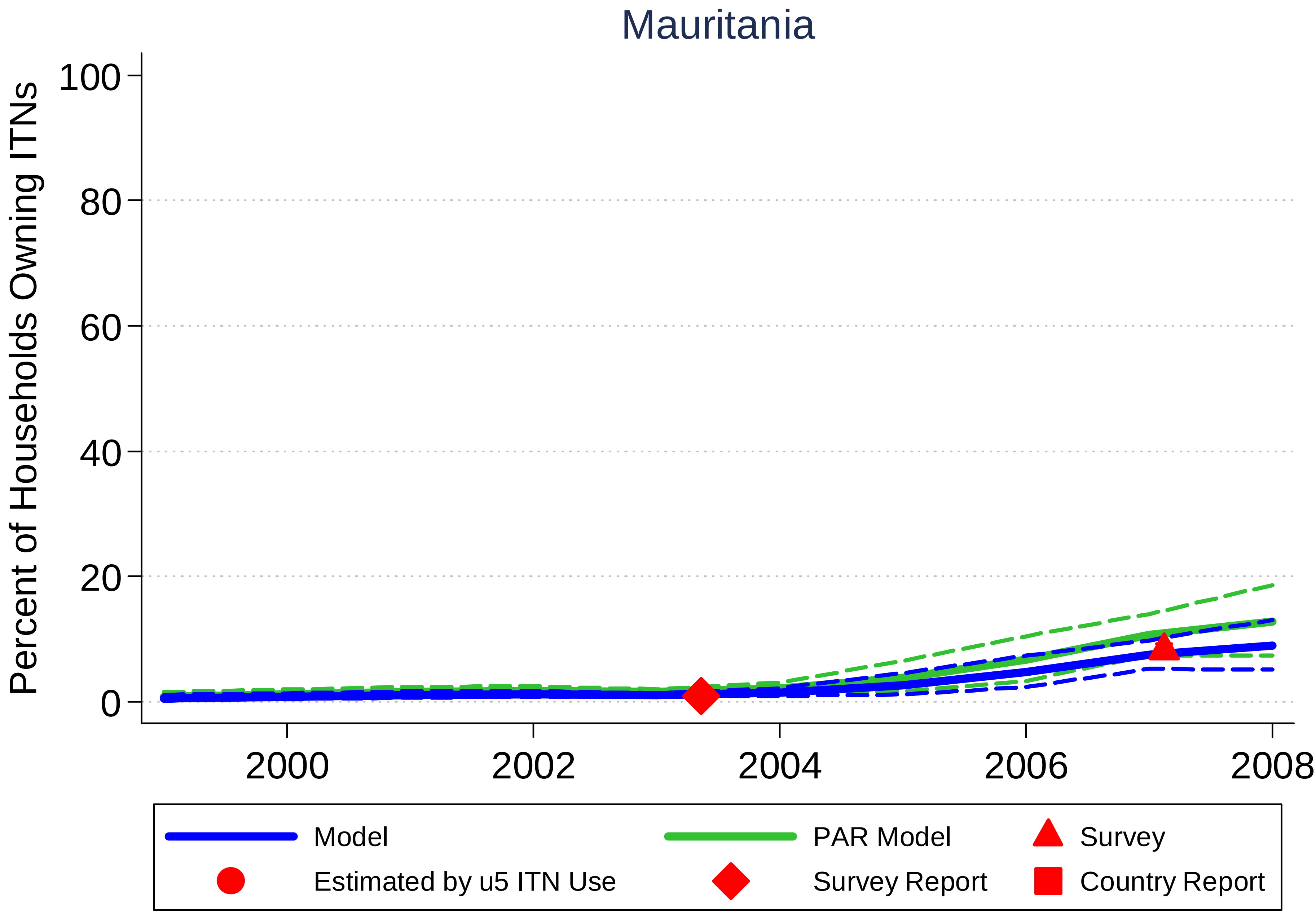
Mauritania
Percent of Households Owning ITNs
0
20
40
60
80
100
2000
2002
2004
2006
2008
Model
PAR Model
Survey
Estimated by u5 ITN Use
Survey Report
Country Report

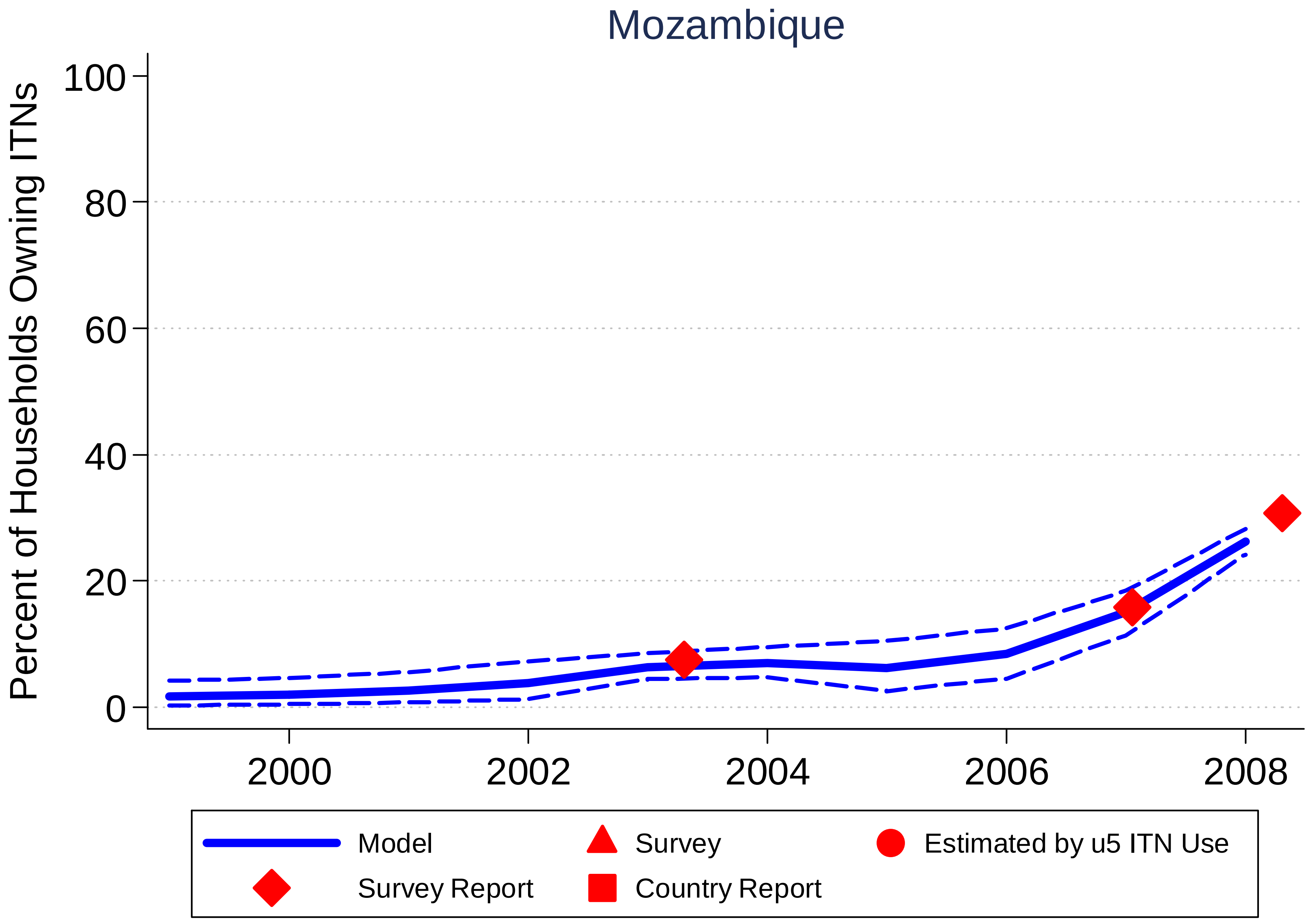
Mozambique
Percent of Households Owning ITNs
100
80
60
40
20
0
2000
2002
2004
2006
2008
Model
Survey
Estimated by u5 ITN Use
Survey Report
Country Report

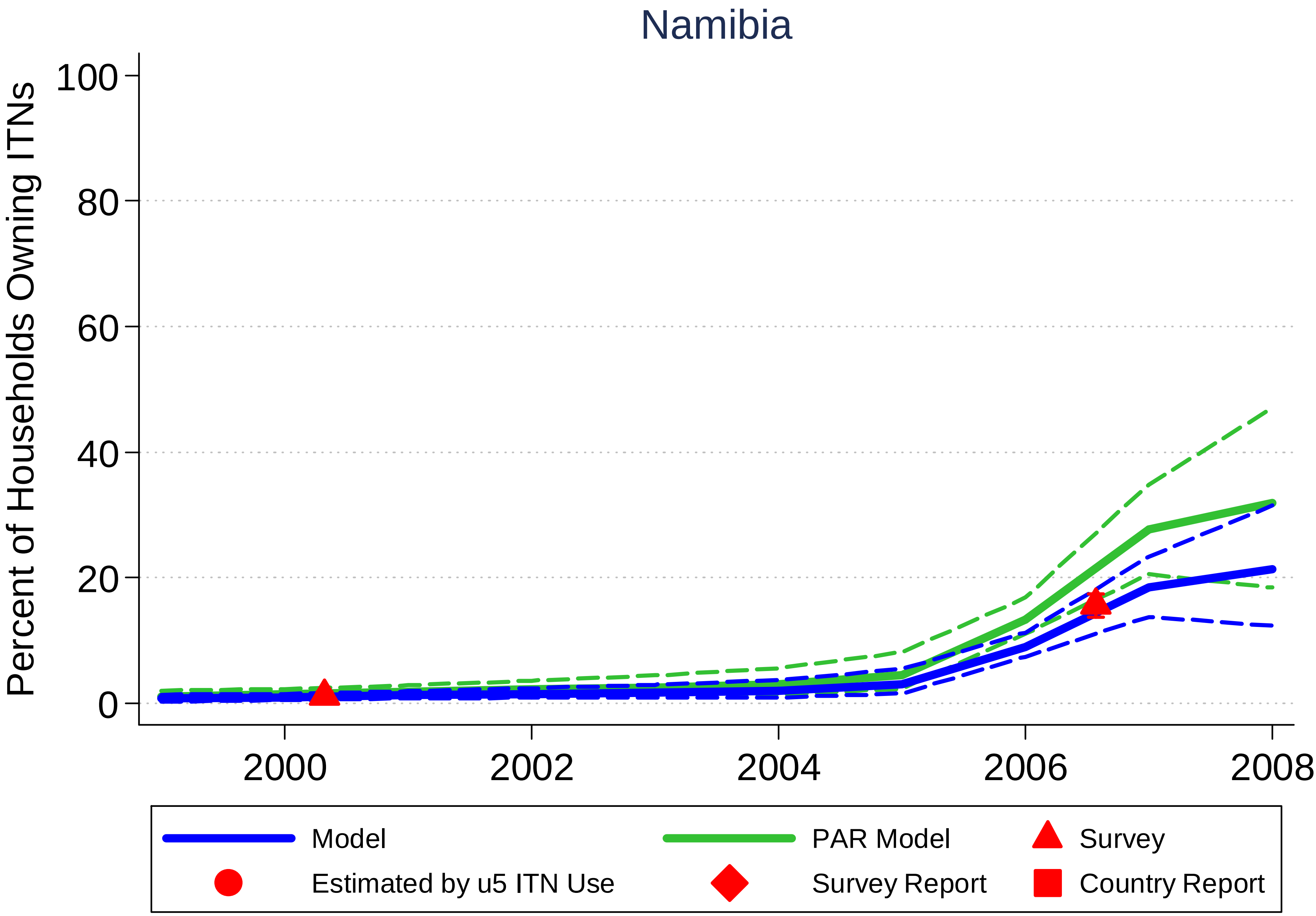

Namibia
Percent of Households Owning ITNs
0
20
40
60
80
100
2000
2002
2004
2006
2008
Model
PAR Model
Survey
Estimated by u5 ITN Use
Survey Report
Country Report

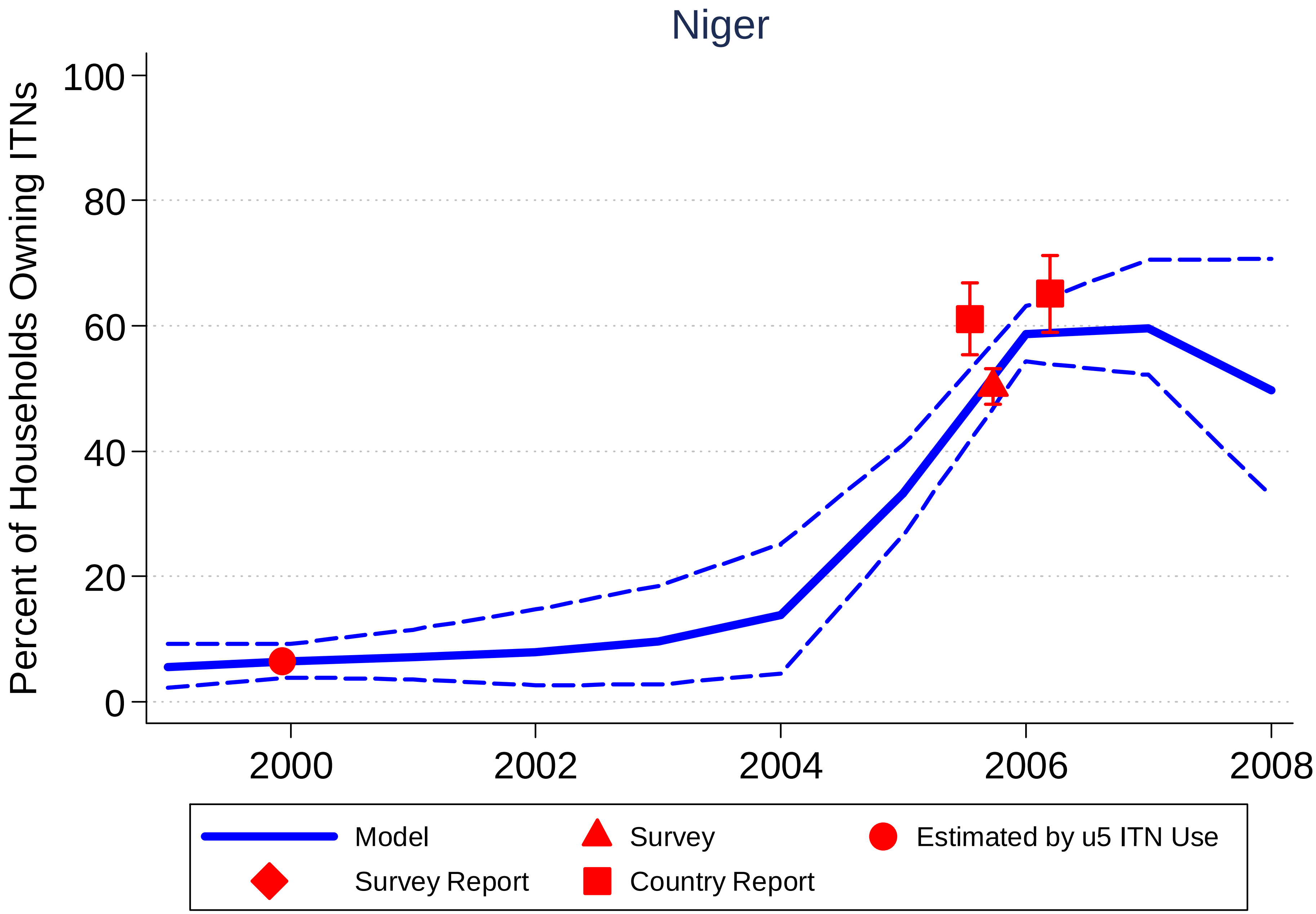

Niger
Percent of Households Owning ITNs
0
20
40
60
80
100
2000
2002
2004
2006
2008
Model
Survey
Estimated by u5 ITN Use
Survey Report
Country Report

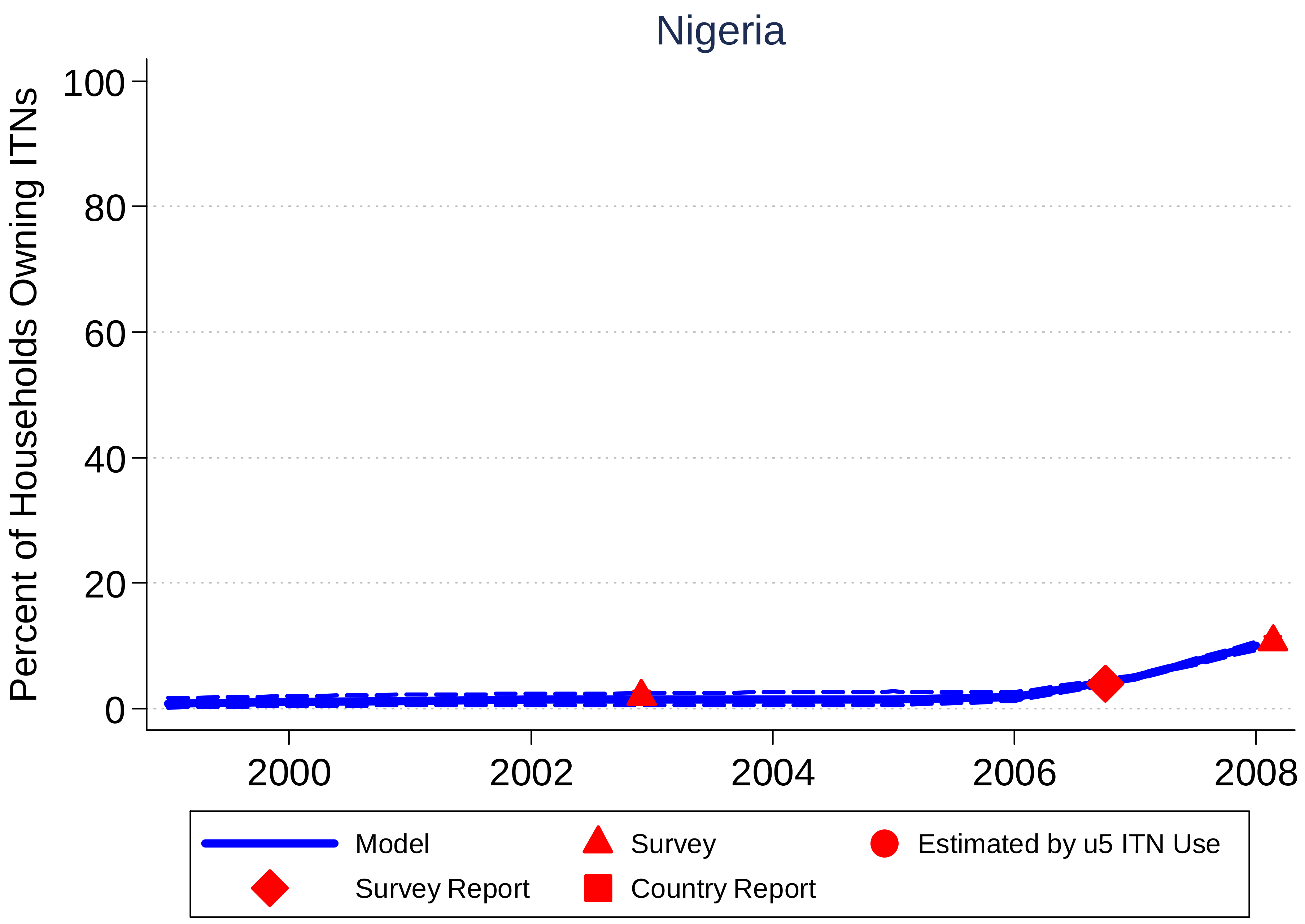
Nigeria
Percent of Households Owning ITNs
0
20
40
60
80
100
2000
2002
2004
2006
2008
Model
Survey
Estimated by u5 ITN Use
Survey Report
Country Report

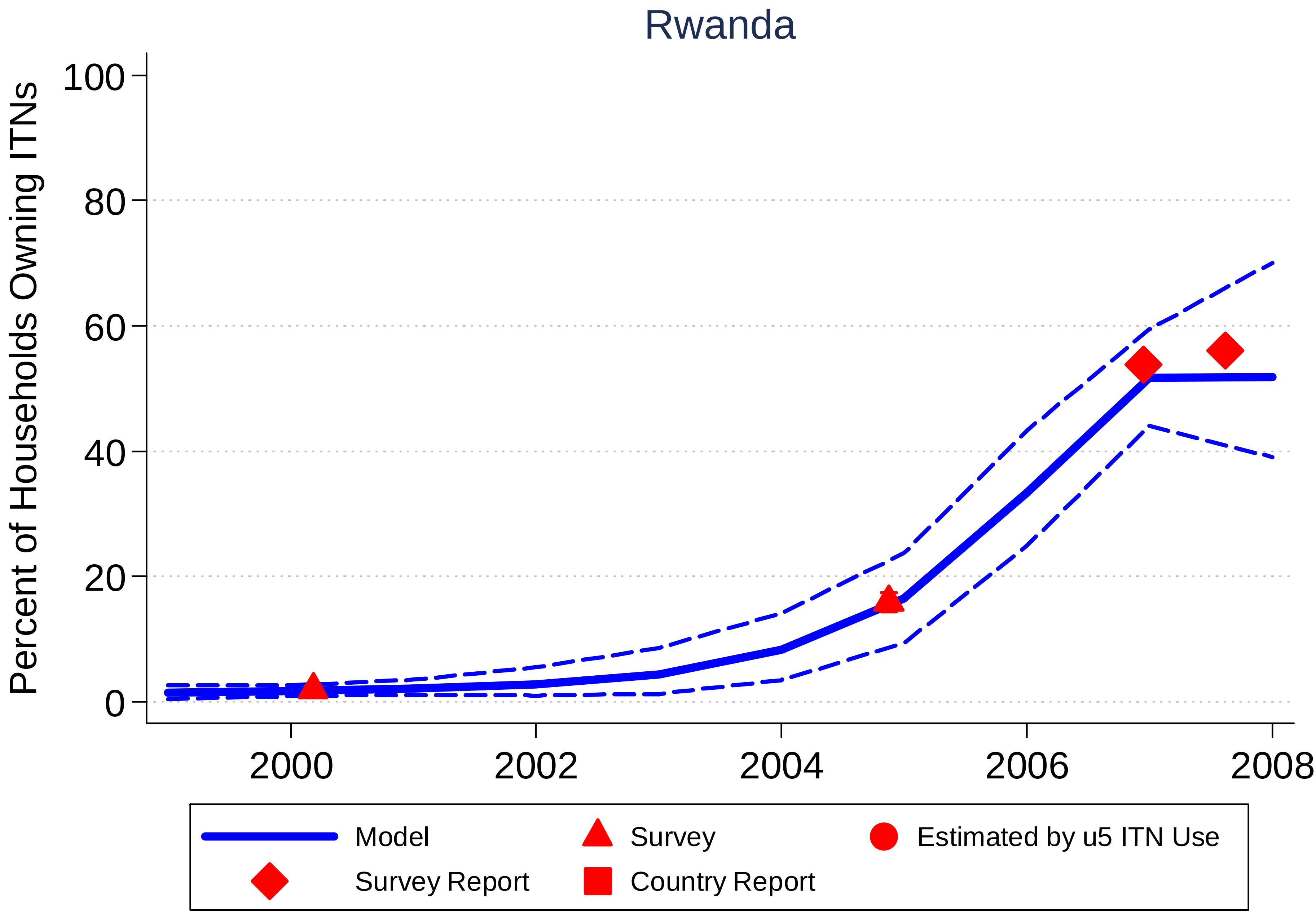

Rwanda
Percent of Households Owning ITNs
0
20
40
60
80
100
2000
2002
2004
2006
2008
Model
Survey
Estimated by u5 ITN Use
Survey Report
Country Report

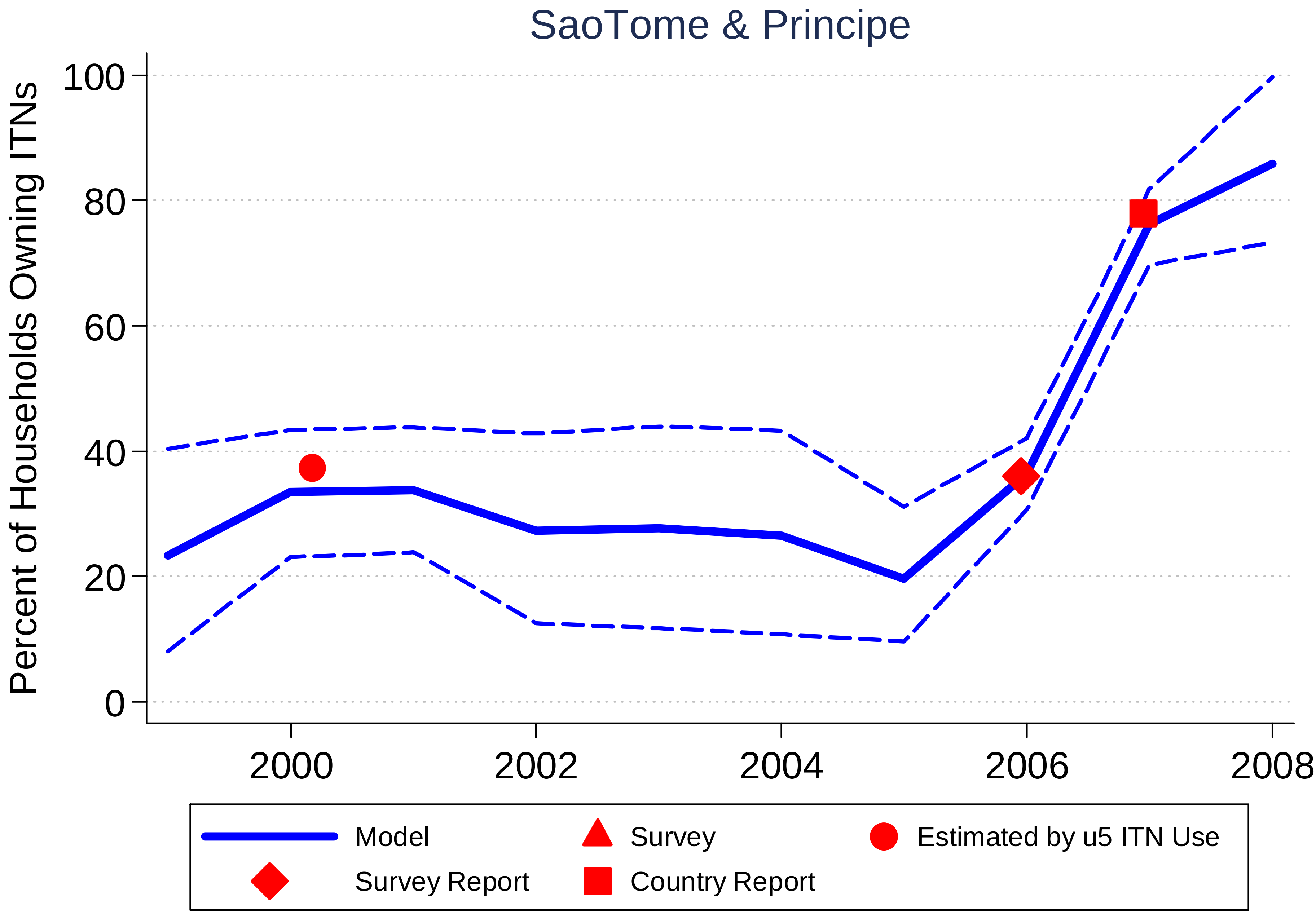

SaoTome & Principe
Percent of Households Owning ITNs
0
20
40
60
80
100
2000
2002
2004
2006
2008
Model
Survey
Estimated by u5 ITN Use
Survey Report
Country Report

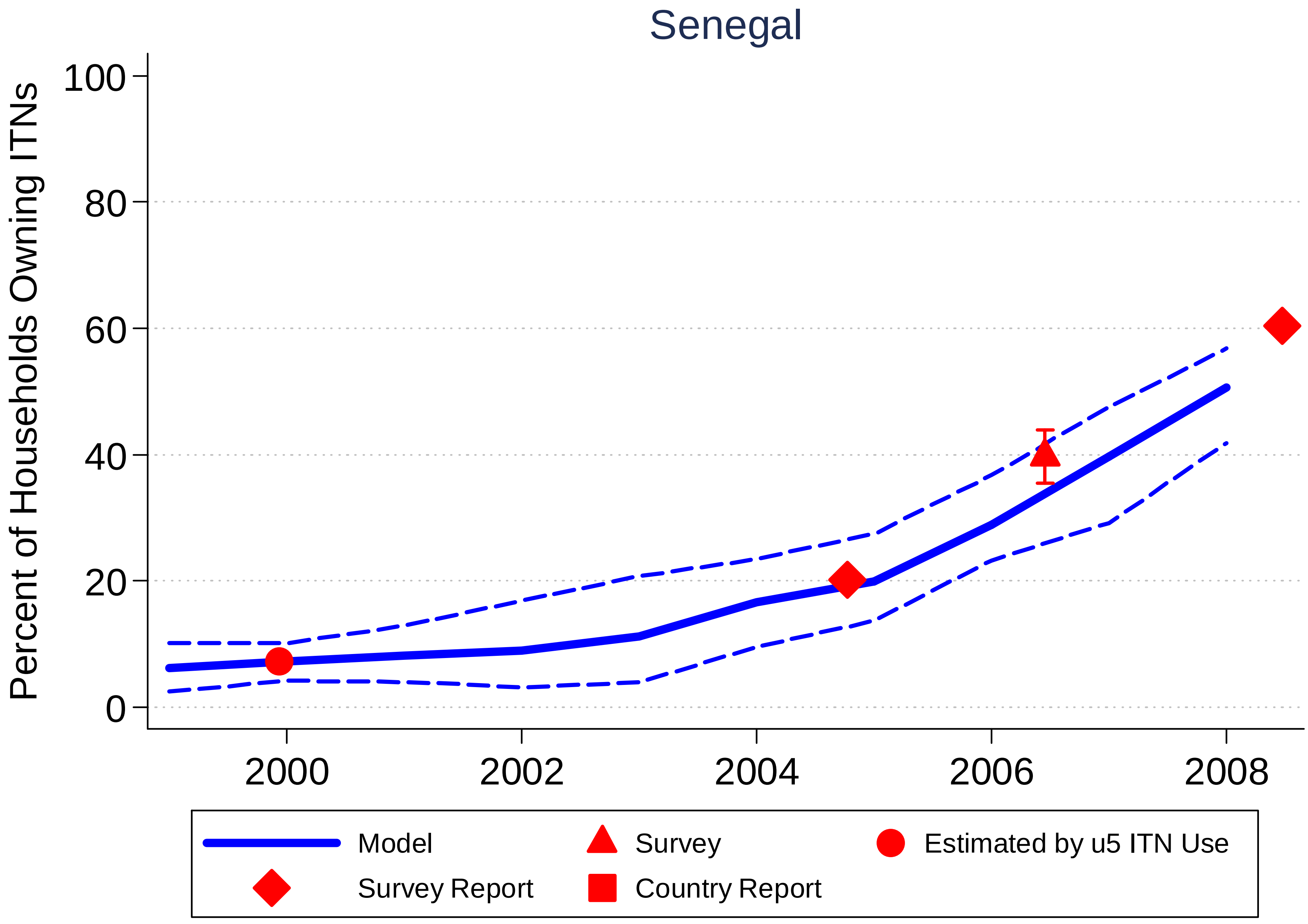
Senegal
Percent of Households Owning ITNs
100
80
60
40
20
0
2000
2002
2004
2006
2008
Model
Survey
Estimated by u5 ITN Use
Survey Report
Country Report

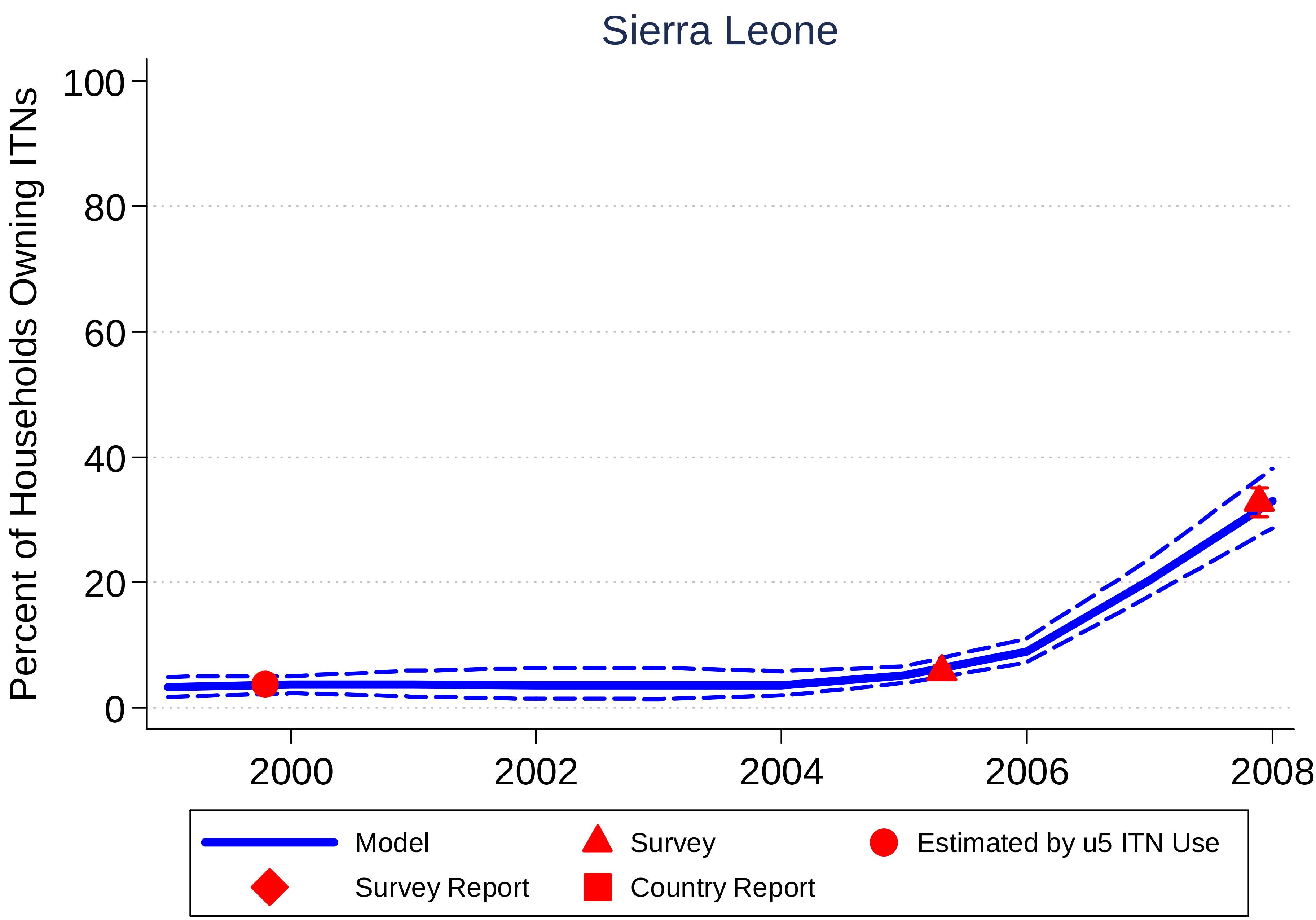

Sierra Leone
Percent of Households Owning ITNs
0
20
40
60
80
100
2000
2002
2004
2006
2008
Model
Survey
Estimated by u5 ITN Use
Survey Report
Country Report

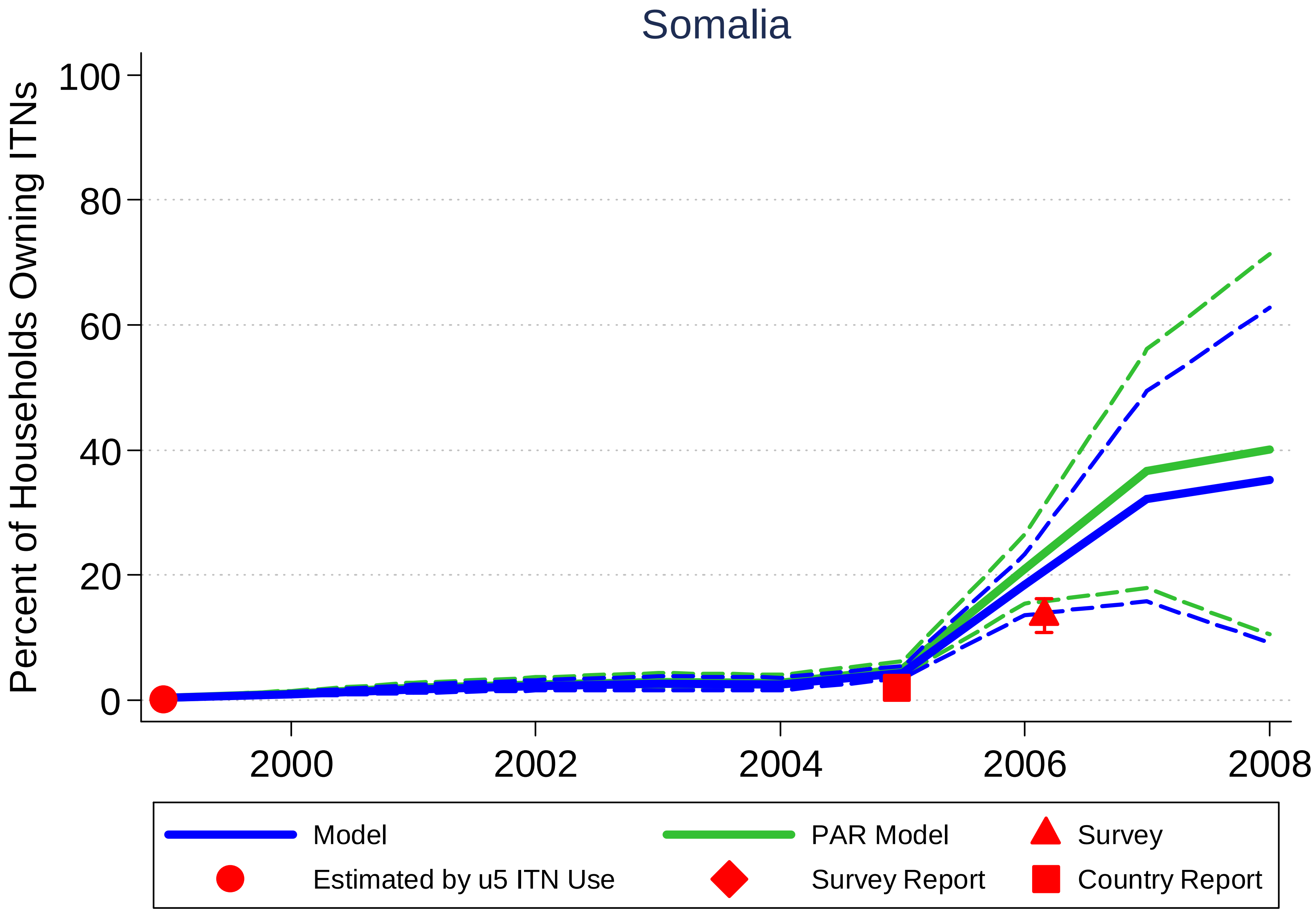
Somalia
Percent of Households Owning ITNs
0
20
40
60
80
100
2000
2002
2004
2006
2008
Model
PAR Model
Survey
Estimated by u5 ITN Use
Survey Report
Country Report

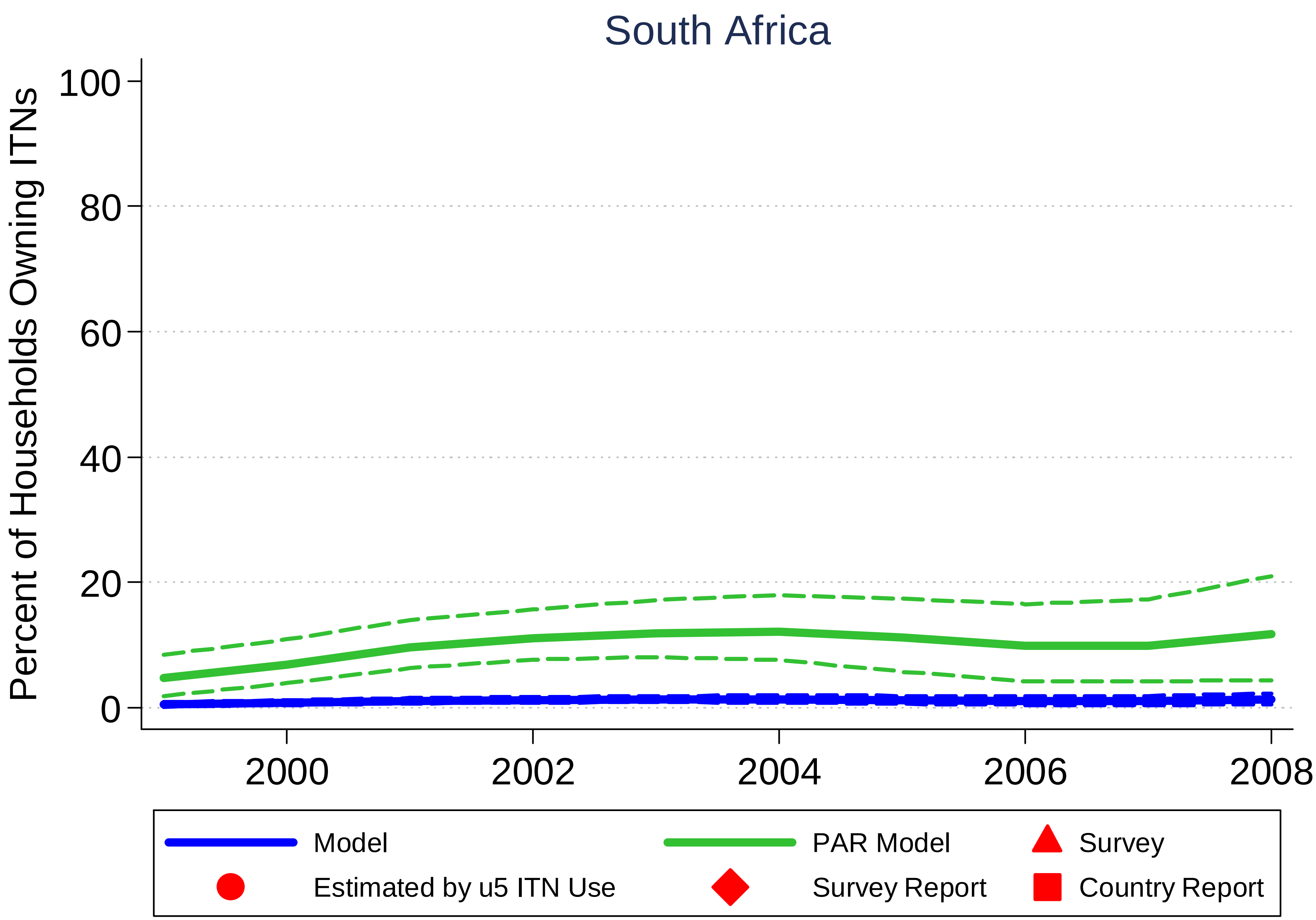

South Africa
Percent of Households Owning ITNs
100
80
60
40
20
0
2000
2002
2004
2006
2008
Model
PAR Model
Survey
Estimated by u5 ITN Use
Survey Report
Country Report

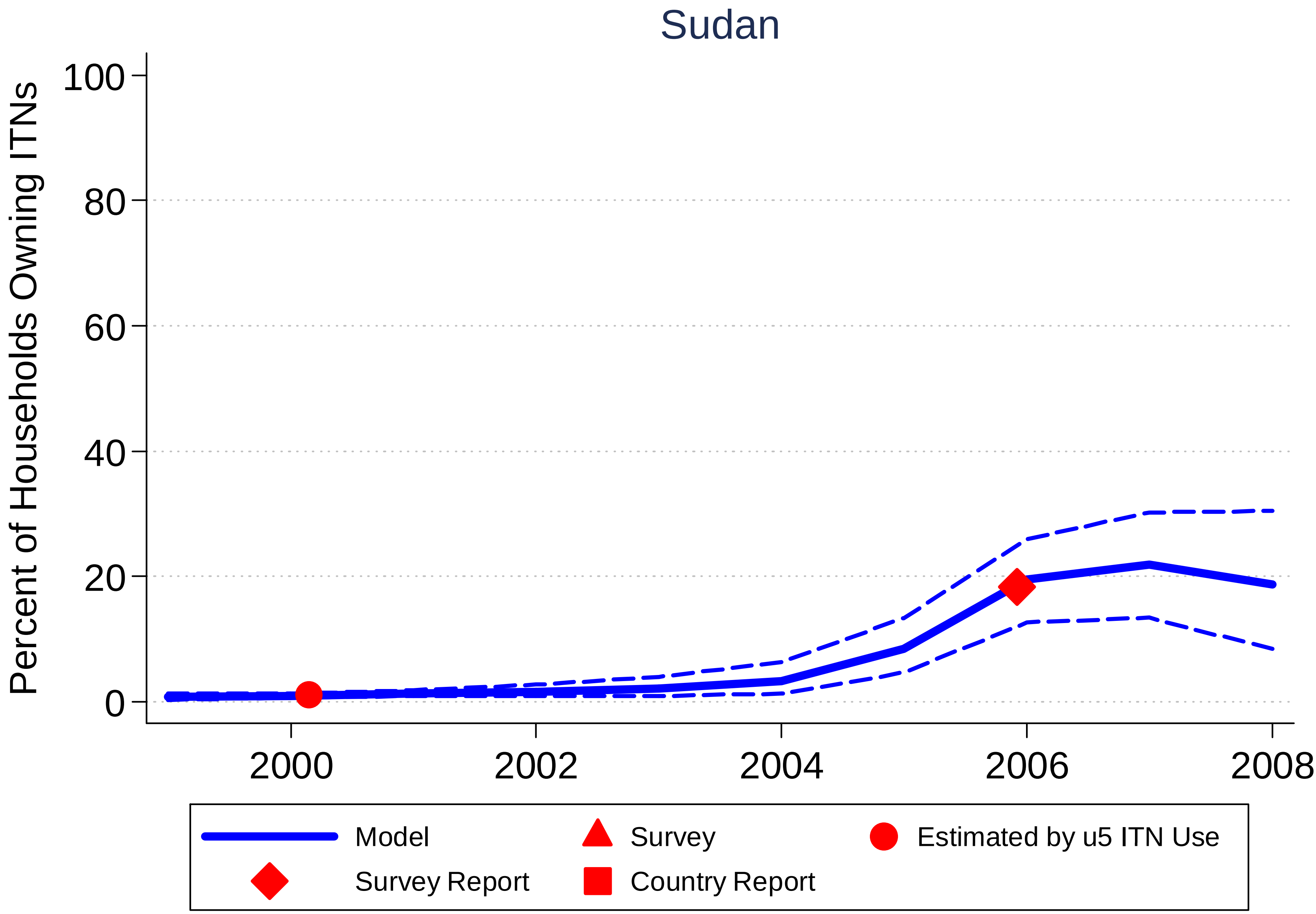
Sudan
Percent of Households Owning ITNs
100
80
60
40
20
0
2000
2002
2004
2006
2008
Model
Survey
Estimated by u5 ITN Use
Survey Report
Country Report

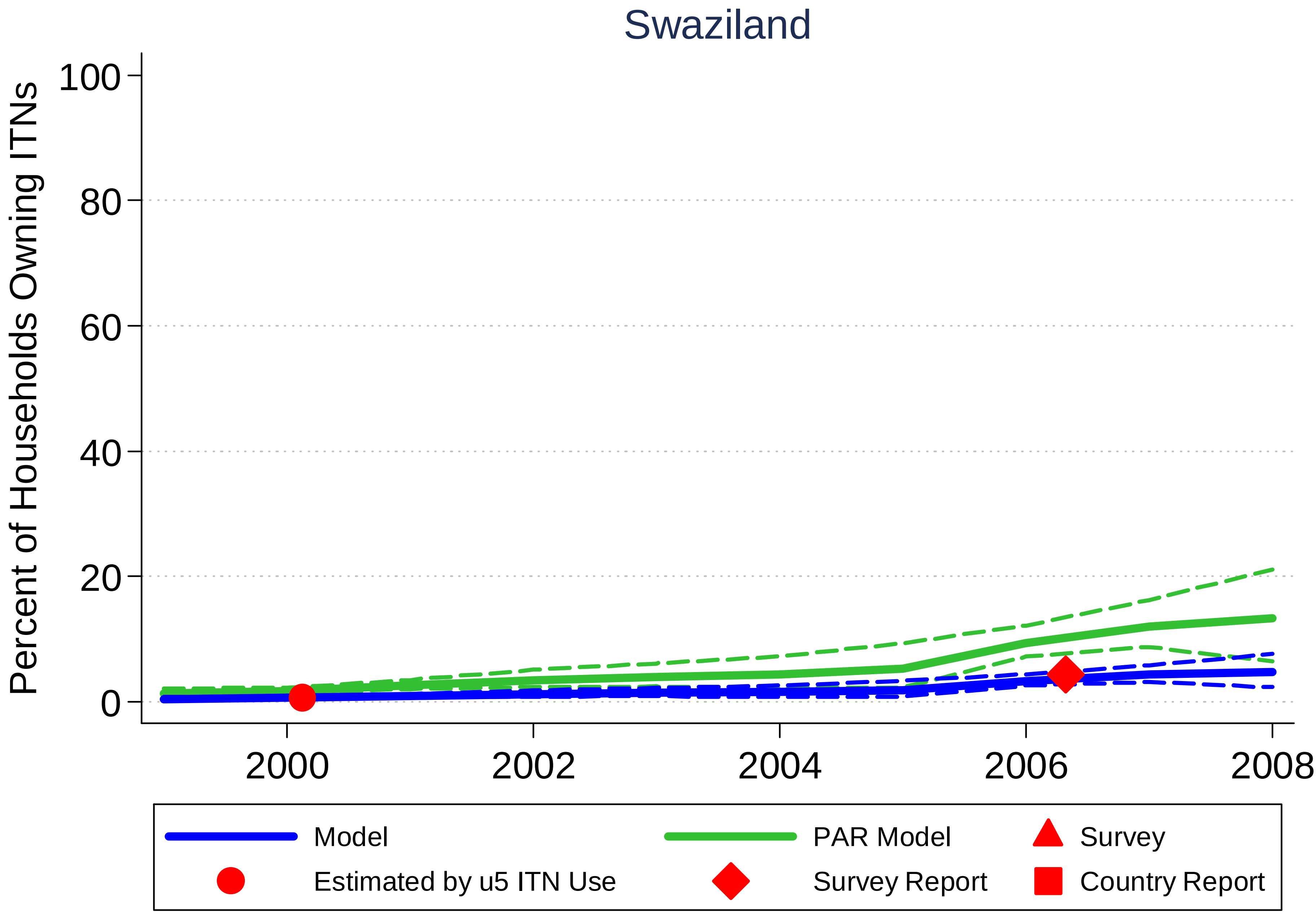

Swaziland
Percent of Households Owning ITNs
0
20
40
60
80
100
2000
2002
2004
2006
2008
Model
PAR Model
Survey
Estimated by u5 ITN Use
Survey Report
Country Report

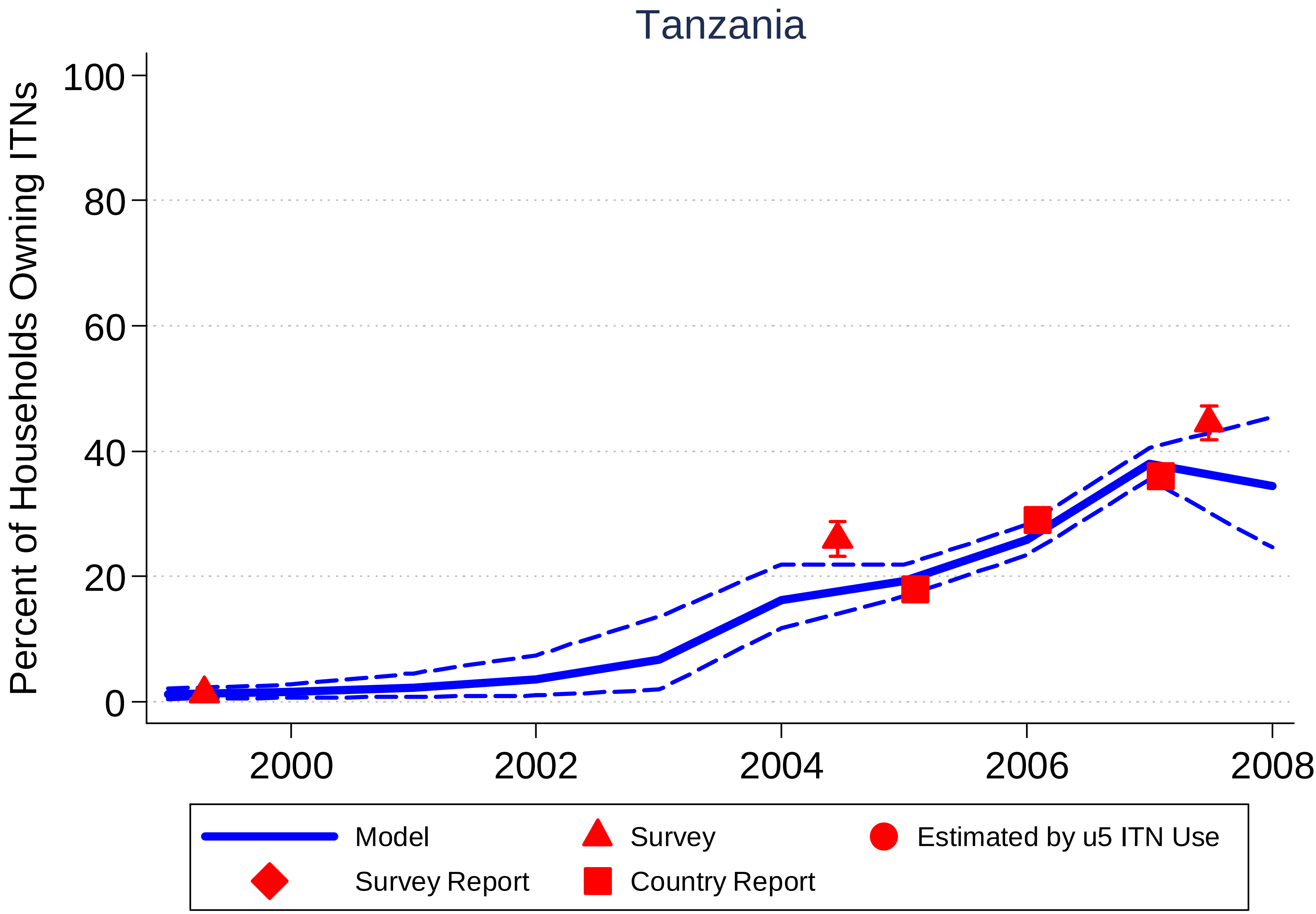
Tanzania
Percent of Households Owning ITNs
0
20
40
60
80
100
2000
2002
2004
2006
2008
Model
Survey
Estimated by u5 ITN Use
Survey Report
Country Report

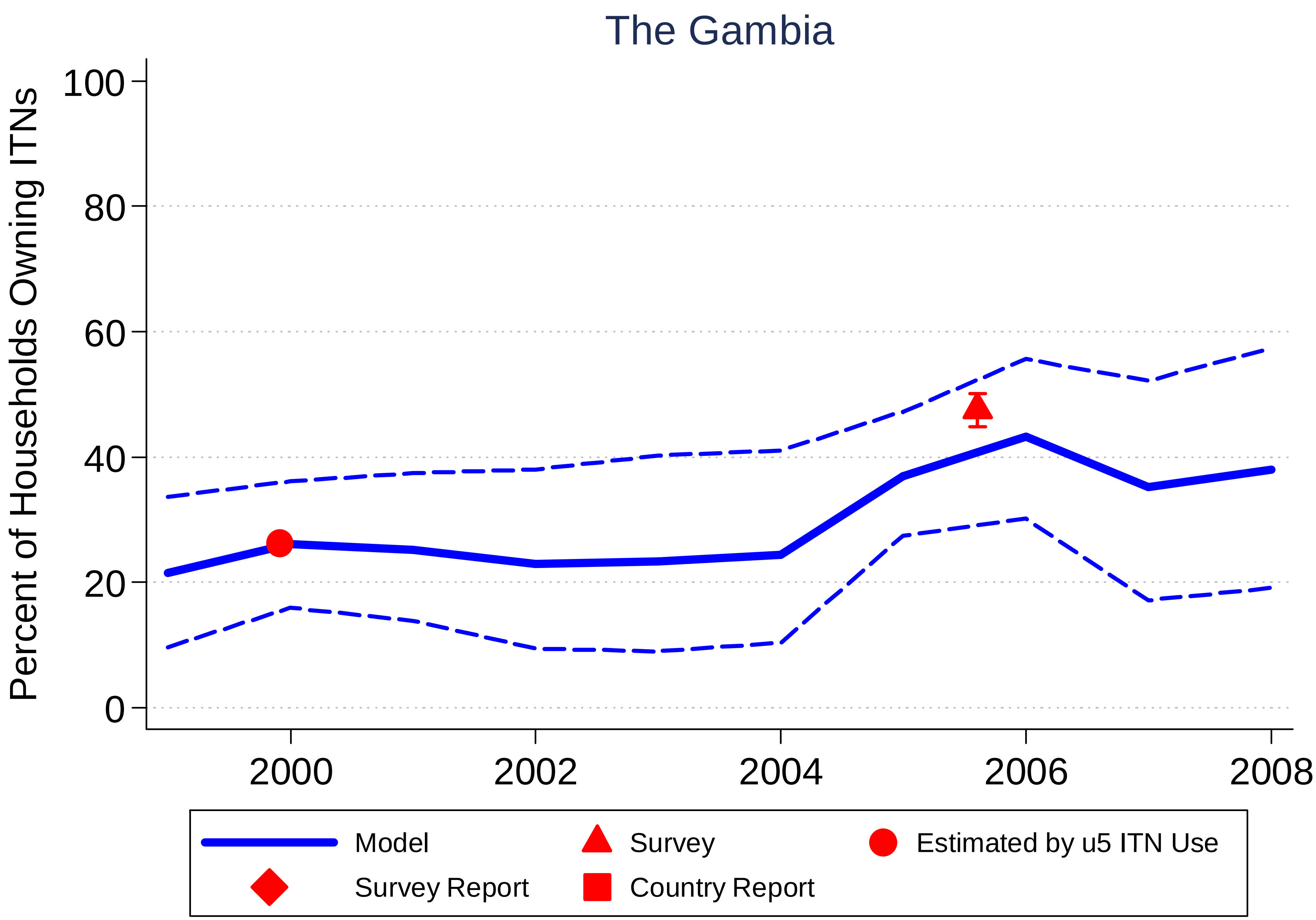
The Gambia
Percent of Households Owning ITNs
100
80
60
40
20
0
2000
2002
2004
2006
2008
Model
Survey
Estimated by u5 ITN Use
Survey Report
Country Report

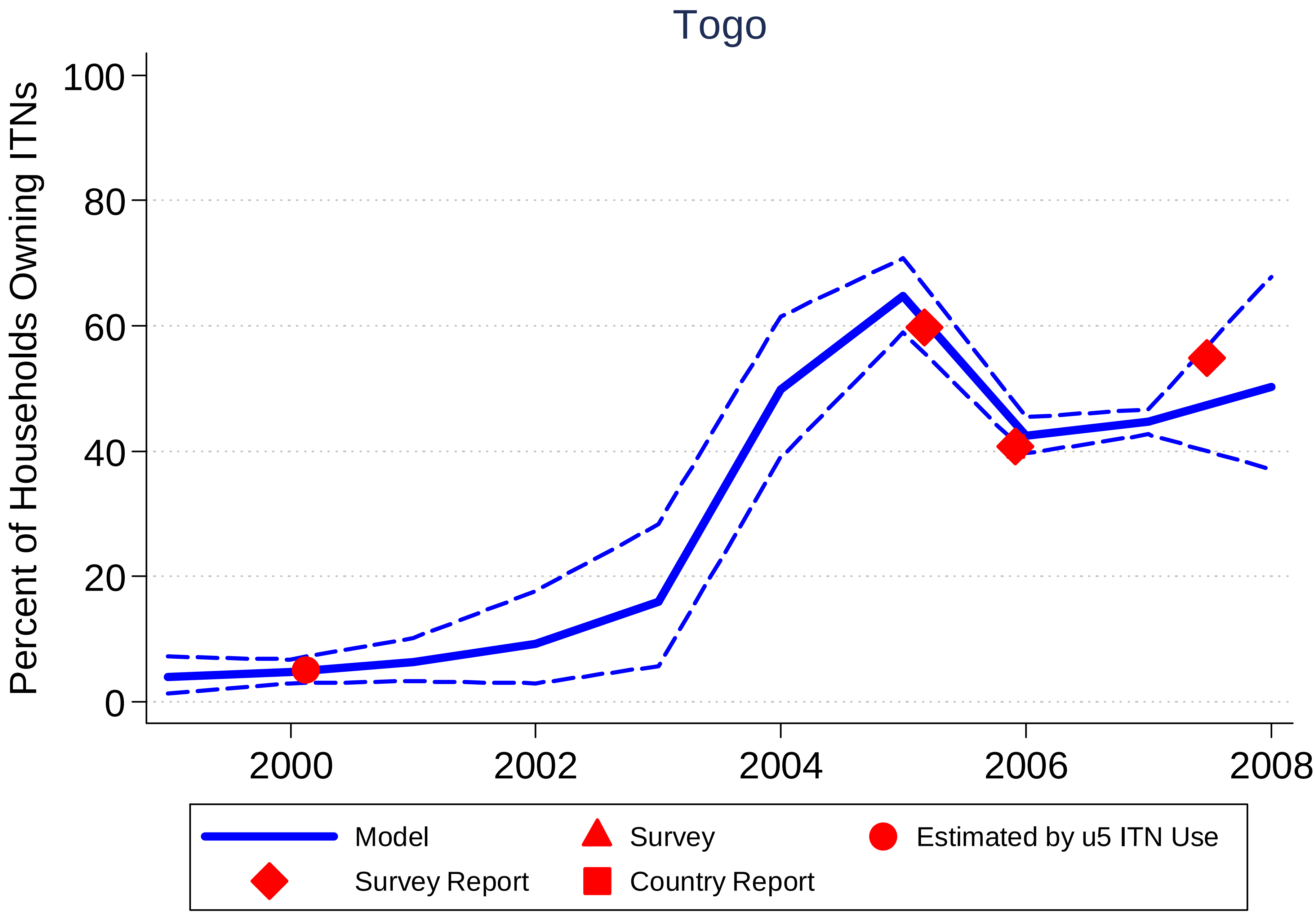

Togo
Percent of Households Owning ITNs
100
80
60
40
20
0
2000
2002
2004
2006
2008
Model
Survey
Estimated by u5 ITN Use
Survey Report
Country Report

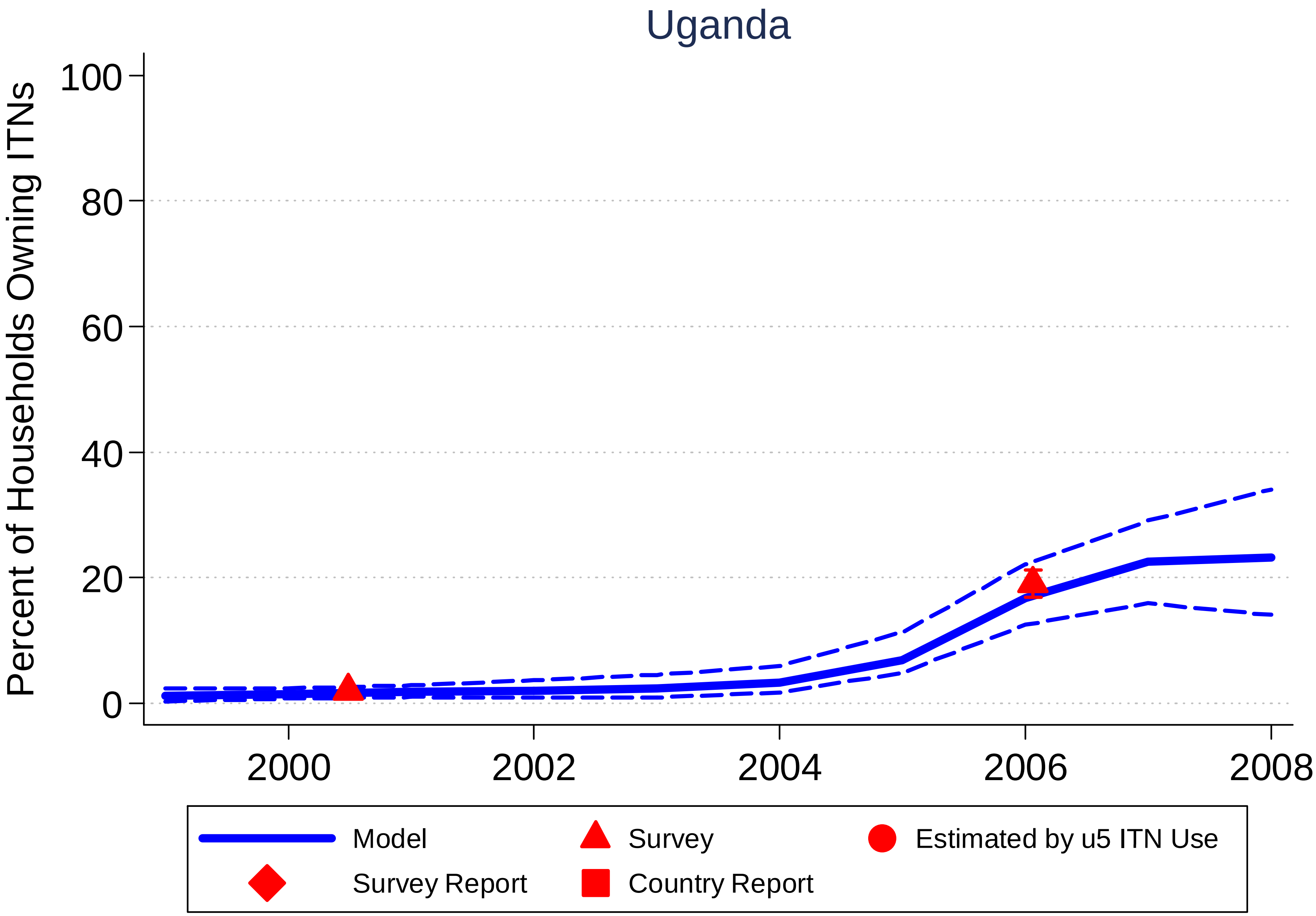

Uganda
Percent of Households Owning ITNs
100
80
60
40
20
0
2000
2002
2004
2006
2008
Model
Survey
Estimated by u5 ITN Use
Survey Report
Country Report

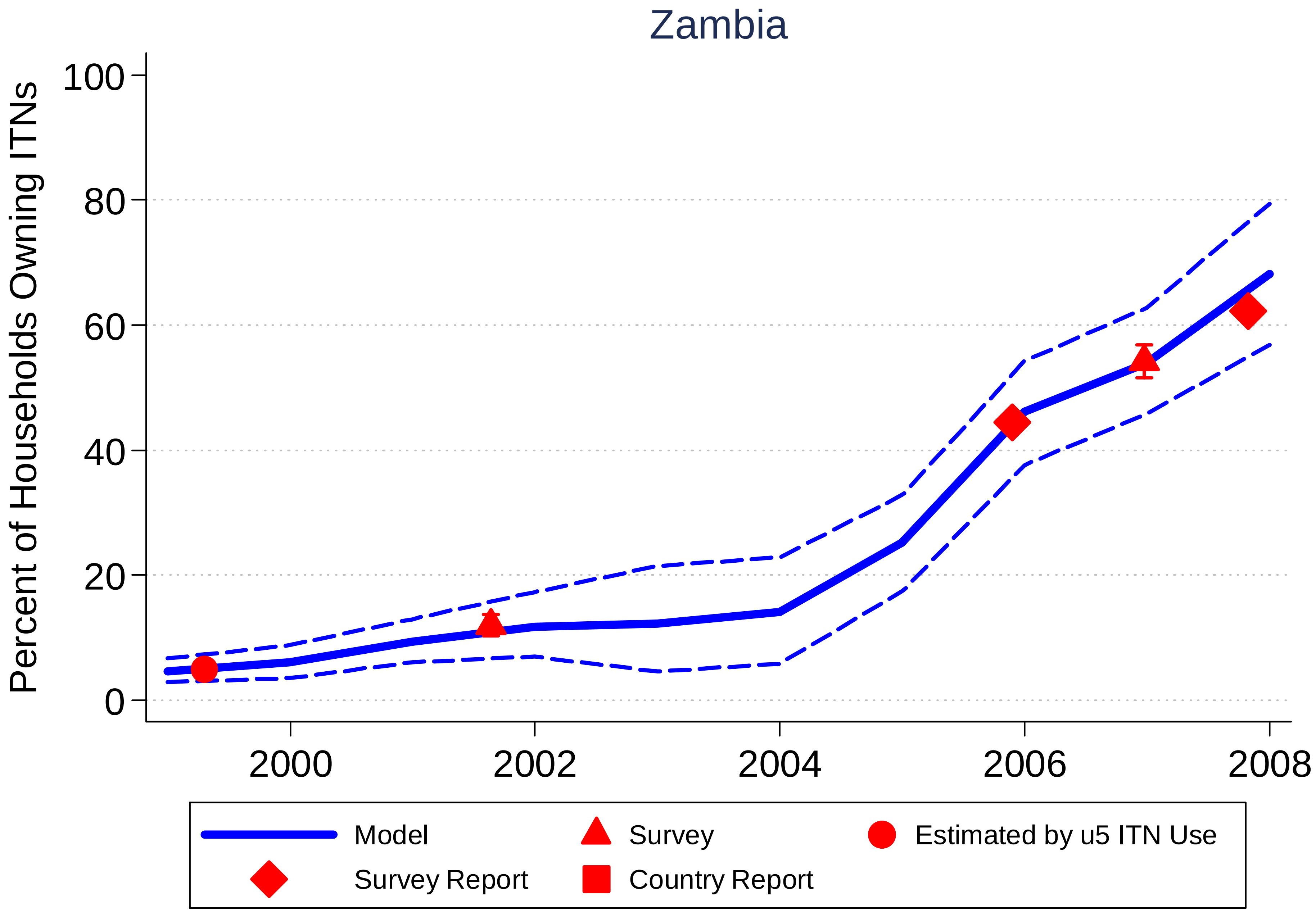
Zambia
Percent of Households Owning ITNs
100
80
60
40
20
0
2000
2002
2004
2006
2008
Model
Survey
Estimated by u5 ITN Use
Survey Report
Country Report

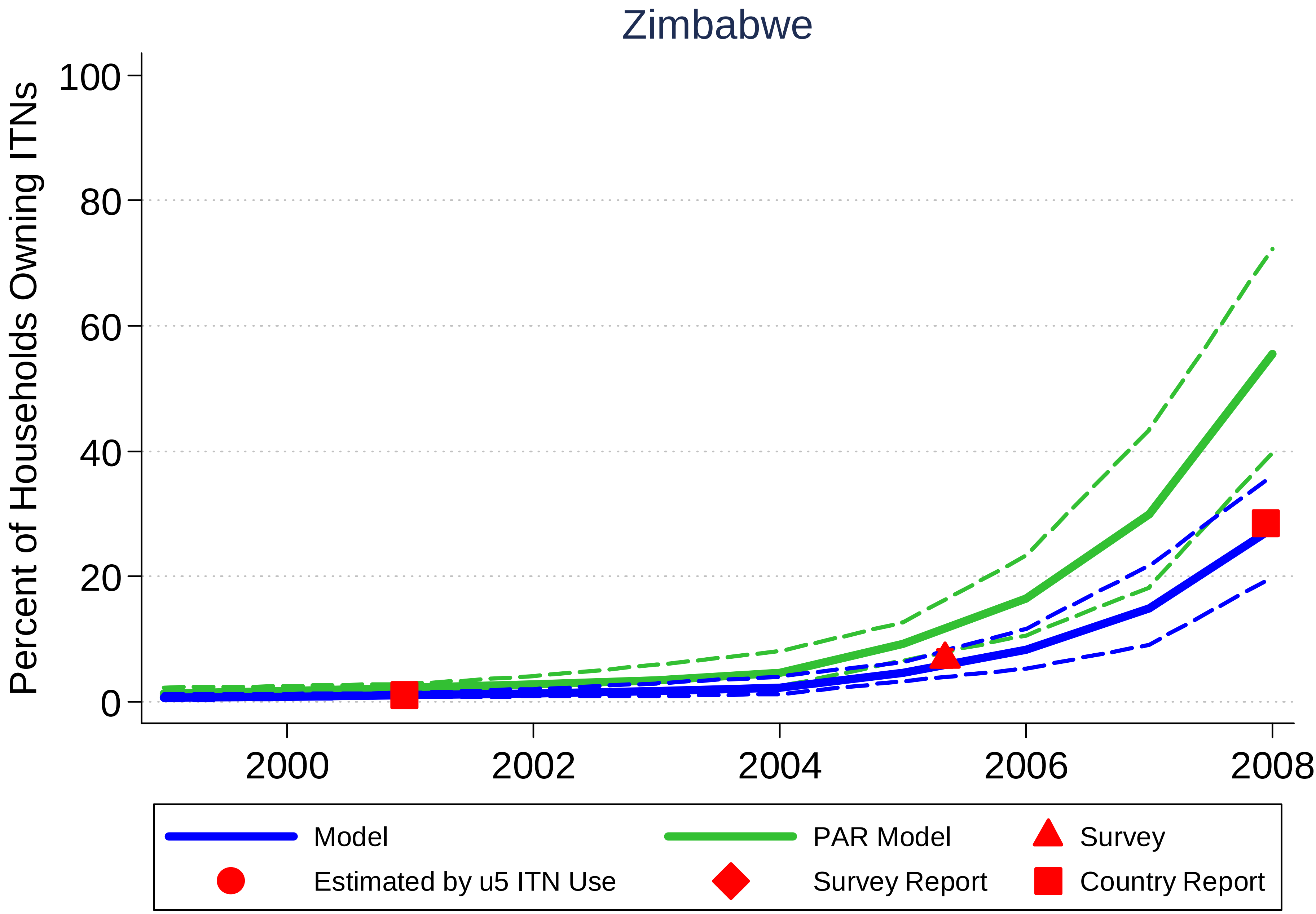

Zimbabwe
Percent of Households Owning ITNs
100
80
60
40
20
0
2000
2002
2004
2006
2008
Model
PAR Model
Survey
Estimated by u5 ITN Use
Survey Report
Country Report
