# Supplementary material for: Rapid Scaling Up of Insecticide-Treated Bed Net Coverage in Africa and Its Relationship with Development Assistance for Health: A Systematic Synthesis of Supply, Distribution, and Household Survey Data
Source: PLoS Med. 2010 Aug 17;7(8):e1000328. doi: 10.1371/journal.pmed.1000328 (PMC2923089; doi:10.1371/journal.pmed.1000328)
Supplement: Figure S2 — Data and model estimates of ITN use in children under 5 coverage for 44 African countries. (0.22 MB PDF) [file pmed.1000328.s002.pdf]

Figure S2. Data and model estimates of ITN use in children under 5 coverage for 44 African countries. Data are: survey estimates during the rainy season (red diamonds); and survey estimates during the dry season (yellow diamonds). Model estimates at the national-level are shown as blue lines with dotted lines indicating 95% uncertainty intervals. Model estimates for the population at risk malaria are shown as green lines with dotted lines indicating 95% uncertainty intervals. Years indicate the mid-point of the calendar year. Note that not all symbols are present for each country.

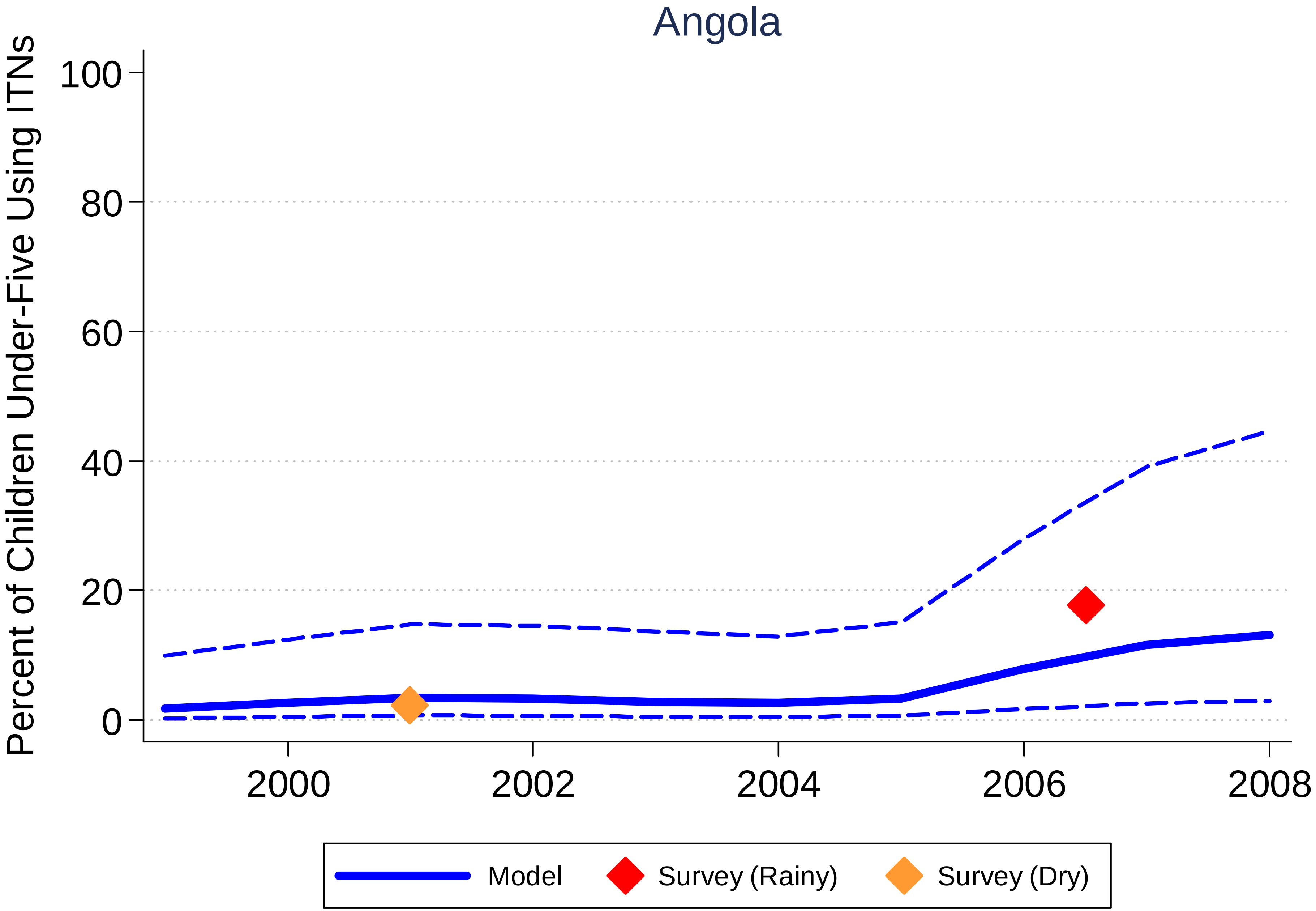
Angola
Percent of Children Under-Five Using ITNs
100
80
60
40
20
0
2000
2002
2004
2006
2008
Model
Survey (Rainy)
Survey (Dry)

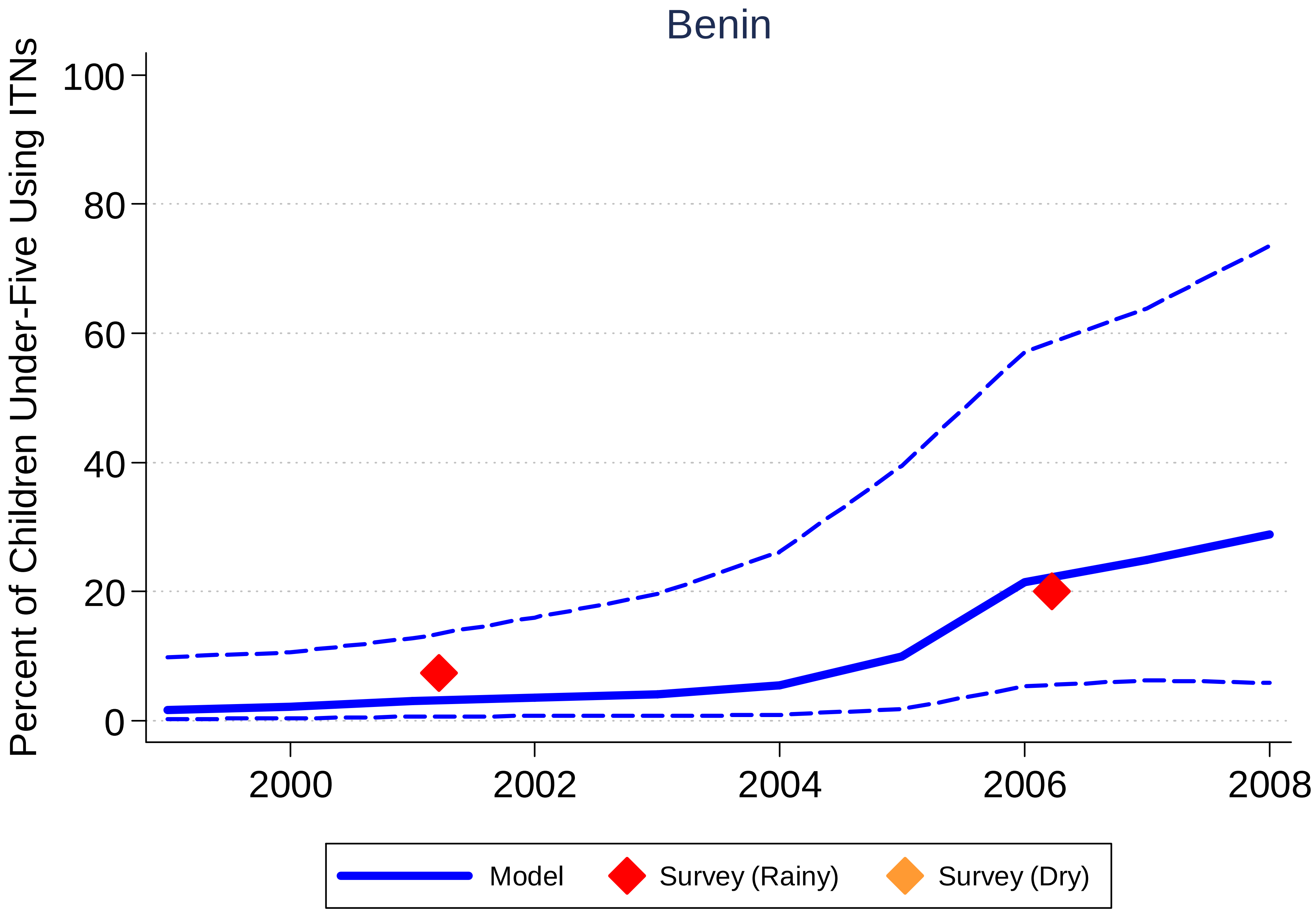
Benin
Percent of Children Under-Five Using ITNs
0
20
40
60
80
100
2000
2002
2004
2006
2008
Model
Survey (Rainy)
Survey (Dry)

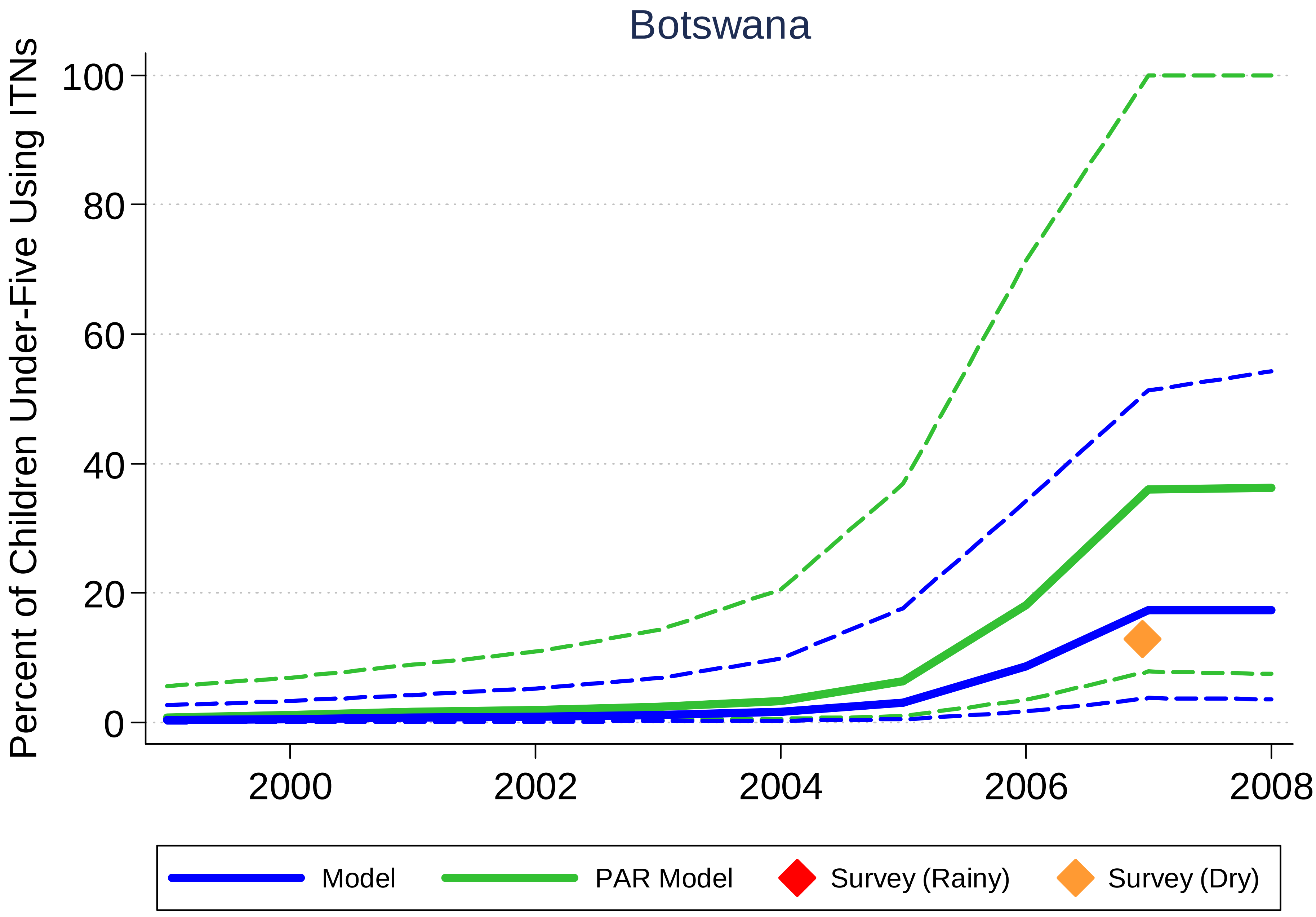
Botswana
Percent of Children Under-Five Using ITNs
0
20
40
60
80
100
2000
2002
2004
2006
2008
Model
PAR Model
Survey (Rainy)
Survey (Dry)

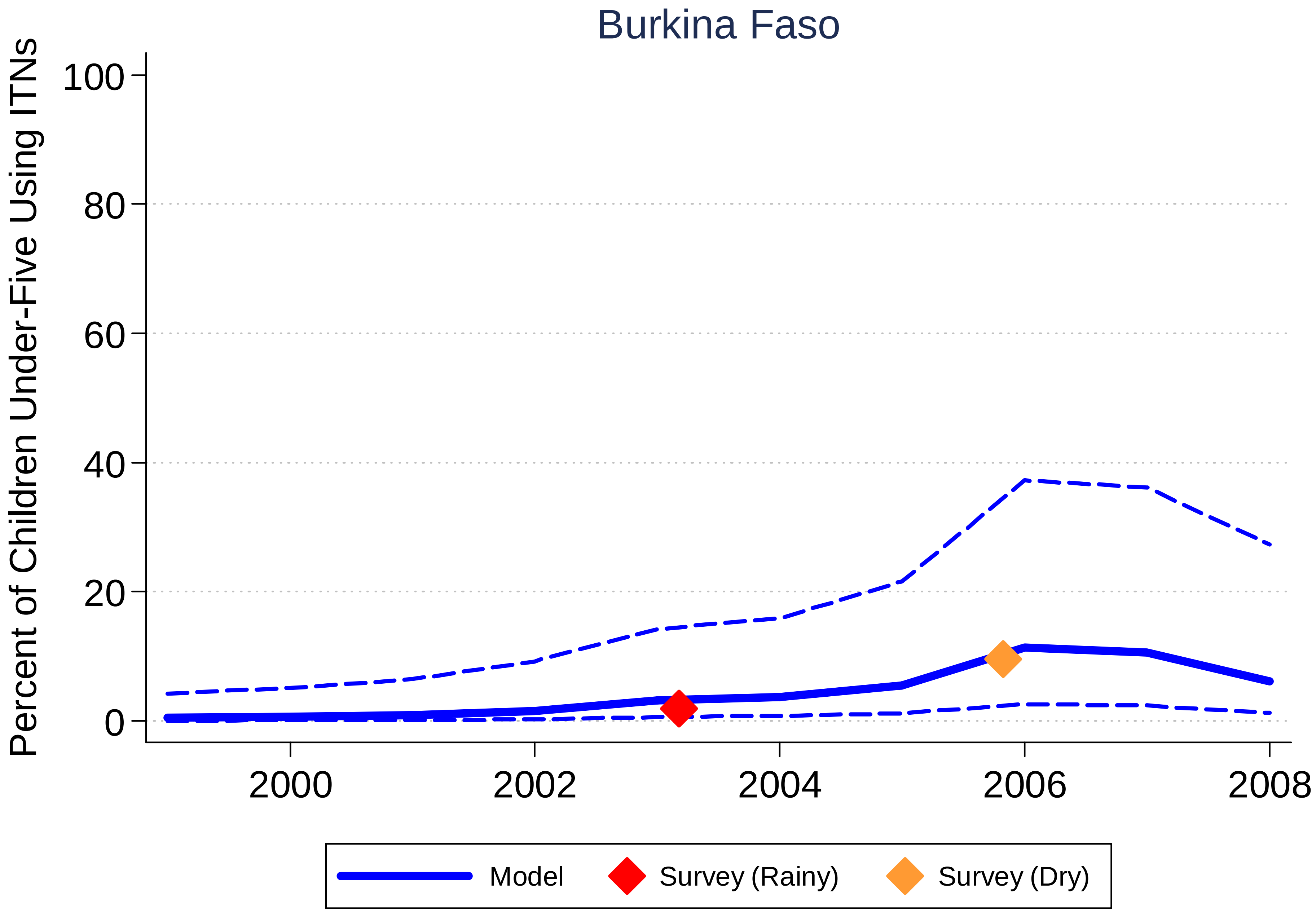

Burkina Faso
Percent of Children Under-Five Using ITNs
0
20
40
60
80
100
2000
2002
2004
2006
2008
Model
Survey (Rainy)
Survey (Dry)

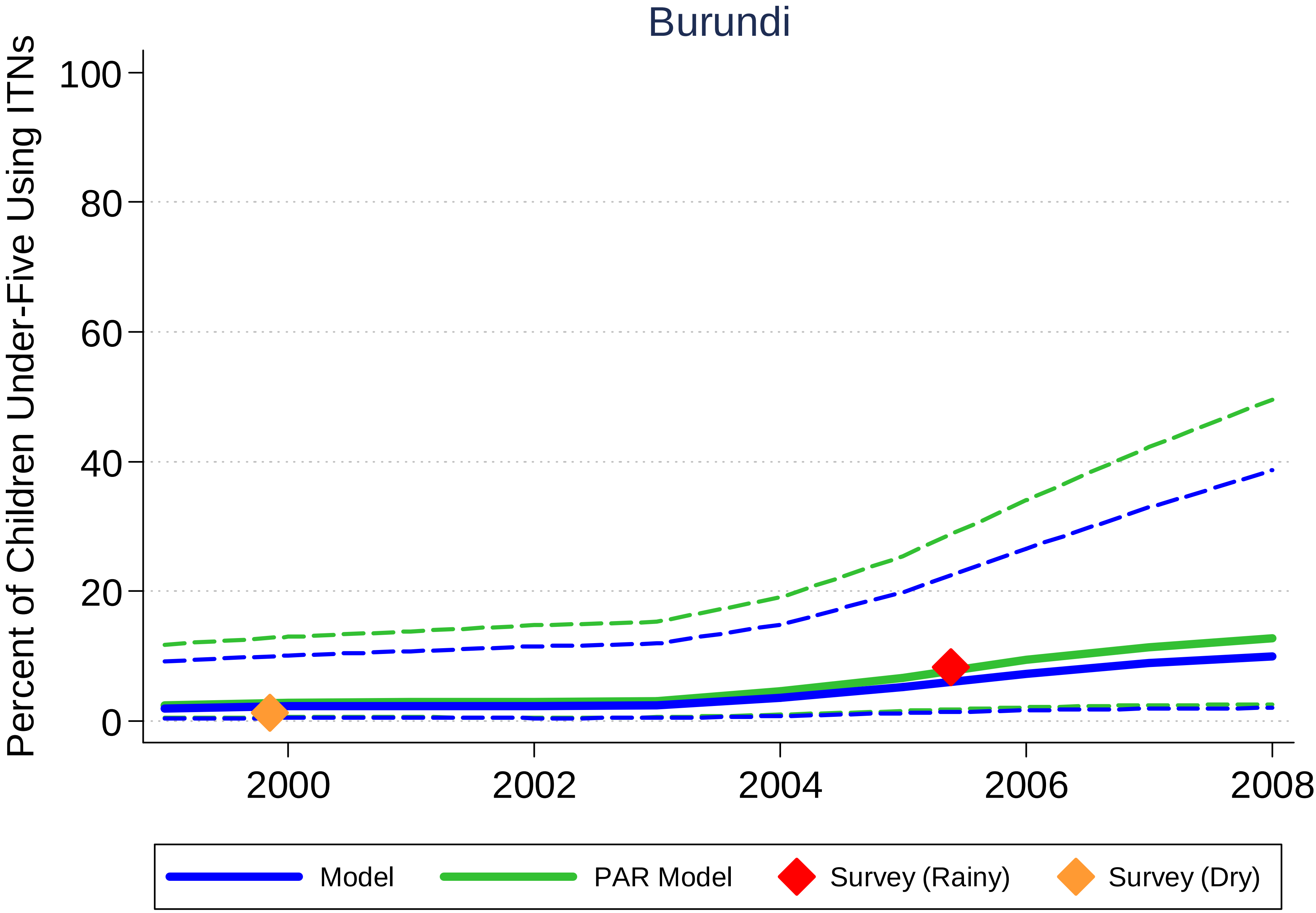
Burundi
Percent of Children Under-Five Using ITNs
100
80
60
40
20
0
2000
2002
2004
2006
2008
Model
PAR Model
Survey (Rainy)
Survey (Dry)

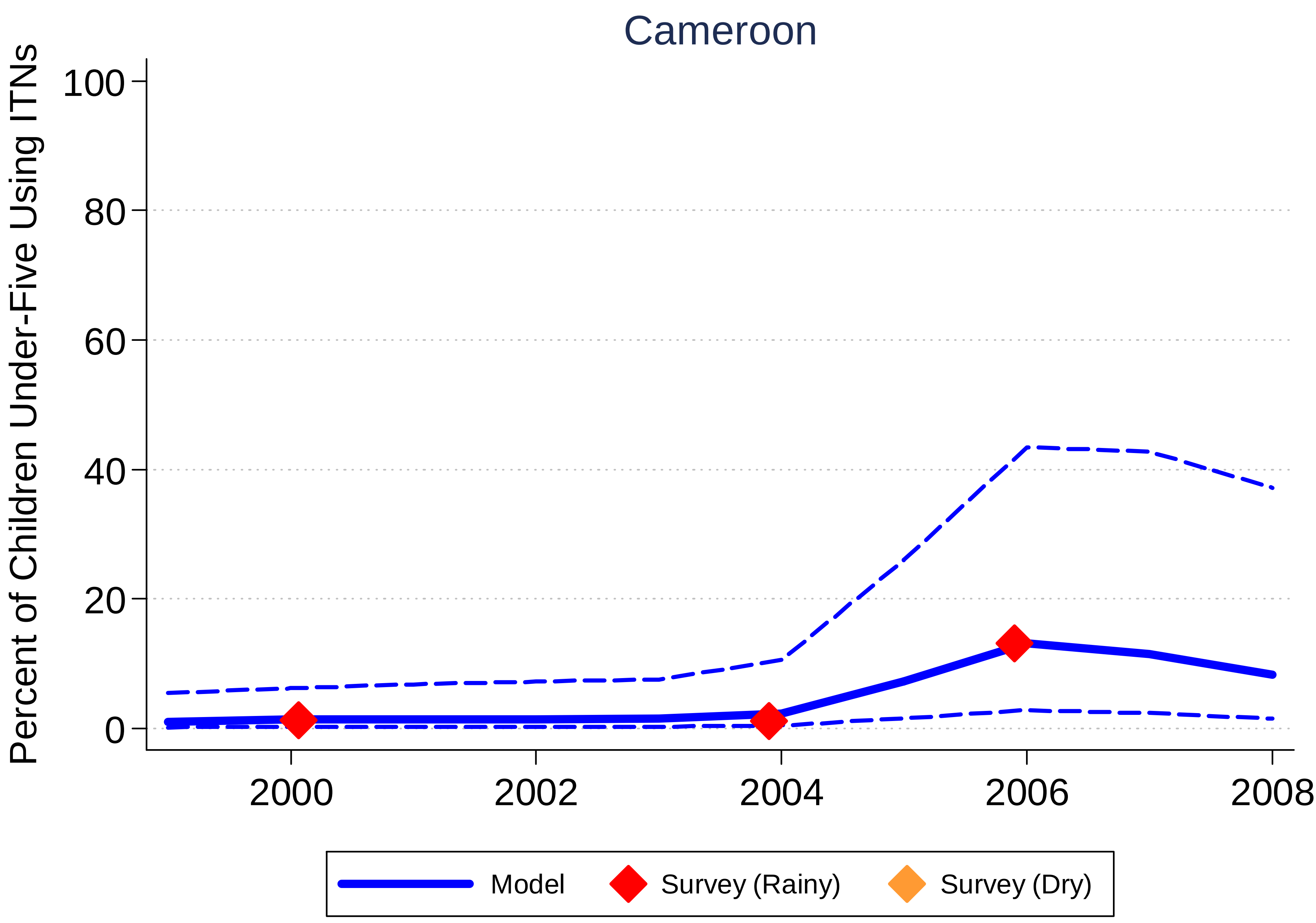

Cameroon
Percent of Children Under-Five Using ITNs
0
20
40
60
80
100
2000
2002
2004
2006
2008
Model
Survey (Rainy)
Survey (Dry)

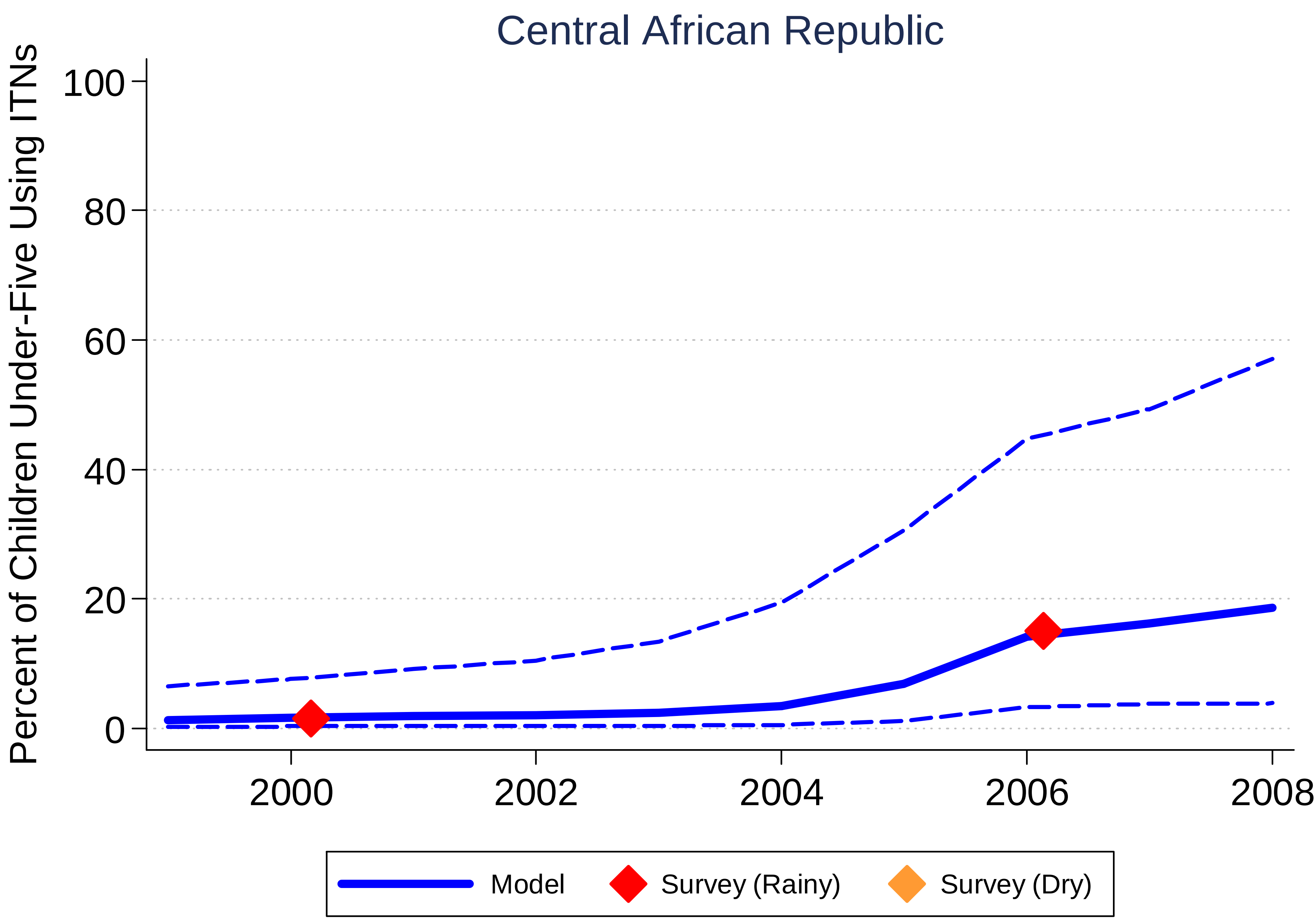

Central African Republic
Percent of Children Under-Five Using ITNs
100
80
60
40
20
0
2000
2002
2004
2006
2008
Model
Survey (Rainy)
Survey (Dry)

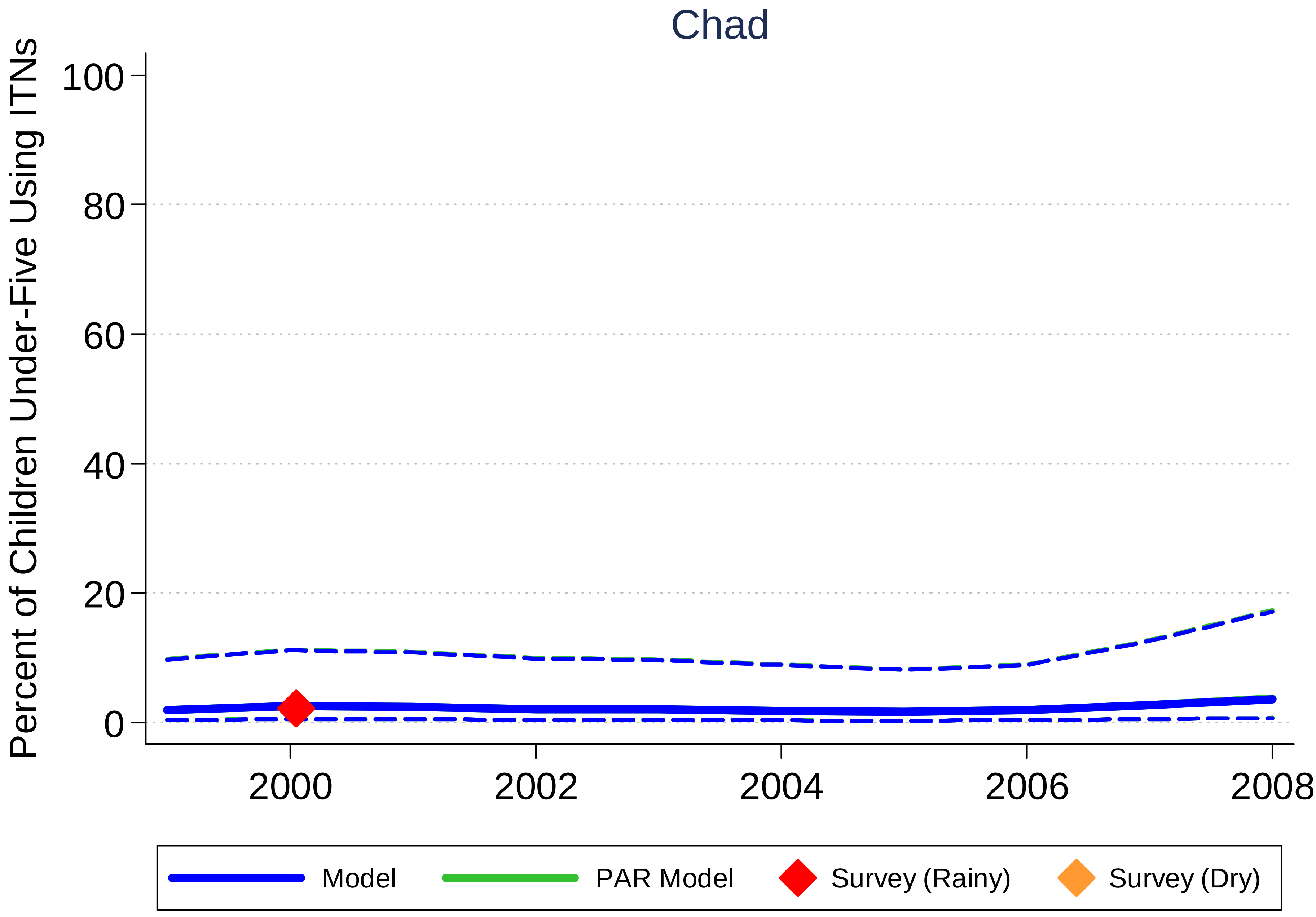

Chad
Percent of Children Under-Five Using ITNs
100
80
60
40
20
0
2000
2002
2004
2006
2008
Model
PAR Model
Survey (Rainy)
Survey (Dry)

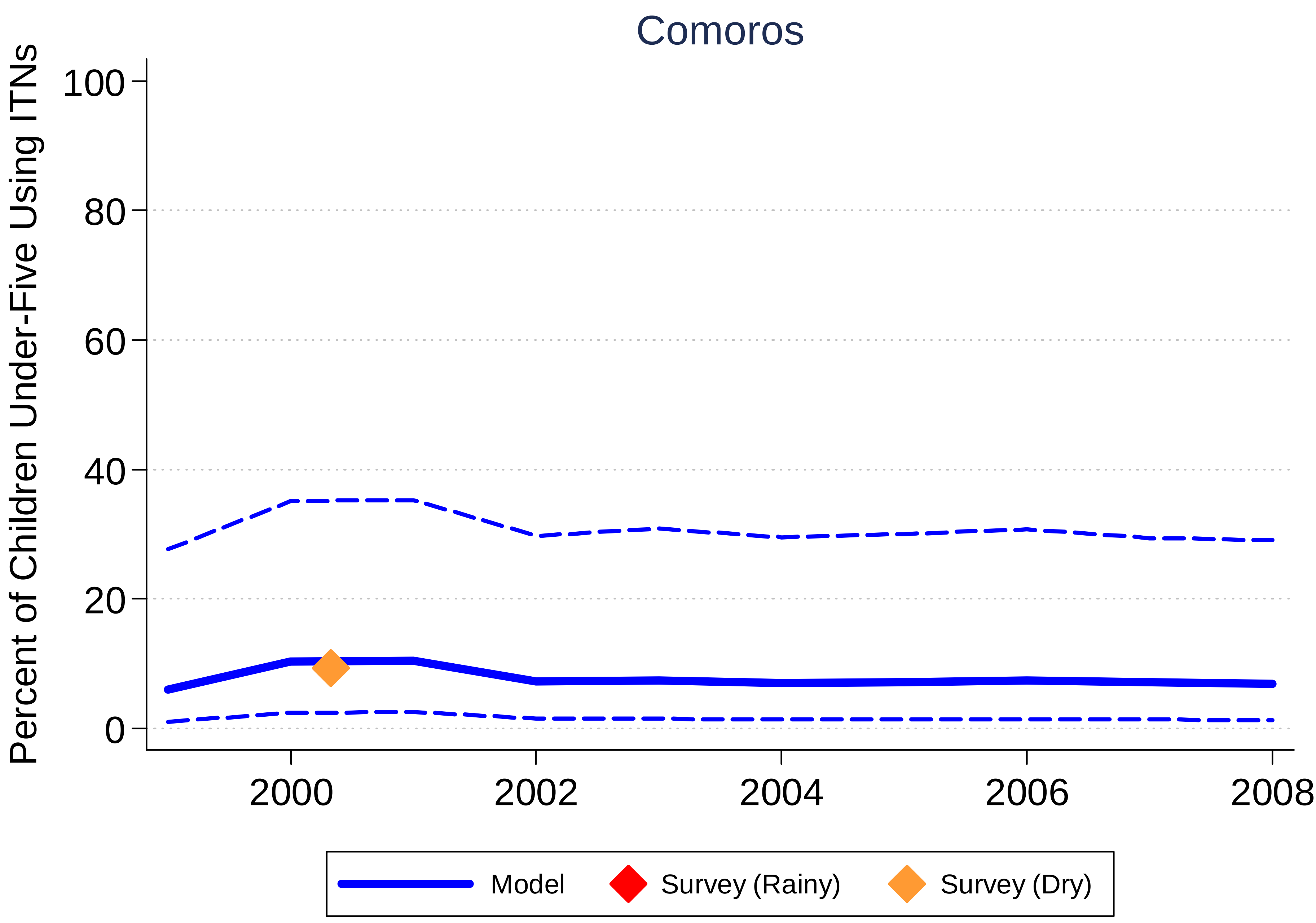

Comoros
Percent of Children Under-Five Using ITNs
0
20
40
60
80
100
2000
2002
2004
2006
2008
Model
Survey (Rainy)
Survey (Dry)

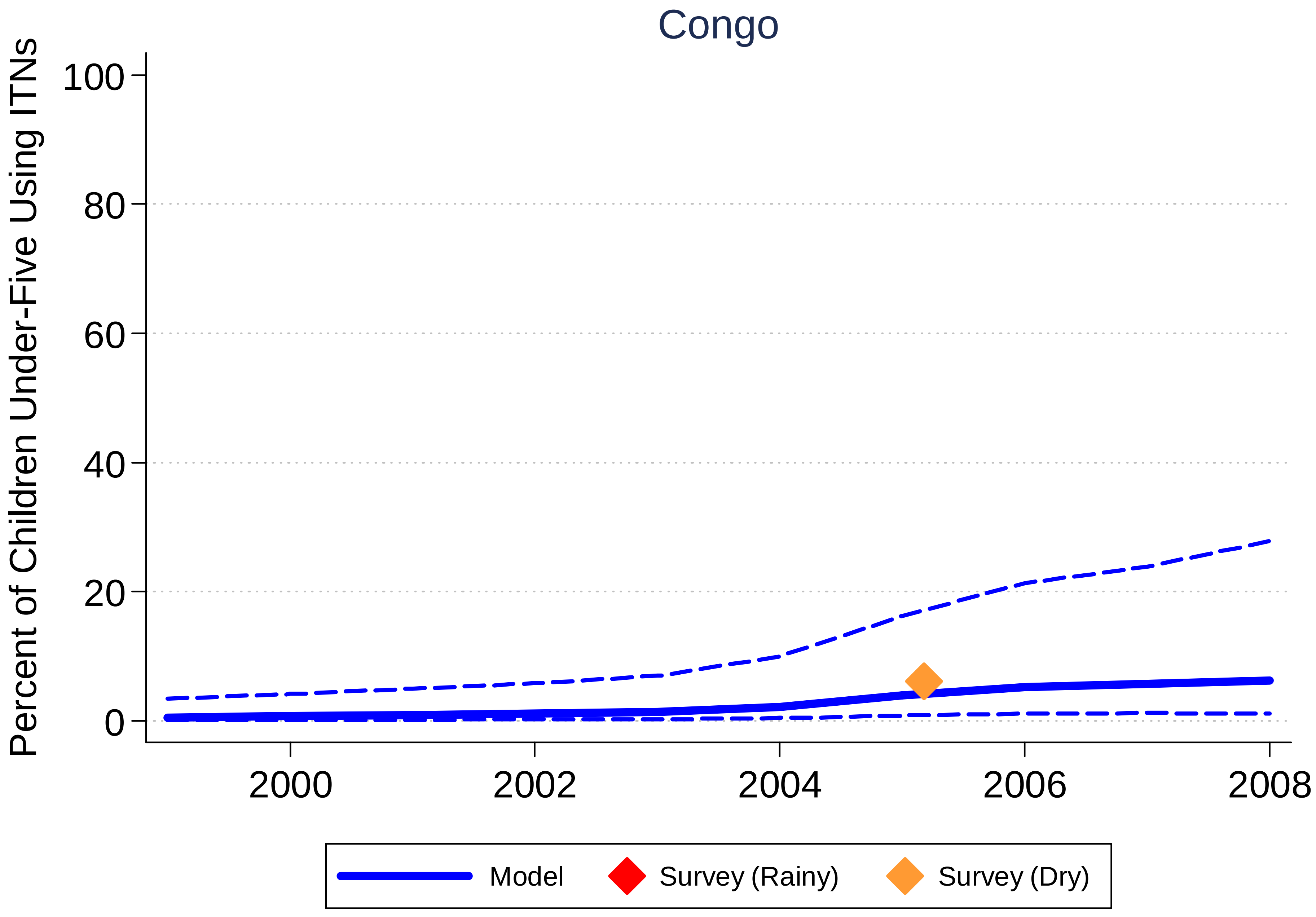

Congo
Percent of Children Under-Five Using ITNs
100
80
60
40
20
0
2000
2002
2004
2006
2008
Model
Survey (Rainy)
Survey (Dry)

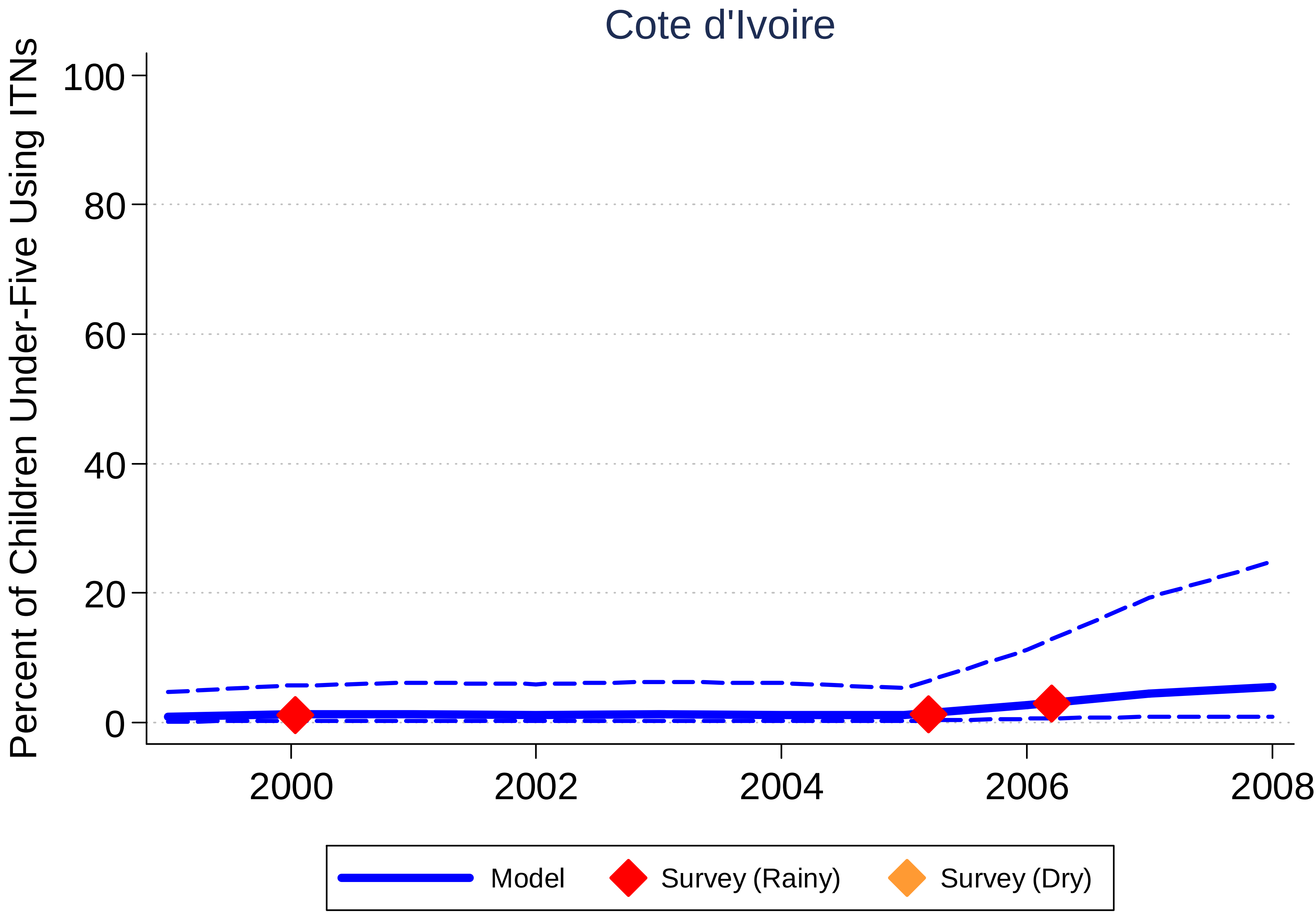

Cote d'Ivoire
Percent of Children Under-Five Using ITNs
0
20
40
60
80
100
2000
2002
2004
2006
2008
Model
Survey (Rainy)
Survey (Dry)

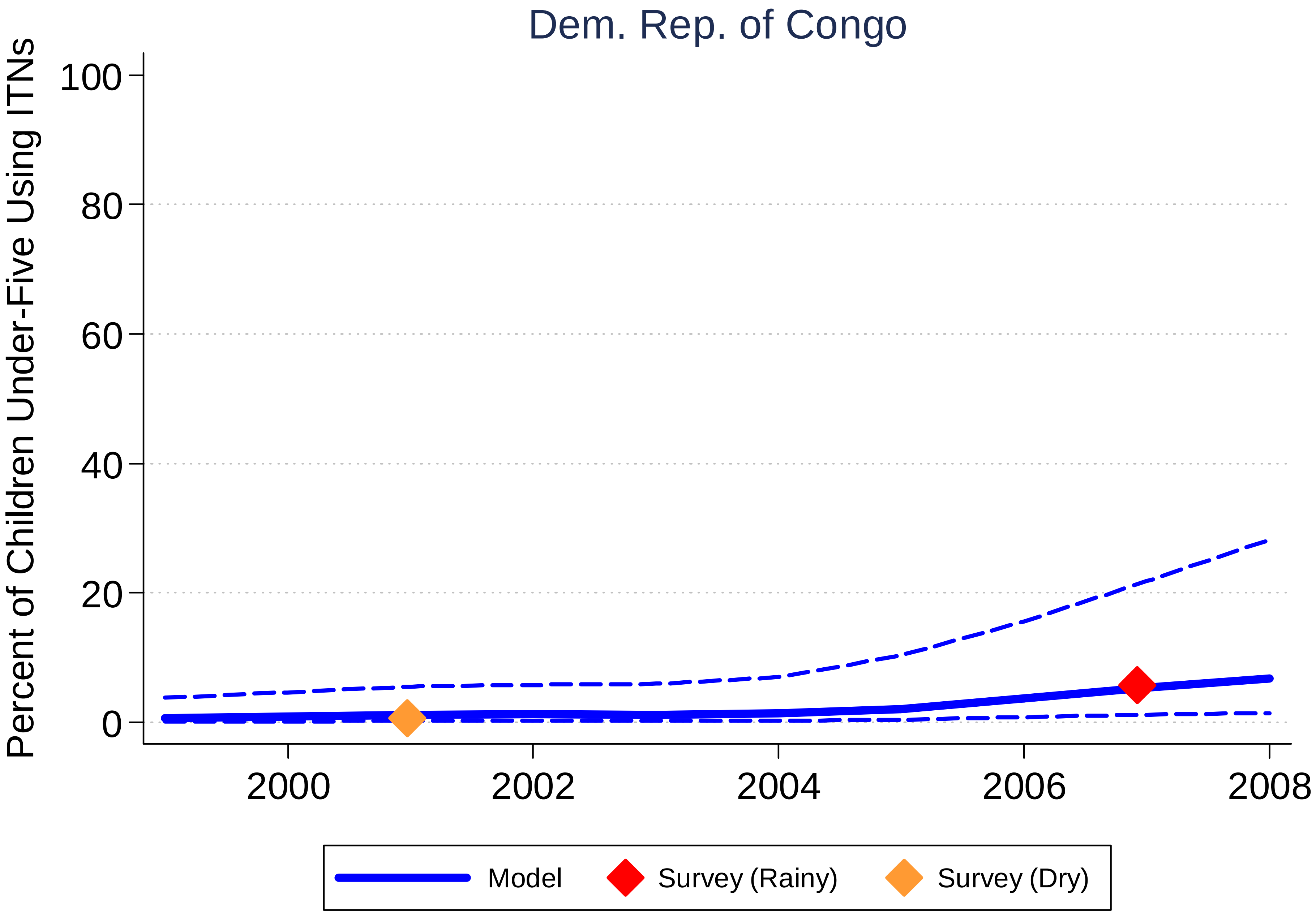

Dem. Rep. of Congo
Percent of Children Under-Five Using ITNs
100
80
60
40
20
0
2000
2002
2004
2006
2008
Model
Survey (Rainy)
Survey (Dry)

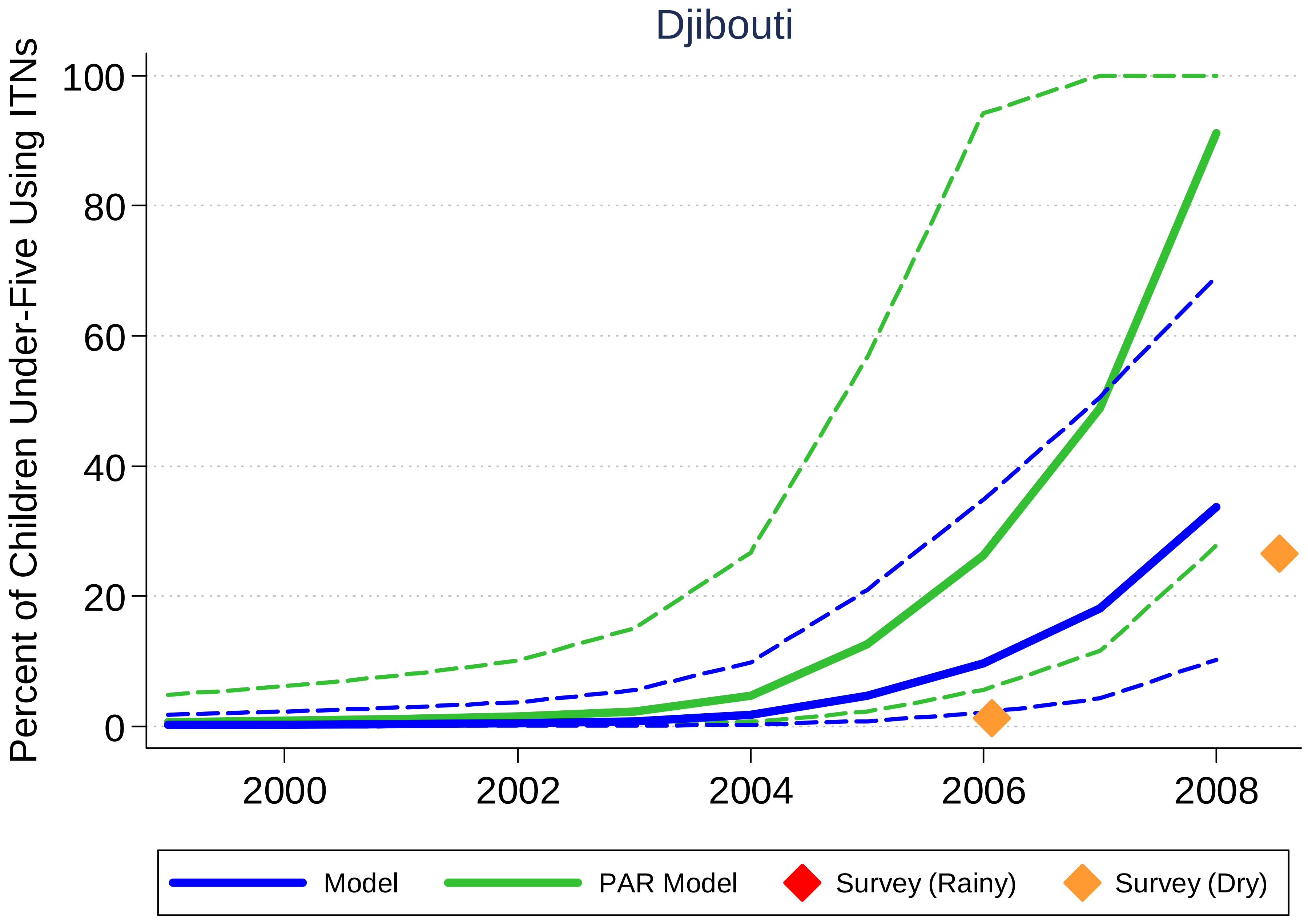
Djibouti
Percent of Children Under-Five Using ITNs
100
80
60
40
20
0
2000
2002
2004
2006
2008
Model
PAR Model
Survey (Rainy)
Survey (Dry)

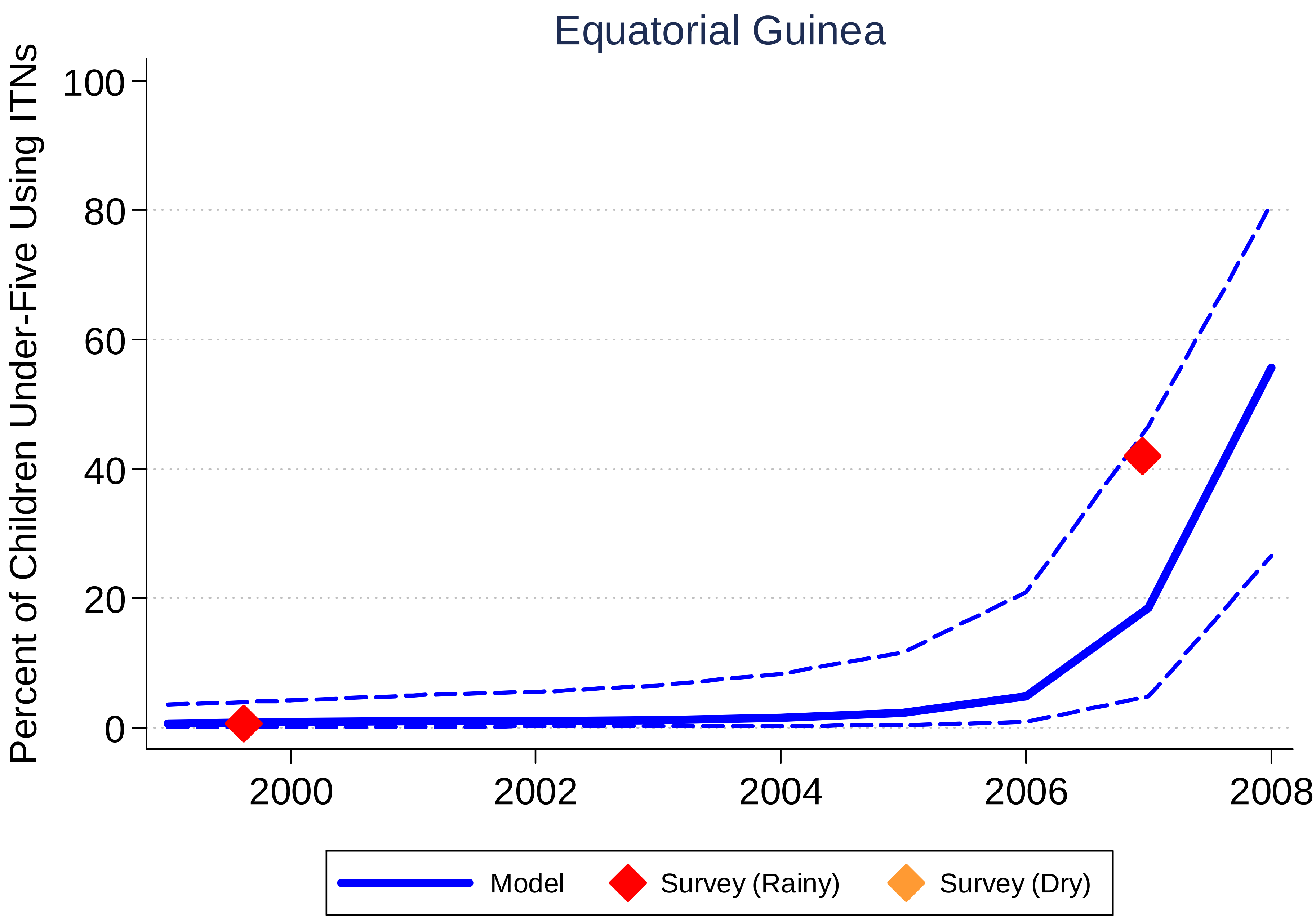

Equatorial Guinea
Percent of Children Under-Five Using ITNs
100
80
60
40
20
0
2000
2002
2004
2006
2008
Model
Survey (Rainy)
Survey (Dry)

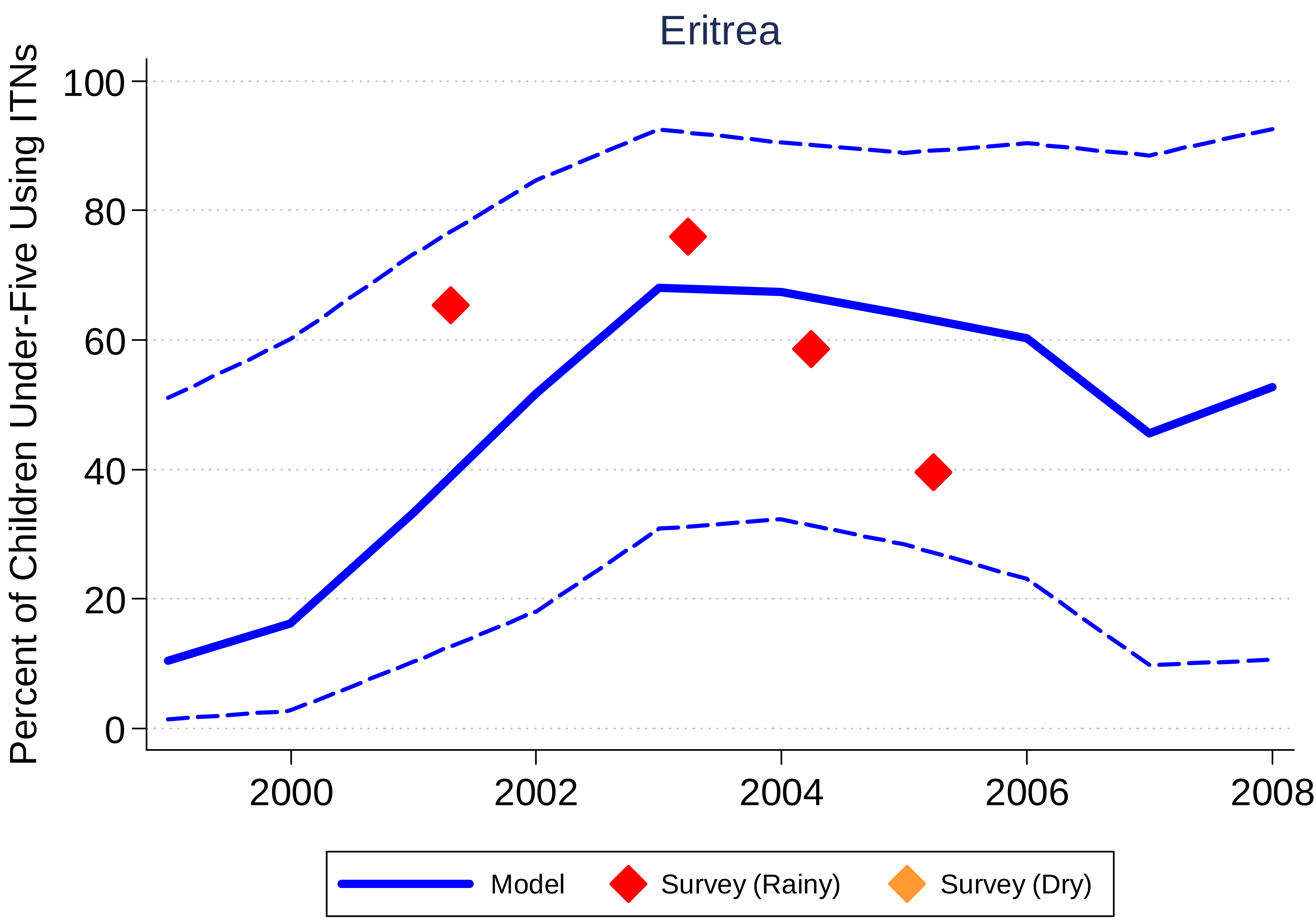
Eritrea
Percent of Children Under-Five Using ITNs
0
20
40
60
80
100
2000
2002
2004
2006
2008
Model
Survey (Rainy)
Survey (Dry)

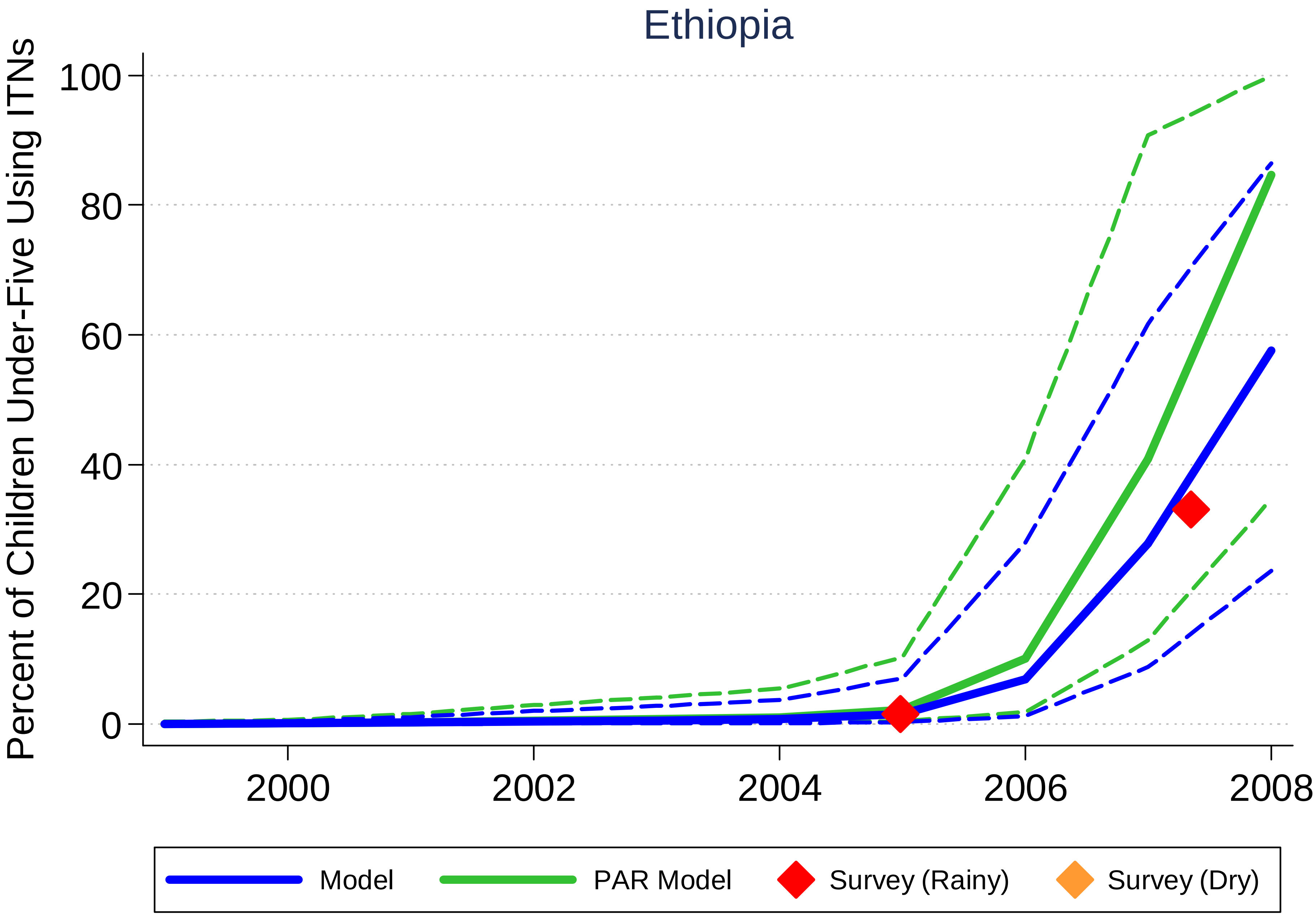
Ethiopia
Percent of Children Under-Five Using ITNs
0
20
40
60
80
100
2000
2002
2004
2006
2008
Model
PAR Model
Survey (Rainy)
Survey (Dry)

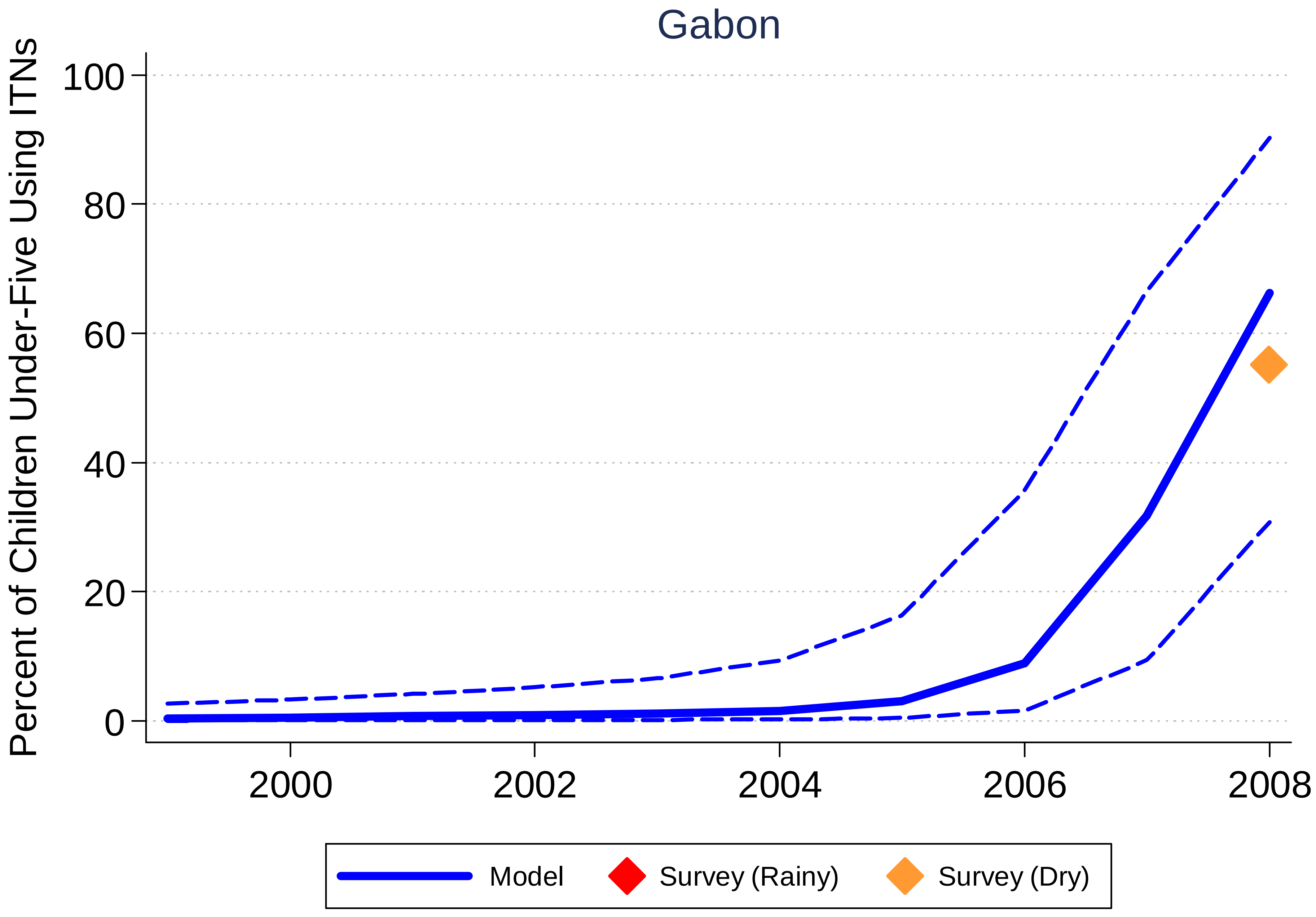
Gabon
Percent of Children Under-Five Using ITNs
100
80
60
40
20
0
2000
2002
2004
2006
2008
Model
Survey (Rainy)
Survey (Dry)

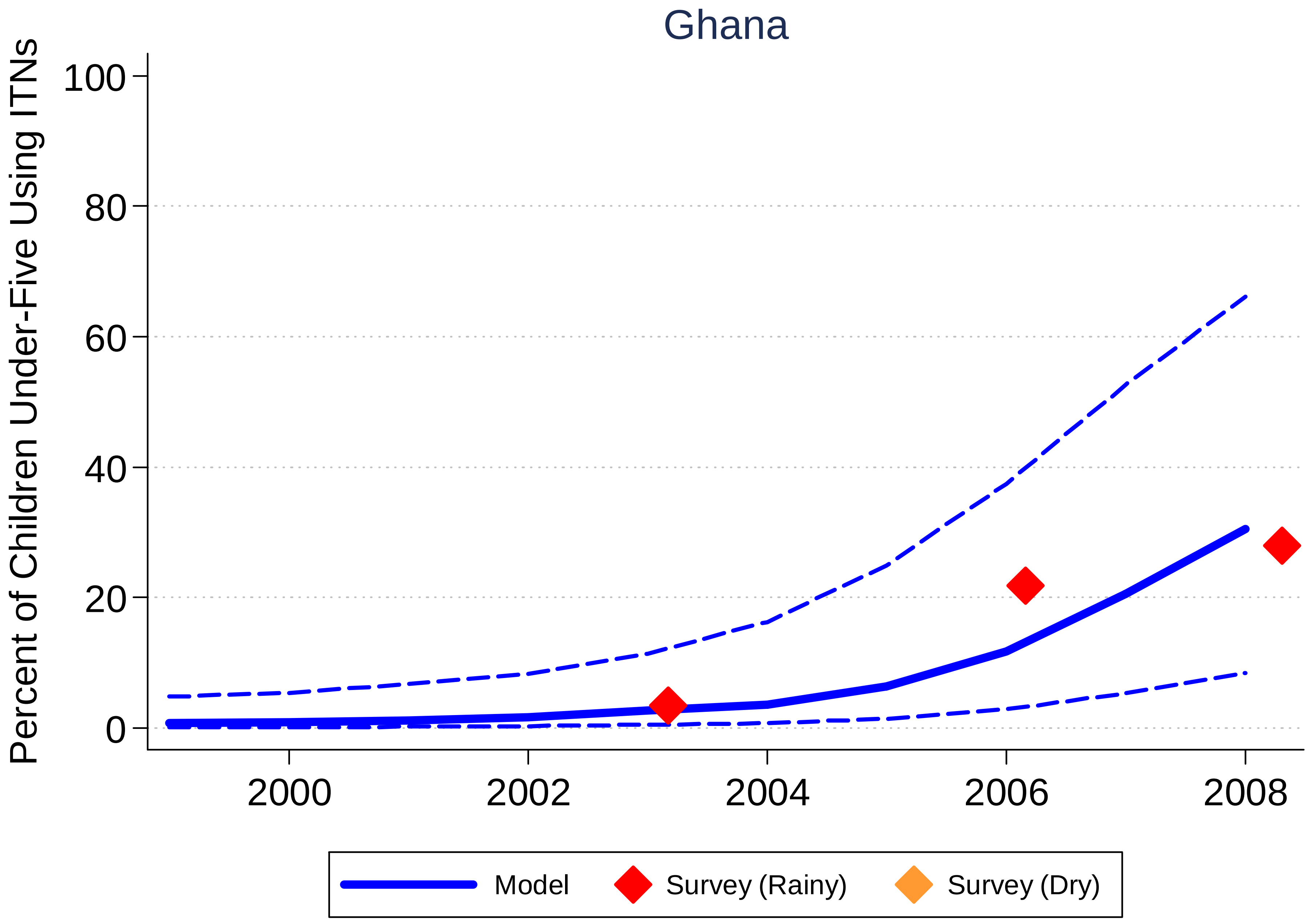

Ghana
Percent of Children Under-Five Using ITNs
0
20
40
60
80
100
2000
2002
2004
2006
2008
Model
Survey (Rainy)
Survey (Dry)

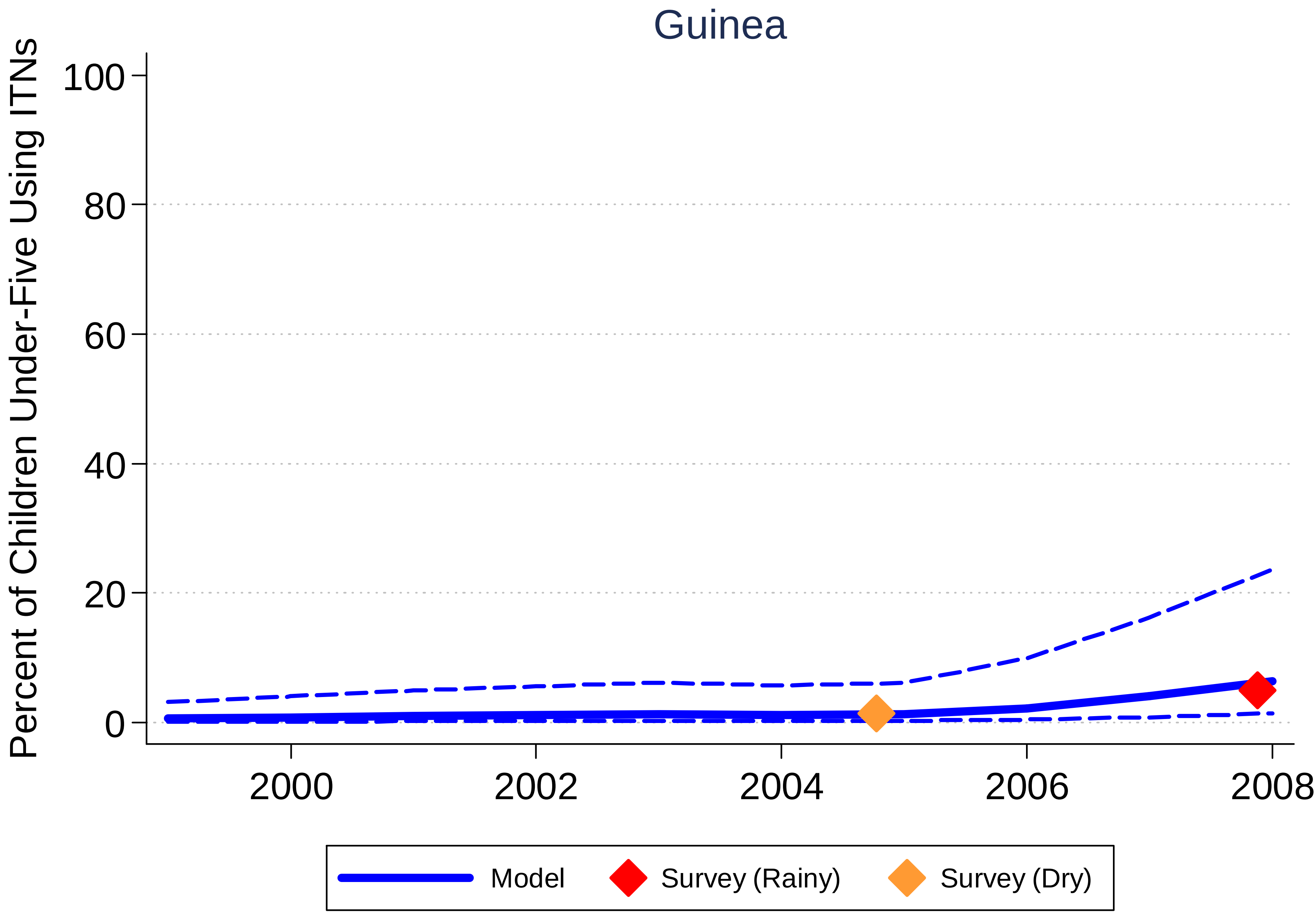

Guinea
Percent of Children Under-Five Using ITNs
100
80
60
40
20
0
2000
2002
2004
2006
2008
Model
Survey (Rainy)
Survey (Dry)

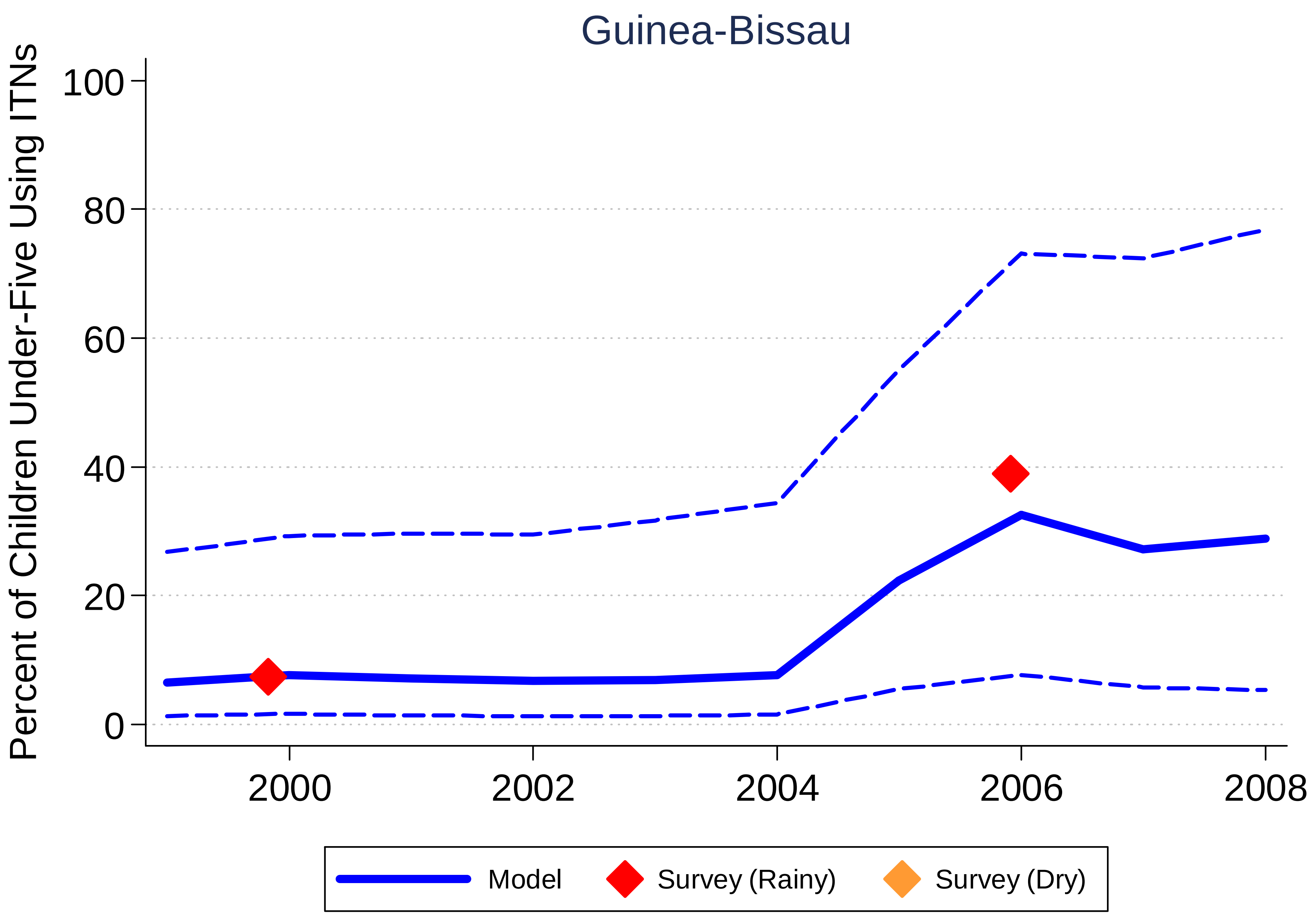

Guinea-Bissau
Percent of Children Under-Five Using ITNs
0
20
40
60
80
100
2000
2002
2004
2006
2008
Model
Survey (Rainy)
Survey (Dry)

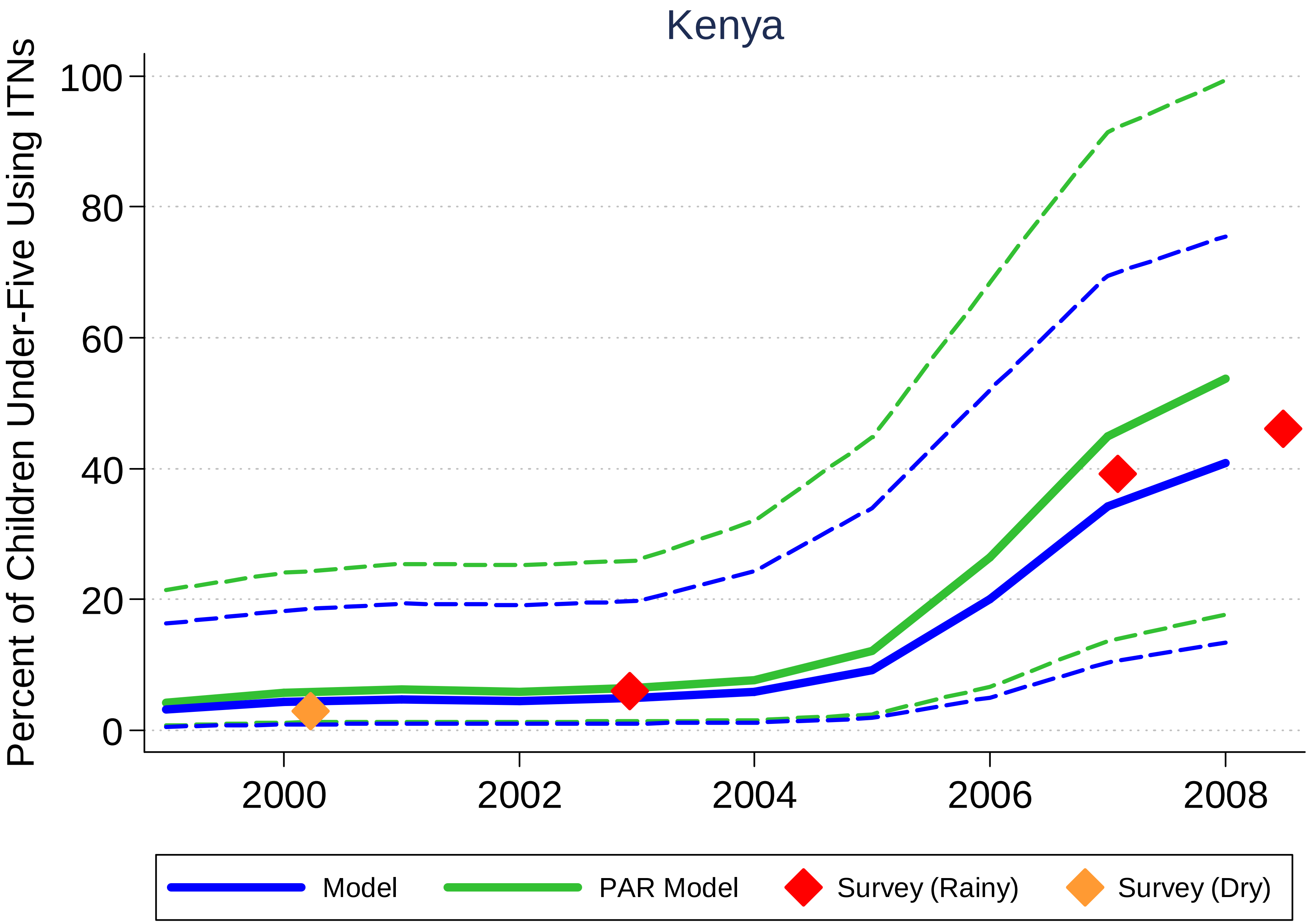
Kenya
Percent of Children Under-Five Using ITNs
0
20
40
60
80
100
2000
2002
2004
2006
2008
Model
PAR Model
Survey (Rainy)
Survey (Dry)

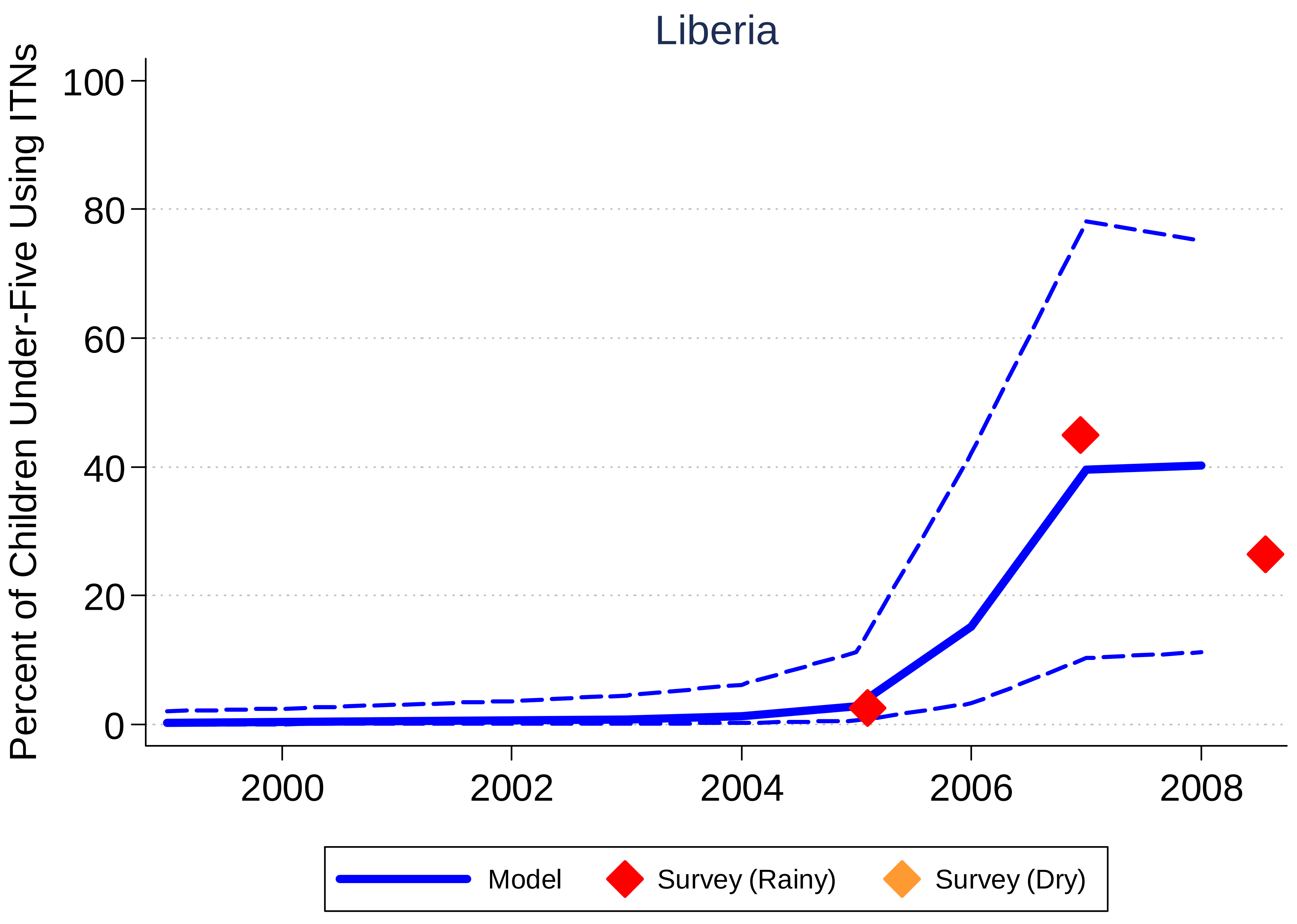
Liberia
Percent of Children Under-Five Using ITNs
100
80
60
40
20
0
2000
2002
2004
2006
2008
Model
Survey (Rainy)
Survey (Dry)

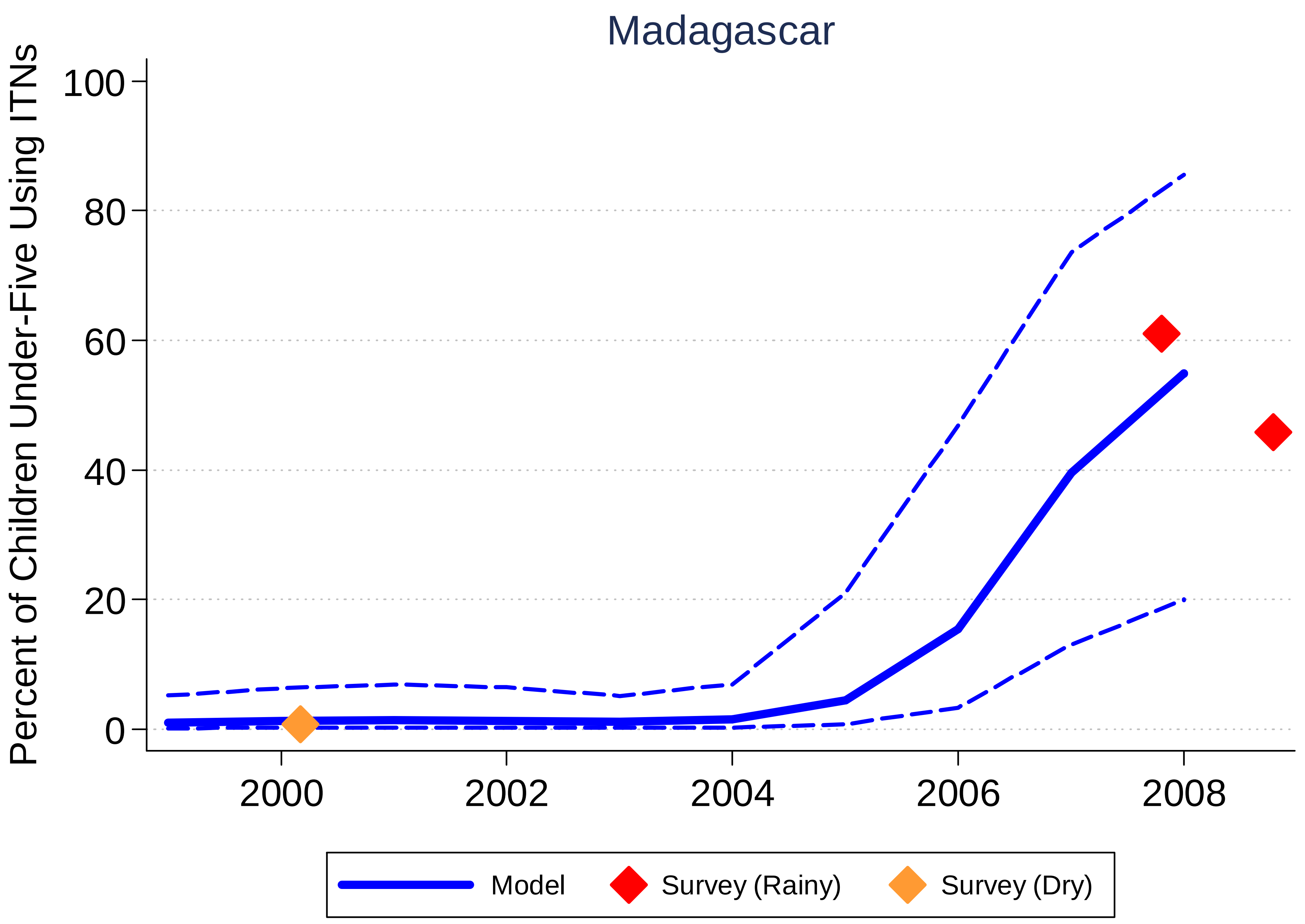

Madagascar
Percent of Children Under-Five Using ITNs
100
80
60
40
20
0
2000
2002
2004
2006
2008
Model
Survey (Rainy)
Survey (Dry)

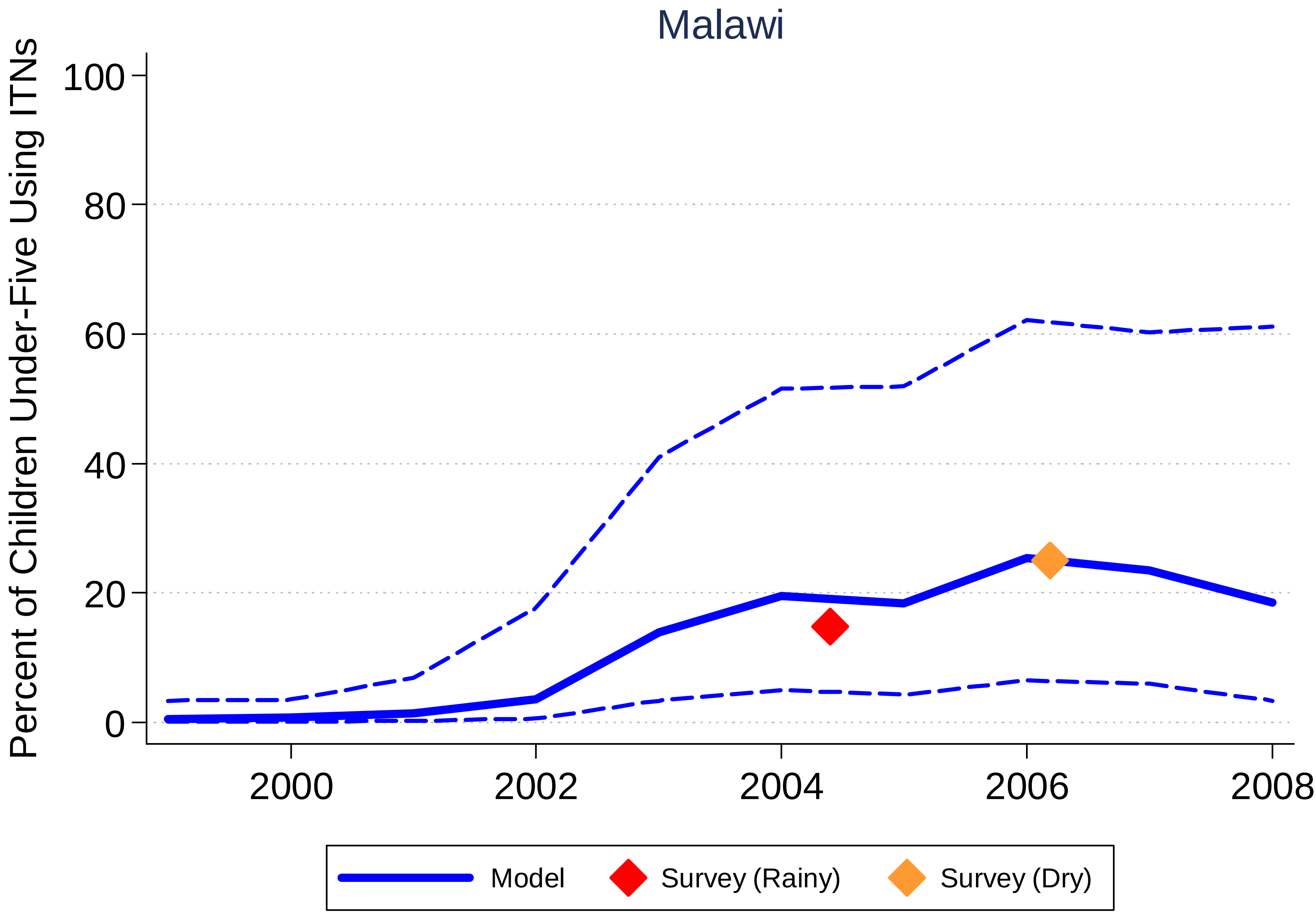
Malawi
Percent of Children Under-Five Using ITNs
100
80
60
40
20
0
2000
2002
2004
2006
2008
Model
Survey (Rainy)
Survey (Dry)

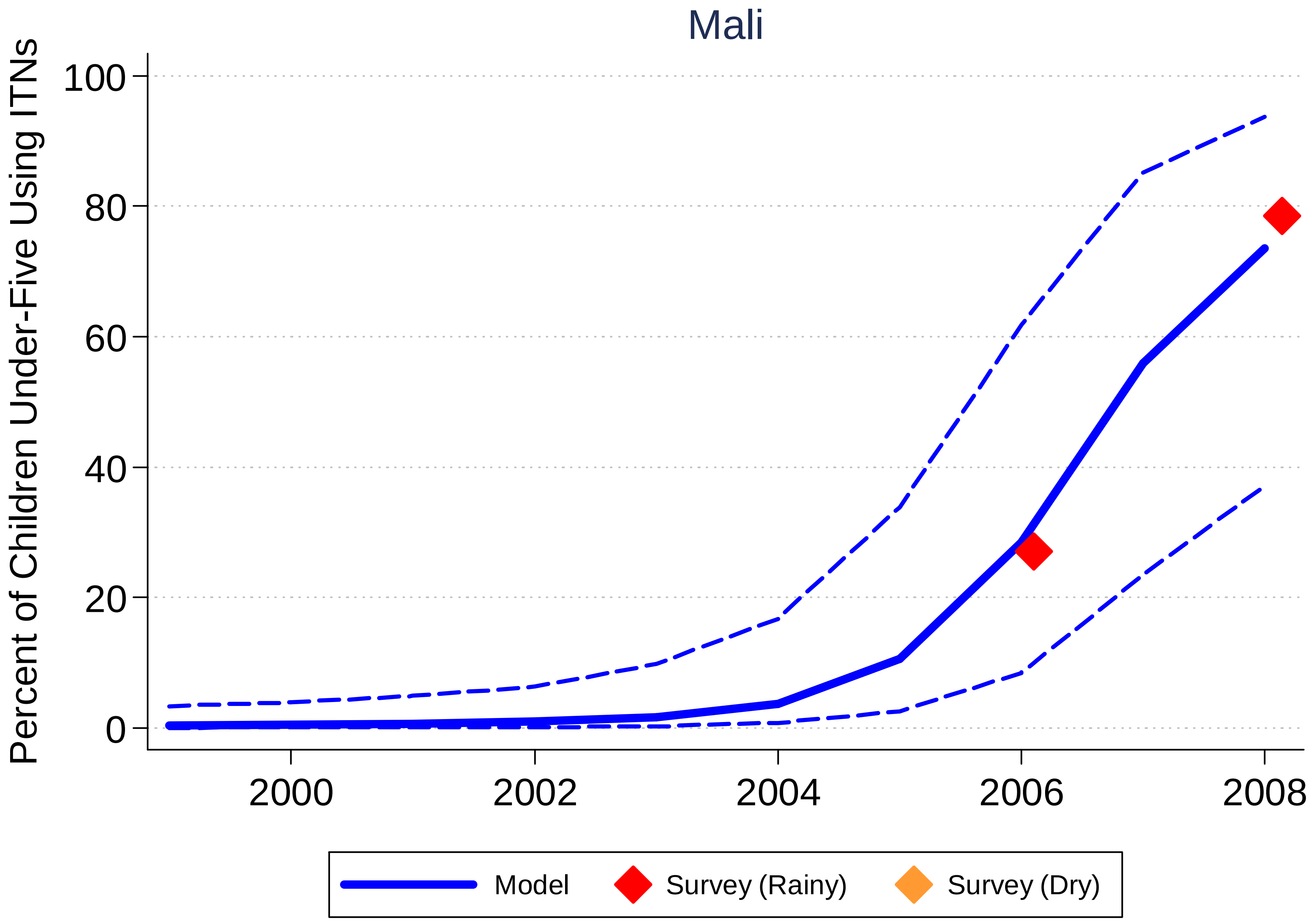

Mali
Percent of Children Under-Five Using ITNs
100
80
60
40
20
0
2000
2002
2004
2006
2008
Model
Survey (Rainy)
Survey (Dry)

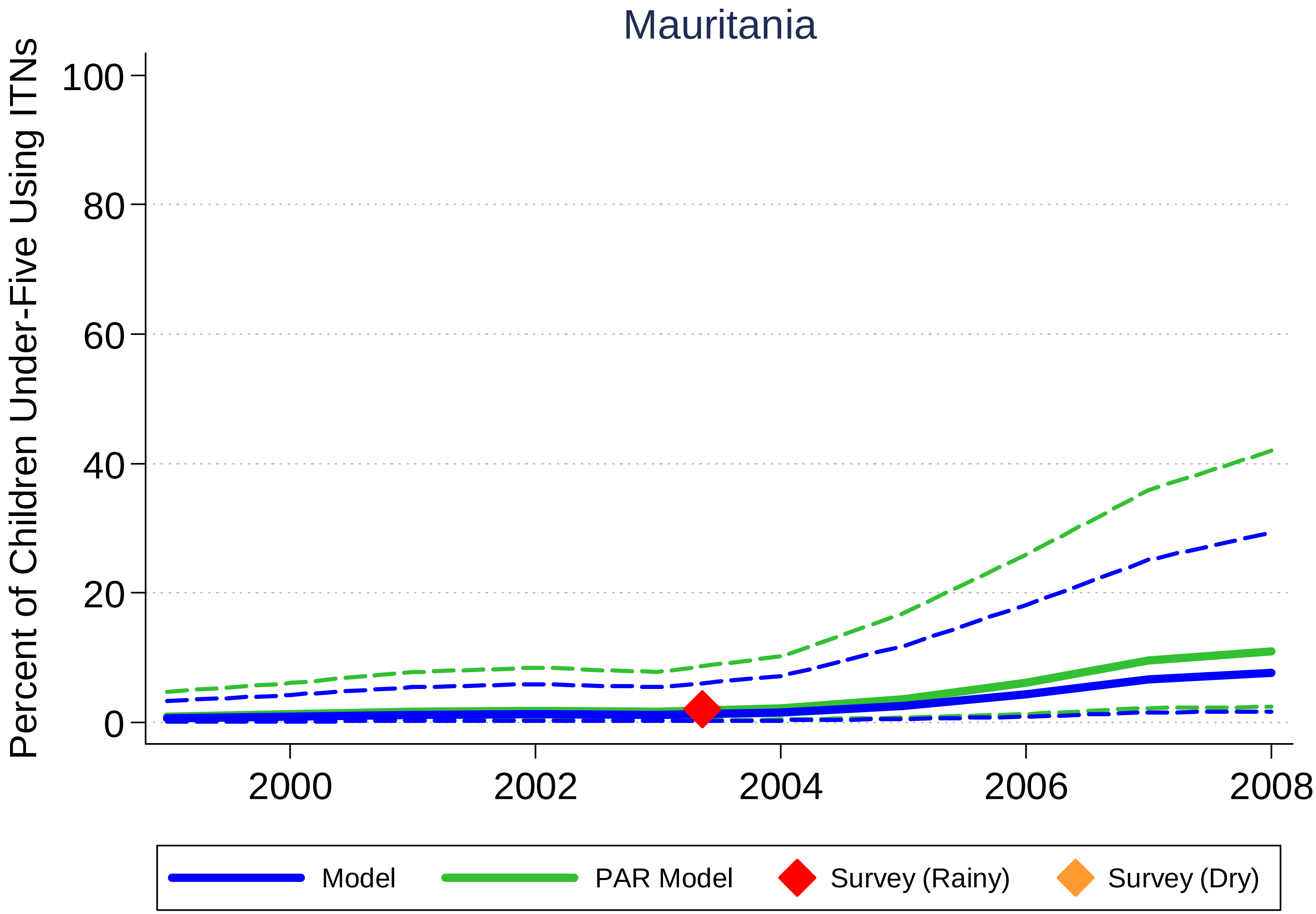

Mauritania
Percent of Children Under-Five Using ITNs
100
80
60
40
20
0
2000
2002
2004
2006
2008
Model
PAR Model
Survey (Rainy)
Survey (Dry)

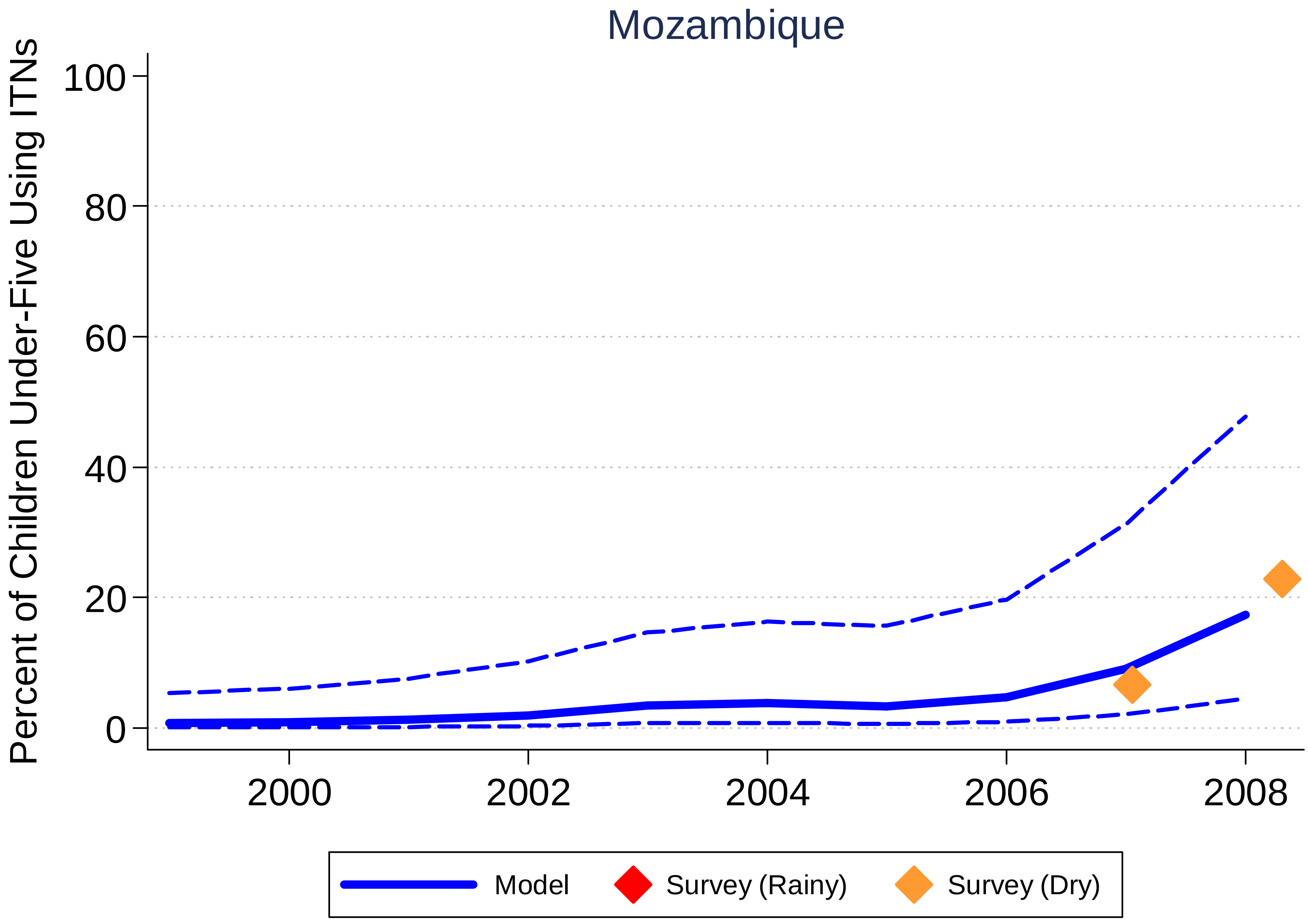

Mozambique
Percent of Children Under-Five Using ITNs
0
20
40
60
80
100
2000
2002
2004
2006
2008
Model
Survey (Rainy)
Survey (Dry)

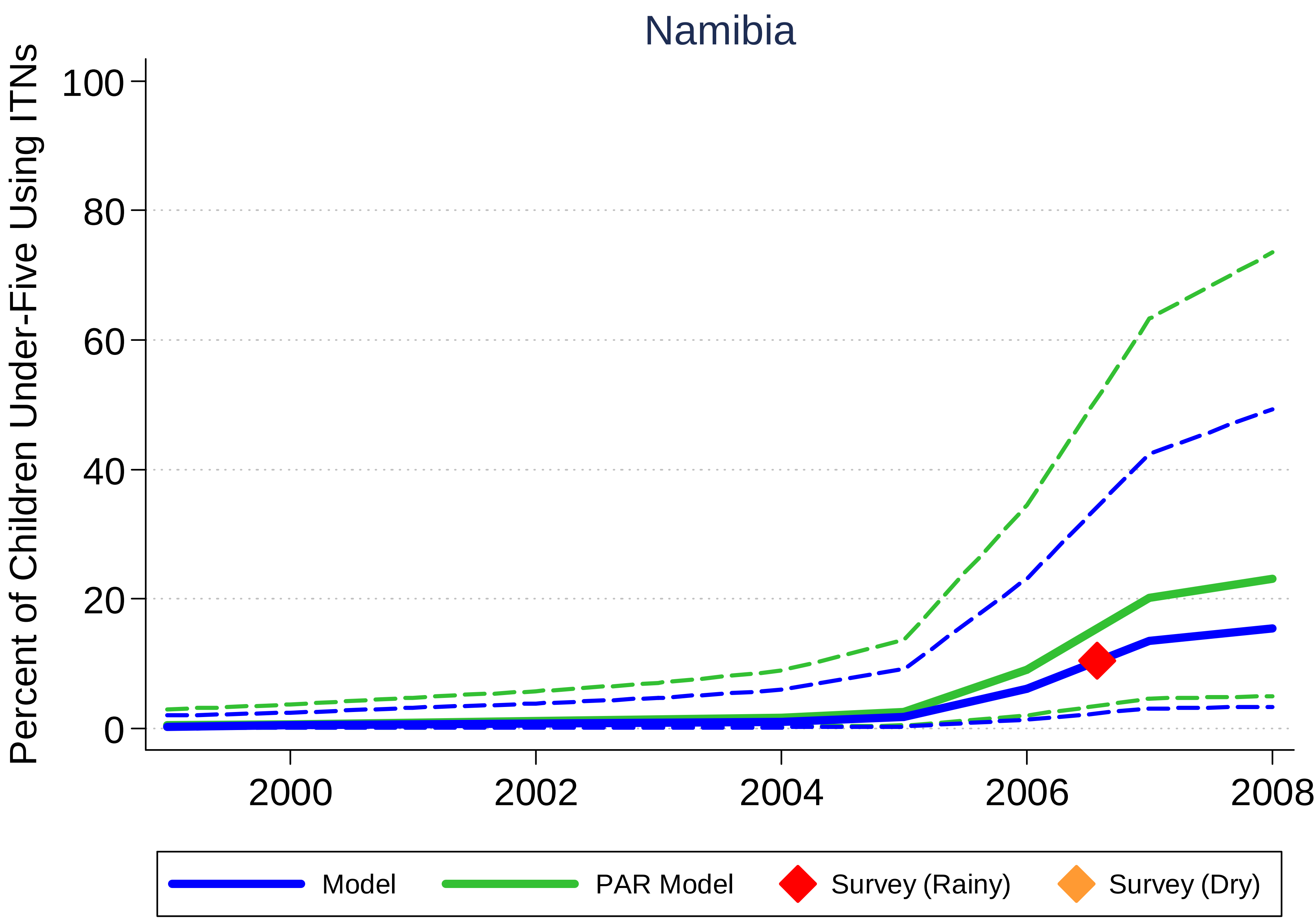

Namibia
Percent of Children Under-Five Using ITNs
0
20
40
60
80
100
2000
2002
2004
2006
2008
Model
PAR Model
Survey (Rainy)
Survey (Dry)

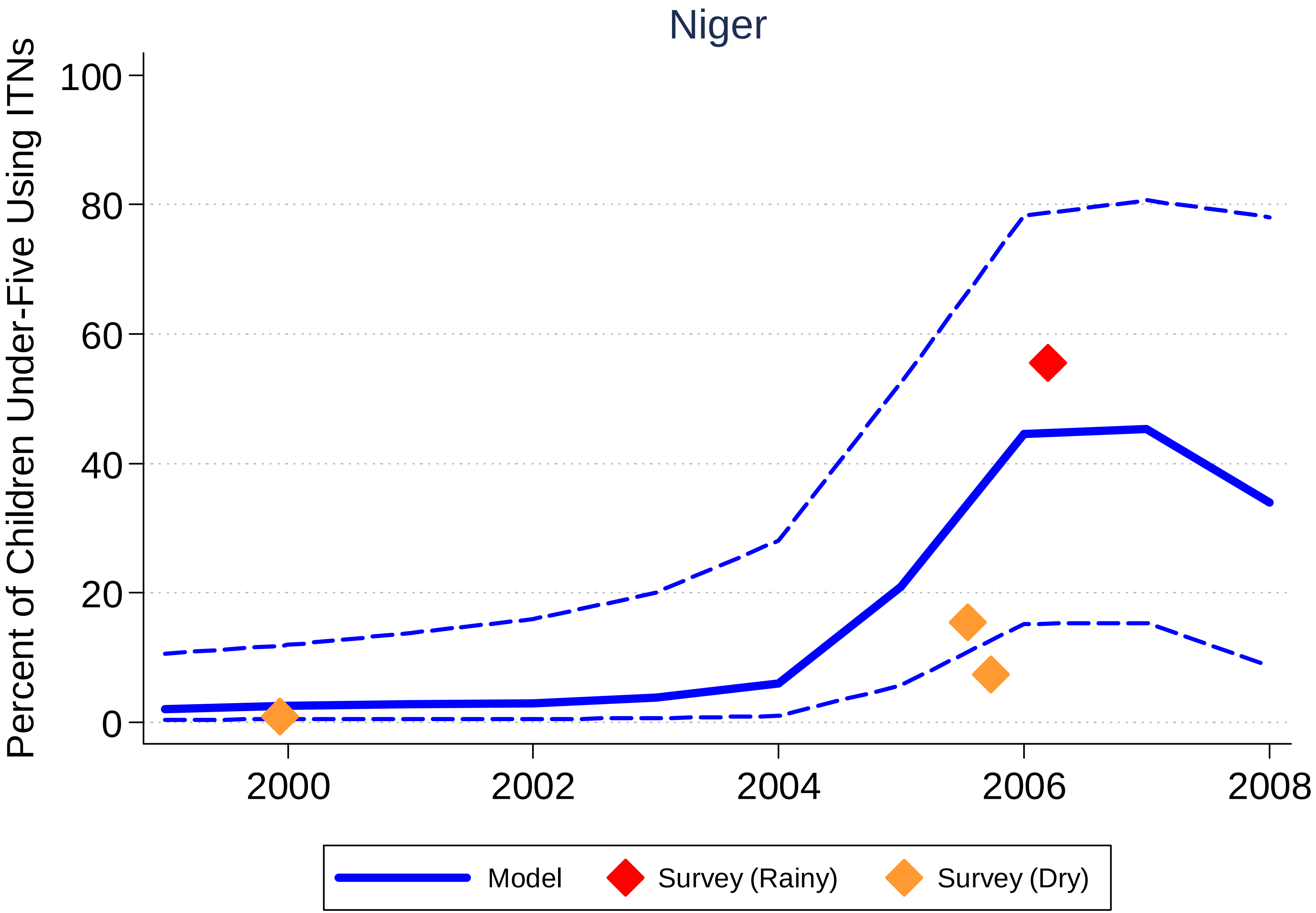

Niger
Percent of Children Under-Five Using ITNs
0
20
40
60
80
100
2000
2002
2004
2006
2008
Model
Survey (Rainy)
Survey (Dry)

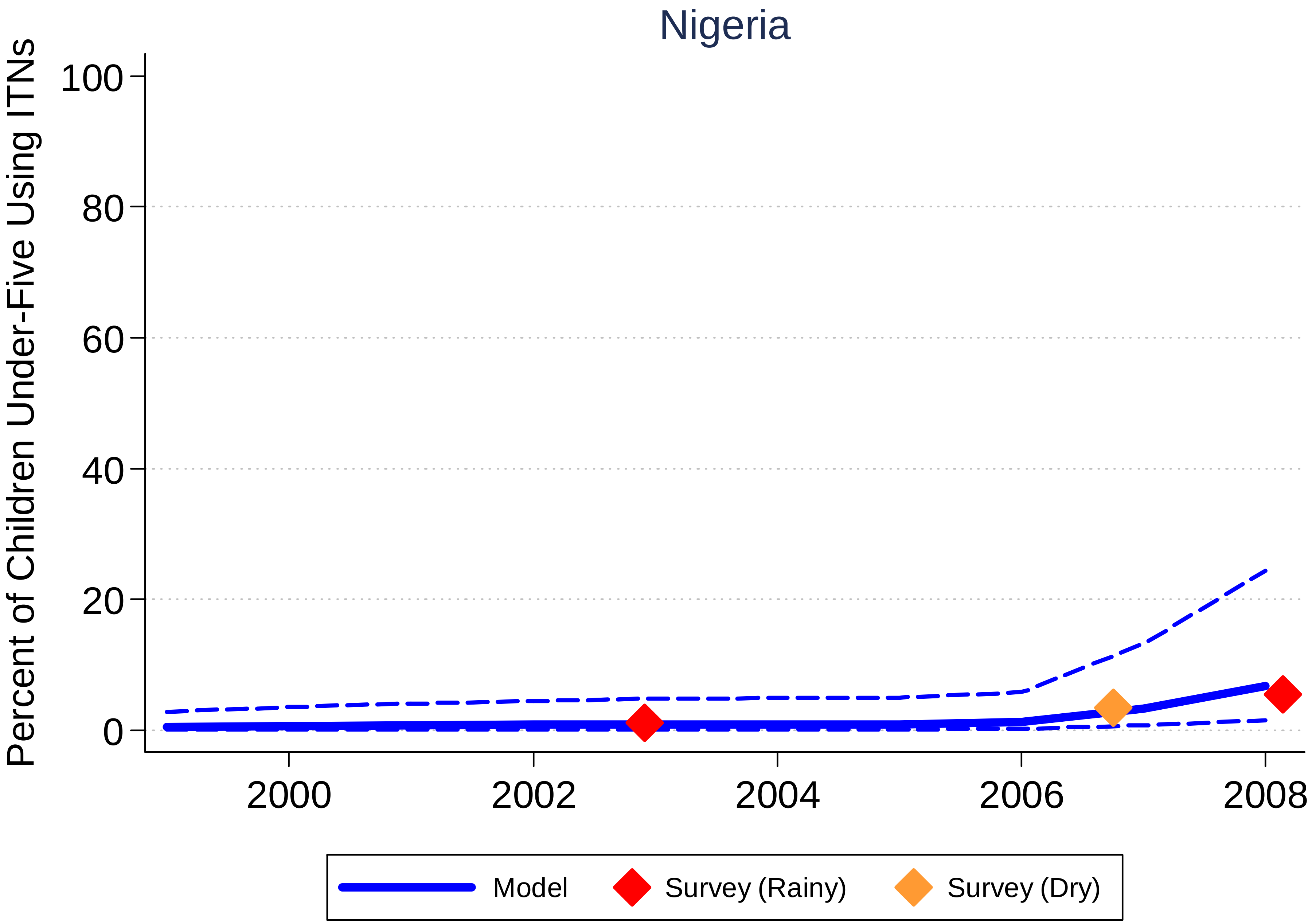
Nigeria
Percent of Children Under-Five Using ITNs
0
20
40
60
80
100
2000
2002
2004
2006
2008
Model
Survey (Rainy)
Survey (Dry)

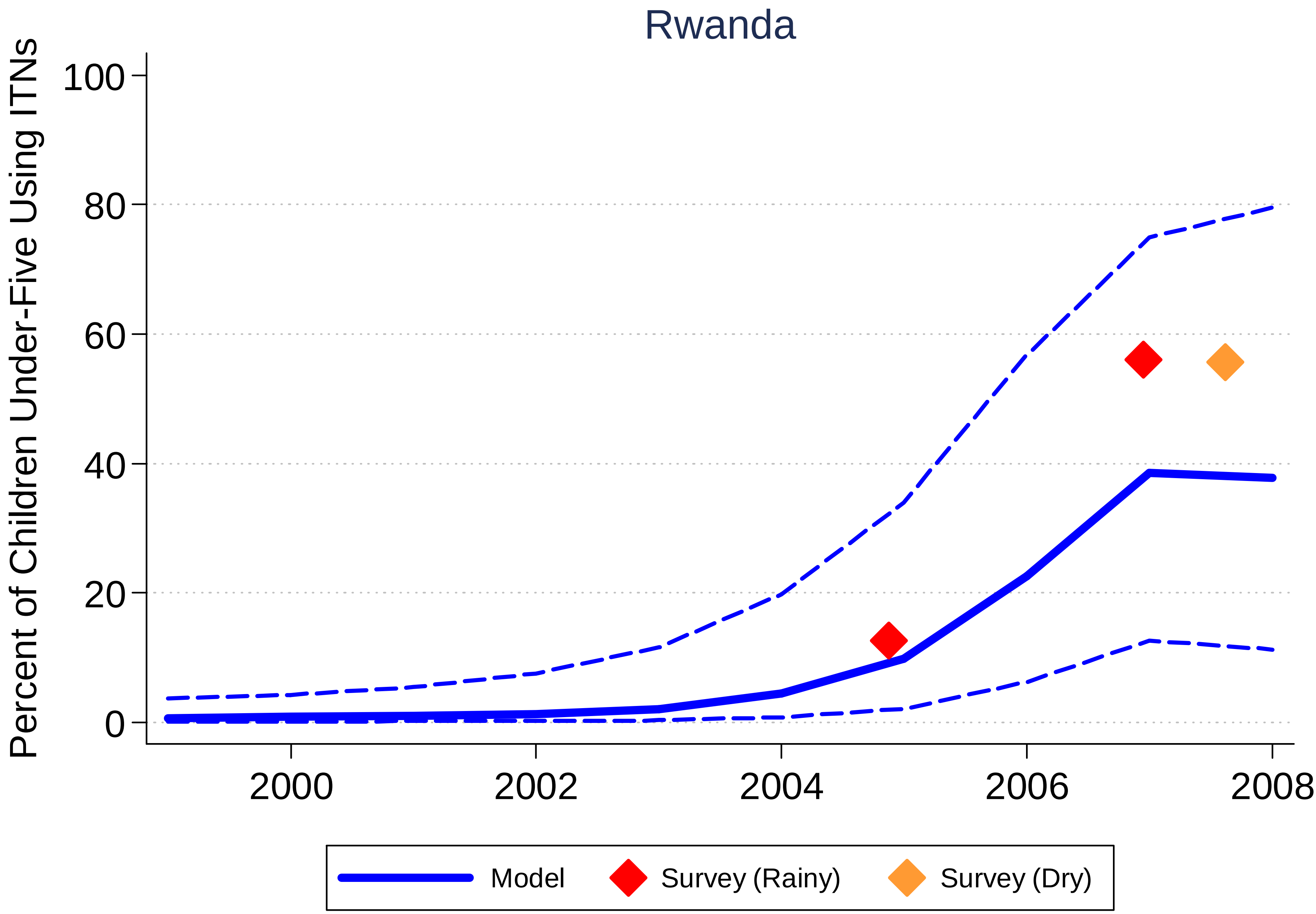
Rwanda
Percent of Children Under-Five Using ITNs
100
80
60
40
20
0
2000
2002
2004
2006
2008
Model
Survey (Rainy)
Survey (Dry)

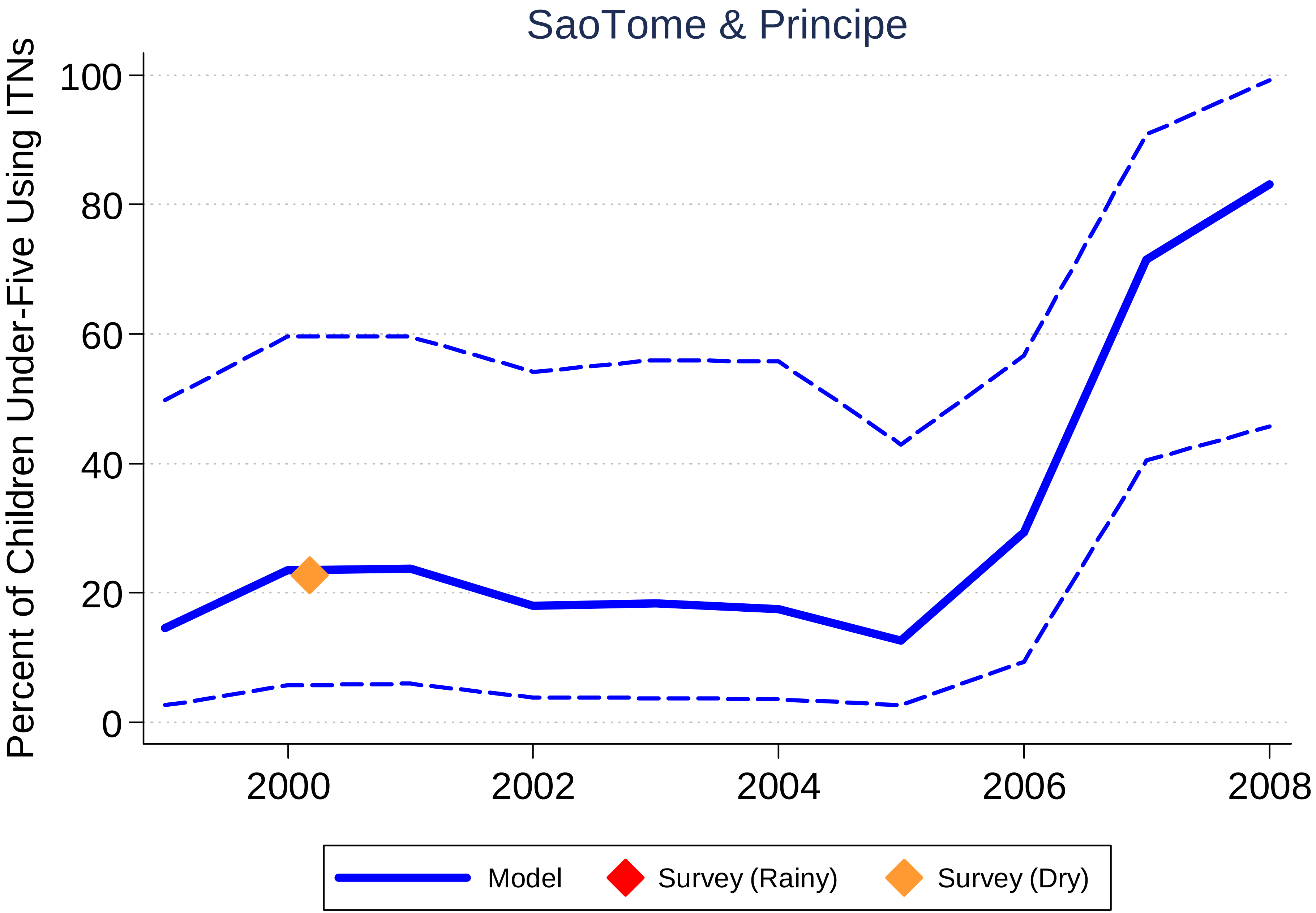

SaoTome & Principe
Percent of Children Under-Five Using ITNs
0
20
40
60
80
100
2000
2002
2004
2006
2008
Model
Survey (Rainy)
Survey (Dry)

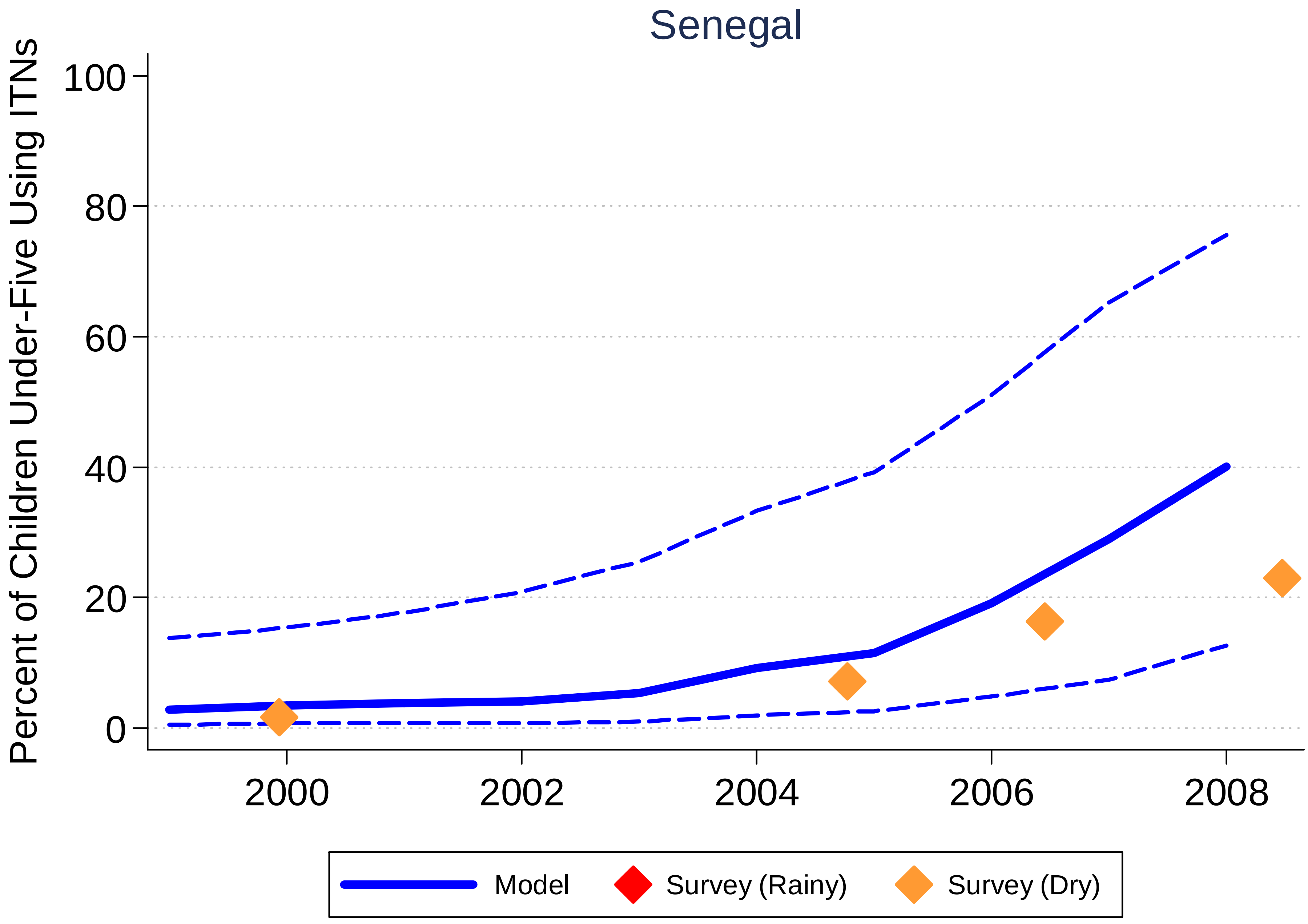
Senegal
Percent of Children Under-Five Using ITNs
100
80
60
40
20
0
2000
2002
2004
2006
2008
Model
Survey (Rainy)
Survey (Dry)

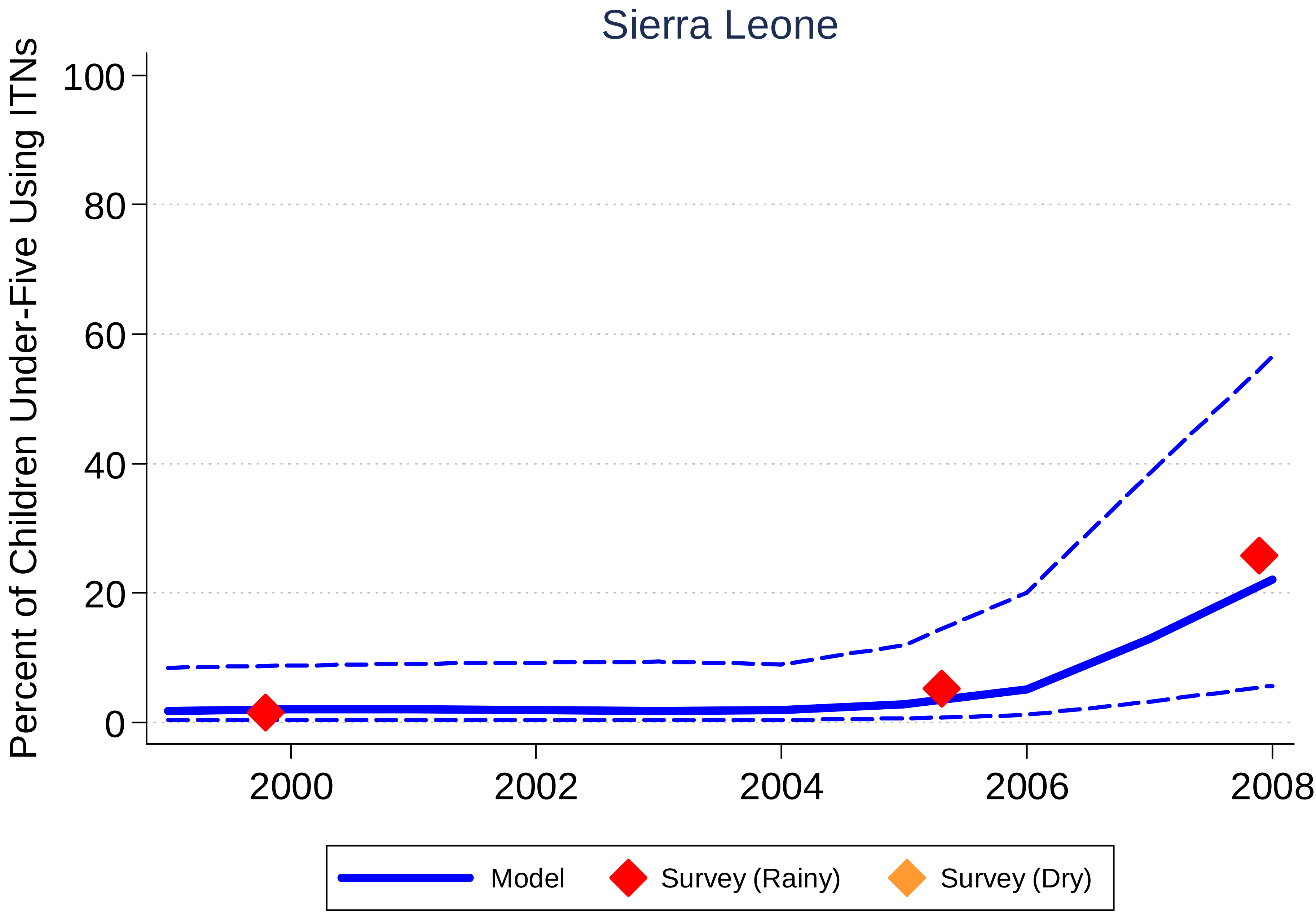
Sierra Leone
Percent of Children Under-Five Using ITNs
0
20
40
60
80
100
2000
2002
2004
2006
2008
Model
Survey (Rainy)
Survey (Dry)

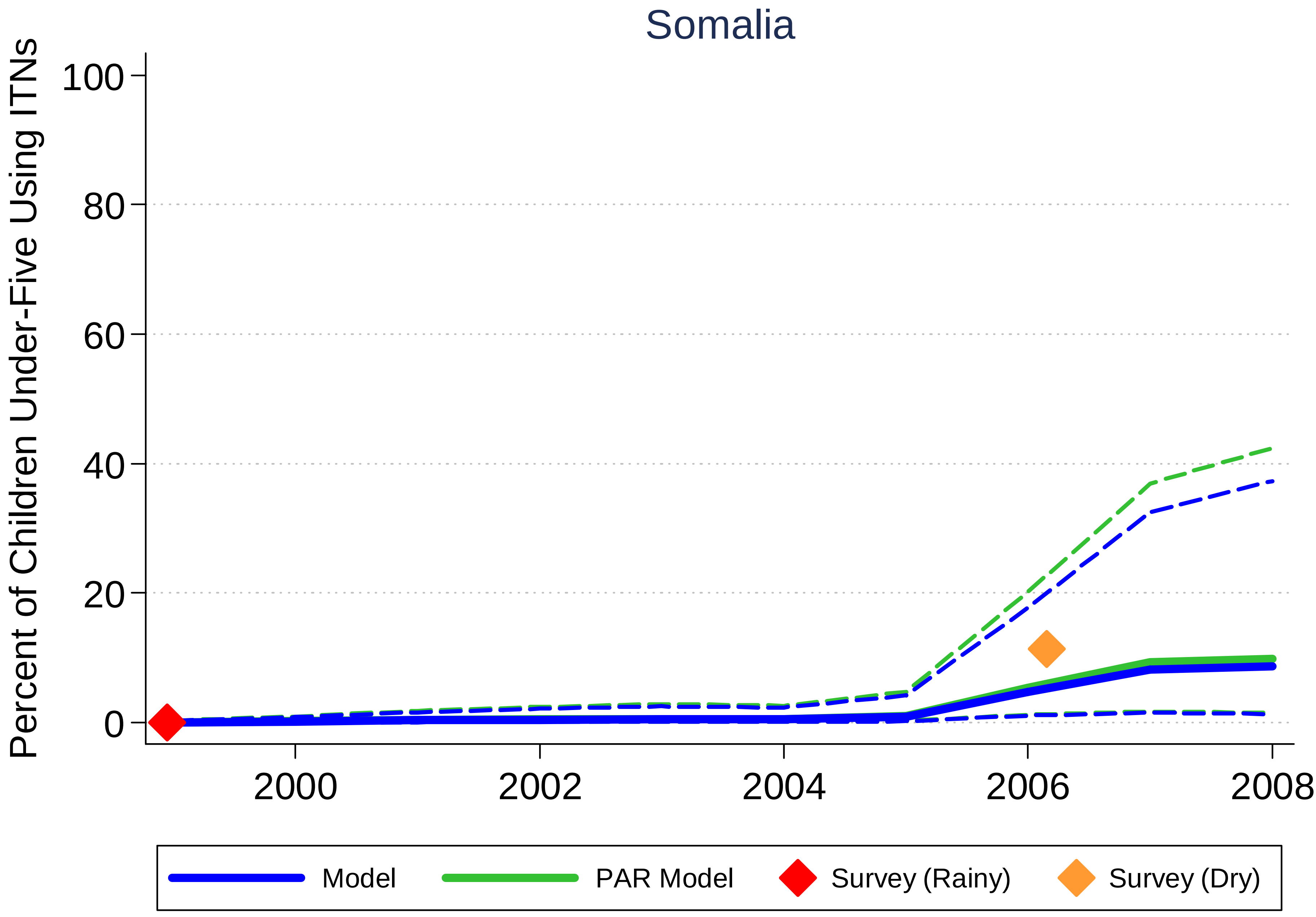
Somalia
Percent of Children Under-Five Using ITNs
100
80
60
40
20
0
2000
2002
2004
2006
2008
Model
PAR Model
Survey (Rainy)
Survey (Dry)

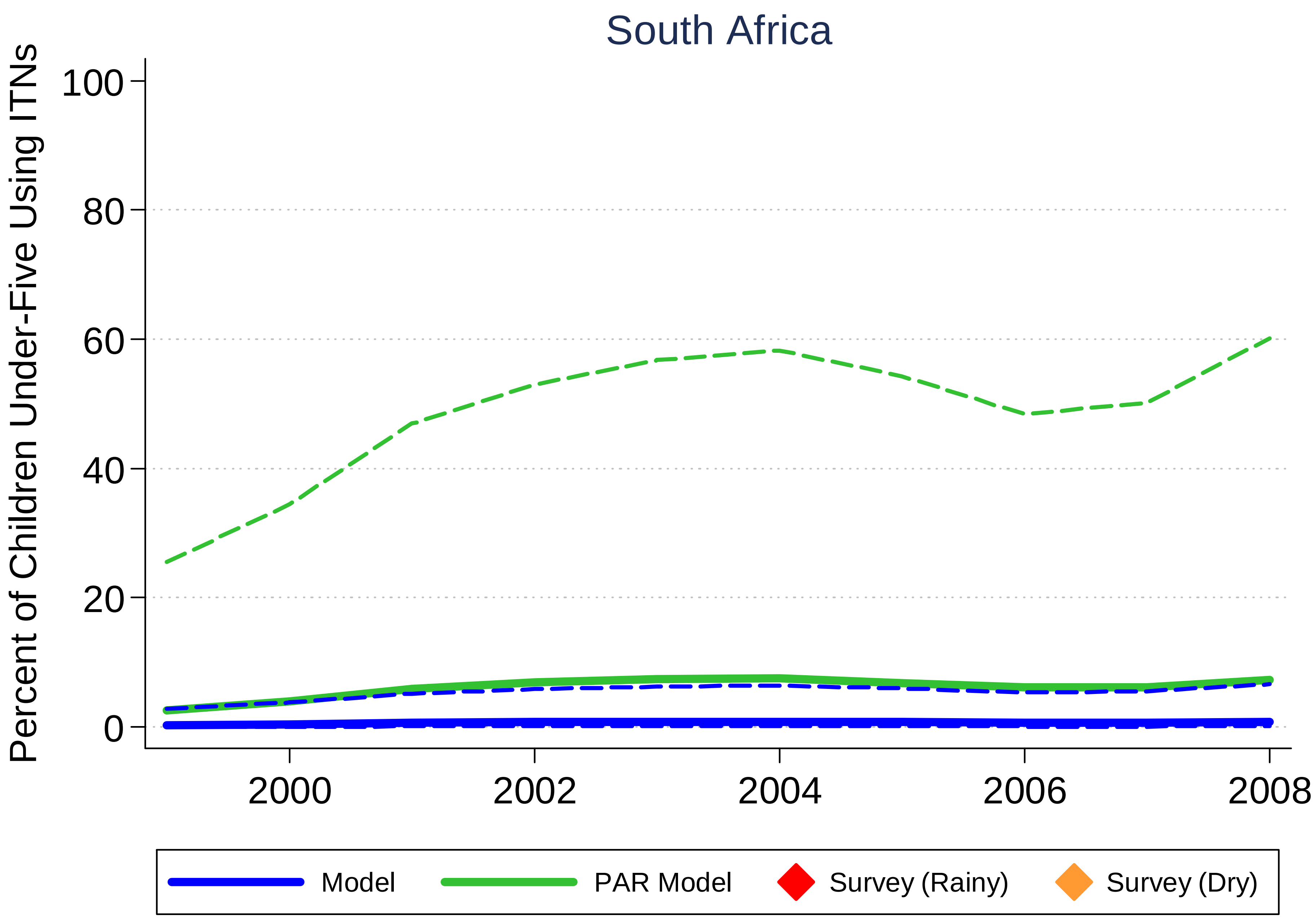
South Africa
Percent of Children Under-Five Using ITNs
0
20
40
60
80
100
2000
2002
2004
2006
2008
Model
PAR Model
Survey (Rainy)
Survey (Dry)

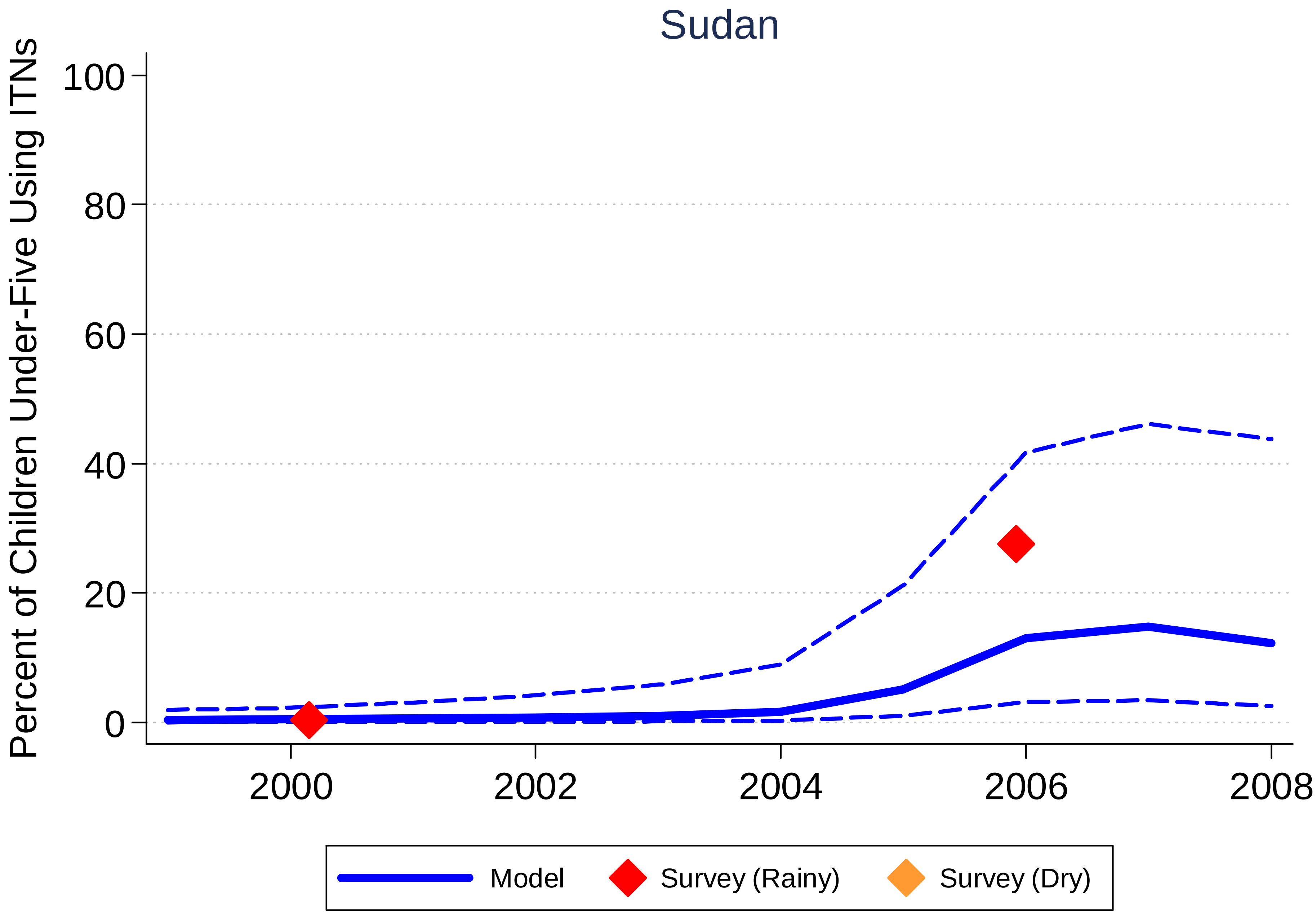

Sudan
Percent of Children Under-Five Using ITNs
100
80
60
40
20
0
2000
2002
2004
2006
2008
Model
Survey (Rainy)
Survey (Dry)

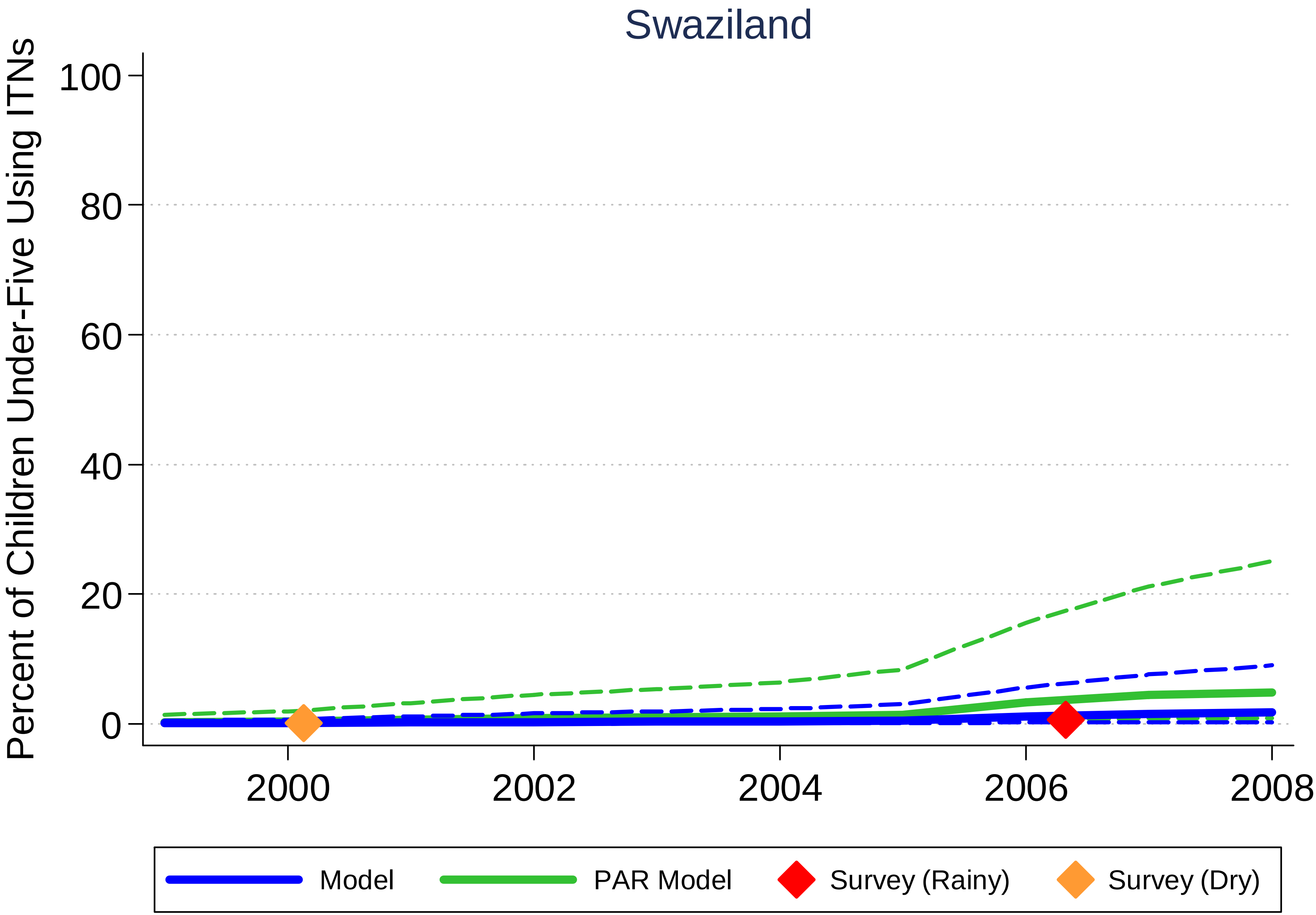
Swaziland
Percent of Children Under-Five Using ITNs
100
80
60
40
20
0
2000
2002
2004
2006
2008
Model
PAR Model
Survey (Rainy)
Survey (Dry)

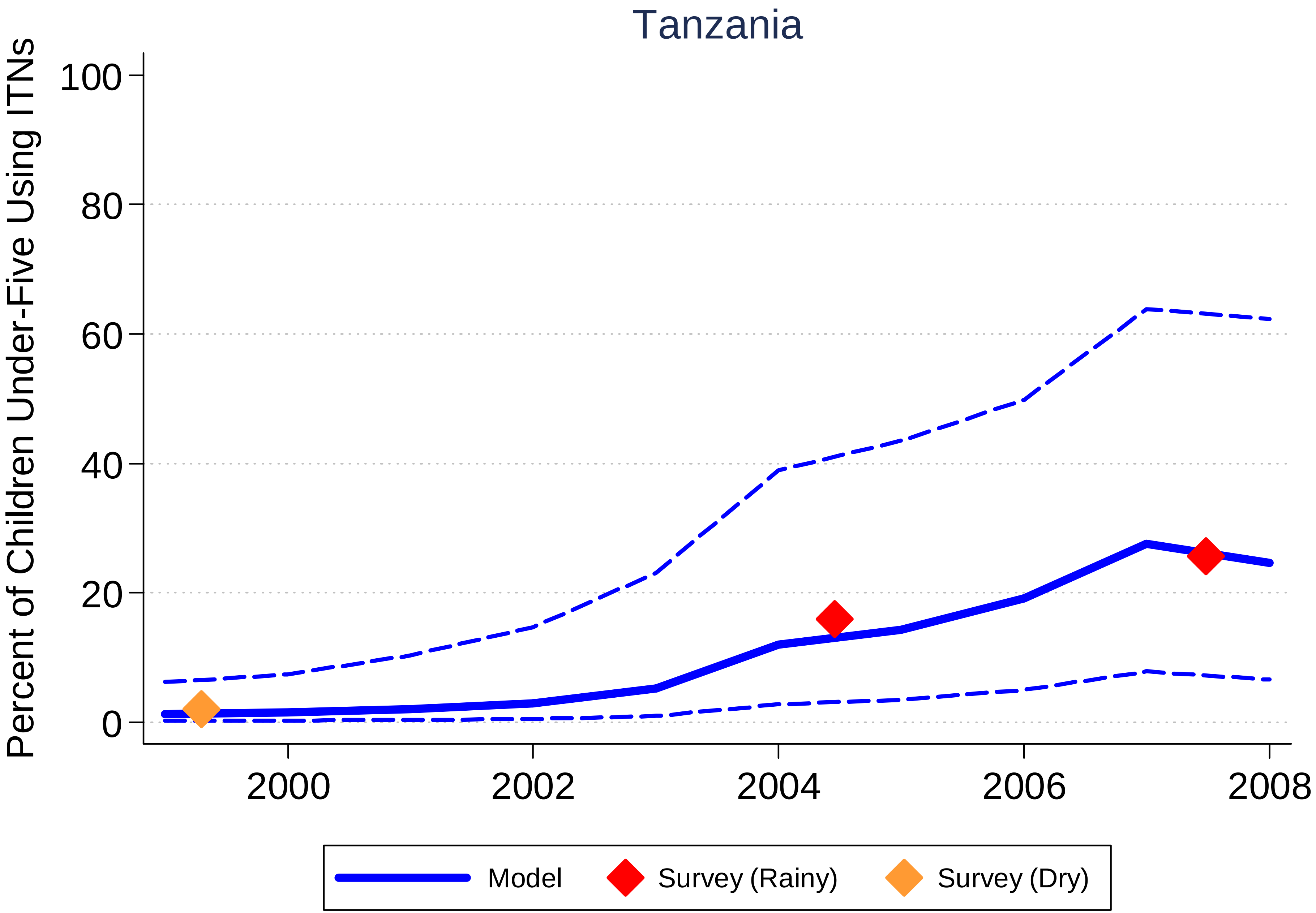

Tanzania
Percent of Children Under-Five Using ITNs
100
80
60
40
20
0
2000
2002
2004
2006
2008
Model
Survey (Rainy)
Survey (Dry)

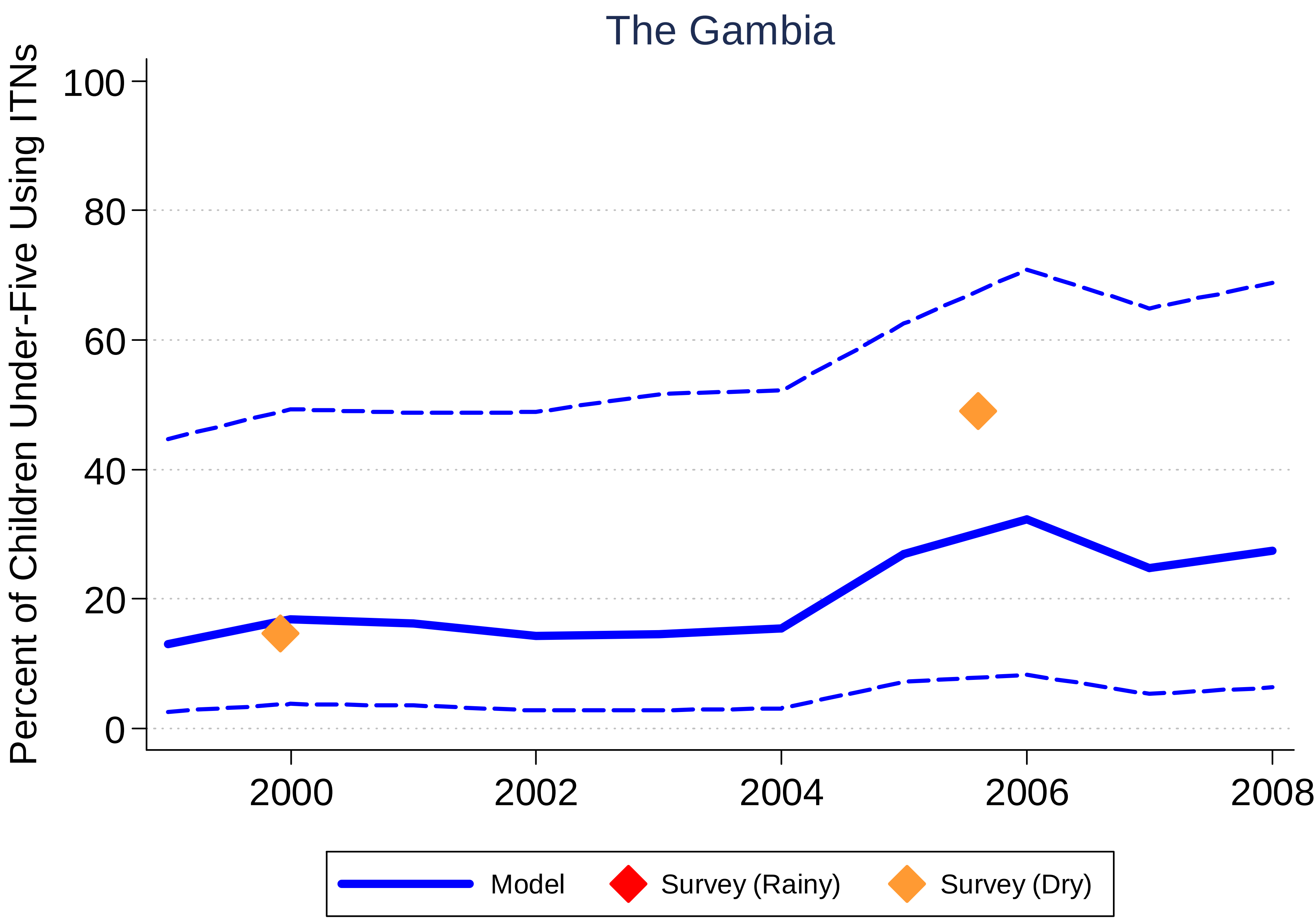

The Gambia
Percent of Children Under-Five Using ITNs
100
80
60
40
20
0
2000
2002
2004
2006
2008
Model
Survey (Rainy)
Survey (Dry)

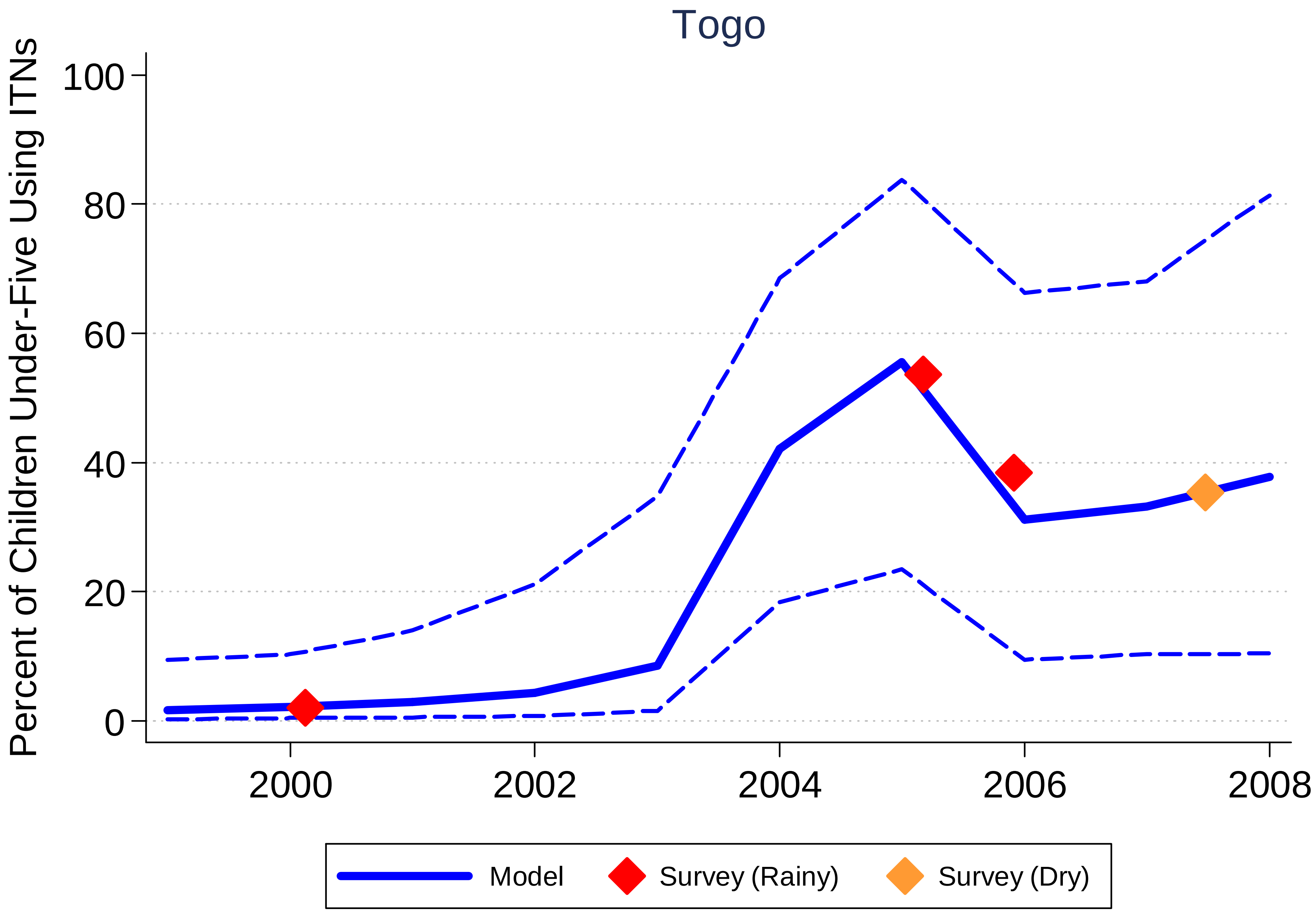
Togo
Percent of Children Under-Five Using ITNs
100
80
60
40
20
0
2000
2002
2004
2006
2008
Model
Survey (Rainy)
Survey (Dry)

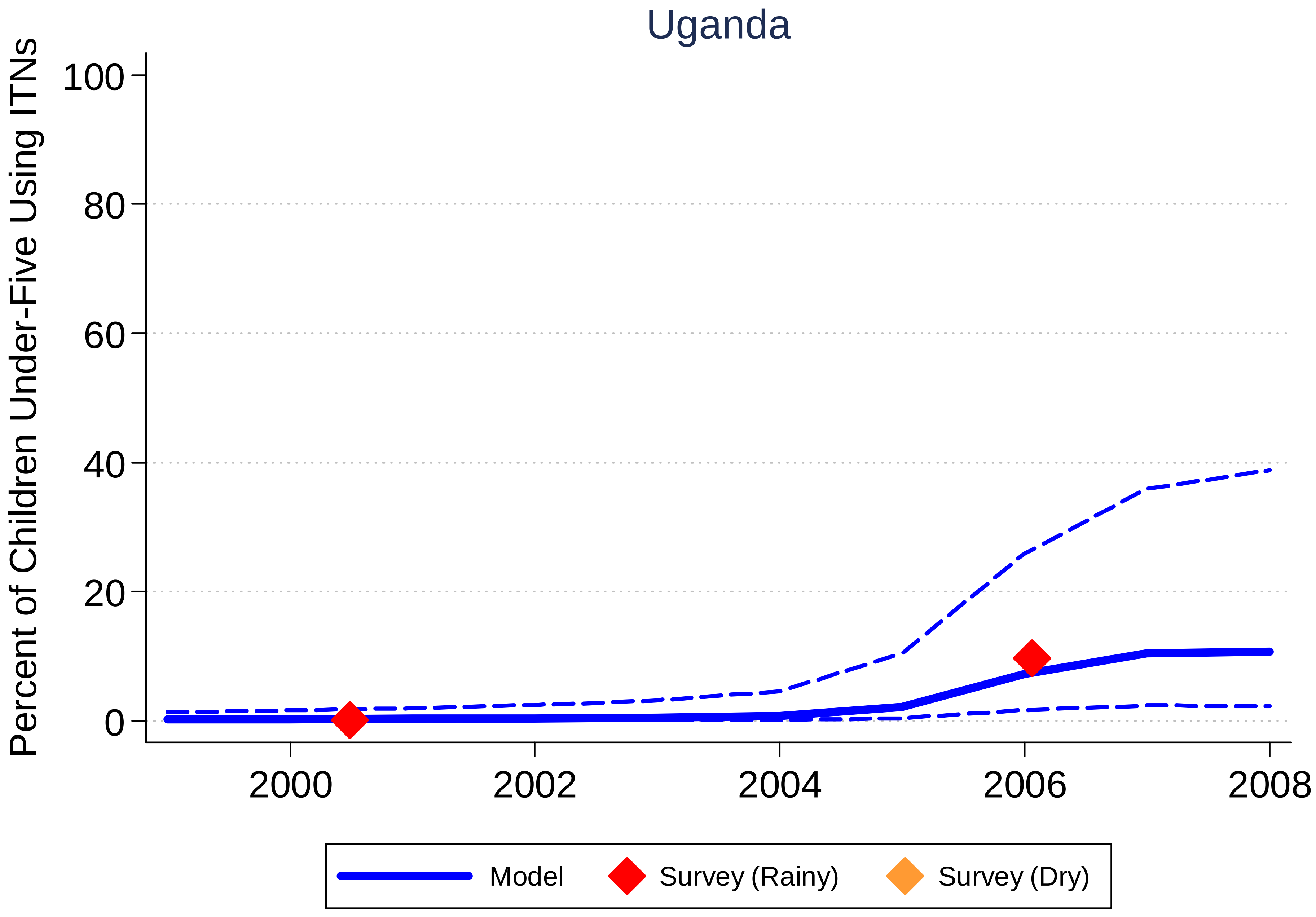

Uganda
Percent of Children Under-Five Using ITNs
0
20
40
60
80
100
2000
2002
2004
2006
2008
Model
Survey (Rainy)
Survey (Dry)

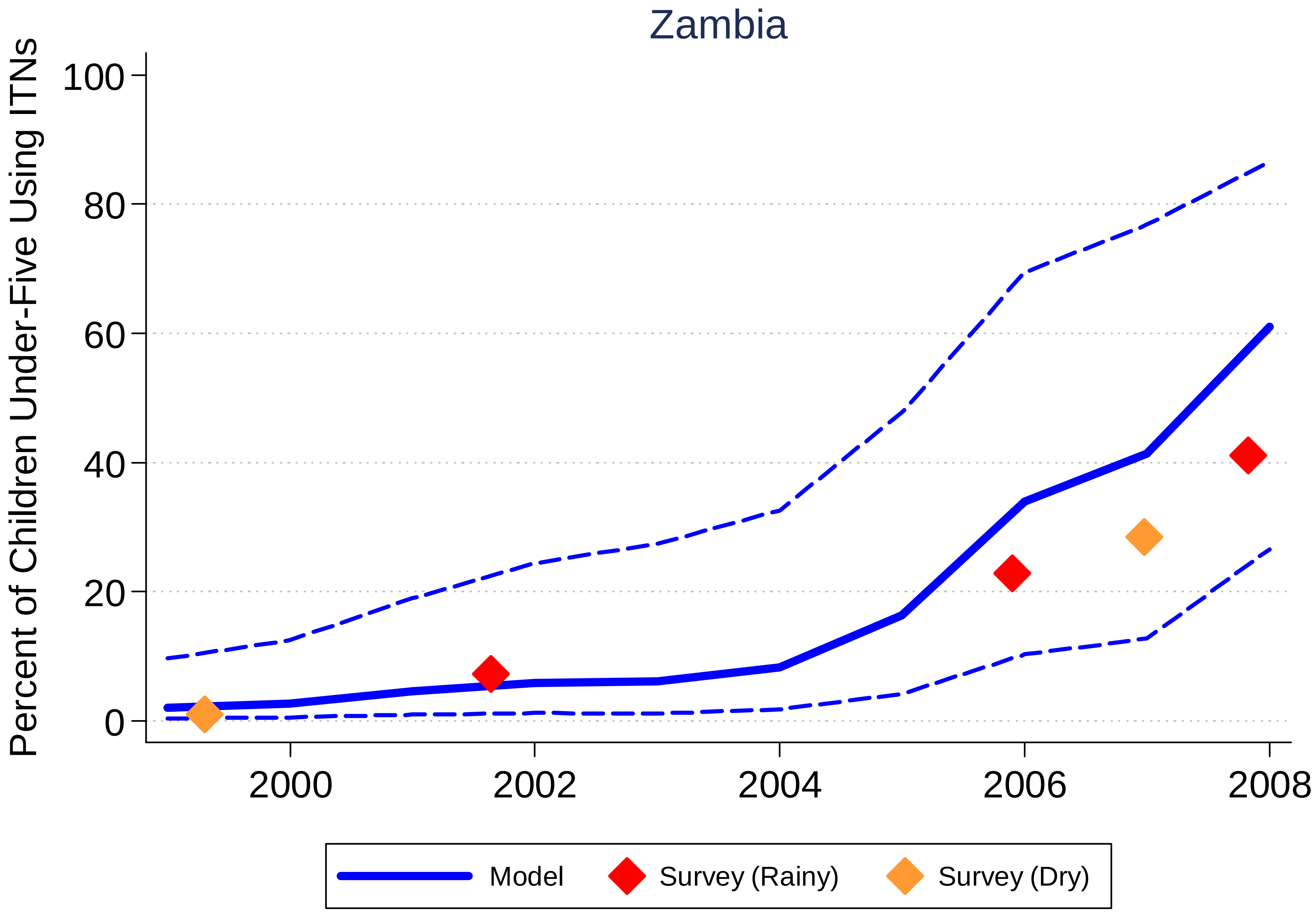

Zambia
Percent of Children Under-Five Using ITNs
100
80
60
40
20
0
2000
2002
2004
2006
2008
Model
Survey (Rainy)
Survey (Dry)

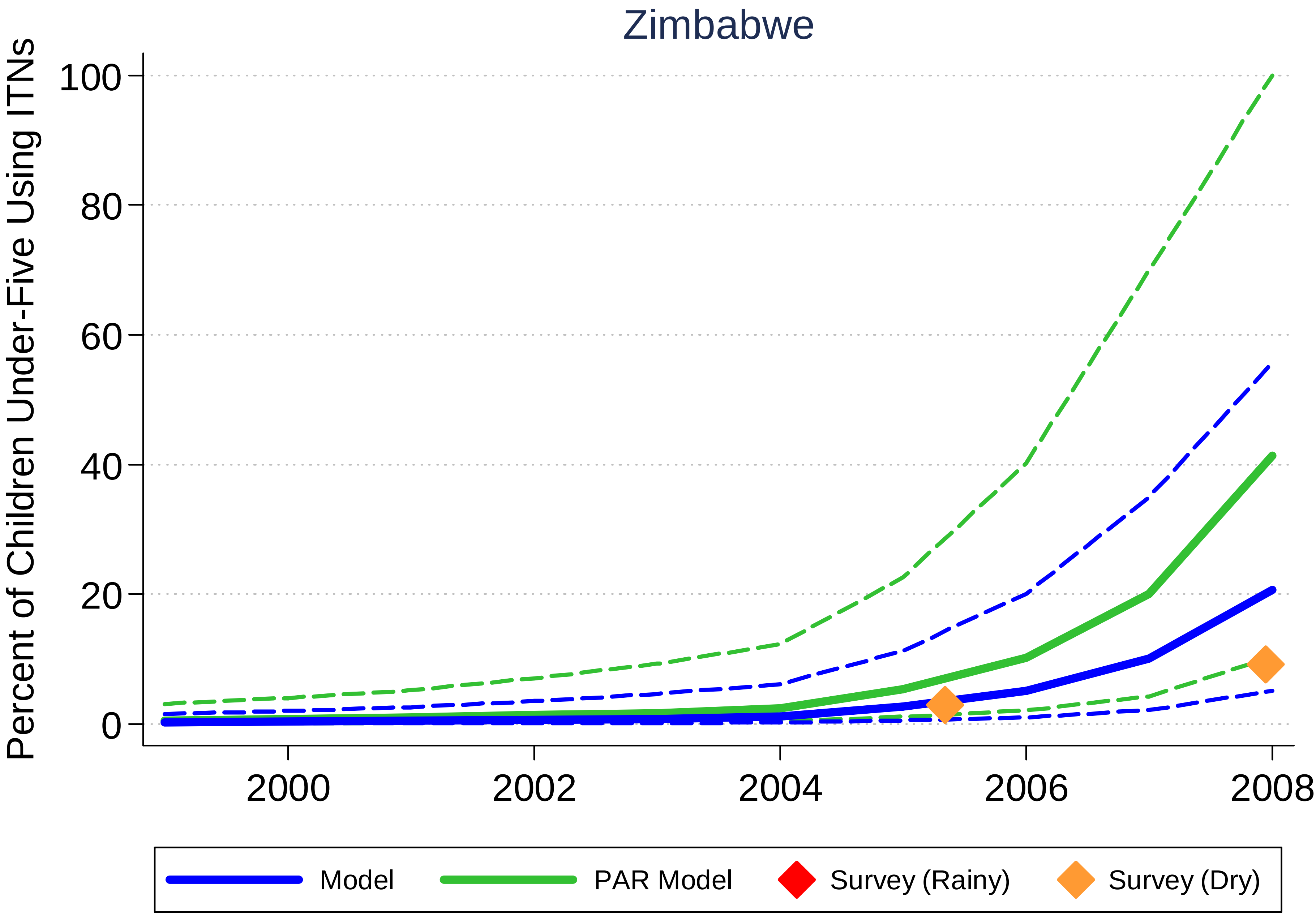

Zimbabwe
Percent of Children Under-Five Using ITNs
0
20
40
60
80
100
2000
2002
2004
2006
2008
Model
PAR Model
Survey (Rainy)
Survey (Dry)
