# Supplementary material for: Rapid Scaling Up of Insecticide-Treated Bed Net Coverage in Africa and Its Relationship with Development Assistance for Health: A Systematic Synthesis of Supply, Distribution, and Household Survey Data
Source: PLoS Med. 2010 Aug 17;7(8):e1000328. doi: 10.1371/journal.pmed.1000328 (PMC2923089; doi:10.1371/journal.pmed.1000328)
Supplement: Table S1 — Published studies used to estimate empirical prior for net discard rate. (0.01 MB PDF) [file pmed.1000328.s003.pdf]

**Table S1. Published studies used to estimate empirical prior for net discard rate.**

| Study | Country, Year | Results |
|---|---|---|
| Hassan et al (2008) [1] | Sudan, 2007 | 92.9% (87.3-96.5%) LLINs retained after 1.5 years |
| Thwing et al (2008) [2] | Niger, 2006 | 97.7% (96.4-98.9%) of households retained at least one LLINs after 9 months |
| Blackburn et al (2006) [3] | Nigeria, 2005 | 94% of ITNs retained after 6 to 8 months |
| Maxwell et al (2006) [4] | Tanzania, | 91.3 to 91.7% of ITNs retained after 3 to 5 years |
| Grabowsky et al (2005) [5] | Ghana, 2003 | 94.4% of ITNs retained after 5 months |
| Lindblade et al (2005) [6] | Kenya, 2004 | 93% of LLINs retained 2 years |

ITNs = Insecticide-treated bed nets; LLINs = Long-lasting insecticide-treated bed nets.

**References**


1. Hassan SHE, Malik EM, Okoued SI, Eltayeb EM (2008) Retention and efficacy of long-lasting insecticide-treated nets distributed in eastern Sudan: a two-step community-based study. Malar J 7: 85.

2. Thwing J, Hochberg N, Eng JV, Issifi S, Eliades MJ et al (2008) Insecticide-treated net ownership and usage in Niger after a nationwide integrated campaign. Trop Med Int Health 13: 827-834.

3. Blackburn BG, Eigege A, Gotau H, Gerlong G, Miri E et al (2006) Successful integration of insecticide-treated bed net distribution with mass drug administration in Central Nigeria. Am J Trop Med Hyg 75: 650-655.

4. Maxwell CA, Rwegoshora RT, Magesa SM, Curtis CF (2006) Comparison of coverage with insecticide-treated nets in a Tanzanian town and villages where nets and insecticide are either marketed or provided free of charge. Malar J 5: 44.

5. Grabowsky M, Nobiya T, Ahun M, Donna R, Lengor M et al (2005) Distributing insecticide-treated bednets during measles vaccination: a low-cost means of achieving high and equitable coverage. Bull World Health 83: 195-201.

6. Lindblade KA, Dotson E, Hawley WA, Bayoh N, Williamson J et al (2005) Evaluation of long-lasting insecticidal nets after 2 years of household use. Trop Med Int Health 10: 1141-1150.
