# Supplementary material for: Rapid Scaling Up of Insecticide-Treated Bed Net Coverage in Africa and Its Relationship with Development Assistance for Health: A Systematic Synthesis of Supply, Distribution, and Household Survey Data
Source: PLoS Med. 2010 Aug 17;7(8):e1000328. doi: 10.1371/journal.pmed.1000328 (PMC2923089; doi:10.1371/journal.pmed.1000328)
Supplement: Table S2 — Total population and population at risk of malaria for 44 African countries. (0.14 MB PDF) [file pmed.1000328.s004.pdf]

Table S2. Total population and population at risk (PAR) of malaria for 44 African countries.

Country	Abbr.	Total Pop.	PAR (%)	Total PAR
Angola	AGO	17,499,410	100	17,499,410
Benin	BEN	9,309,370	100	9,309,370
Botswana	BWA	1,905,516	48	914,648
Burkina Faso	BFA	15,213,310	100	15,213,310
Burundi	BDI	8,856,221	78	6,907,852
Cameroon	CMR	18,920,240	100	18,920,240
Central African Republic	CAF	4,424,292	100	4,424,292
Chad	TCD	11,087,700	99	10,976,823
Comoros	COM	860,100	100	860,100
Congo	COG	3,847,191	100	3,847,191
Cote d'Ivoire	CIV	19,624,240	100	19,624,240
Dem. Rep. of Congo	COD	64,703,620	100	64,703,620
Djibouti	DJI	847,715	37	313,655
Equatorial Guinea	GNQ	519,697	100	519,697
Eritrea	ERI	5,005,678	100	5,005,678
Ethiopia	ETH	85,219,112	68	57,948,996
Gabon	GAB	1,350,156	100	1,350,156
Ghana	GHA	23,946,820	100	23,946,820
Guinea	GIN	9,572,039	100	9,572,039
Guinea-Bissau	GNB	1,745,838	100	1,745,838
Kenya	KEN	38,549,712	76	29,297,780
Liberia	LBR	3,942,212	100	3,942,212
Madagascar	MDG	20,215,200	100	20,215,200
Malawi	MWI	14,288,370	100	14,288,370
Mali	MLI	12,716,080	100	12,716,080
Mauritania	MRT	3,203,648	70	2,242,554
Mozambique	MOZ	21,812,550	100	21,812,550
Namibia	NAM	2,102,140	67	1,408,434
Niger	NER	14,730,800	100	14,730,800
Nigeria	NGA	151,478,096	100	151,478,096
Rwanda	RWA	10,008,620	100	10,008,620
Sao Tome & Principe	STP	160,174	100	160,174
Senegal	SEN	12,687,620	100	12,687,620
Sierra Leone	SLE	5,968,523	100	5,968,523
Somalia	SOM	8,956,006	88	7,881,285
South Africa	ZAF	48,832,128	11	5,371,534
Sudan	SDN	39,445,020	100	39,445,020
Swaziland	SWZ	1,148,255	36	413,372
Tanzania	TZA	41,463,920	100	41,463,920
The Gambia	GMB	1,754,068	100	1,754,068
Togo	TGO	6,762,421	100	6,762,421
Uganda	UGA	31,902,610	100	31,902,610
Zambia	ZMB	12,154,060	100	12,154,060
Zimbabwe	ZWE	13,481,230	50	6,740,615
