# Supplementary material for: Rapid Scaling Up of Insecticide-Treated Bed Net Coverage in Africa and Its Relationship with Development Assistance for Health: A Systematic Synthesis of Supply, Distribution, and Household Survey Data
Source: PLoS Med. 2010 Aug 17;7(8):e1000328. doi: 10.1371/journal.pmed.1000328 (PMC2923089; doi:10.1371/journal.pmed.1000328)
Supplement: Text S1 — Bayesian estimation of ITN coverage. (0.76 MB PDF) [file pmed.1000328.s005.pdf]

Text S1.
Bayesian estimation of ITN coverage

1 Introduction

This Web appendix describes our application of Bayesian inference to impute missing data for ITN coverage. The challenge we face is to devise an objective and replicable method for estimating survey-based measurements of coverage of ITN ownership, and also to appropriately represent the uncertainty in these estimates. Bayesian inference is well-suited for this task [1–5], and we use it together with a stock-and-flow model of the ITN supply chain to resolve the issue that data from manufacturers, agencies, and households measure different points along the supply and distribution chain.

We face extreme missingness, where no survey measurement of coverage is available for 75% of country-years. Standard approaches to missing data, such as multiple imputation [6] or bi-directional distance dependent regression [7], are unable to cope with this level of missingness. We triangulate all relevant data and incorporate expert prior beliefs by designing a deterministic compartmental model of the LLIN supply chain, and then using Bayesian inference to combine data derived from direct and indirect measurements of the stocks and flows in the compartmental model. This permits us to generate harmonized estimates incorporating the survey data that does exist, expert priors, reports from manufacturers and National Malaria Control Programs (NMCPs) on the LLIN supply chain, and empirical priors on how LLINs are retained.

The compartmental model we develop defines precise relationships between net supply, distribution, and ownership over time; for example, for a net to be used in a household today, it must have been obtained by the household sometime previously, and, before that, it must have been manufactured and delivered for distribution. We formalize this via a compartmental model with parameters describing the supply, distribution, ownership, and loss of nets by households. The model uses a discrete one-year time step and allows flows into a compartment to be part of flows out of the compartment for the same year. This approach ensures that our estimates of supply, distribution, ownership, and loss of nets are consistent over time.

We use Bayesian inference to estimate the parameters of the model from all available data. This requires specifying a data likelihood function for the observations as a function of the model parameters, and then inverting this probability with Bayes' theorem and specified prior distributions to obtain the parameter posterior distributions. We accomplish this task computationally with a Markov Chain Monte Carlo (MCMC) algorithm as implemented by the Python package PyMC [8].

The remainder of this document is organized as follows: Section 2 describes the compartmental model of the ITN supply chain. Section 3 describes the statistical model, which allows us to perform Bayesian inference on the deterministic model from Section 2. Section 4 concludes with an explanation of the computational approach we use for Bayesian inference, which is to draw samples from the model posterior distribution with the popular Markov Chain Monte Carlo (MCMC) technique.

2 Compartmental model

This section describes our deterministic model of the ITN distribution system within a single country c. It is based on a five compartment model, depicted in Figure 1, which shows how LLINs arrive in the country from manufacturers and are then distributed to households, where they are used and eventually discarded (or cease to be ITNs). The model uses discrete one-year time steps, which matches the available data. The model allows flows into a compartment to contribute to flows out of the compartment during the same year.

To make this exposition as simple as possible, the model is introduced in three parts. Section 2.1

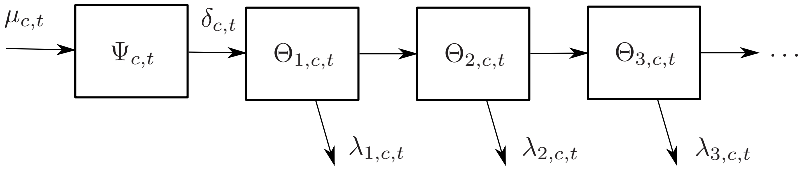

Figure 1. Stock-and-flow model of the ITN distribution system within country c at time t. Stocks (denoted by capital Greek letters) are included for the LLINs in the country but not yet in households ($\Psi_{c,t}$), and the LLINs that have been in households for y years ($\Theta_{1,c,t}, \Theta_{2,c,t}, \Theta_{3,c,t}$). Flows (denoted by lower-case Greek) are included for LLINs sent to the country ($\mu_{c,t}$), LLINs distributed to households ($\delta_{c,t}$), and LLINs discarded by households after $(i-1)$ to i years ($\lambda_{1,c,t}, \lambda_{2,c,t}, \lambda_{3,c,t}$).

defines the stock-and-flow parameters in the model, and Section 2.2 defines how these stocks and flows are related temporally. Section 2.3 defines and justifies additional model parameters that we use to map from LLIN household stock to LLIN and ITN coverage. Section 2.4 uses some examples to illustrate the power of this compartmental model.

2.1 Stock-and-flow parameters

Our compartmental model has the following stock parameters for each time t from 1999 to 2009:

$$\begin{aligned}\Psi_{c,t} &= \text{number of LLINs in country } c \text{ available for distribution or purchase by households} \\ &\quad \text{at the start of year } t \\ \Theta_{1,c,t} &= \text{number of LLINs that have been in households for 0 to 1 year at the start of year } t \\ \Theta_{2,c,t} &= \text{number of LLINs that have been in households for 1 to 2 years at the start of year } t \\ \Theta_{3,c,t} &= \text{number of LLINs that have been in households for 2 to 3 years at the start of year } t\end{aligned}$$

The model also includes the following flow parameters for each one-year time period t from 1999 to 2008:

$$\begin{aligned}\mu_{c,t} &= \text{number of LLINs sent to country } c \text{ during year } t \\ \delta_{c,t} &= \text{number of LLINs distributed to households during time period } t \\ \lambda_{i,c,t} &= \text{number of } (i-1)\text{- to } i\text{-year-old LLINs discarded by households} \\ &\quad \text{during time period } t \text{ for } i = 1, 2, 3\end{aligned}$$

2.2 Stock-and-flow dynamics

This section describes a set of straightforward relationships between the stock and flow parameters over time. $\Psi_{c,t}$ denotes the number of LLINs in the country but not in households at the start of year t. The number of LLINs at the start of the next year differs from $\Psi_{c,t}$ according to $\mu_{c,t}$, the number of LLINs sent to the country during year t, and $\delta_{c,t}$, the number of LLINs distributed to households during that same period:

$$\Psi_{c,t+1} = \Psi_{c,t} + \mu_{c,t} - \delta_{c,t}.$$

Note that this formulation permits nets to be distributed during the year they are received. Perfect throughput would yield $\delta_{c,t} = \mu_{c,t}$ and $\Psi_{c,t} = 0$.

$\Theta_{1,c,t}$ denotes the number of LLINs that have been in households for 0 to 1 year at the start of year t. It is precisely the number of LLINs distributed to households during the previous year:

$$\Theta_{1,c,t+1} = \delta_{c,t}.$$

For $i > 1$, $\Theta_{i,c,t}$ denotes the number of LLINs that have been in households for $(i-1)$ to i years at the start of year t, and is given by

$$\Theta_{i,c,t+1} = \Theta_{i-1,c,t} - \lambda_{i-1,c,t} \quad \text{for } i = 2, 3.$$

These model dynamics enforce one major assumption in the compartmental model, which is about LLIN lifetime: LLINs cease to be effective after three years. At all times, the 3-plus-year-old LLIN stock is 0.

2.3 Additional model parameters

This section describes some additional model parameters, which complement the stock and flow parameters introduced above, and allow us to meld additional relevant data. We begin with the parameters we use in mapping between LLIN stock and LLIN coverage (defined as the fraction of households with at least one net).

We model the distribution of nets in households as a negative binomial distribution, which fits all available data with RMSE below the survey sampling error (Figure 2 shows this graphically).

The negative binomial distribution is controlled by its mean and its dispersion, and we parametrize it by introducing coverage parameter η_c and dispersion parameter α_c and taking $\eta_c\Theta_{c,t}/\operatorname{pop}_{c,t}$ to be the mean of the negative binomial and α_c to be the dispersion (where $\Theta_{c,t} = \sum_{i=1}^{3} \Theta_{i,c,t}$ is the LLIN stock in country c at time t). The probability that a negative binomial with these parameters is 0 is then

$$\Pr\left[\text{NegativeBinomial}\left(\eta_c\Theta_{c,t}/\operatorname{pop}_{c,t}\right) = 0\right] = \left(\frac{\alpha_c}{\eta_c\Theta_{c,t}/\operatorname{pop}_{c,t} + \alpha_c}\right)^{\alpha_c}.$$

We introduce $\Delta_{c,t}$ to denote the LLIN coverage in country c at time t, and then the negative binomial model implies that

$$\Delta_{c,t} = 1 - \left(\frac{\alpha_c}{\eta_c\Theta_{c,t}/\operatorname{pop}_{c,t} + \alpha_c}\right)^{\alpha_c}.$$

In order to define an analogous formula for ITN coverage (which includes non-LLINs as well as LLINs), we use parameter $\Omega_{c,t}$ to denote the non-LLIN ITN stock in country c at time t. Then, using $\Sigma_{c,t}$ to denote the ITN coverage in country c at time t, we have:

$$\Sigma_{c,t} = 1 - \left(\frac{\alpha_c}{\eta_c(\Theta_{c,t} + \Omega_{c,t})/\operatorname{pop}_{c,t} + \alpha_c}\right)^{\alpha_c}.$$

2.4 Examples: inferences from consistency of the compartmental model

The compartmental model above is simple, but it has direct implications on the consistent set of parameter values. For example, the number of LLINs that have been in households for 1 to 2 years in 2009 is at most the number of LLINs that have been in households for 0 to 1 year in 2008. Or, in other words, $\Theta_{2,2009} \geq \Theta_{1,2008}$.

This sort of reasoning can be quantitative as well. For example, if there are 1 million LLINs in the country but not in households at the start of 2008, .5 million LLINs are sent to the country during 2008, and 1.2 million LLINs are distributed to households during 2008, then there are $\Psi_{2008} + \mu_{2008} - \delta_{2008} = .3$ million LLINs in the country but not in households at the start of 2009.

In order to systematically leverage consistency conditions like these, the next section connects the model parameters to observed data through a Bayesian statistical model.

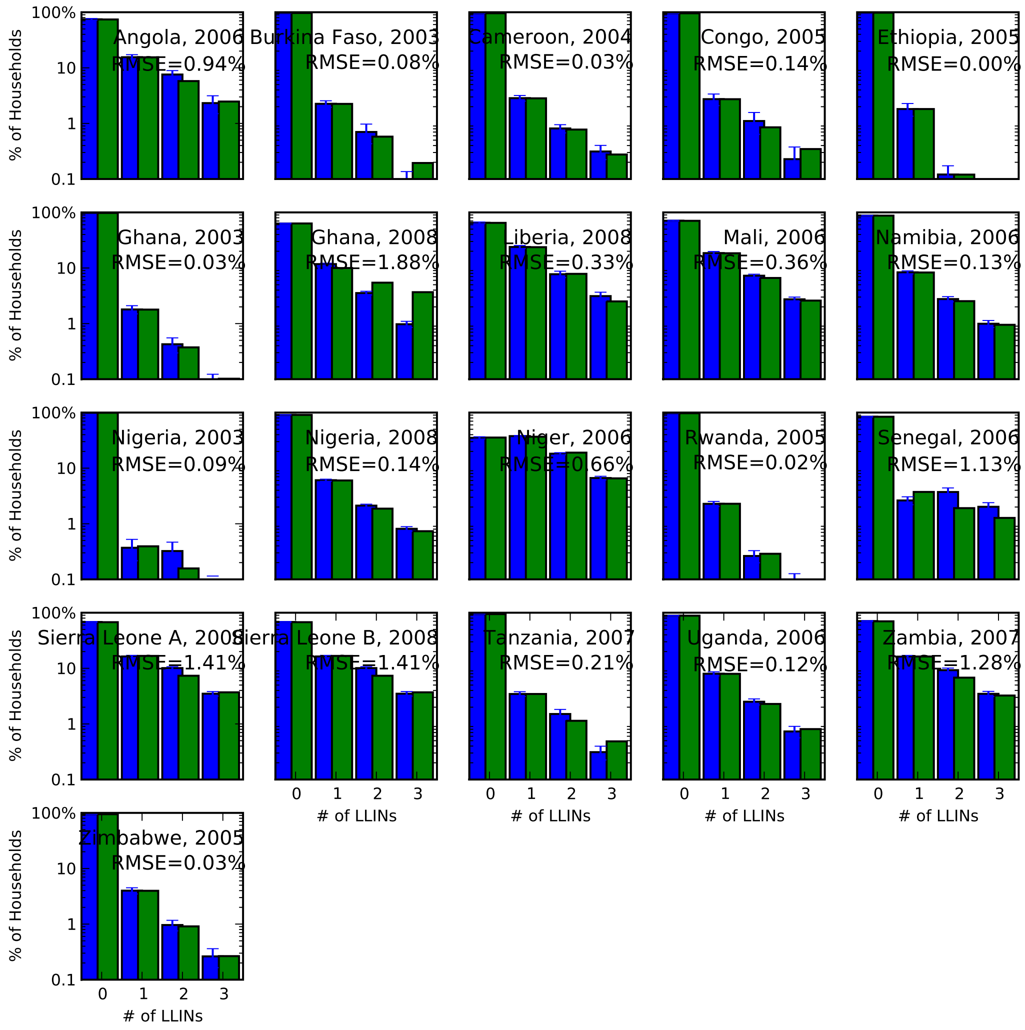

Figure 2. Validation of the negative binomial model for fraction of nets in households. Blue bars show the fraction of households from survey data, and green bars show the negative binomial distribution that fits the data with minimum RMSE. The root mean squared survey error is larger than the fit error for all surveys.

Data Source	Observed Value	Expectation	Standard Deviation
Manufacturer LLIN Supply	$\log m_{c,t}$	$\log \mu_{c,t}$	σ_{m_c}
NMCP LLIN Distribution	$\log d^r_{c,t}$	$\log(\delta_{c,t}) + \epsilon_c$	$\sigma_{d^r_c}$
Survey ITN Coverage	$\text{ITN}_{c,t}$	$\Sigma_{c,t}$	$\sigma_{\text{ITN}_{c,t}}$
Survey LLIN Coverage	$\text{LLIN}_{c,t}$	$\Delta_{c,t}$	$\sigma_{\text{LLIN}_{c,t}}$
Survey LLIN in Households	$s_{c,t}$	$\Theta_{c,t}$	$\sigma_{s_{c,t}}$
Survey LLIN Distribution	$d_{c,t}$	$\delta_{c,t} \cdot (1-\pi_c)^{t_s-(t+.5)}$	$\sigma_{d_{c,t}}(1+\sigma_{d_c})$
Survey Report ITN Coverage	$\text{ITN}^r_{c,t}$	$\Sigma_{c,t}$	$\sigma_{\text{ITN}_{c,t}}\gamma_c$

Table 1. Country-specific data sources for country c, together with their expectations and standard deviations. In this table, all data values are normalized, i.e., instead of listing log-normally-distributed data $m_{c,t}$, we list normally distributed data $\log(m_{c,t})$.

3 Bayesian Statistical Model

This section takes the model parameters from the compartmental model in Section 2 and connects them to all relevant sources of data using Bayesian inference. The data sources are the following: manufacturers' reports on LLINs sent to the country during a year ($m_{c,t}$); NMCP reports on LLINs distributed to households through public health programs ($r_{c,t}$); survey reports on ITN coverage ($\text{ITN}^r_{c,t}$); household survey data on ITN coverage ($\text{ITN}_{c,t}$), LLIN coverage ($\text{LLIN}_{c,t}$), the number of LLINs in households ($N_{c,t}$), and the number of LLINs distributed to households during one-year time periods $d_{c,t}$. Table 1 lists these data types and their model-based expectations and standard deviations.

This section is divided into three parts. Section 3.1 addresses how we model the data likelihood for each type of data to be melded. Section 3.2 and 3.3 define and justify the prior probabilities selected for the model parameters, which we inform with an empirical Bayes approach whenever possible.

3.1 Data Likelihoods

In order to apply Bayesian inference, we must define the data likelihood as a function of the model parameters. There are several types of data we will use to estimate consistent parameters for the compartmental model from the previous section, and we must define a joint likelihood function for all of them. We assume that this joint likelihood factors, and model it as the product of the individual likelihood described below. This assumption implies that, conditioned on the model parameters, the values of manufacturer, NMCP, and household surveys and reports are all independent.

Manufacturers' reports provide information about the number of LLINs sent to the country during a given year. We model the manufacturer-reported number as a log-normally distributed realization of the model parameter $\mu_{c,t}$. We introduce an additional parameter σ_{m_c} to represent the dispersion of the log-normal, which will be fit from the data together with $\mu_{c,t}$ and the other model parameters. Thus, a report that $m_{c,t}$ LLINs were sent to the country during year t has likelihood given by

$$\log(m_{c,t}) \sim \text{Normal}\left(\log(\mu_{c,t}), \sigma^2_{m_c}\right).$$

In the absence of data, we select the following prior for the dispersion parameter, based on expert judgment:

$$\sigma_{m_c} \sim \text{Lognormal}(\log(.05), .5^2)$$

This corresponds to a standard error of 5% in the manufacturers' data and a standard deviation of 5% in this standard error. In other words, the manufacturers' reports for the country being 5% or 15% higher or lower is likely, but being 30% higher or lower is unlikely.

NMCP reports provide information about the number of LLINs distributed in each country. These numbers were reported to WHO by NMCPs and, like the manufacturers' reports, they also do not include a measure of uncertainty. They are subject to bias, as described in the methods section of the paper. For this reason, and because comparing the NMCP report data with survey data on LLINs distributed during the same time period shows that there are systematic differences (as well as random variation) between these data sources, we model the NMCP-reported numbers as log-normally distributed random variables that are not centered on the model parameter distribution $\delta_{c,t}$, but on a systematically biased function of this parameter. To implement this, we introduce two parameters, ϵ_c and $\sigma_{d^r_c}$, to represent the offset and dispersion of the NMCP report data; these parameters are fit together with $\delta_{c,t}$ and the other model parameters. An NMCP report that $d^r_{c,t}$ LLINs were distributed during year t then has likelihood given by:

$$\log(d^r_{c,t}) \sim \text{Normal}\left(\log(\delta_{c,t}) + \epsilon_c, \sigma^2_{d^r_c}\right).$$

Empirical priors for ϵ_c and $\sigma_{d^r_c}$ are fit from the NMCP data and survey data, as described in Section 3.3.

Household survey data on LLIN stocks provide the most direct estimate of a parameter in the compartmental model. This information comes from household surveys, where an interviewer directly observes the number of nets and the brand of each net to determine if it is an LLIN. In addition to an estimate of total LLINs in households, the survey design provides a rigorous uncertainty interval around the estimate. We model the household survey data on LLIN stock at time t as normally distributed with mean $\Theta_{c,t} = \sum_{i=1}^{4} \Theta_{i,c,t}$ and standard deviation given by the standard error of the household survey.

To accommodate the fact that no survey is instantaneous, we use the mean survey date (in years) for t. Mean survey dates do not correspond with the time step in the compartmental mode (i.e., they are not Jan. 1), so we use linear interpolation to estimate stock values at intermediate times within a year. For example, a survey in Congo was conducted during 2008 that has mean survey date August 30, 2008; for this, we model the observed value at the value three-quarters of the way from 2008 to 2009, i.e., $t = 2008.75$ and $s_{c,t}$ is a normally distributed realization of $(.25)\Theta_{\text{Congo},2008} + (.75)\Theta_{\text{Congo},2009}$. In general, if $t = \lfloor t \rfloor + r$, where $\lfloor t \rfloor$ is the largest integer less-than-or-equal-to t, and r is the residual $r = t - \lfloor t \rfloor$, we have $\Theta_{c,t} = (1-r)\Theta_{c,\lfloor t \rfloor} + r\Theta_{c,\lfloor t \rfloor + 1}$. The likelihood of a household survey finding LLINs stock of $s_{c,t}$ with standard error $\sigma_{s_{c,t}}$ is thus given by:

$$s_{c,t} \sim \text{Normal}\left(\Theta_{c,t}, \sigma^2_{s_{c,t}}\right).$$

Household survey data on LLIN distribution can also be extracted from the DHS, MICS, and MIS surveys used for measuring household LLIN stock. These interviews include questions about how long ago each net was acquired, and this constitutes a direct measurement of the number of LLINs in households at the time of the survey that were received during time period t for 2 or 3 time periods before the survey was conducted. These are not direct measurements of the quantity $\delta_{c,t}$, however, because some LLINs have been discarded in the period of time between receipt and the survey interview. For a survey conducted at time t^s, the tally of LLINs distributed during time period t is a measurement of $\delta_{c,t} - \sum_{i=1}^{t^s - t} \lambda_{i,c,t+i}$. We model net loss as a constant rate, i.e. $\lambda_{i,c,t} = \pi_c \Theta_{i,c,t}$ for all i and t, and LLIN acquisition at mid-year, so the expected value above simplifies to $\delta_{c,t} \cdot (1-\pi_c)^{t^s - (t+.5)}$. However, the survey responses are also subject to recall bias. To account for this, we introduce a recall bias parameter σ_{rb_c}, and model the dispersion of the LLIN distribution survey data as the sampling error of the survey scaled up by a factor of $1 + \sigma_{rb_c}$. All together, this yields the following likelihood for observing $d_{c,t}$ LLINs in households at time t^s that were distributed during time period t with standard error $\sigma_{d_{c,t}}$:

$$d_{c,t} \sim \text{Normal}\left(\delta_{c,t} \cdot (1-\pi_c)^{t^s - (t+.5)}, \left(\sigma_{d_{c,t}} (1 + \sigma_{rb_c})\right)^2\right).$$

An empirical prior for π_c is developed from published studies on bed net retention behaviors, as described in Section 3.3. For the recall bias parameter, we choose a prior of

$$\sigma_{rb_c} \sim \text{Lognormal}(\log(.0)5, .5^2),$$

which corresponds to a belief that recall bias contributes about an additional 10% error beyond the survey sampling error and about a 5% standard deviation in this standard error. In other words, it would be likely to find that recall bias increases the error by 5% or by 15%, but it would be unlikely to learn that recall bias increases the error by 30%.

Household survey data on ITN and LLIN coverage also measures an important and relevant quantity that does not appear directly in the compartmental model. Predicting the percentage of households with at least 1 ITN is the primary objective of this analysis. Fortunately, there is a strong correlation between the number of ITNs in households and ITN coverage. Since coverage cannot exceed 100%, we do not model this as a linear relationship. Instead we use the 2 parameter non-linear function described in Section 2.3 to map household stocks to household coverage: we model the fraction of households with at least one LLIN as $\Delta_{c,t} = 1 - \left(\frac{\alpha_c}{\eta_c \Theta_{c,t}/\operatorname{pop}_{c,t} + \alpha_c}\right)^{\alpha_c}$, where η_c and α_c are country-specific coverage parameters, and $\operatorname{pop}_{c,t}$ is the population of country c at time t. To model the fraction of households with at least one ITN, we introduce an additional parameter $\Omega_{c,t}$ to represent the non-LLIN ITN stock in country c at time t, and then, similar to the LLIN case above, we model the fraction of households with at least one ITN as $\Sigma_{c,t} = 1 - \left(\frac{\alpha_c}{\eta_c(\Theta_{c,t}+\Omega_{c,t})/\operatorname{pop}_{c,t} + \alpha_c}\right)^{\alpha_c}$, where η_c, α_c and $\operatorname{pop}_{c,t}$ are the same as above. With these models in hand, the survey-based direct measurements of $\mathrm{ITN}_{c,t}$ and $\mathrm{LLIN}_{c,t}$ with standard errors $\sigma_{\mathrm{ITN}_{c,t}}$ and $\sigma_{\mathrm{LLIN}_{c,t}}$ are modeled as

$$\mathrm{LLIN}_{c,t} \sim \mathrm{Normal}\left(\Delta_{c,t}, \left(\sigma_{\mathrm{LLIN}_{c,t}}\right)^2\right)$$
$$\mathrm{ITN}_{c,t} \sim \mathrm{Normal}\left(\Sigma_{c,t}, \left(\sigma_{\mathrm{ITN}_{c,t}}\right)^2\right)$$

Survey reports on coverage provide similar information to the household survey coverage measurements, but often without information to quantify the survey design effects on uncertainty. We model the data from these reports by introducing a survey design effect parameter γ_c. If the report says that the sample size is $N_{c,t}$, then we calculate the raw sampling error as $\sigma_{\mathrm{ITN}^r_{c,t}} = \mathrm{ITN}^r_{c,t}(1 - \mathrm{ITN}^r_{c,t})/\sqrt{N_{c,t}}$, and model the likelihood as

$$\mathrm{ITN}^r_{c,t} \sim \mathrm{Normal}\left(\Sigma_{c,t}, \left(\sigma_{\mathrm{ITN}^r_{c,t}} \cdot \gamma_c\right)^2\right).$$

An empirical prior for γ_c is selected by comparing the sampling error assuming simple random sampling to the sampling error taking into account the complex survey design for the surveys where we have microdata available, as described in Section 3.3.

3.2 Bayesian Priors

In order to fit the model with Bayesian inference, we must specify priors on the parameters of the compartmental model. In data-rich settings, the model estimates will be driven by the data, and the prior values will not make an appreciable difference. In settings where there is not much data available, the noninformative priors will lead to credible estimates that have appropriately wide uncertainty intervals.

We use the following noninformative priors:

$$\log \mu_{c,t} \sim \begin{cases} \mathrm{Normal}\left(\log\left(.001\operatorname{pop}_{c,t}\right), 2^2\right), & \text{if } t \geq 2004; \\ \mathrm{Normal}\left(\log\left(.001\operatorname{pop}_{c,t}\right), .2^2\right), & \text{if } t \leq 2003; \end{cases}$$

$$\log \delta_{c,t} \sim \begin{cases} \mathrm{Normal}\left(\log\left(.001\right)\operatorname{pop}_{c,t}\right), 2^2\right), & \text{if } t \geq 2004; \\ \mathrm{Normal}\left(\log\left(.001\right)\operatorname{pop}_{c,t}\right), .2^2\right), & \text{if } t \leq 2003; \end{cases}$$

$$\log \Omega_{c,t} \sim \mathrm{Normal}\left(\log\left(.001\operatorname{pop}_{c,t}\right), 2^2\right)$$

Parameter	Value	Prior Expectation	Prior Standard Deviation
LLIN Discard Rate	π_c	.051	.026
NMCP Flow Bias	ϵ_c	.92	.22
NMCP Flow Error	$\sigma_{d_c^r}$	1.4	.17
ITN Coverage Parameter	η_c	4.0	.12
ITN Dispersion Parameter	α_c	3.0	1.2
Survey Design Error	γ_c	1.9	.37

Table 2. Empirical priors, together with their expectations and standard deviations.

For stocks, we start the system dynamics model in 1999, before LLINs were introduced, and we impose a restriction that stock variables are never negative:

$$\log \Psi_{c,1999} = 0$$
$$\log \Theta_{c,1999} = 0$$
$$\Psi_{c,t}, \Theta_{c,t}, \Omega_{c,t} \geq 0$$

We include three additional priors that encode beliefs about time-trends in the distribution system. A "proven capacity" prior captures the belief that, if a given quantity of nets was distributed during a given year, this is evidence that the supply chain *can* distribute this many nets, and so it is unlikely for many less to be distributed in subsequent years (provided nets are available for distribution). We approximate flow by mid-year stock for non-LLIN ITNs, and formalize this prior as:

$$\left(\log(\Omega_{c,t+.5} + \delta_{c,t}) - \max_{t'<t} \log\left(\Omega_{c,t'+.5} + \delta_{c,t'}\right)\right)^{-} \sim \text{Normal}(0, .5^2),$$

where $(x)^{-} = \begin{cases} x, & \text{if } x < 0; \\ 0, & \text{otherwise.} \end{cases}$

An "ITN composition" prior captures the expert knowledge that LLINs became more prevalent than non-LLIN ITNs as they became available. We formulate this in terms of household ownership stock:

$$\frac{\Theta_{c,t}}{\Theta_{c,t} + \Omega_{c,t}} \sim \begin{cases} \text{Normal}(0, .5^2) & \text{if } t \leq 2001; \\ \text{Normal}(1, .5^2) & \text{if } t \geq 2005. \end{cases}$$

A "coverage smoothing" prior represents the expert belief that coverage levels do not change drastically year-to-year. This is formalized as:

$$\log \Sigma_{c,t} - \log \Sigma_{c,t-1} \sim \text{Normal}(0, .5^2).$$

3.3 Empirical Bayesian Priors

Whenever possible, we use data-based estimates to select the Bayesian parameters in the statistical model above. This empirical-Bayes approach typically takes the form of fitting a mixed effects model across all country-years where a direct measurement of the quantity of interest can be made, and using the results to select priors for the country-specific model described above. Table 2 summarizes the results of this approach.

To select a prior for the *LLIN Discard Rate* (π_c), we pool six studies on LLIN retention (listed in Web Appendix C), each of which reports the fraction of nets remaining in households (r_i) after some follow-up time period has elapsed (T_i). We model the observed value as

$$r_i \sim \text{Normal}\left((1 - \pi_c)^{T_i}, \sigma^2\right),$$

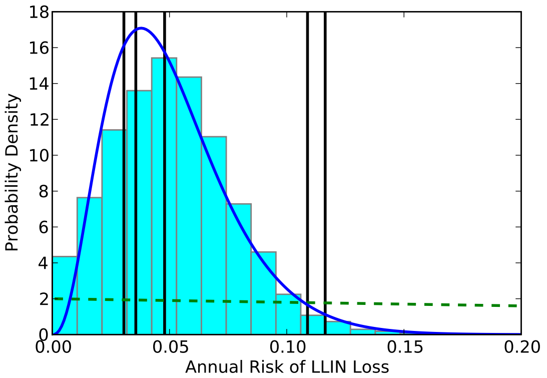

Figure 3. Empirical prior for LLIN Discard Rate (π_c). Dashed green curve is the hyper-prior, black vertical lines represent the data, cyan histogram shows the hyper-posterior, and the solid blue curve shows the empirical prior, which is used for Bayesian inference in the compartmental model.

where σ is an additional parameter introduced to model the dispersion of the studies. To complete the model, we select uninformative (hyper)-priors for π_c and σ,

$$\pi_c \sim \text{Beta}(1, 2)$$
$$\sigma \sim \text{InverseGamma}(11, 1)$$

Sampling the posterior distribution with MCMC (220000 samples, of which the first 20000 were discarded and the remaining 20000 were thinned by a factor of 20) yields a posterior distribution of π_c with mean .051 and standard deviation .026, so we use the Beta distribution with this mean and standard deviation as an empirical prior,

$$\pi_c \sim \text{Beta}(3.6, 68).$$

This empirical prior is summarized in Figure 3.

We next select priors for *bias and error in NMCP LLIN distribution data* (ϵ_{d_r} and σ_{d_r}). These are obtained by pooling data for the 17 country-years (c, t) where both survey distribution data $d_{c,t}$ and NMCP distribution data $d^r_{c,t}$ are available, and fitting a 2 parameter model consistent with the models for the likelihoods of NMCP LLIN flow data and survey LLIN flow above:

$$\log(d^r_{c,t}) \sim \text{Normal}\left(\log(d_{c,t}/(1-\overline{\pi_c})^{t_s-(t+.5)}) + \epsilon_c, \left(\sigma_{d^r_c}\right)^2 + \left((1.1)\sigma_{d_{c,t}}\right)^2\right).$$

To complete the model, we select uninformative hyper-priors

$$\epsilon_c \sim \text{Normal}(0, 1)$$
$$\sigma_{d^r_c} \sim \text{Exp}(1)$$

Sampling the posterior distribution with MCMC (220000 samples, of which the first 20000 were discarded, and the remaining were thinned by a factor of 20) yields a marginal posterior distribution of ϵ_c with mean .92 and standard deviation .22 and of $\sigma_{d^r_c}$ with mean 1.4 and standard deviation .17. We use normal distributions as empirical priors for ϵ_c and $\sigma_{d^r_c}$ with these parameters. In other words, as empirical priors for each country-specific model above, we take

$$\epsilon_c \sim \text{Normal}\left(.92, .22^2\right)$$
$$\sigma_{d^r_c} \sim \text{Normal}\left(1.4, .17^2\right)$$

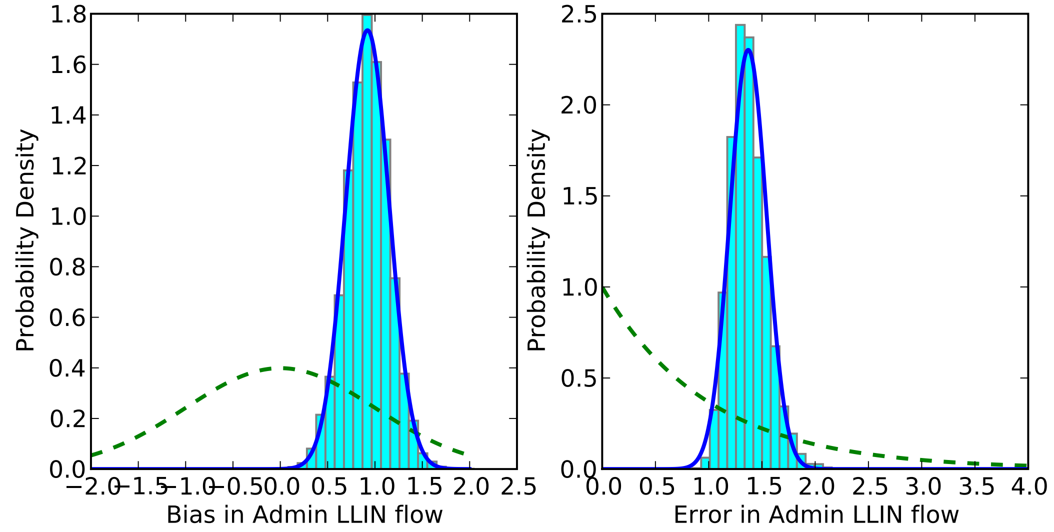

Figure 4. Empirical prior for NMCP Flow Bias and Error (ϵ_c and $\sigma_{d_c^r}$). Dashed green curves are the hyper-prior, cyan histogram shows the hyper-posterior, and the solid blue curve shows the empirical prior which is used for Bayesian inference in the compartmental model.

This empirical prior is summarized in Figure 4, and the data on which these priors are based is shown as a scatter plot in Figure 5.

To select empirical priors for the *ITN coverage parameter and ITN dispersion parameter* (η_c and α_c), we pool all data for country-years (c,t) where survey data provide estimates of both LLIN stock ($s_{c,t}$) and LLIN coverage ($\mathrm{LLIN}_{c,t}$). Both of these measurements have reliable estimates of uncertainty ($\sigma_{s_{c,t}}$ and $\sigma_{\mathrm{LLIN}_{c,t}}$), which we also make use of. We obtain empirical priors on η_c and α_c by fitting a model with latent $\Theta_{c,t}$ parameters:

$$\Theta_{c,t} \sim \mathrm{Normal}\left(s_{c,t}, \left(\sigma_{s_{c,t}}\right)^2\right)$$

$$\mathrm{LLIN}_{c,t} \sim \mathrm{Normal}\left(\Delta_{c,t}, \left(\sigma_{\mathrm{LLIN}_{c,t}}\right)^2\right)$$

To fit this model, we specify uninformative (hyper)-priors on η_c and α_c:

$$\eta_c \sim \mathrm{Normal}(5, 3^2)$$

$$\alpha_c \sim \mathrm{Exp}(1)$$

Sampling the posterior distribution with MCMC (220000 samples, of which the first 20000 were discarded, and the remaining were thinned by a factor of 20) yields a marginal posterior distribution of η_c with mean 4.0 and standard deviation .12 and of α_c with mean 3.0 and standard deviation 1.2. We use a normal distribution for the empirical prior for η_c and a gamma distribution for the empirical prior for α_c, with parameters to match these means and standard deviations. In other words, as empirical priors for each country-specific model above, we take

$$\eta_c \sim \mathrm{Normal}\left(4.0, .12^2\right)$$

$$\alpha_c \sim \mathrm{Gamma}\left(6.3, 2.1\right)$$

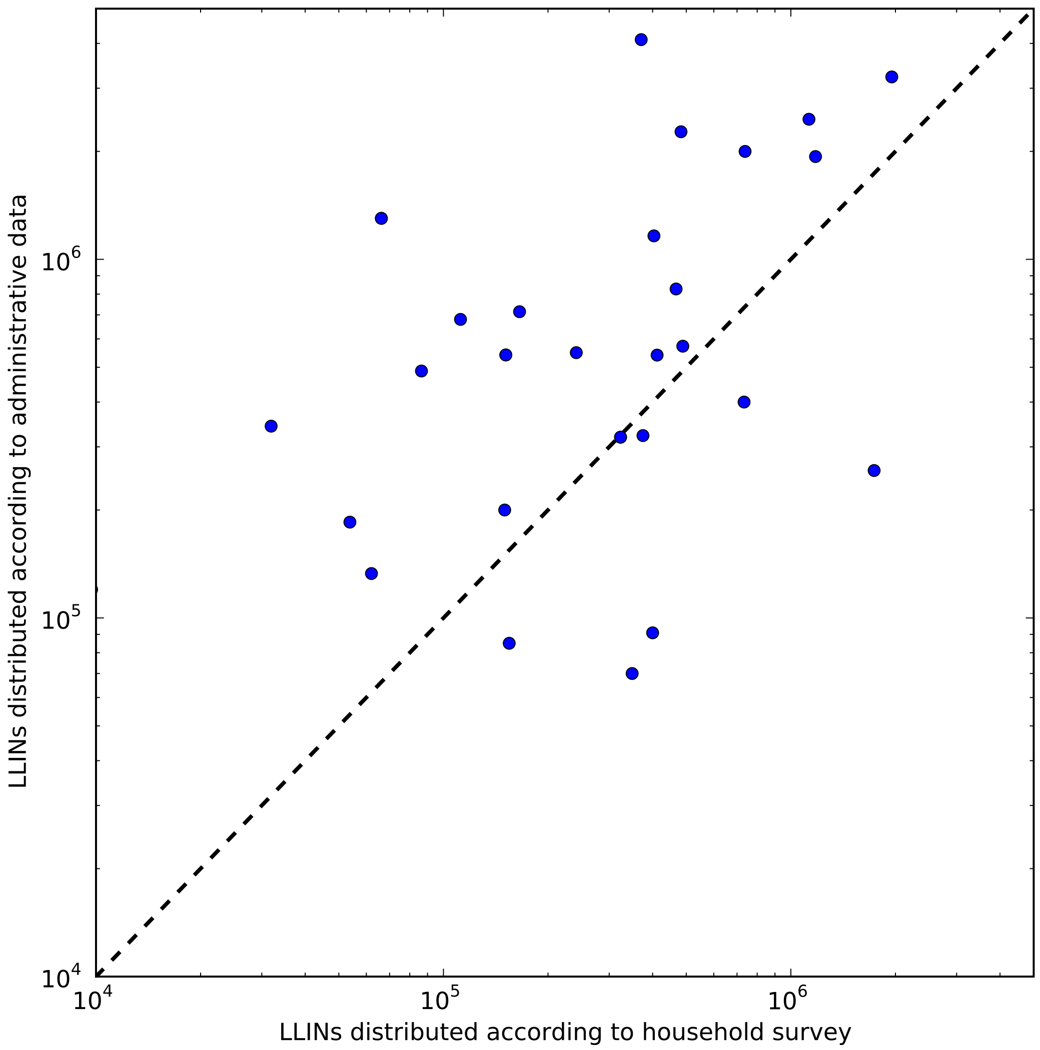

Figure 5. Data on LLINs distributed to households in country c during year t, as estimated from household surveys and NMCP reports. The dashed line shows the identity $y = x$, where points should fall if the household survey data and NMCP reports match.

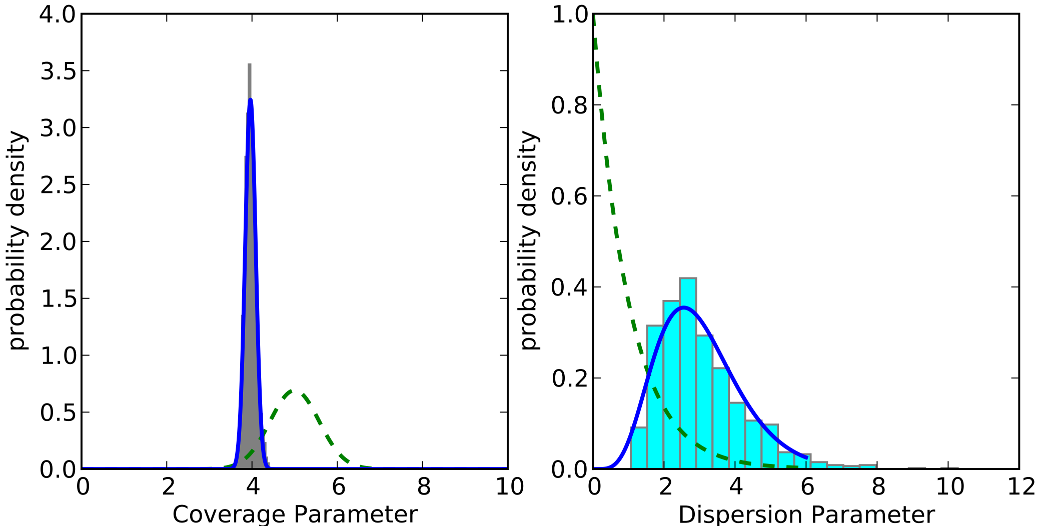

Figure 6. Empirical prior for Coverage Parameter and Dispersion Parameter (η_c and α_c). Dashed green curves are the hyper-prior, cyan histogram shows the hyper-posterior, and the solid blue curve shows the empirical prior which is used for Bayesian inference in the compartmental model.

These empirical priors are summarized in Figure 6.

Finally, to select the empirical prior for the *survey design effect*, we compare the sampling error assuming simple random sampling to the sampling error taking into account the complex survey design. This yields enough high-quality data that we simply use the mean and variance of the survey design effect to select the empirical prior:

$$\gamma_c \sim \text{Normal}(1.90, .37^2)$$

Figure 7 summarizes this.

4 MCMC approach to Bayesian inference

The previous sections completely defined the system dynamics model and statistical model used to harmonize all relevant sources of ITN data. This section describes the computational approach used to fit the model parameters.

We used the Python package PyMC [8], which performs Markov Chain Monte Carlo (MCMC) to draw samples from the model's posterior distribution. We ran a separate chain for each country model for $5,250,000$ steps, discarding the first $250,000$ steps as a "burn-in" period, and thinning the remaining samples by a factor of 1000 to ensure independence. This yields 5000 draws from the posterior distribution, from which we estimate the marginal mean and 95% uncertainty intervals of all model parameters of interest. For example, Figure 8 summarizes the posterior distributions of the exponentiated NMCP Flow Bias parameters (exponentiating the parameter maps it to a scale that is easier to interpret, as a bias factor).

Before this calculation is run, the empirical priors are generated, also by MCMC. These smaller models do not require as much burn-in or thinning, as described in Section 3.3 above.

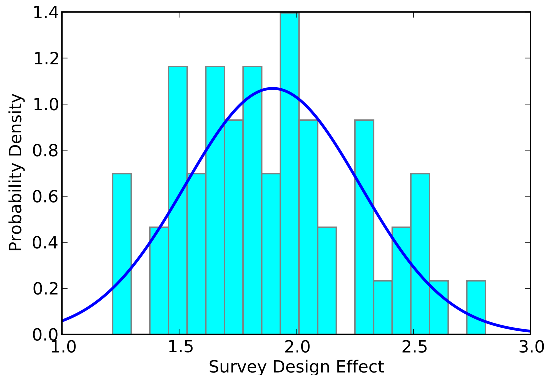

Figure 7. Empirical prior for Survey Design Effect (γ_c). Cyan histogram shows the survey design effect observed distribution, and the solid blue curve shows the empirical prior used for Bayesian inference in the compartmental model.

References

1. Poole DJ, Raftery AE (2000) Inference for deterministic simulation models: The Bayesian melding approach. Journal of the American Statistical Association : 1244-1255.

2. Alkema L, Raftery AE, Brown T (2008) Bayesian melding for estimating uncertainty in national HIV prevalence estimates. Sex Transm Infect 84: i11-16.

3. Ghys PD, Walker N, McFarland W, Miller R, Garnett GP (2008) Improved data, methods and tools for the 2007 HIV and AIDS estimates and projections. Sex Transm Infect 84: i1-4.

4. Ades AE, Welton NJ, Caldwell D, Price M, Goubar A, et al. (2008) Multiparameter evidence synthesis in epidemiology and medical decision-making. J Health Serv Res Policy 13: 12-22.

5. Devine O, Qualters J (July 2008) Bayesian updating of model-based risk estimates using imperfect public health surveillance data. Human and Ecological Risk Assessment 14: 696-713(18).

6. King G, Honaker J, Joseph A, Scheve K (2001) Analyzing incomplete political science data: An alternative algorithm for multiple imputation. American Political Science Review 95: 49-69.

7. Lim S, Stein D, Charrow A, Murray C (2008) Tracking progress towards universal childhood immunisation and the impact of global initiatives: a systematic analysis of three-dose diphtheria, tetanus, and pertussis immunisation coverage. The Lancet 372: 2031-2046.

8. Patil A, Huard D, Fonnesbeck C (in press) PyMC: Markov Chain Monte Carlo for Python, version 2.0. Journal of Statistical Software .

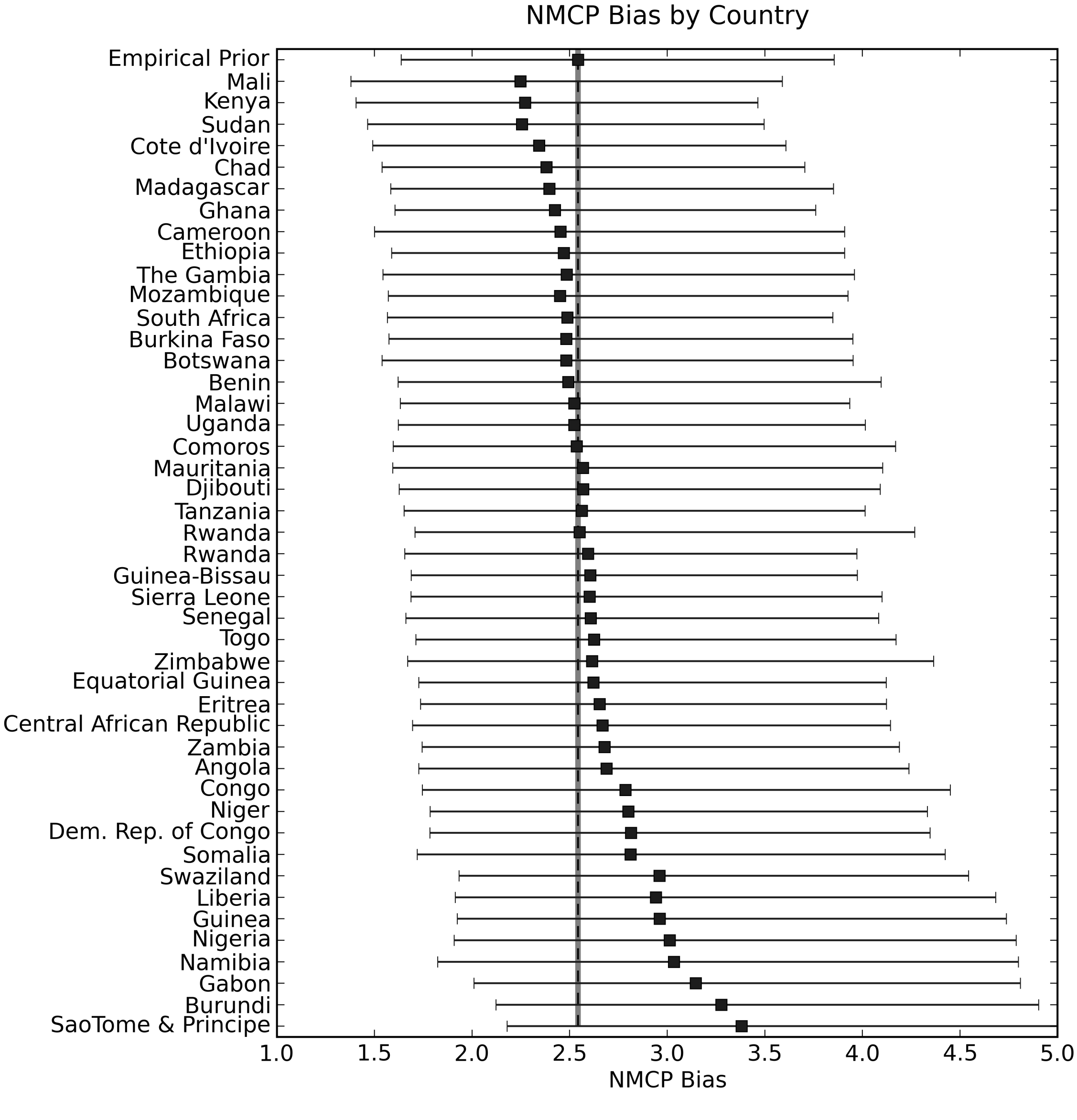

Figure 8. Posterior distributions for exponentiated NMCP Flow Bias parameters (e^{ϵ_c}) plotted by country. The empirical prior distribution shown first, for comparison.
